# Comparative Anatomy of the Bony Labyrinth (Inner Ear) of Placental Mammals

**DOI:** 10.1371/journal.pone.0066624

**Published:** 2013-06-21

**Authors:** Eric G. Ekdale

**Affiliations:** 1 Department of Biology, San Diego State University, San Diego, California, United States of America; 2 Department of Paleontology, San Diego Natural History Museum, San Diego, California, United States of America; University of Maryland, United States of America

## Abstract

**Background:**

Variation is a naturally occurring phenomenon that is observable at all levels of morphology, from anatomical variations of DNA molecules to gross variations between whole organisms. The structure of the otic region is no exception. The present paper documents the broad morphological diversity exhibited by the inner ear region of placental mammals using digital endocasts constructed from high-resolution X-ray computed tomography (CT). Descriptions cover the major placental clades, and linear, angular, and volumetric dimensions are reported.

**Principal Findings:**

The size of the labyrinth is correlated to the overall body mass of individuals, such that large bodied mammals have absolutely larger labyrinths. The ratio between the average arc radius of curvature of the three semicircular canals and body mass of aquatic species is substantially lower than the ratios of related terrestrial taxa, and the volume percentage of the vestibular apparatus of aquatic mammals tends to be less than that calculated for terrestrial species. Aspects of the bony labyrinth are phylogenetically informative, including vestibular reduction in Cetacea, a tall cochlear spiral in caviomorph rodents, a low position of the plane of the lateral semicircular canal compared to the posterior canal in Cetacea and Carnivora, and a low cochlear aspect ratio in Primatomorpha.

**Significance:**

The morphological descriptions that are presented add a broad baseline of anatomy of the inner ear across many placental mammal clades, for many of which the structure of the bony labyrinth is largely unknown. The data included here complement the growing body of literature on the physiological and phylogenetic significance of bony labyrinth structures in mammals, and they serve as a source of data for future studies on the evolution and function of the vertebrate ear.

## Introduction

The ear region, which functions in hearing via the cochlea as well as balance and equilibrium via the vestibule and semicircular canals, is one of the most intensively studied sensory systems in vertebrate anatomy and physiology. The external morphology of the petrosal bone, which surrounds the delicate structures of the inner ear in all mammals, is a common source of characters used in phylogenetic analyses [1]–[7]. Because petrosals preserve readily in the fossil record [8], the otic region is a valuable resource for paleontologists when making biological inferences about extinct mammals [9]–[17].

A link between body mass and dimensions of the inner ear have been hypothesized for several mammal groups, particularly primates [18], and inner ear dimensions that are normalized to body mass are hypothesized to correlate with both hearing [9], [19]–[22] and agility [23]–[26]. Such assertions begin with and are necessarily dependent on the comparative morphology of the bony labyrinth among extant mammals, for which physiological capabilities can be measured directly. However, the morphologies of the osseous chambers of the inner ear are unknown for many mammal species. The goal of this study is to document the structure of the bony labyrinth across a broad spectrum of placental mammals, to assess a link between inner ear anatomy and body mass, investigate high-level phylogenetic patterns, and explore the link between bony labyrinth morphology and inner ear function that has been hypothesized for individual clades [9], [19]–[26], although over a much wider taxonomic array of placental mammals.

**Figure 1:**
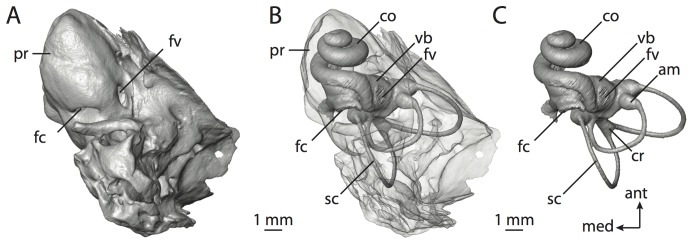
Petrosal of*Dasypus novemcinctus* (TMM M-1885) within which sits endocast of bony labyrinth. Each part of the figure are oriented the same with anterior towards top and medial towards left. **A**, tympanic view of petrosal bone; **B**, bone rendered semi-transparent to reveal bony labyrinth; **C**, endocast of bony labyrinth. Abbreviations listed at the end of the Materials and Methods section.

The internal cavities within the petrosal comprise the bony labyrinth of the inner ear, including the cochlea anteroventrally and the vestibular apparatus (with semicircular canals) posterodorsally (Figure 1). The dimensions of inner ear structures are correlated to the physiological capabilities of the otic region, including both hearing and balance. Ratios between measurements and the volume of the cochlea are related to auditory frequency limits [18]–[20], [27]–[28], which correlate with vocalization and social behavior, and the dimensions of the semicircular canals relate to the sensitivity of the canals [29], which may in turn correlate to agility and locomotor behaviors [23]–[26].

The labyrinth of the inner ear is difficult to study because the inner ear structures are completely surrounded by bone, and removal of this bone is necessary in order to observe the inner ear cavities (Figure 1). The structures of the inner ear can be exposed via dissolution of the surrounding bone [21], [30]–[31] or through serially sectioning the petrosal [32]–[33]. Alternatively, non-destructive techniques, such as high resolution X-ray computed tomography (CT), can be used to digitally image the internal cavities of the ear region [12], [25], [34].

Morphology of the inner ear is phylogenetically informative at both more- and less-inclusive taxonomic levels. For example, the cochlea completes at least one complete 360° turn in living therian mammals, but less in monotremes and more basal mammals [31], [35]–[36]. The bony labyrinth of Mesozoic therians exhibit ancestral morphologies, such as a fusion of the posterior and lateral semicircular canals to form a secondary common crus, which is lost in several clades within crown Theria [37]–[40]. Within Primates, dimensions of the semicircular canals and other labyrinthine elements reflect evolutionary and phylogenetic history [41]–[42], and further phylogenetic information can be found in the inner ear of squamate reptiles [43]–[47].

Given the functional and phylogenetic importance of this region of the skull, it is surprising that broad comparisons of the inner ear of mammals are lacking (the most notable exceptions are the seminal works of Hyrtl [48], Gray [30]–[31], [49], and Fleischer [50]). Furthermore, most authors, owing to the functional division between the cochlea and vestibular apparatus, decouple the structural continuity within the labyrinth. Functional studies therefore are restricted either to the cochlea and the sense of hearing [9], [18], [20]–[21], [28], [51]–[53], or to the vestibular apparatus and the sense of balance [24], [54]–[59]. Rarely is the labyrinth considered as a whole and compared across a large number of species (one exception is a morphometric study of strepsirrhine primates [41]). Such a comparison for the bony labyrinth of placental mammals is provided here, along with potential functional and phylogenetic considerations.

### Systematic Context of Descriptions

The morphological descriptions of the placental mammal bony labyrinth are arranged in a phylogenetic framework. As a point of departure for comparison, the bony labyrinth of a marsupial (*Didelphis virginiana*) is described. The opossum is considered in many respects to retain ancestral morphologies for Theria [60]–[63] (however, see discussion by Clemens [64]), and didelphids hold a basal position on the marsupial phylogeny [65]–[66]. Moreover, *Didelphis* commonly is used as a marsupial outgroup in phylogenetic analyses investigating placental relationships [2], [67]–[69]. Whereas certain aspects of the cranial morphology of the opossum are apomorphic (e.g., reduced pterygoids), comparisons of the bony labyrinth suggest the otic morphology largely is plesiomorphic [70].

**Figure 2:**
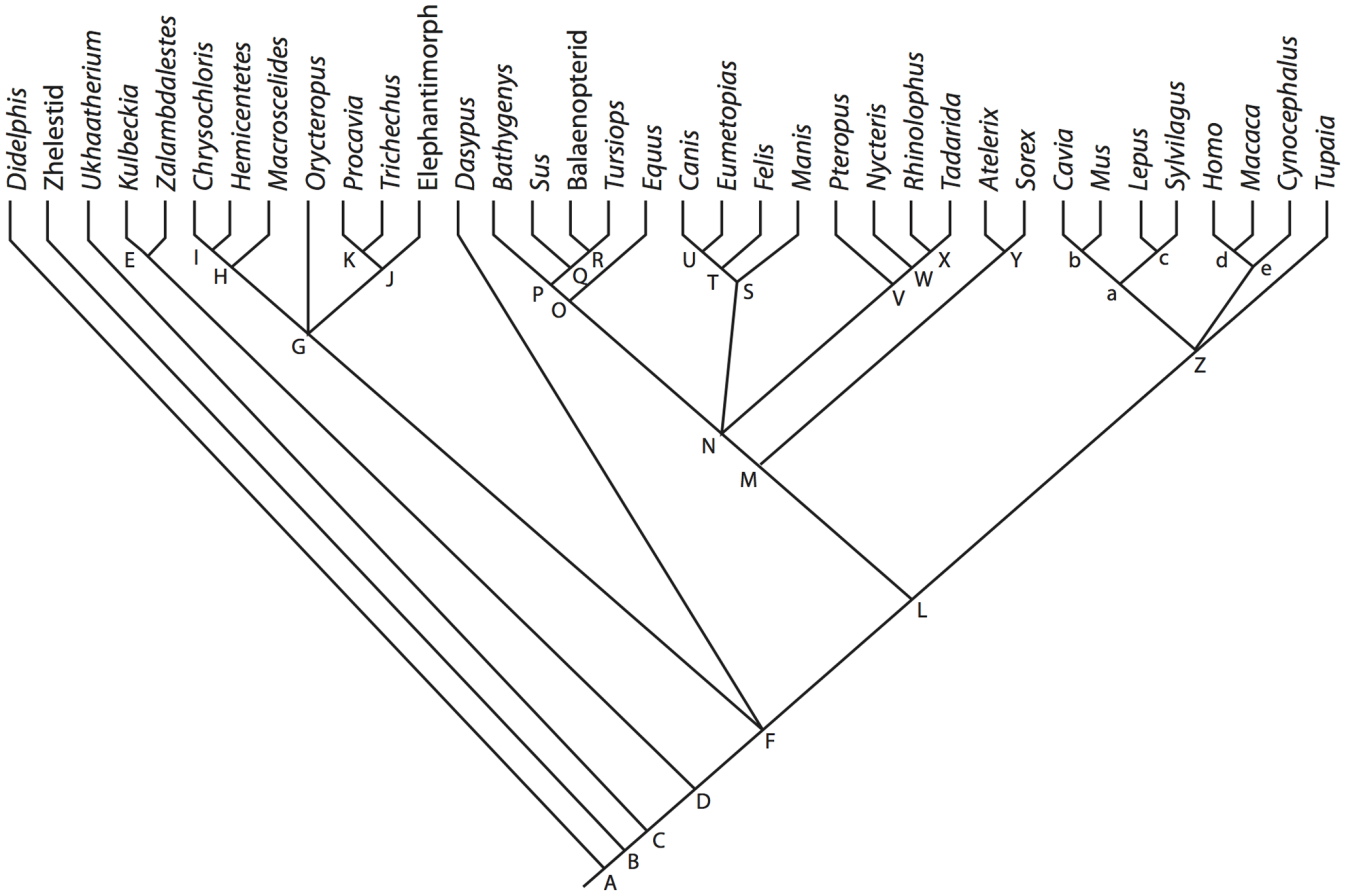
Cladogram of Theria including taxa considered. **Relationships follow published phylogenies** [6], [66]. Nodes: **A**, Theria; **B**, Eutheria; **C**, *Ukhaatherium*+Zalambdalestidae+Placentlia; **D**, Zalambdalestidae+Placentalia; **E**, Zalambdalestidae; **F**, Placentalia; **G**, Afrotheria; **H**, Afrosoricida+*Macroscelides*; **I**, Afrosoricida; **J**, Paenungulata; **K**, *Procavia*+*Trichechus*; **L**, Boreoeutheria; **M**, Laurasiatheria; **N**, Cetartiodactyla+Perissodactyla+Ferae+Chiroptera; **O**, Cetartiodactyla+Perissodactyla; **P**, Cetartiodactyla; **Q**, *Sus*+Cetacea; **R**, Cetacea; **S**, Ferae; **T**, Carnivora; **U**, Caniformia; **V**, Chiroptera; **W**, Microchiroptera; **X**, *Rhinolophus*+*Tadarida*; **Y**, Eulipotyphla; **Z**, Euarchontoglires; **a**, Glires; **b**, Rodentia; **c**, Lagomorpha; **d**, Primatomorpha; **e**, Primates.

From *Didelphis*, descriptions of the labyrinths of eutherians (which includes crown Placentalia and all extinct therians more closely related to Placentalia than its extant sister taxon, Marsupialia) are arranged taxonomically following the relationships recovered for Mesozoic non-placental eutherians [6] and extant placentals [66]. The relationships recovered in previous studies and used here are based on extensive taxonomic sampling, and a composite tree following the results of those studies is illustrated in Figure 2.

The descriptions of the bony labyrinths of crown placental mammals begin with Afrotheria, and follow in order with Xenarthra, Laurasiatheria, and Euarchontoglires (see Figure 2). The descriptions are organized taxonomically within these major divisions to allow the reader skip ahead to the account of the species in which he or she is interested. Table S1 is a complete list of species examined with CT scanning parameters, and Table S2 includes further information and additional sources of data for many of the specimens. Each section begins with an overview of the inner ear morphology of the larger clade (e.g., Theria, Afrotheria) to serve as a ground plan to which the specific descriptions can be compared. Ancestral character states are reconstructed (inferred rather than measured) based upon the described morphology presented here and reported in the text and Table S3.

## Materials and Methods

### Specimens

At least one representative of the major clades of placental mammals recovered by the phylogenetic analyses of Bininda-Emonds and others [66] (see Figure 2) was selected based on availability of specimens in the Texas Memorial Museum Recent mammal collection at the Texas Natural Science Center at the University of Texas at Austin (where the research was performed; all specimens consisting of skeletal material were accessioned into the collections prior to the study and were lent to me). In addition, CT data of many taxa were made available to me from “Digital Morphology: a National Science Foundation Digital Library at The University of Texas at Austin” (www.digimorph.org). No live animals were used for any part of the research reported here. All specimens used in this study are listed in Table S1, along with institutional abbreviations. Anatomical and orientation terminology follows that of previous researchers [9], [71]–[72].

Whenever possible, isolated petrosal bones were CT scanned to maximize the resolution of the images (CT methods described below). The left petrosal was examined for each taxon, with a few exceptions, for consistency. Although cranial asymmetry is known within the ear region [73], the physiological significance of such asymmetry is poorly understood, and the results of recent studies suggest that such asymmetry may not be significant [74]. Images of the bony labyrinth are reversed in figures in the cases where right petrosals were used instead of elements from the left side of the skull for easy visual comparison (as noted in captions).

All specimens were presumed mature, although no rigorous assessment of maturity was ascertained. Although the external surface of the petrosal changes through accretionary growth, there is evidence that the structures of the inner ear do not change significantly once the walls of the bony labyrinth have ossified [75]–[76]. Based on those studies, the maturity of individuals used here should not affect the observed morphology. Because post-ossification changes in the bony labyrinth have been investigated for the rabbit [76], the human [77]–[78], and the marsupial *Monodelphis domestica* [75] only and not all of Mammalia, the overall consistency of this pattern among all therian mammal species cannot be assessed. Such a survey is beyond the scope of the current study, and it is assumed that any variation in the mature and fully ossified bony labyrinths used in the following comparisons does not affect the observation of characters at the gross morphological level.

### Computed Tomography Methods

Digital images obtained through computed tomography (CT) was employed to observe the internal chambers of the petrosal that constitute the bony labyrinth. The majority of the specimens used for this study were scanned at the University of Texas High-resolution X-ray CT facility (UTCT) in Austin, TX. The only specimen not scanned at UTCT was *Trichechus manatus* (MSW 03156), which was scanned at Washington University in Saint Louis, MO. Parameters for CT scanning and post-scanning image processing are provided in Table S1.

The bony labyrinths were digitally segmented from the CT image data into the various partitions of the inner ear (e.g., cochlea and vestibule) in order to calculate partial volumes of the osseous cavities, as well as create a 3-dimensional representation of the bony labyrinth for visualization purposes. Segmentation was performed in the computer software packages VGStudio Max 1.2 ^©^ [79] and Amira 3.1 ^©^ [80] (currently distributed by Visualization Sciences Group – an FEI Company). The bony channels for the vestibular and cochlear aqueducts were included in the segments of the cochlea and vestibule respectively. The boundaries between the compartments were kept as planar as possible. The medial border of the fenestra vestibuli was used as the dividing line between the cochlea and vestibule, where the entire fenestra is included within the segmented vestibule. Determination of the air-to-bone boundary during segmentation was accomplished visually, modified from the half-maximum height protocol [27].

The endocasts constructed for this study are oriented with the plane of the lateral semicircular canal parallel to the horizon. Such an orientation was selected because the lateral semicircular canal usually is held horizontal when the animal is in a state of alertness [81]. However, the lateral canal is not parallel to the earth-horizon at all times in every animal [82]. Nonetheless, a standard position is used here to aid in comparison. Anterior view is oriented down a line connecting the ampullar aperture of the lateral semicircular canal and the vestibular aperture of the posterior limb of the lateral canal (or vestibular aperture of the posterior ampulla if the canal does not open directly into the vestibule at its posterior end). The labyrinth is oriented with respect to this anterior position in all other views.

### Measurement Methods

**Figure 3:**
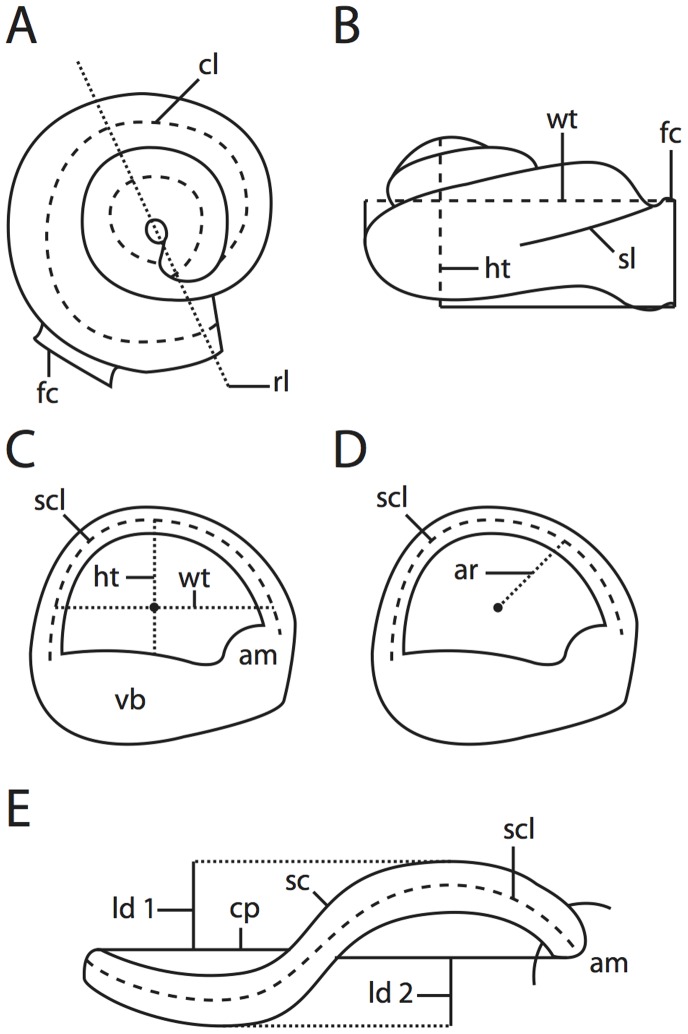
Measurement methods employed. **A**, coiling of cochlea; **B**, height and width of cochlea used for calculation of aspect ratio; **C**, height, width, and length of semicircular canal; **D**, arc radius of curvature calculated from height and width of arc; **E**, linear deviation of semicircular canal, used with arc radius of curvature to calculate angular deviation. Abbreviations listed at the end of the Materials and Methods section.

Angular, linear, and volumetric measurements were made in the Amira software [80], and brief descriptions and illustrations of the measurements are provided here (Figure 3). Methodologies for various measurements follow earlier studies [1], [9], [34], [77], and a more expanded discussion of measurement methodology is provided elsewhere [75], [83]–[84]. Volumes for individual compartments within the internal cavities and the linear length of the bony labyrinth were measures of overall size of the labyrinth. The degree of coiling exhibited by the cochlea is reported (in degrees) along with the total number of completed turns (calculated as total degrees divided by 360°; “Coil-96″ of Ekdale and Rowe [83]). A shape index (aspect ratio) of the cochlear spiral was calculated by dividing the height of the spiral by the width of the basal turn (Figure 3B). A high aspect ratio is considered to be above 0.55, following previous observations of “flattened” and “sharp-pointed” cochleae [30]–[31], where “flattened” cochleae have an aspect ratio 0.55 and below.

The total length of the cochlear canal from base to apex was measured using the SplineProbe tool in the Amira software [80]. The length of the cochlear canal approximates the length of the soft-tissue basilar membrane, upon which the spiral organ of hearing sits, which may correlate to audible frequencies [21], [27].

Angles were taken between the planes of all of the semicircular canals, as well as between the basal turn of the cochlea and the plane of the lateral semicircular canal, when the planes were oriented perpendicular to the field of view [75]. The lengths of the slender (unampullated) portions of the semicircular canals [85] were measured using the SplineProbe tool in the Amira software, similar to the method used for measuring the length of the cochlear canal. The radius of curvature of a semicircular canal (the dimension “R” of Jones and Spells [54] and Spoor and Zonneveld [34]) is calculated as half the average between the height and width of the canal arc (Figures 3C–D). The size of the semicircular canal arc is correlated to the afferent sensitivity of the canal [29].

The total angular deviation of a semicircular canal from its respective plane is calculated trigonometrically using two linear measurements of the canal (adapted from previous methods [56], ). The linear measures utilized in the calculation of angular deviation are the arc radius of curvature of the canal and total linear deviation of the canal from its plane (Figure 3E) [73]. Substantial deviation of a semicircular canal from its plane is defined here as any ratio of the total linear deviation over the cross-sectional diameter at the midpoint of the semicircular canal being greater than 1. In short, if the total linear deviation of a canal from its plane is greater than the diameter of the canal in cross-section, the deviation is considered substantial, in a non-statistical sense. The measure of substantial deviation is arbitrary and does not have any basis in the physiology of the semicircular canal system. The functional implications of non-planar semicircular canals are poorly understood, and the substantial values are intended to emphasize the phenomenon of non-planarity, rather than to make any functional interpretations at this time.

The sagittal labyrinthine index, which is defined as the percentage of the width of the posterior semicircular canal arc below the plane of the lateral semicircular canal [34], quantifies the relative positions of the lateral and posterior semicircular canals. High sagittal labyrinthine indices separate the great apes from other primates [42], and the index might be useful in the phylogenetic assessment of other mammal groups.

Several additional indices were calculated, including the ratio of the length of the slender portion of a semicircular canal over arc radius and the aspect ratio (height over width) of the arcs of the vertical (anterior and posterior) semicircular canals. These ratios have been hypothesized to distinguish aquatic species from their terrestrial ancestors [25], [85]. The stapedial ratio describes the shape of the footplate of the stapes [87], which contacts the inner ear spaces via the fenestra vestibuli. Marsupial mammals tend towards circular fenestrae with ratios below 1.8, whereas the fenestrae of placentals tend to be more elliptical. In absence of the stapedial footplate, the dimensions of the fenestra vestibuli can be used.

To ascertain whether the dimensions of the inner ear are correlated to overall body size of the animal, specific measurements were plotted over body mass (all data logarithmically transformed using the natural log) and the coefficient of correlation (“r”) was calculated in Microsft Excel 2008 for Macintosh. At an *a priori* significance level of 5% (*P = *0.05) based on the current sample size for which body masses are known (N = 28), any coefficient of correlation 0.38 or above is considered significant using a two-tailed probability model, which is most common in statistical analyses [88]. However, a correlation that is found to be statistically significant might not be strong (i.e., a relationship might exit, but the influence of body mass might be low), so coefficients of determination (r^2^) were calculated also to determine the strength of recovered correlations. The coefficient of determination reports the percentage of variation in variable Y that can be explained by X, and coefficients of correlation above 0.70 are considered strong in this study (i.e., situations in which over half of the variation in a bony labyrinth dimension can be explained by body mass).

If the body mass of the specimen examined was not known, an average was calculated from various published sources [89]–[91]. Body mass data are unavailable for the fossils examined (*Bathygenys reevesi* and Balaenopteridae), and a body mass was not used for *Canis familiaris* given the broad range of body masses observed in domestic dogs [92]–[93]. Further correlations were investigated between dimensions of the cochlea, as well as dimensions of the semicircular canals.

### Phylogenetic Methods

A phylogenetic analysis was not performed for this study owing to the restricted anatomical region in question and the level at which the taxa were sampled. However, several characters that have been used in phylogenetic analyses, and those are described for the taxa below. Ancestral states, both continuous and discrete, for the hypothetical common ancestors of the clades pictured in Figure 2 were reconstructed in the computer program Mesquite [94]. The ancestral state reconstructions were inferred based upon the data presented in the current paper, although the presented anatomical descriptions are compared with those from the published literature. Although discrete character states of ancestors were reconstructed using the parsimony method in the Mesquite software, the maximum likelihood method was utilized for continuous characters [95]. Characters that were traced across the cladogram are (1) entry of the lateral semicircular canal into a secondary common crus, the posterior ampulla, or the vestibule, (2) position of the lateral semicircular canal with respect to the posterior semicircular canal, (3) largest semicircular canal arc among the anterior, lateral, and posterior canals, (4) aspect ratio of the cochlear spiral in profile, (5) degree of cochlear coiling, and (6) contribution (percentage) of the volume of the cochlea to the entire labyrinth. Ancestral states for nodes are provided in Table S3. Matrices can be found at Morphobank.org Project P833 (http://morphobank.org/index.php/Projects/ProjectOverview/project_id/833). The matrices include discrete characters only (within the “Matrices” section of the project page), continuous characters only, and a combined discrete and continuous character matrix (the latter two can be downloaded within the “Documents” section of the project page).

### Abbreviations in Figures

Abbreviations used in figures: **aa**, anterior ampulla; **ac**, anterior semicircular canal; **am**, ampulla; **ant**, anterior direction; **ar**, semicircular canal arc radius of curvature; **av**, bony channel for vestibular aqueduct; **cc**, canaliculus cochleae for cochlear aqueduct; **cf**, foramina within cribriform plate; **cl**, length of cochlear canal; **cn**, canal for cranial nerve VIII; **co**, cochlea; **cp**, plane of semicircular canal; **cr**, common crus; **cv**, canal for cochlear vein; **dor**, dorsal direction; **er**, elliptical recess of vestibule; **fc**, fenestra cochleae; **fn**, canal for cranial nerve VII; **fv**, fenestra vestibuli; **hf**, hiatus Fallopii for exit of greater petrosal nerve; **ht**, height; **iam**, internal auditory meatus; **in**, incus; **la**, lateral ampulla; **lat**, lateral direction; **lc**, lateral semicircular canal; **ld**, linear deviation of semicircular canal from its plane; **ma**, malleus; **med**, medial direction; **pa**, posterior ampulla; **pc**, posterior semicircular canal; **pf**, perilymphatic foramen; **pl**, primary bony lamina; **pos**, posterior direction; **pr**, promontorium; **ps**, outpocketing for perilymphatic sac; **rl**, reference line for measuring coiling of cochlea; **sa**, subarcuate fossa; **sc**, semicircular canal; **scr**, secondary common crus; **sg**, canal for spiral ganglion within primary bony lamina; **sl**, secondary bony lamina; **sr**, spherical recess of vestibule; **st**, stapes; **vb**, vestibule; **ven**, ventral direction; **vn**, canal for vestibular branch of cranial nerve VIII; **wt**, width.

## Results

### Gross Anatomy of Inner Ear

The inner ear of mammals consists of a set of interconnected spaces within the petrosal bone known as the bony labyrinth (Figure 1), which contain a series of soft-tissue sacs and ducts known as the membranous labyrinth. The membranous labyrinth is separated into an inferior division that includes the cochlear duct (containing the spiral organ of hearing) and saccule of the vestibule (containing receptors sensitive to linear motion), and a superior division that includes the utricle of the vestibule, the anterior, lateral, and posterior semicircular ducts and ampullae, and the common crus between the anterior and posterior ducts (all of which are involved with detecting rotational movement of the head). The osseous semicircular and cochlear canals of the bony labyrinth mirror the shape of the membranous ducts within, although the bony canals may not accurately reflect the size of the ducts [96].

The bony cochlear canal is divided within the promontorium of the petrosal into the scala tympani that communicates with the fenestra cochleae (which is covered with a secondary tympanic membrane to accommodate pressure changes within the membranous labyrinth in life), and the scala vestibuli that terminates at the fenestra vestibuli (which accommodates the footplate of the stapes). The division is formed by a bony primary spiral lamina that curves along the modiolus (central bony pillar around which the cochlea coils) on the axial (inner) wall of the cochlea. A secondary bony lamina often mirrors the primary lamina for a short distance on the opposing (radial) wall of the cochlea. The two laminae are connected by the basilar membrane (the laminae do not contact each other directly), upon which the spiral organ of hearing sits. The basilar membrane defines the tympanic wall of the membranous cochlear duct (also known as the scala media). The vestibular membrane crosses the width of the scala vestibuli to complete the cochlear duct at its vestibular edge. A small opening known as the helicotrema is situated at the apex of the cochlea, and it serves as a connection between the scalae tympani and vestibuli. The cochlear duct is filled with endolymph, and the surrounding space, which includes both the scala tymapni and the scala vestibuli, is filled with perilymph. The perilymphatic duct exits the inner ear near the fenestra cochleae through a bony passage known as the canaliculus cochleae.

The bony vestibule is divided into the spherical recess inferiorly and the elliptical recess superiorly. The recesses correspond loosely to the saccule (spherical recess) and utricle plus semicircular ducts (elliptical recess), but the shapes of the membranous sacs are preserved minimally within the bony vestibule. The saccule, utricle, and semicircular ducts are filled with endolymph (exchange occurs between the cochlea and saccule at their junction), and perilymph fills the remainder of the space. Varying amounts of perilymph surround the semicircular ducts in different species [30]–[31], [49], and the endolymphatic duct exits the inner ear from the medial edge of the labyrinths, passing through the bone and opening into the cranial cavity.

### Anatomical Comparisons

#### Theria

**Figure 4:**
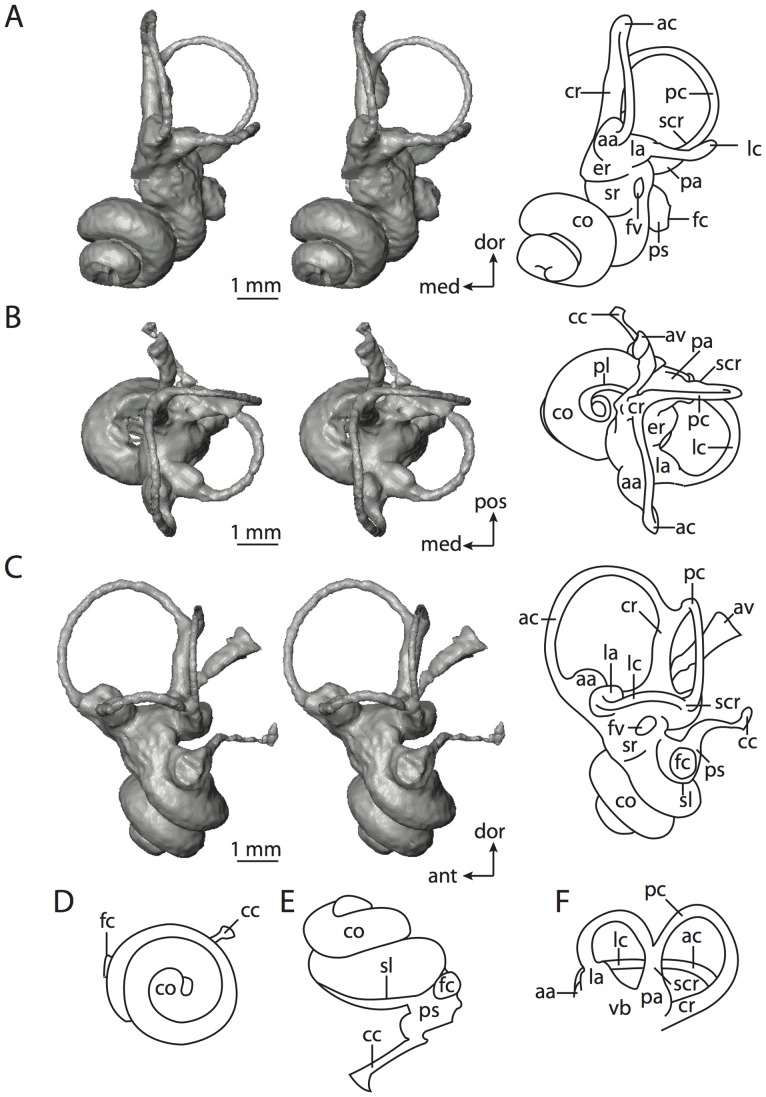
Bony labyrinth of*Didelphis virginiana*. **A**, stereopair and labeled line drawing of digital endocast in anterior view; **B**, stereopair and labeled line drawing of digital endocast in dorsal view; **C**, stereopair and labeled line drawing of digital endocast in lateral view; **D**, line drawing of cochlea viewed down axis of rotation to display degree of coiling; **E**, line drawing of cochlea in profile; **F**, vestibular apparatus displaying secondary common crus. Abbreviations listed at the end of the Materials and Methods section.

**Figure 5:**
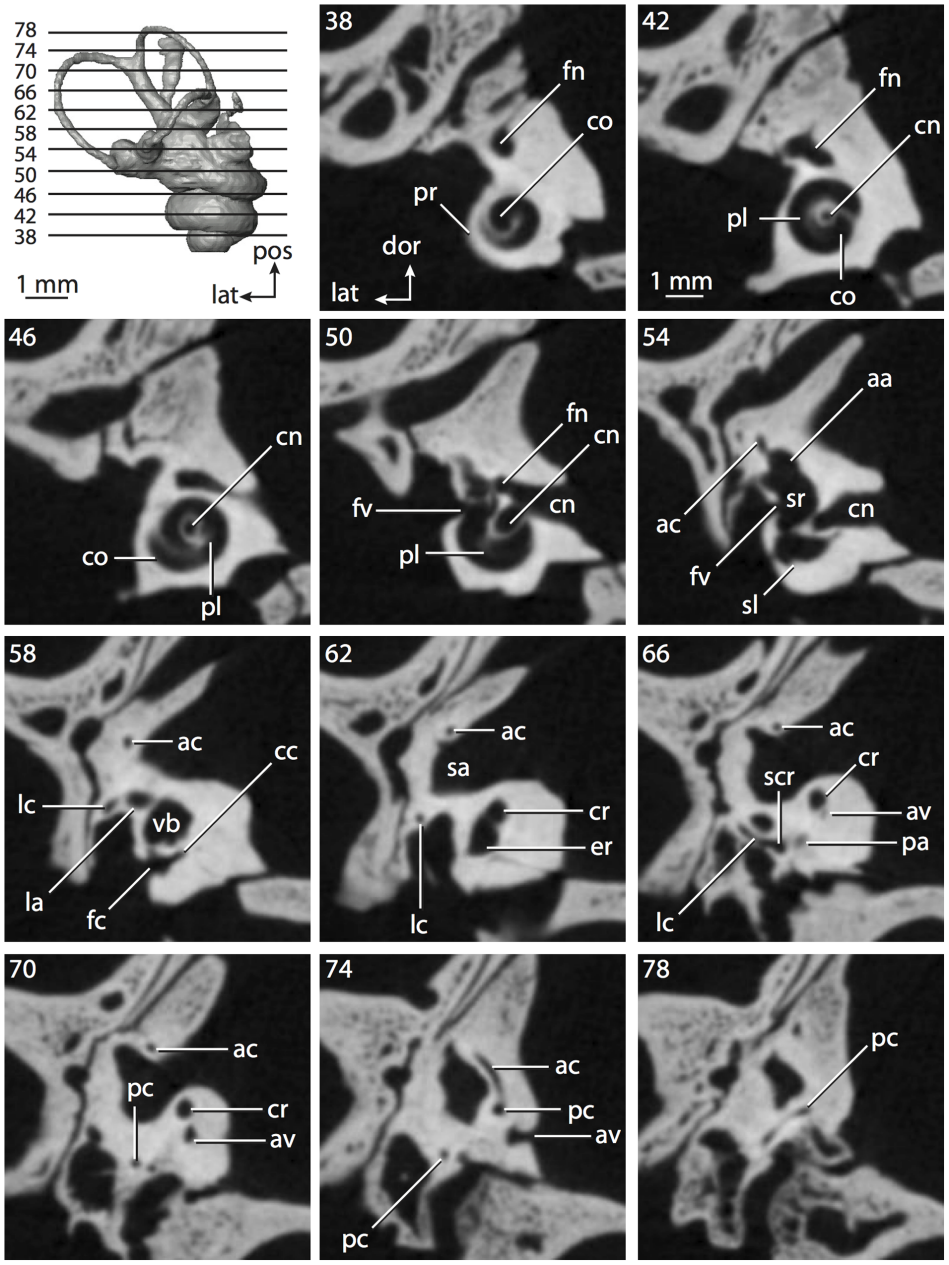
Original CT slices through ear region of*Didelphis virginiana.* Numbers refer to specific CT slices. Abbreviations listed at the end of the Materials and Methods section.

Theria includes the most recent common ancestor of extant marsupials (such as *Didelphis virginiana*, which is used to represent Marsupialia) and extant placentals (such as *Homo sapiens*) and all of the descendents of that ancestor. As inferred from ancestral state reconstructions based on the data presented in this paper, the bony labyrinth of the hypothetical therian ancestor possessed a secondary common crus formed between the lateral and posterior semicircular canals (see the labyrinth of *Didelphis* in Figures 4 and 5). The secondary common crus likely was inherited from a much more distant mammalian ancestor [16].

The plane of the lateral canal is positioned low with respect to the ampullar entrance of the posterior canal so that the area of the arc of the posterior canal is not divided by the lateral canal in anterior view, as it is in most extant placentals (e.g., *Chrysochloris* or *Sylvilagus*). As observed in most mammal species, the arc of the anterior semicircular canal is the largest among the three canals. Ancestral state reconstructions based on the specimens examined for this study indicate that the cochlea completes a 685° coil (nearly two turns). However, as discussed below in the descriptions of the bony labyrinths of *Didelphis virginiana* and *Kulbeckia kulbecke*, the cochlea of the therian ancestor likely completed a single 360° turn only [11], [83]. The overestimate of the cochlear coiling in the ancestral therian likely is the result of the broad range of cochlear coiling in extant taxa (as described throughout the remainder of the paper) and the algorithm used to reconstruct ancestral states (see Materials and Methods).

The cochlea contributes 66% of the total inner ear volume. The aspect ratio of the cochlea of the ancestral therian is reconstructed as low, although the aspect ratio in *Didelphis* (0.62) is higher than that calculated for basal taxa along the eutherian lineage (e.g., the zalambdalestid *Kulbeckia kulbecke*). The ancestor of Theria likely possessed a cochlea with a low aspect ratio given the close similarities between basal metatherian and eutherian labyrinths [11], and the ancestral state is reconstructed as such.

#### Marsupialia

The structure of the inner ear of *Didelphis virginiana* is described for comparison with the inner ear structures of crown placentals and their Mesozoic eutherian relatives. Dimensions of the bony labyrinth as a whole structure of *Didelphis* (and all other taxa) are provided in Table 1. Dimensions of the cochlea are provided in Table 2, and dimensions and orientations of the semicircular canals are reported in Tables 3–5.

**Table 1:** Body mass and dimensions of skull and bony labyrinth^a^.

Taxon		BM (g)	SL (mm)	BLV (mm^3^)	BLL (mm)
Marsupialia					
	*Didelphis*	2800	107	12.1	5.15
Eutheria					
	*Kulbeckia*	NA	NA	5.37	4.73
	*Ukhaatherium*	NA	NA	2.17	3.57
	*Zalambdalestes*	NA	NA	6.07	5.31
	Zhelestid	NA	NA	6.28	4.51
Afrotheria					
	*Chrysochloris*	44.4	23.9	4.11	3.93
	Elephantimorpha	NA	NA	1145	26.0
	*Hemicentetes*	110	30.6	2.78	4.08
	*Macroscelides*	38.4	32.3	9.19	4.31
	*Orycteropus*	60000	245	107	15.0
	*Procavia*	3800	75.9	19.4	8.50
	*Trichechus*	500000	NA	621	19.3
Xenarthra					
	*Dasypus*	4754	NA	26.5	8.06
Laurasiatheria					
	*Atelerix*	866	38.1	4.58	5.46
	Balaenopteridae	NA	NA	1076	19.7
	*Bathygenys*	NA	90.7	29.8	7.40
	*Canis*	NA	87.1	31.4	8.10
	*Equus*	258324	530	165	16.5
	*Eumetopias*	735000	NA	139	13.7
	*Felis*	3408	NA	45.8	8.91
	*Manis*	4500	75.1	28.5	6.66
	*Nycteris*	29.3	27.8	2.13	3.39
	*Pteropus*	435	65.2	7.01	6.19
	*Rhinolophus*	17.2	24.3	5.89	3.76
	*Sorex*	6.07	16.9	0.81	2.81
	*Sus*	88286	240	61.9	9.95
	*Tadarida*	12.1	NA	3.86	3.22
	*Tursiops*	179500	543	168	10.1
Euarchontoglires					
	*Cavia*	728	67.8	22.2	7.13
	*Cynocephalus*	1000	NA	20.3	7.17
	*Homo*	8000	NA	165	16.3
	*Lepus*	2350	94.4	24.3	7.39
	*Macaca*	4668	NA	41.6	11.2
	*Mus*	15.5	20.8	1.47	2.71
	*Sylvilagus*	1160	68.7	11.3	5.82
	*Tupaia*	131	49.6	9.83	6.67

a Abbreviations: BM, body mass; SL, skull length; BLV, bony labyrinth volume; BLL, bony labyrinth length. Values for extinct eutherians are averages [83].

**Table 2:** Dimensions and orientations of the cochlea of placentals^a^.

Taxon	Volume	Coil	2° Lamina	Length	Aqueduct	Ratio	Angle
Marsupialia							
* Didelphis*	8.30	791	427	7.54	1.68	0.62	19.6
Eutheria							
* Kulbeckia*	2.59	446	209	4.93	0.60	0.44	12.1
* Ukhaatherium*	1.23	380	76.8	2.77	0.36	0.35	6.63
* Zalambdalestes*	2.91	368	95.3	3.40	0.48	0.36	13.5
Zhelestid	4.15	545	198	4.93	0.37	0.46	34.0
Afrotheria							
* Chrysochloris*	2.93	1191	301	6.65	0.45	0.63	41.9
Elephantimorpha	351	765	NA	32.5	NA	0.42	48.5
* Hemicentetes*	1.39	540	240	3.79	0.28	0.38	18.4
* Macroscelides*	6.59	720	334	7.11	0.58	0.80	25.1
* Orycteropus*	59.3	709	390	14.9	4.82	0.45	31.9
* Procavia*	9.24	1363	190	15.0	1.21	0.72	45.4
* Trichechus*	442	407	NA	22.5	NA	0.55	27.7
Xenarthra							
* Dasypus*	17.5	816	383	11.2	1.17	0.63	17.9
Laurasiatheria							
* Atelerix*	2.28	624	240	4.99	0.77	0.69	53.8
Balaenopteridae	974	886	238	53.0	3.65	0.48	23.2
* Bathygenys*	16.2	667	NA	8.51	NA	0.32	26.8
* Canis*	20.7	1156	104	13.9	2.08	0.64	20.8
* Equus*	84.3	900	153	22.1	11.3	0.41	37.9
* Eumetopias*	74.2	795	249	19.3	4.16	0.68	31.6
* Felis*	31.1	1092	243	16.8	3.60	0.69	45.8
* Manis*	14.0	863	NA	9.64	2.85	0.54	20.3
* Nycteris*	1.42	795	316	6.66	0.66	0.61	47.2
* Pteropus*	4.13	656	335	7.66	0.73	0.61	36.2
* Rhinolophus*	5.24	1115	935	11.6	0.59	0.63	5.5
* Sorex*	0.37	493	179	2.52	0.23	0.47	9.41
* Sus*	36.3	1274	NA	22.9	2.64	0.71	23.8
* Tadarida*	2.80	752	659	6.95	0.12	0.52	29.2
* Tursiops*	157	661	396	24.0	6.47	0.47	21.3
Euarchontoglires							
* Cavia*	12.3	1457	195	13.4	2.52	1.29	35.1
* Cynocephalus*	9.83	954	65.4	12.2	0.90	0.50	34.6
* Homo*	71.5	889	22.2	22.5	10.9	0.36	62.4
* Lepus*	13.1	693	147	8.80	1.34	0.64	40.6
* Macaca*	21.0	1088	81.0	16.9	3.53	0.48	47.8
* Mus*	0.86	628	327	3.87	0.17	0.62	10.8
* Sylvilagus*	6.26	817	200	8.75	1.05	0.71	40.3
* Tupaia*	5.43	1125	220	10.5	0.66	0.66	28.9

a Measurements: Volume, total volume of cochlear canal (mm^3^); Coil, the total degrees completed by the cochlea; 2° Lamina, extension of secondary lamina through cochlea (°); Length, length of canal (mm); Aqueduct, length of cochlear aqueduct (mm); Ratio, aspect ratio calculated as height of spiral over width; Angle, formed between basal turn of cochlea and lateral semicircular canal (°). Values for extinct eutherians are averages [83].

**Table 3:** Dimensions of vestibular elements and orientations of semicircular canals^a^.

Taxon	Aqueduct Length	Stapedial Ratio	Labyrinth Index	Semicircular Canal Angles		
				A–L	A–P	L–P
Marsupialia						
* Didelphis*	2.58	1.6	0.0	109	102	104
Eutheria						
* Kulbeckia*	1.24	2.0	0.0	79.9	79.9	89.6
* Ukhaatherium*	NA	1.5	0.0	88.8	105	88.4
* Zalambdalestes*	1.74	1.7	0.0	81.0	93.6	85.6
Zhelestid	1.06	1.6	0.0	88.8	96.8	93.1
Afrotheria						
* Chrysochloris*	0.37	2.8	21.7	65.6	86.9	96.7
Elephantimorpha	13.9	1.6	0.0	66.3	73.7	92.6
* Hemicentetes*	NA	1.6	4.1	79.3	87.9	87.0
* Macroscelides*	2.08	1.9	32.7	100	90.7	95.7
* Orycteropus*	8.25	1.8	0.0	78.5	91.9	87.4
* Procavia*	3.39	2.1	44.9	87.4	112	86.3
* Trichechus*	12.3	1.6	0.0	52.2	84.9	77.5
Xenarthra						
* Dasypus*	2.63	1.7	23.0	62.4	67.7	87.3
Laurasiatheria						
* Atelerix*	NA	1.8	26.4	82.2	91.7	92.1
Balaenopteridae	3.83	1.5	0.0	71.6	105	75.6
* Bathygenys*	NA	NA	45.2	86.0	99.6	91.3
* Canis*	NA	1.3	0.0	80.4	101	89.1
* Equus*	11.7	1.7	10.5	84.7	93.3	90.1
* Eumetopias*	2.26	1.5	0.0	79.7	105	90.6
* Felis*	3.77	1.9	13.1	76.8	91.4	96.7
* Manis*	2.45	1.7	20.5	77.0	84.8	88.6
* Nycteris*	NA	1.0	0.0	85.9	112	94.9
* Pteropus*	1.62	1.8	29.7	84.9	98.3	90.4
* Rhinolophus*	1.40	1.4	38.3	79.9	104	87.9
* Sorex*	1.58	1.7	11.9	75.3	89.6	89.3
* Sus*	3.18	1.3	16.5	82.8	96.0	87.9
* Tadarida*	1.42	2.0	22.1	74.7	98.4	98.4
* Tursiops*	2.23	1.4	0.0	52.2	84.9	77.5
Euarchontoglires						
* Cavia*	3.82	2.9	25.3	77.2	105	85.5
* Cynocephalus*	1.80	2.0	30.8	92.2	90.0	91.8
* Homo*	5.47	3.0	55.8	98.9	100	89.8
* Lepus*	3.71	1.7	32.4	84.2	94.0	88.6
* Macaca*	3.76	2.5	50.1	83.1	100	89.0
* Mus*	1.28	1.9	25.8	88.8	94.4	95.6
* Sylvilagus*	2.08	1.5	33.9	92.7	97.5	77.9
* Tupaia*	2.61	2.6	13.1	82.3	106	102

a Measurements: Aqueduct Length, length of bony channel for vestibular aqueduct (mm); Stapedial Ratio, height of fenestra vestibuli over width; Labyrinth Index, sagittal labyrinthine index [34]; Semicircular Canal Angles, formed between planes of anterior (A), lateral (L) and posterior (P) canals (°). Values for extinct eutherians are averages [83].

**Table 4:** Linear dimensions of the semicircular canals^a^.

Taxon	Radius			Length			Lumen Diameter		
	Ant	Lat	Post	Ant	Lat	Post	Ant	Lat	Post
Marsupialia									
* Didelphis*	1.46	0.88	1.23	8.24	5.07	7.53	0.26	0.30	0.28
Eutheria									
* Kulbeckia*	1.19	0.92	0.96	5.70	3.94	4.55	0.18	0.20	0.20
* Ukhaatherium*	0.84	0.74	0.69	3.81	3.16	3.39	0.17	0.13	0.15
* Zalambdalestes*	1.46	1.21	1.20	6.92	5.20	5.85	0.19	0.17	0.18
Zhelestid	1.17	0.79	0.86	5.80	3.49	4.62	0.19	0.19	0.19
Afrotheria									
* Chrysochloris*	1.10	0.67	0.71	4.71	2.62	3.60	0.15	0.18	0.16
Elephantimorpha	4.99	2.67	5.51	24.6	12.5	24.3	1.85	1.69	1.77
* Hemicentetes*	1.10	0.68	0.89	4.96	2.44	4.79	0.13	0.15	0.09
* Macroscelides*	1.32	1.05	1.02	5.61	4.21	5.22	0.19	0.20	0.20
* Orycteropus*	3.10	3.27	3.50	15.4	16.4	18.86	0.58	0.53	0.55
* Procavia*	1.99	1.79	2.18	10.2	7.65	10.7	0.21	0.33	0.27
* Trichechus*	4.30	4.46	3.54	17.3	14.2	16.5	0.51	0.52	0.51
Xenarthra									
* Dasypus*	1.64	1.60	1.92	9.69	7.38	11.3	0.22	0.23	0.23
Laurasiatheria									
* Atelerix*	1.24	0.88	1.22	5.88	3.67	5.80	0.16	0.15	0.15
Balaenopteridae	2.54	2.11	1.92	10.7	8.54	9.46	0.32	0.51	0.41
* Bathygenys*	1.91	1.52	1.79	9.72	7.11	10.0	0.44	0.33	0.38
* Canis*	1.73	1.57	1.43	8.58	7.08	7.37	0.31	0.35	0.33
* Equus*	3.62	3.55	3.50	17.4	14.3	18.63	0.51	0.45	0.48
* Eumetopias*	3.00	3.13	2.86	13.0	14.8	14.1	0.38	0.53	0.45
* Felis*	1.92	1.68	1.91	8.78	7.48	9.39	0.26	0.26	0.26
* Manis*	1.46	1.06	1.66	6.59	3.71	7.03	0.55	0.62	0.59
* Nycteris*	0.97	0.87	0.79	4.34	3.40	4.36	0.12	0.14	0.13
* Pteropus*	1.57	1.28	1.35	6.86	5.86	7.03	0.17	0.24	0.20
* Rhinolophus*	0.83	0.69	0.74	3.52	3.21	3.90	0.07	0.09	0.08
* Sorex*	0.65	0.48	0.63	3.20	1.63	3.42	0.12	0.14	0.13
* Sus*	2.50	2.08	2.18	12.1	8.04	10.7	0.42	0.39	0.41
* Tadarida*	0.85	0.73	0.74	3.90	3.26	3.59	0.15	0.17	0.16
* Tursiops*	1.19	1.36	0.84	4.14	4.61	4.35	0.27	0.25	0.26
Euarchontoglires									
* Cavia*	1.88	1.57	1.63	9.01	6.49	8.18	0.21	0.29	0.25
* Cynocephalus*	1.93	1.47	1.70	9.93	6.99	8.38	0.27	0.37	0.32
* Homo*	2.94	2.35	3.10	13.6	10.3	14.73	0.92	0.86	0.89
* Lepus*	2.34	1.66	1.69	11.5	6.86	8.10	0.27	0.26	0.26
* Macaca*	2.70	2.47	2.54	12.8	10.6	13.05	0.33	0.50	0.41
* Mus*	0.78	0.60	0.67	3.86	2.48	3.60	0.15	0.15	0.15
* Sylvilagus*	1.86	1.29	1.44	8.98	5.65	7.38	0.12	0.24	0.18
* Tupaia*	1.73	1.44	1.50	9.24	7.85	8.07	0.18	0.22	0.20

a Measurements expressed in millimeters. Values for extinct eutherians are averages [83].

**Table 5:** Deviations and aspect ratios of the semicircular canals^a^.

Taxon	Linear			Angular			Ratio		
	Ant	Lat	Post	Ant	Lat	Post	Ant	Lat	Post
Marsupialia									
* Didelphis*	0.22	0.38	0.00	8.62	23.7	0.00	0.97	0.79	1.08
Eutheria									
* Kulbeckia*	0.23	0.04	0.09	11.10	2.70	5.09	1.02	0.97	1.02
* Ukhaatherium*	0.06	0.04	0.12	8.22	6.21	9.92	0.94	0.95	0.90
* Zalambdalestes*	0.08	0.13	0.14	5.83	6.32	6.85	1.08	0.88	0.98
Zhelestid	0.23	0.11	0.23	12.9	6.88	15.20	0.95	0.75	0.89
Afrotheria									
* Chrysochloris*	0.13	0.00	0.23	6.81	0.00	18.9	1.32	1.01	0.91
Elephantimorpha	1.60	0.14	1.36	18.50	3.01	14.3	0.72	1.31	1.10
* Hemicentetes*	0.18	0.07	0.10	9.41	5.90	6.48	0.88	0.93	0.72
* Macroscelides*	0.26	0.06	0.24	11.40	3.27	13.5	0.91	0.75	0.82
* Orycteropus*	1.06	0.41	0.70	19.70	7.21	11.5	0.81	1.03	1.28
* Procavia*	0.27	0.18	0.23	7.79	5.78	6.06	0.68	0.72	0.79
* Trichechus*	0.59	0.69	0.00	7.86	8.87	0.00	0.91	0.89	1.19
Xenarthra									
* Dasypus*	0.37	0.50	0.26	13.00	18.1	7.76	0.58	0.96	1.16
Laurasiatheria									
* Atelerix*	0.23	0.29	0.31	10.6	18.90	14.6	0.87	0.99	0.97
Balaenopteridae	0.40	0.20	0.53	9.03	5.44	15.9	0.91	0.39	1.21
* Bathygenys*	0.27	0.21	0.42	8.10	7.92	13.5	0.86	0.99	0.95
* Canis*	0.18	0.14	0.27	5.98	5.10	10.8	0.82	1.01	0.98
* Equus*	0.14	0.29	0.35	2.22	4.68	5.74	0.93	1.15	1.04
* Eumetopias*	0.04	0.89	0.47	0.76	16.4	9.45	0.96	1.24	1.18
* Felis*	0.15	0.13	0.00	4.48	4.43	0.00	0.77	1.04	1.01
* Manis*	0.17	0.00	0.21	6.69	0.00	7.25	0.76	0.82	0.93
* Nycteris*	0.07	0.10	0.31	4.14	6.61	22.7	0.91	0.71	0.95
* Pteropus*	0.28	0.32	0.11	10.2	14.3	4.67	0.94	0.97	0.85
* Rhinolophus*	0.12	0.05	0.18	8.31	4.14	13.9	0.83	0.46	0.98
* Sorex*	0.09	0.00	0.23	7.94	0.00	21.2	1.63	0.88	0.72
* Sus*	0.00	0.08	0.10	0.00	2.20	2.63	0.78	0.83	0.74
* Tadarida*	0.03	0.06	0.00	2.03	4.69	0.00	0.81	0.58	0.91
* Tursiops*	0.00	0.21	0.00	0.00	8.86	0.00	0.95	0.96	1.60
Euarchontoglires									
* Cavia*	0.62	0.43	0.86	19.1	15.80	30.7	0.75	0.49	0.99
* Cynocephalus*	0.45	0.09	0.09	13.4	3.51	3.04	0.82	0.85	1.05
* Homo*	0.99	0.29	0.68	19.5	7.08	12.7	0.86	0.85	1.08
* Lepus*	0.16	0.06	0.32	3.92	2.07	10.9	0.86	0.87	0.81
* Macaca*	1.23	0.33	0.52	26.4	7.68	11.8	0.87	0.89	0.98
* Mus*	0.18	0.02	0.04	13.3	1.90	3.43	0.67	0.92	0.75
* Sylvilagus*	0.16	0.12	0.62	4.95	5.34	25.3	0.97	0.84	0.94
* Tupaia*	0.69	0.21	0.28	23.1	8.41	10.8	0.85	0.71	0.96

a Measurements: Linear deviations expressed in millimeters; angular deviations expressed in degrees; aspect ratio calculated as height of canal arc divided by width. Values for extinct eutherians are averages [83].

*Didelphis* is a common animal in North America, despite it being the only North American marsupial. The body mass of the specimen used (TMM M-2517) is 2.8 kg (see Table 1), which is on the higher end of the mass range of the species (1.6–3.1 kg [89]). The cochlea of *Didelphis* contributes 69% of the total volume of the inner ear, which is close to that calculated for the ancestral therian (66%). The cochlear spiral is high in profile (Table 2; Figure 4E), the structure completes nearly two and a quarter turns (Table 2; Figure 4D), and the secondary bony lamina extends along the radial wall of the cochlear canal (sl in Figures 4C and 5, slice 54) beyond the first turn of the cochlea. The vestibular wall of the cochlea is expanded behind the fenestra cochleae to accommodate the perlimphatic sac (ps Figure 4A, C, and E). The bony canaliculus cochleae for the cochlear aqueduct extends as a straight tube from the swelling for the perilymphatic sac. The plane of the basal turn of the cochlea is rotated ventrally and anteriorly from the plane of the lateral semicircular canal by 19.6° (Table 2; Figure 4C).

The basal end of the cochlea is inflected at the junction between the cochlea and the spherical recess of the vestibule. The fenestra vestibuli, in which the stapes sits, is rounded in shape (stapedial ratio in Table 3). The division between the spherical and elliptical recesses within the bony vestibule is not distinct in *Didelphis* (anterior ampulla labeled aa is within elliptical recess spherical recess is labeled sr in Figure 5, slice 54), although the swelling of the spherical recess is observed in anterior view of the labyrinth (sr in Figure 4A). The elliptical recess is bowed slightly medially (er in Figure 4B). The anterior and posterior ends of the elliptical recess are penetrated by two large openings each, with the anterior (medial) and lateral (lateral) ampullae in the anterior aspect and the common crus and posterior ampulla at the posterior extremity (the opening for the common crus is medial to that of the posterior ampulla; cr and pa in Figure 4B). The lateral semicircular canal does not possess a separate opening into the vestibule. Rather, the posterior limb of the lateral canal joins with the lateral limb of the posterior canal to form a secondary common crus (scr in Figure 4B, C, and F; Figure 5, slice 66). Presence of the secondary crus in *Didelphis* is a plesiomorphic condition inherited from the ancestor of Theria. The lateral canal does not extend posterior to the plane of the posterior semicircular canal as is observed in species such as *Mus musculus* and *Procavia capensis*, and the lateral extent of the lateral and posterior semicircular canal arcs are equivalent when the labyrinth is in dorsal or anterior view (lc and pc in Figure 4A and B).

The bony channel for the vestibular aqueduct exits the vestibule ventral and anterior to the common crus. The channel extends dorsally and posteriorly, crossing the common crus in medial view, as a slender and straight tube before widening as it curves medially and becomes flattened. The channel for the vestibular aqueduct is more robust and over one and a half times longer than the bony canaliculus cochleae for the cochlear aqueduct (Tables 2 and 3).

The planes of all three semicircular canals form obtuse angles with one another, particularly between the anterior and lateral canals (Table 3). The anterior canal is the largest of the three, in terms of the length of the slender portion of the canal and arc radius (Table 4). However, the cross-sectional diameter of the lumen of the lateral semicircular canal is greater than either the anterior or posterior canals. The arcs of the anterior and posterior canals approach circularity (aspect ratios in Table 5), although the arc of the lateral semicircular canal is lower, being relatively wider than either the anterior or posterior canal arcs. The ratio of the slender portion of the semicircular canal length over arc radius of curvature calculated for the posterior canal is greatest among the three canals (6.11; ratios for anterior and lateral canals are 5.63 and 5.47 respectively).

In gross morphology, the posterior canal is straight along its course and fits onto a single plane (pc in Figure 4B–C). However, both the anterior and lateral canals deviate from their average plane (Table 5). The lateral canal is dorsally deflected along its midsection, but the anterior and posterior limbs are straight as they enter the lateral ampulla and secondary common crus respectively (Figure 4A). The course of the anterior semicircular canal diverges medially at its midpoint (ac in Figure 4A and B), and the lateral canal is sigmoid when viewed with its plane parallel to the horizon (lc in Figure 4C). The total angular deviation of the lateral semicircular canal from its plane is greater than that calculated for the other canals (Table 5), and only he lateral canal exhibits substantial linear deviation from its plane (ratio of the total linear deviation over canal lumen diameter equals 1.27; a ratio of 1.0 or above is considered substantial).

Compared to the reconstructions for the therian ancestor, the bony labyrinth of *Didelphis* retains several plesiomorphic therian characters, namely the presence of the secondary common crus and a relatively larger anterior semicircular canal compared to the lateral and posterior canals [11], [83]. A third character that likely is ancestral for therians, or at least eutherians, is a cochlea that is coiled to around 360° [11], [83]. However, the cochlea is derived for *Didelphis* in this regard, as it completes over two turns (Table 2). Lastly, the position of the lateral semicircular canal of *Didelphis* is similar to that of Cretaceous eutherians [83] in that the lateral canal does not divide the space enclosed by the posterior canal in anterior view, a condition that is observed in many placentals, including the golden mole *Chrysochloris* (see below).

#### Eutheria

**Figure 6:**
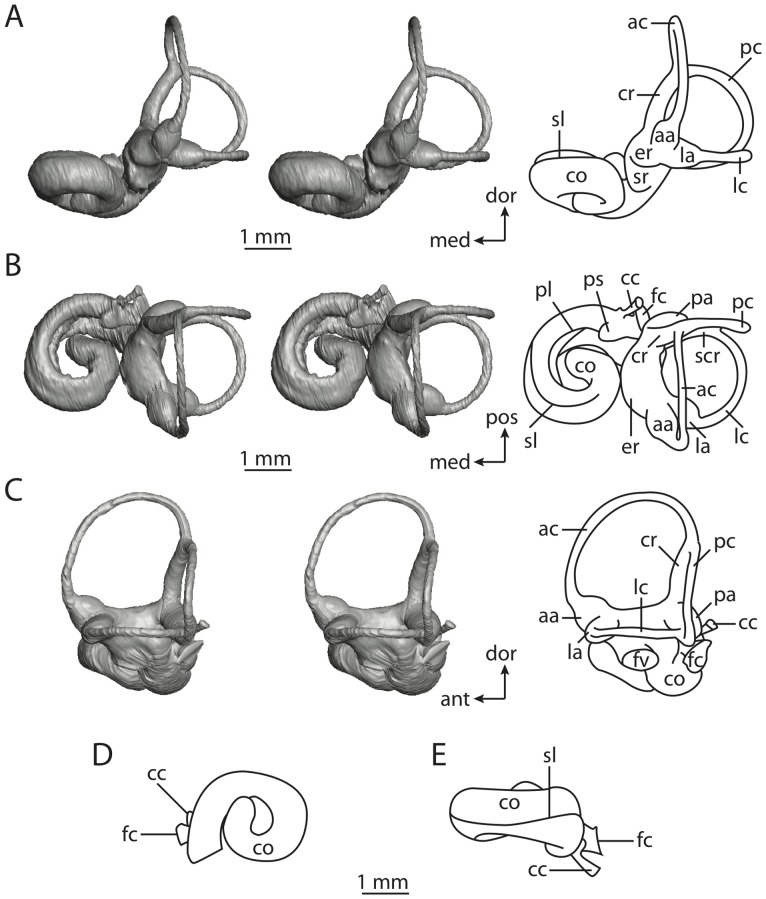
Bony labyrinth of*Kulbeckia kulbecke* (images reversed). **A**, stereopair and labeled line drawing of digital endocast in anterior view (modified from Ekdale and Rowe [83]); **B**, stereopair and labeled line drawing of digital endocast in dorsal view; **C**, stereopair and labeled line drawing of digital endocast in lateral view; **D**, line drawing of cochlea viewed down axis of rotation to display degree of coiling; **E**, line drawing of cochlea in profile. Abbreviations listed at the end of the Materials and Methods section.

**Figure 7:**
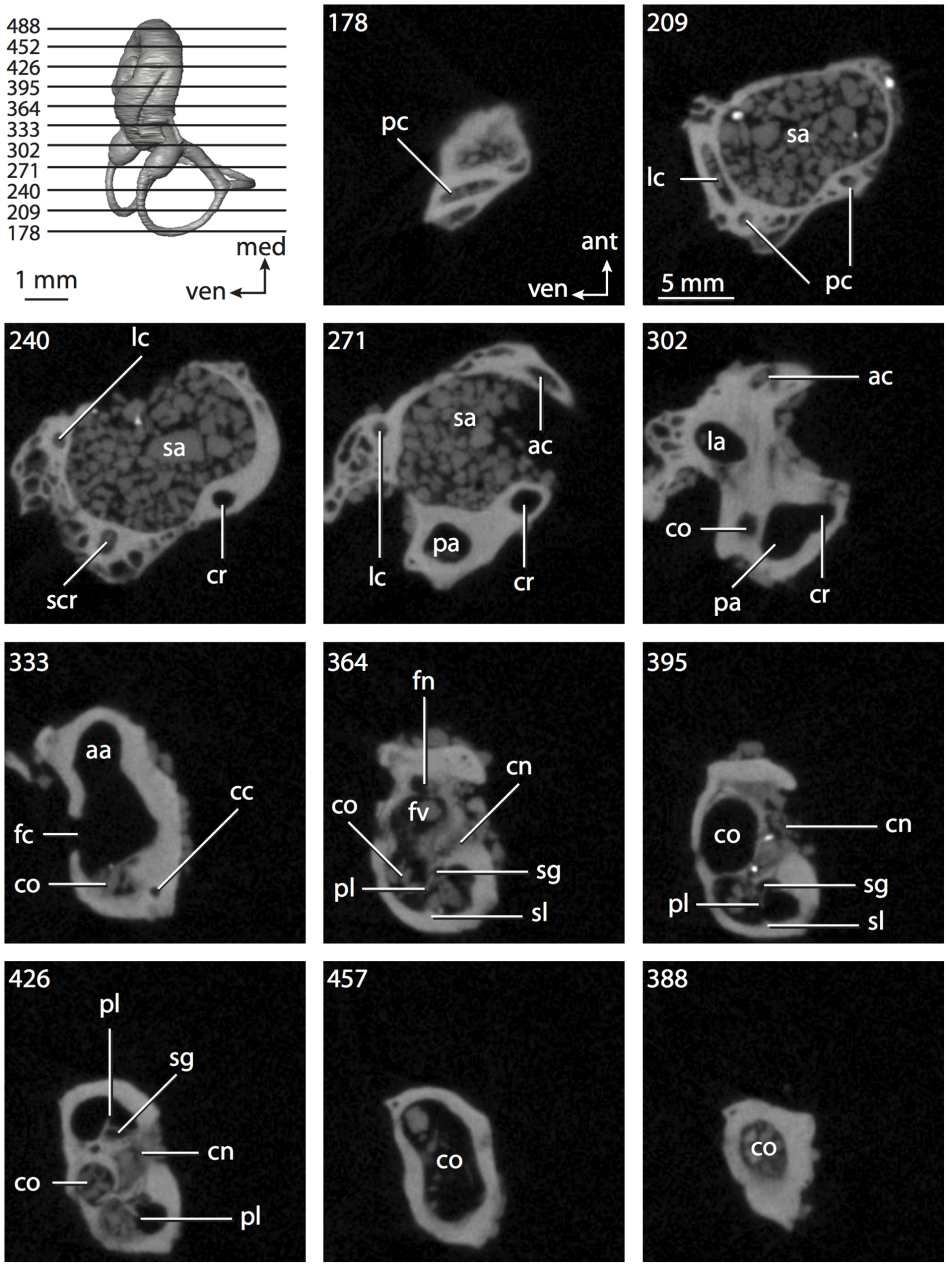
CT slices through ear region of*Kulbeckia kulbecke.* Numbers refer to specific CT slices. Additional information provided by Ekdale and Rowe [83]. Abbreviations listed at the end of the Materials and Methods section.

Eutheria is defined as the most recent common ancestor of crown Placentalia and all taxa more closely related to Placentalia than to Marsupialia (the marsupial mammals). A brief overview of the labyrinth of *Kulbeckia kulbecke* is provided here as a representative of non-placental Mesozoic eutherians (which are thought to exhibit the ancestral condition for Eutheria, if not Theria [83]), although data from three additional non-placental eutherian taxa were used to reconstruct hypothetical ancestral states (Tables S1–2). The non-placental eutherian taxa that were examined are from the Cretaceous of Asia, and they include a representative of a monophyletic group of zhelestids from the Bissekty Formation of Uzbekistan [83], *Kulbeckia kulbecke* (also from Uzbekistan), *Ukhaatherium nessovi*, and *Zalambdalestes lechei* (the latter two taxa from Mongolia). The relationships depicted for these taxa in Figure 2 follow the analyses by Wible and others [6], and more thorough descriptions of the bony labyrinths of these taxa are provided elsewhere [83]. The gross morphology of the non-placental taxa does not vary significantly among the taxa examined, and the values of measurements for *Kulbeckia* are averages across a sample of four petrosal specimens. The bony labyrinth of *Kulbeckia* is illustrated in Figures 6 and 7.

The total length of the bony labyrinth of *Kulbeckia* is slightly less than that measured for *Didelphis* (Table 1; labyrinth length could only be measured for two specimens, URBAC 00–16 and URBAC 04–36), as are the volumetric contribution of the cochlea to the total volume of the inner ear as well (48% versus 69%), the aspect ratio of the cochlear spiral (Figure 6E), degree of coiling (Figure 6D), and spiral length of the cochlear canal (Table 2). The canaliculus cochleae extends from a swelling of the cochlea for the perilymphatic sac near the fenestra cochleae (cc and ps in Figure 6B).

The plane of the basal turn of the cochlea is rotated from the plane of the lateral semicircular canal by a lesser degree in *Kulbeckia* than in *Didelphis* (Table 2), and the basal end of the cochlea is inflected before it joins the spherical recess of the vestibule near the fenestra vestibuli (average stapedial ratio of 1.9 [97]). The spherical and elliptical recesses are distinguished by a constriction of the vestibule lateral to the fenestra vestibuli (sr and er in Figure 6A). As was observed in *Didelphis*, the posterior and lateral semicircular canals fuse to form a secondary common crus (scr in Figure 6B; Figure 7, slice 240), which in turn empties into the posterior ampulla. The lateral semicircular canal does not extend posterior to the plane of the posterior canal (lc and pc in Figure 6A and C), and the lateral extent of the canal arcs are equivalent when the labyrinth is in dorsal or anterior view (Figure 6B). The common crus between the anterior and posterior semicircular canals is situated medial to the posterior ampulla (cr and pa in Figure 6B). The bony channel for the vestibular aqueduct was observed anteromedial to the common crus in two *Kulbeckia* specimens (URBAC 00–16 and 04–36).

The planes of the lateral and posterior semicircular canals almost form a right angle, but the angles that each of these canals form with the anterior canal are acute (Table 3). The anterior semicircular canal is the largest in terms of radius and length of the slender portion of the canal (Table 4). However, the lateral canal is the largest in terms of cross-sectional diameter. The aspect ratios of the anterior and posterior semicircular canals are identical (Table 5) with arcs that are higher than they are wide. In contrast, the arc of the lateral semicircular canal is wider than it is high. The ratio of the slender portion of the anterior semicircular canal length over arc radius of curvature (4.80) is the largest ratio calculated among the three canals, although the ratio for the posterior canal is close to that of the anterior (4.75; ratio for lateral canal is 4.29).

The anterior semicircular canal deviates the most from its plane, and the lateral canal is the most planar (Table 5). The anterior and posterior limbs of the lateral canal are straight as they enter the lateral ampulla and secondary common crus respectively (lc in Figure 6C). Only the anterior canal deviates from its plane by a substantial amount, although the posterior canal curves anteriorly when viewed perpendicular to its plane (pc in Figure 6C).

The inner ear morphology of *Kulbeckia* and the other Mesozoic taxa, as well as *Didelphis*, were used to reconstruct the ancestral states of Eutheria. The bony labyrinth of the ancestor of Eutheria retained the ancestral therian conditions in all respects. The lateral semicircular canal formed a secondary common crus with the posterior canal, the plane of the lateral canal was low compared to the ampullar entrance of the posterior semicircular canal, the arc of the anterior semicircular canal was the largest among the three semicircular canals, and the aspect ratio of the cochlea was low (below 0.55). All ancestors at the nodes leading to crown Placentalia retained the ancestral eutherian states for all discrete characters.

The contribution of the ancestral eutherian cochlea to the total inner ear volume was 64%, which was only slightly less than that reconstructed for Theria (66%), and the percentage decreased through time (59% for the most recent common ancestor of *Ukhaatherium* and Placentalia; 56% for the most recent common ancestor of Zalambdalestidae, which includes *Kulbeckia* and *Zalambdalestes*, and Placentalia). The contribution of the cochlea of the ancestral zalambdalestid was 51%.

The ancestral eutherian cochlea was reconstructed to complete 580°, which was less than that reconstructed as the ancestral therian condition (685°). As discussed above in the reconstruction of the ancestor of Theria, these values are overestimates, and the ancestral eutherian most likely possessed a cochlea that completed a single turn only (as has been suggested elsewhere [11], [83]). Likewise, the ancestral states at the nodes leading up to Placentalia likely were 360°, despite reconstruction of the most recent common ancestor of *Ukhaatherium* and Placentalia as 510°, the ancestor of Zalambdalestidae and Placentalia as 570°, and the most recent common ancestor of Zalambdalestidae as 461°. The zalambdalestid reconstruction appears particularly egregious given that neither zalambdalestid taxa nor any stratigraphically older fossils possessed cochleae coiled over 360°.

#### Placentalia

Placentalia includes the most recent common ancestor of extant placental mammals (e.g., *Hemicentetes semispinosum*, *Dasypus novemcinctus*, and *Homo sapiens*) plus all of its descendants. Placentalia is divided into the three major lineages Afrotheria, Xenarthra, and Boreoeutheria, which in turn is divided into Laurasiatheria and Euarchontoglires [66], [98].

Entry of the lateral semicircular canal directly into the vestibule in absence of a secondary common crus is the single unambiguous otic synapomorphy for Placentalia, which is a condition not found outside of the crown (at least within Eutheria) [83]. The vast majority of placental taxa lack a secondary common crus (only exceptions among sampled taxa are *Orycteropus afer* and *Canis familiaris*). The cochlea of the ancestor of placental mammals completes 738° (over two turns), and the volumetric contribution of the cochlea to the entire labyrinth (58%) is less than that of the ancestral eutherian (64%).

The arc of the anterior semicircular canal is the largest among the three canal arcs, which is retained from the ancestor of Theria. The reconstructed states of both the position of the plane of the lateral semicircular canal compared to the ampullar entrance of the posterior canal and the aspect ratio of the cochlea in profile are equivocal owing to variation in the position of the lateral canal within Afrotheria and variation in the shape of the cochlear spiral in both Afrotheria and Boreoeutheria.

#### Afrotheria

Afrotheria is a clade of placentals endemic to Africa that includes the groups Afrosoricida (tenrecs and golden moles), Macroscelidea (elephant shrews), Tubulidentata (aardvark), Hyracoidea (hyraxes), Sirenia (dugongs and manatees), and Proboscidea (elephants). Monophyly of Afrotheria is controversial, primarily because it was not recognized in classical morphological studies of placentals, whether based on strict cladistic methodologies or not [33], [99]–[107]. Monophyletic Afrotheria (including the afrosoricids and macroscelids) was first proposed by Springer and others [108], although the first use of the name “Afrotheria” was by Stanhope and others [109]. The earliest support for Afrotheria as a whole was restricted to molecular evidence [108]–[114]. Although more recent morphological evidence has been proposed to support the clade [114]–[117], strict morphological analyses fail to recover afrotherian monophyly [6], [118].

The members of Afrotheria studied here are *Macroscelides proboscideus* (Macroscelidea), *Orycteropus afer* (Tubulidentata), a fossil elephantimorph proboscidean (either *Mammut* or *Mammuthus* [84]), *Trichechus manatus* (Sirenia), *Procavia capensis* (Hyracoidea), and the two afrosoricids *Chrysochloris* sp. (Chrysochloridae) and *Hemicentetes semispinosum* (Tenrecidae). There is a broad range in body mass among these taxa (Table 1), from 44 grams in *Chrysochloris* [89] to upwards of 8,000 kg in extinct elephantimorphs [119]. Likewise, the inner ear cavities vary in size. The overall volume of the bony labyrinth within Afrotheria ranges from 4.11 mm^3^ in *Chrysochloris* to 26.0 mm^3^ in the fossil elephantimorph. Dimensions of the bony labyrinths of afrotherians are provided in Table 1. Dimensions of the cochlea are provided in Table 2, and dimensions and orientations of the semicircular canals are reported in Tables 3–5.

Afrotheria often is placed as the sister taxon to all other placentals [98], [112], although the results of Bininda-Emonds and colleagues that are used here include Afrotheria in a basal polytomy with Xenarthra and a clade comprising the remaining placentals [66]. Three major lineages are included within Afrotheria, which are Tubulidentata (aardvarks), Paenungulata (hyraxes, manatees, and elephants), and a clade including Macroscelidea (elephant shrews) and Afrosoricida (golden moles and tenrecs). The three major lineages are placed within a polytomy at the base of Afrotheria (Figure 2).

The bony labyrinth of the ancestor of Afrotheria retained the ancestral morphology of Placentalia in that the lateral semicircular canal entered into the vestibule directly and the arc of the anterior semicircular canal was the greatest among the three canal arcs. The reconstructed ancestral states of the position of the lateral semicircular canal compared with the posterior canal, as well as the aspect ratio of the cochlea, are equivocal based on the afrotherian morphology described here. The states reconstructed and inferred for all of the nodes within Afrotheria are identical to that of the afrotherian ancestor, except the state for the largest semicircular canal arc which is equivocal for the clade consisting of *Procavia* and *Trichechus* (the posterior arc is largest for *Procavia* and the lateral is largest for *Trichechus*; see below).

The volumetric contribution of the cochlea to the total labyrinthine volume of the ancestral afrotherian was 56%, which was close to that reconstructed for the ancestor of Placentalia (58%). The ancestral cochlear contribution of the paenungulate clade consisting of *Procavia* and *Trichechus* was the same as that of the afrotherian ancestor (56%), although the contribution of the cochlea of the ancestor of Paenungulata was almost ten percent less (48%), likely on account of the low contribution of the cochlea of the proboscidean (see below). Contributions of 63% and 64% were reconstructed for the ancestors of Afrosoricida and the more inclusive clade that also includes Macroscelidea, respectively. The ancestral afrotherian cochlea coiled 751°, which was greater than the ancestral placental condition, but less than the values reconstructed for the nodes within Afrotheria (768° for the clade consisting of Afrosoricida and Macroscelides; 833° for Afrosoricida; 790° for Paenungulata; 853° for the clade consisting of *Procavia* and *Trichechus*).

#### Afrosoricida

The group Afrosoricida contains Tenrecidae (tenrecs) and Chrysochloridae (golden moles). Although traditional classifications (for example, that of Simpson [100]) group tenrecs and chrysochlorids with other insectivorous mammals, such as the lipotyphlans *Erinaceus* (hedgehog) and *Sorex* (shrew), the results of more recent molecular studies [108]–[109] ally Tenrecidae and Chrysochloridae with other placentals within the clade of African endemic mammals Afrotheria. *Chrysochloris* sp. (Chrysochloridae) and *Hemicentetes semispinosum* (Tenrecidae) represent the afrosoricids.

**Figure 8:**
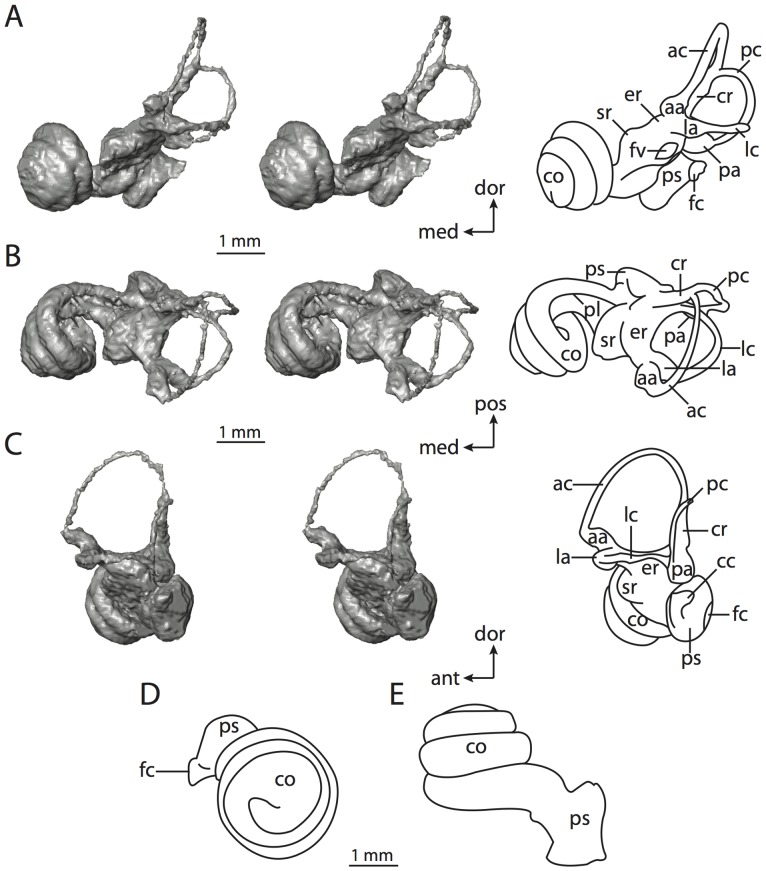
Bony labyrinth of*Chrysochloris* sp. **A**, stereopair and labeled line drawing of digital endocast in anterior view; **B**, stereopair and labeled line drawing of digital endocast in dorsal view; **C**, stereopair and labeled line drawing of digital endocast in lateral view; **D**, line drawing of cochlea viewed down axis of rotation to display degree of coiling; **E**, line drawing of cochlea in profile. Abbreviations listed at the end of the Materials and Methods section.

**Figure 9:**
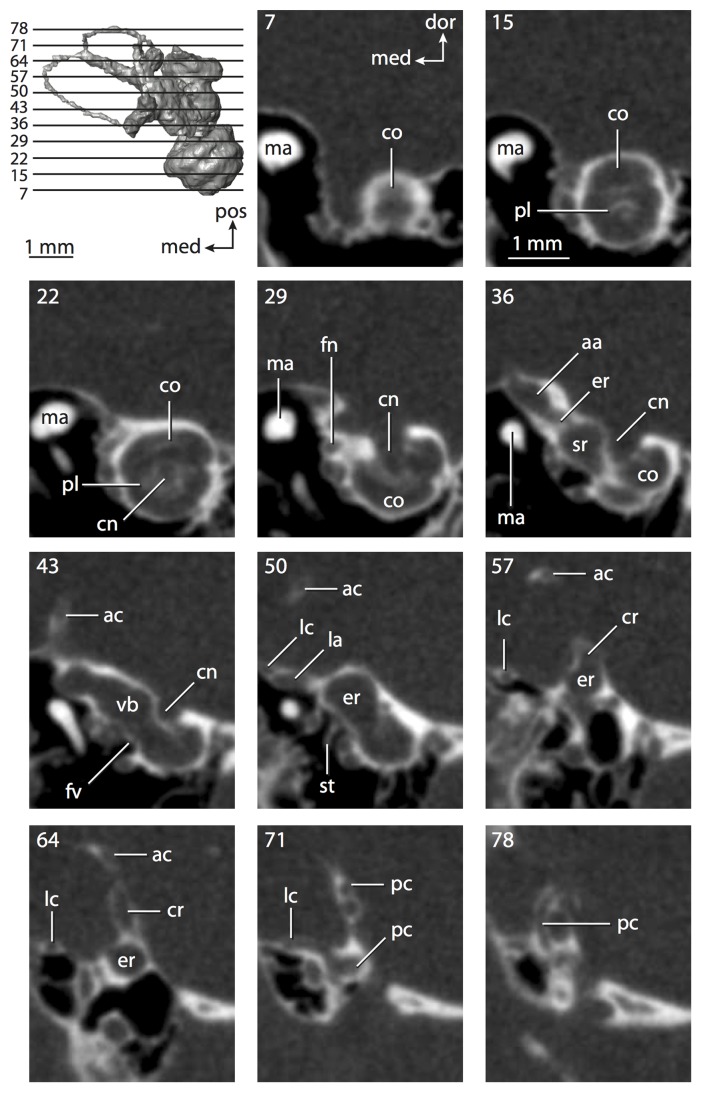
CT slices through ear region of*Chrysochloris* sp. Numbers refer to specific CT slices. Abbreviations listed at the end of the Materials and Methods section.

**Figure 10:**
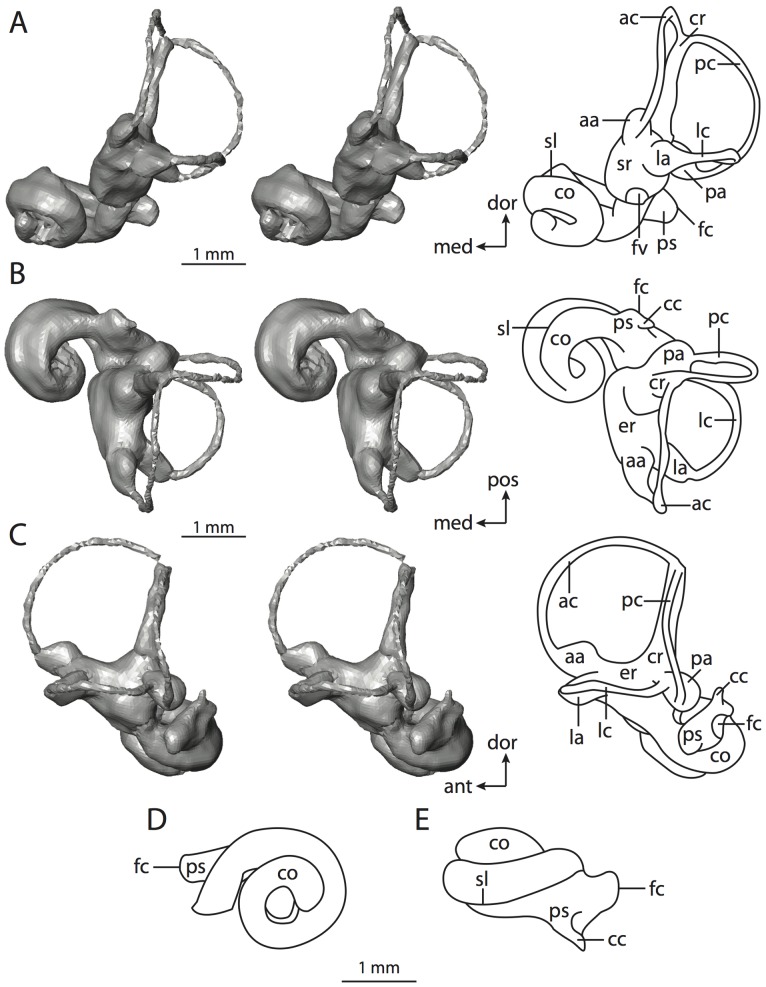
Bony labyrinth of*Hemicentetes semispinosum*. **A**, stereopair and labeled line drawing of digital endocast in anterior view; **B**, stereopair and labeled line drawing of digital endocast in dorsal view; **C**, stereopair and labeled line drawing of digital endocast in lateral view; **D**, line drawing of cochlea viewed down axis of rotation to display degree of coiling; **E**, line drawing of cochlea in profile. Abbreviations listed at the end of the Materials and Methods section.

**Figure 11:**
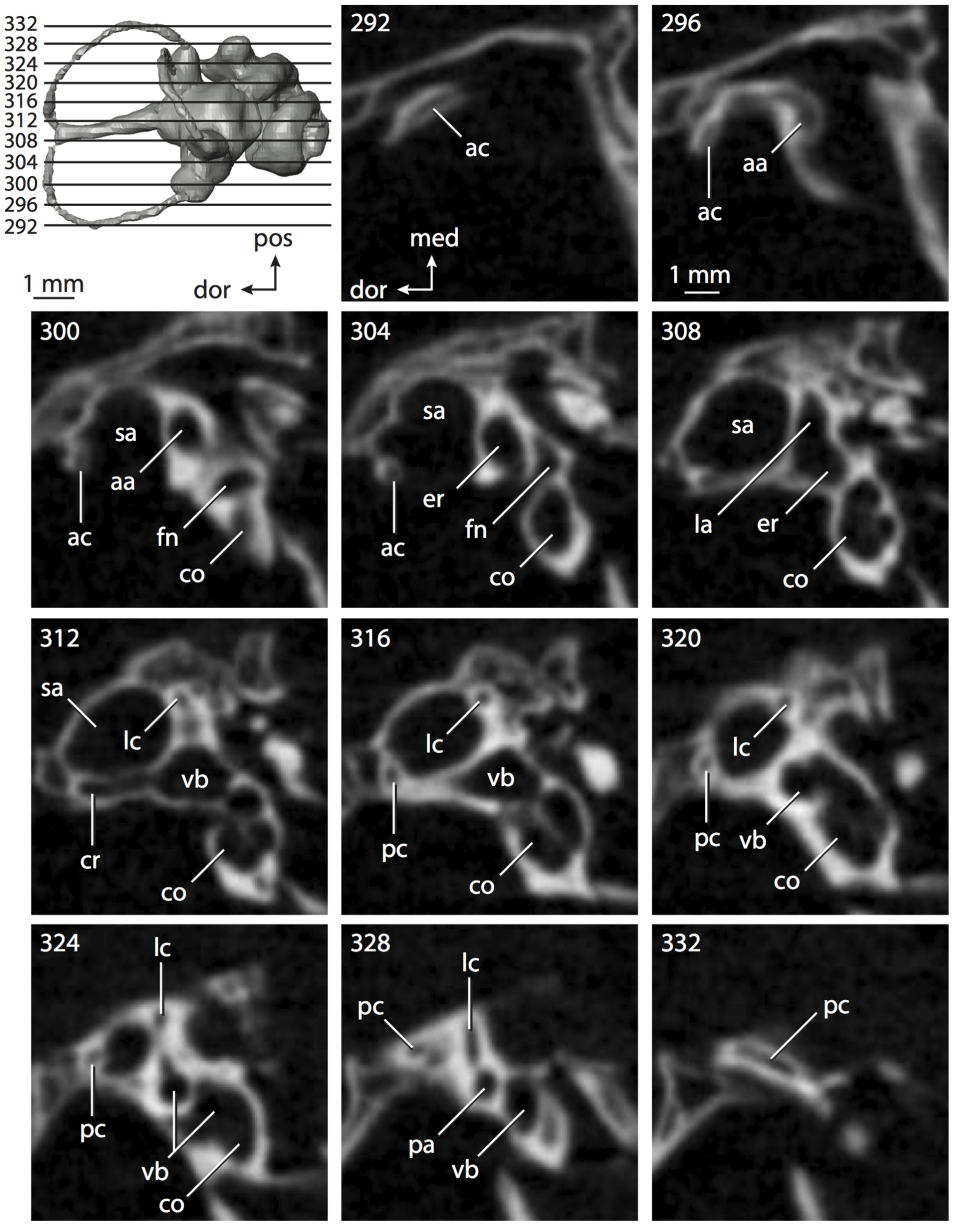
CT slices through ear region of*Hemicentetes semispinosum*. Numbers refer to specific CT slices. Abbreviations listed at the end of the Materials and Methods section.

The bony labyrinths of *Chrysochloris* (Figures 8–9) and *Hemicentetes* (Figures 10–11) differ in several ways, one of which is absolute size, where the former is smaller in bony labyrinth length than the latter, but the volume of the labyrinth is smaller in *Hemicentetes* than in *Chrysochloris* (Table 1; size difference also observed in body mass [89]). The cochlear canal is not only longer and more voluminous in *Chrysochloris* than in *Hemicentetes*, but the spiral also completes a greater degree of coiling (Table 2). The proportion of the total labyrinth volume is greater in *Chrysochloris* (71%) than in *Hemicentetes* (50%) as well.

The cochlea is more planispiral in *Hemicentetes* (co in Figure 10E) than it is in *Chrysochloris* co in Figure 8E). That is, the cochlea in *Hemicentetes* forms a spire with a lower aspect ratio than in *Chrysochloris* (Table 2). The second turn of the cochlea in *Chrysochloris* is nearly equal in diameter to the basal turn, and obscures most of the basal turn when the cochlea is viewed vestibularlly (from down the axis of rotation; Figure 8E), although the apical turn nearly fits within the arc of the basal turn of the cochlea of *Hemicentetes* (Figure 10D). The plane of the basal turn of the cochlea of *Hemicentetes* also is rotated less from the plane of the lateral semicircular canal than it is in *Chrysochloris* (Table 2).

The bony canaliculus cochleae for the cochlear aqueduct is shorter in *Hemicentetes* (Table 2), and the cochlea is expanded for the perilymphatic sac behind the fenestra cochleae in both afrosoricid species (cc and ps in Figures 8B and 10B). The swelling hooks posteriorly before the canaliculus cochleae exits the cochlea, and the canaliculi of these taxa are not as delicate as that observed in *Didelphis*. The fenestra vestibuli is more oval in *Chrysochloris*, with a higher stapedial ratio (Table 3).

The spherical and elliptical recesses are distinguishable within the vestibule of *Chrysochloris* (sr and er in Figures 8A through C and 9, slice 36), where the former projects anteriorly towards the cochlea. As a whole, the spherical recess is ovoid in shape. The elliptical recess is smaller than the spherical recess in *Chrysochloris*, and forms a gently curved tube with openings for the semicircular canal system in dorsal view (er in Figure 8B). Each end of the tube is extended into a chamber dorsally, and each extension is expressed as a short pedestal in the endocast. The anterior and posterior ampullae open into the anterior chamber of the elliptical recess. The posterior chamber is penetrated by three major apertures, which lead to the posterior ampulla, the common crus, and the posterior limb of the lateral semicircular canal. The latter of these apertures opens into the vestibule near the anterodorsal edge of the opening for the posterior ampulla. The lateral semicircular canal does not extend posterior to the plane of the posterior canal, nor does the lateral canal extend as far laterally as the posterior. The morphology is the same for *Hemicentetes* in this regard.

The spherical and elliptical recesses are less distinguishable in *Hemicentetes* than they are in *Chrysochloris*, and the vestibule forms a continuous cavity, albeit irregular in shape (er in Figure 10A through C). The vestibule of *Hemicentetes* is penetrated by four major openings only, as opposed to five in *Chrysochloris*. The openings into the vestibule of *Hemicentetes*, in addition to the junction of the vestibule and cochlea, lead to the ampullae of the three semicircular canals, as well as the common crus. As in *Didelphis*, the posterior limb of the lateral semicircular canal does not have its own aperture into the vestibule in *Hemicentetes*. However, the lateral canal of *Hemicentetes* does not join with the posterior semicircular canal to form a secondary common crus, which is observed in the opossum; rather the lateral canal empties into the posterior ampulla (lc and pa in Figure 10C). A groove on the anterior wall of the posterior ampulla that presumably accommodated the membranous lateral semicircular duct in life extends from the lateral semicircular canal to the vestibule. Such a condition suggests the lateral duct is separate from the membranous posterior ampulla in this species, although such cannot be determined with certainty through examination of the bony labyrinth alone.

No trace of the bony channel for the vestibular aqueduct was observed in the CT slices of *Hemicentetes* (Figure 11). The aqueduct likely is present (there is no record of any mammal lacking this structure) but the bony channel likely is small and too narrow to be captured on the CT images. The channel for the vestibular aqueduct is observed in CT data of *Chrysochloris*, in which the channel exits the elliptical recess medial to the common crus. An indistinct groove for the endolymphatic duct along the medial wall of the elliptical recess (expressed as a ridge on the endocast) extends from the channel for the vestibular aqueduct to the junction between the elliptical and spherical recesses.

The planes of the semicircular canals do not form right angles with one another in either afrosoricid species examined (Table 3). In *Chrysochloris*, the largest angle was measured between the posterior and lateral canals, and the smallest was measured between the anterior and lateral canals. The widest angle measured in *Hemicentetes* is between the anterior and posterior canals, which not only is the largest angle between two semicircular canals in either taxon (Table 3), but it is also the closest angle to 90° in the labyrinths of either *Chrysochloris* or *Hemicentetes*. The smallest angle in *Hemicentetes* is between the posterior and lateral canals.

The anterior canal is the largest of all semicircular canals in terms of length of the slender portion of the canal and arc radius for both afrosoricid taxa included in the present study (Table 4). Likewise, the lateral semicircular canal was the smallest in both species in at least the length of the slender portion of the canal and arc radius. Not only is the anterior semicircular canal the largest among all of the canals, it has the highest aspect ratio in *Chrysochloris* (Table 5), indicating that the height of the arc is larger in proportion to the width than it is in other canals. The height of the lateral semicircular canal arc is nearly equal to the width of that arc in *Chrysochloris* and nearly so in *Hemicentetes* (ratio around 1). The aspect ratio of the posterior canal arc is the lowest among all of the canals between the two species. The ratios between the length of the slender portion of a semicircular canal and the radius of its arc for *Chrysochloris* are 4.30 for the anterior canal, 3.89 for the lateral canal, and 5.07 for the posterior canal. A similar pattern is observed in *Hemicentetes* where the posterior semicircular canal has the highest canal length to arc radius ratio (5.41), and the lateral canal has the lowest (3.59; ratio for anterior canal equals 4.52).

The angular deviation of the anterior and lateral semicircular canals from their planes in *Chrysochloris* are less than that observed for the same canals in *Hemicentetes*, although the lateral semicircular canal of *Chrysochloris* is the only planar canal between the taxa (Table 5). The least planar canal in *Chrysochloris* is the posterior, and the posterior is the only canal to deviate substantially from its plane in *Chrysochloris* (ratio of the total linear deviation over cross-sectional diameter of the posterior canal is 1.31; ratio for the anterior canal is 0.87). The arc of the posterior canal of *Chrysochloris* is curved (pc in Figure 8C), and the same canal is sigmoidal in *Hemicentetes* (pc in Figure 10C). Both the anterior and posterior canals of *Hemicentetes* deviate substantially from their planes (ratios are 1.38 and 1.11 respectively), although the ratio is only 0.47 for the lateral semicircular canal.

Lastly, the plane of the lateral semicircular canal is high with respect to the posterior canal in both *Chrysochloris* and *Hemicentetes* to an extent that it divides the space enclosed by the arc of the posterior semicircular canal into dorsal and ventral sections when the labyrinth is oriented in anterior view (lc in Figures 8A and 10A; sagittal labyrinthine index [34] in Table 3). Within Afrotheria, a similar condition is observed in *Macroscelides* and *Procavia* as described below, although the inices of the two afrosoricids are lower than the other afrotherians exhibiting this feature of the bony labyrinth (see below). In fact, the labyrinthine index of *Hemicentetes* is smaller than that calculated for any other mammal in this study (in which the lateral canal divides the space enclosed by the posterior canal arc when the labyrinth is in anterior view).

The bony labyrinths of both afrosoricid taxa retain the ancestral placental condition in that the lateral semicircular canal does not form a secondary common crus (although the canal opens into the posterior ampulla rather than the vestibule in *Hemicentetes*), and the anterior semicircular canal has the greatest radius among the three canals. Although the cochlea of *Chrysochloris* exhibits a great degree of coiling (over three complete turns; Table 2), the coiling in *Hemicentetes* is only slightly greater than the average calculated for zhelestids from the Bissekty Formation (1.5 versus 1.4 turns; [83]), and nearly 200° (over one half turn) less than the ancestral placental condition.

Both *Hemicentetes* and *Chrysochloris* are derived with respect to the ancestral eutherian condition in the placement of the lateral semicircular canal that visually divides the space enclosed by the posterior semicircular canal when the labyrinth is in anterior view. Such a condition is not observed in *Didelphis* or any Mesozoic eutherian, including *Kulbeckia* as described above (also see descriptions of Ekdale and Rowe [83]).

#### Macroscelidea

**Figure 12:**
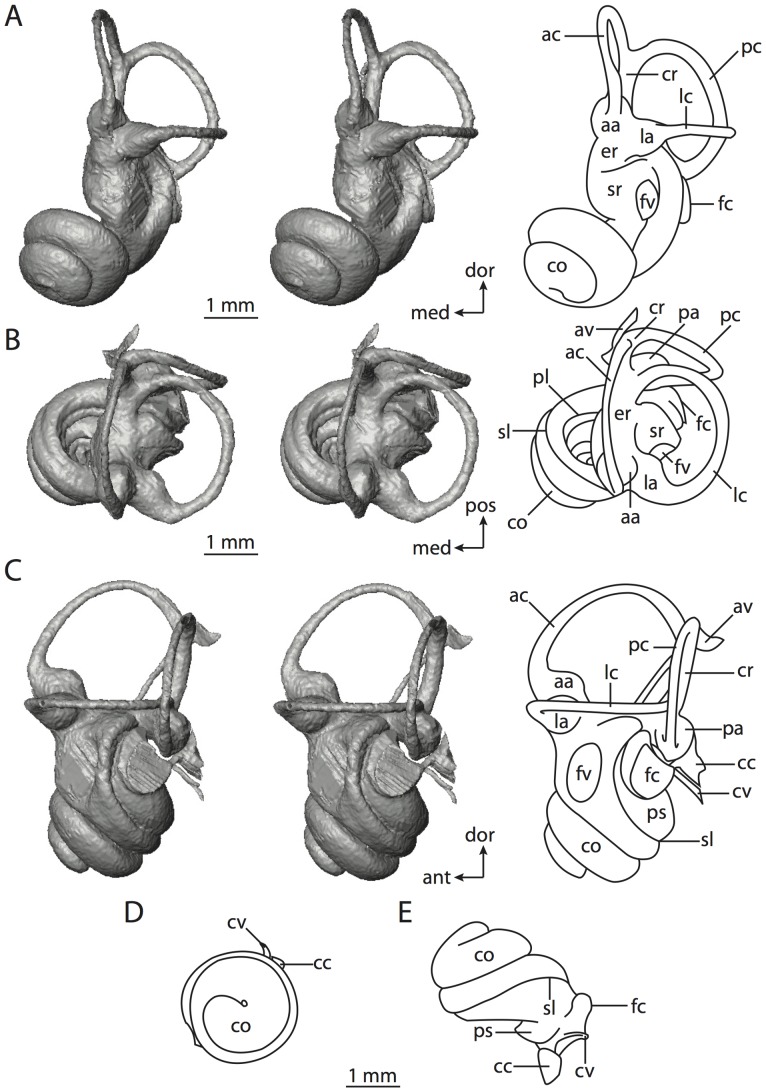
Bony labyrinth of*Macroscelides proboscideus*. **A**, stereopair and labeled line drawing of digital endocast in anterior view; **B**, stereopair and labeled line drawing of digital endocast in dorsal view; **C**, stereopair and labeled line drawing of digital endocast in lateral view; **D**, line drawing of cochlea viewed down axis of rotation to display degree of coiling; **E**, line drawing of cochlea in profile. Abbreviations listed at the end of the Materials and Methods section.

**Figure 13:**
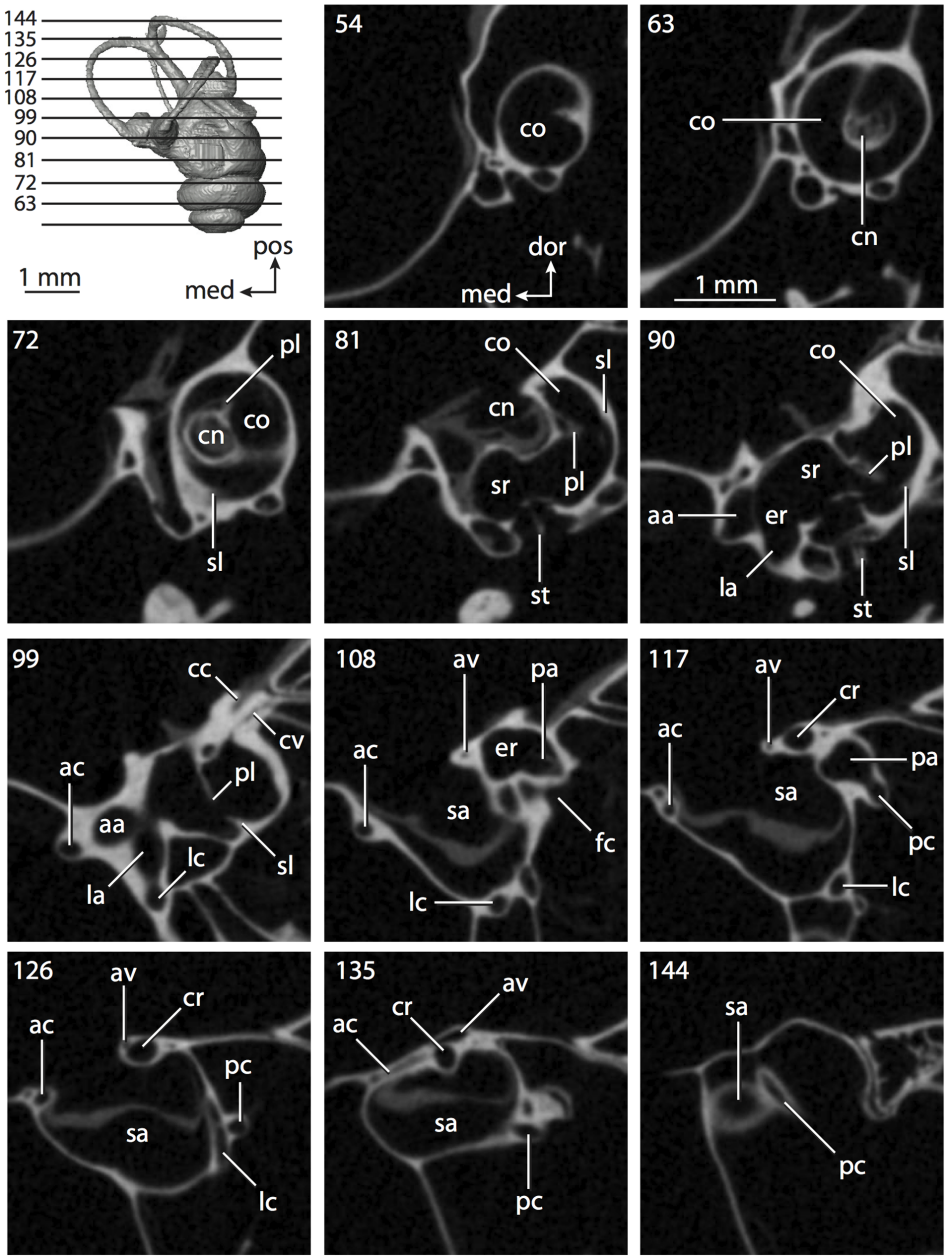
CT slices through ear region of*Macroscelides proboscideus*. Abbreviations listed at the end of the Materials and Methods section.

Macroscelidea contains the elephant shrews or sengis. The phylogenetic affinities of Macroscelidea are contentious, although the analyses of Bininda-Emonds and others [66], as well as other molecular studies [65], [112], include macroscelideans within Afrotheria. Within Afrotheria, Macroscelidea holds a sistergroup relationship with Afrosoricida [66]. Only one species of Macroscelidea (*Macroscelides proboscideus*) was examined in the present study (Figures 12–13). The average body mass of *Macroscelides* is less than body masses reported for either afrosoricid taxon examined ([89]), although the skull is longer (Table 1). However, the dimensions of the bony labyrinth of *Macroscelides* tend to be intermediate between the afrosoricids (Tables 1–5).

The cochlea of *Macroscelides* contributes 71% of the total volume of the inner ear, which is almost identical to that of *Chrysochloris*. Furthermore, the cochlea of *Macroscelides* not only has a higher aspect ratio than *Hemicentetes*, but the ratio in *Macroscelides* is higher than that calculated for any other afrotherian examined in this study (Figure 12E; Table 2). The second turn of the cochlea in *Macroscelides* sits upon the basal whorl (Figure 12D), as is observed in *Chrysochloris*, but not *Hemicentetes*. The cochlear spiral of *Macroscelides* completes two whorls.

The secondary bony lamina for the basilar membrane is well developed on the radial wall of the basal turn of the cochlea (expressed as the distinct groove on the endocast; sl in Figures 12 and 13, slices 72 through 99), and is prolonged beyond three quarter turns of the basal whorl, but ends before the basal whorl is complete. The secondary lamina curves sharply at the basal end of the cochlear canal, between the fenestrae cochleae and vestibuli (sl in Figure 12C), the latter of which has an aspect ratio of 1.9 (similar to that observed in *Hemicentetes*). The plane of the basal turn of the cochlea deviates from that of the lateral semicircular canal by a greater degree in *Macroscelides* than in *Hemicentetes*, but not as much as in *Chrysochloris* (Table 2).

A triangular outpocketing for the perilymphatic sac that leads to two bony canals is situated medial to the fenestra cochleae (ps, cc, and cv in Figure 12E). One of these canals is situated ventral to the other (cv in Figure 12C). The dorsal canal extending from the triangular outpocketing is the bony canaliculus cochleae for the cochlear aqueduct, and the ventral channel transmits a cochlear vein in life (which is observed traveling with the membranous aqueduct of various mammal species) [30]–[31]. The ventral canal for the cochlear vein is more delicate than the canaliculus cochleae (cc and cv in Figure 13, slice 99), and forms a straight tube that widens slightly before it opens on the medial surface of the petrosal. Unlike the canal for the cochlear vein, the canaliculus cochleae widens rapidly as it extends away from the cochlea, forming a pyramid shaped conduit for the cochlear aqueduct (cc in Figure 12C). The canaliculus cochleae is longer than the channel in either *Chrysochloris* or *Hemicentetes* (Table 2). The canaliculus is more easily observed in the CT scans of *Macroscelides* than in the data of afrosoricids, also (cc in Figure 13, slice 99).

The division between the spherical and elliptical recesses is not well defined in *Macroscelides*, although there is a slight constriction in the vestibule lateral to the fenestra vestibuli (Figure 12A). The elliptical recess of the vestibule is not curved in *Macroscelides*, but the chamber contains five major openings, as also is observed in the bony labyrinth of *Chrysochloris*, for the common crus, ampullae of the semicircular canals, and a separate opening for the posterior limb of the lateral semicircular canal near the vestibular aperture of the posterior ampulla (er in Figure 12B). The bony channel for the vestibular aqueduct exits the spherical recess anterior to the common crus at a greater distance than it does in *Didelphis* (av in Figure 13, slice 108). The channel is a straight tube of uniform diameter across most of its length until it turns posterolaterally and flattens into a fissure-like chamber before opening onto the endocranial surface of the petrosal (near the union of the posterior and anterior semicircular canals at the apex of the common crus; av in Figure 13, slice 135).

The semicircular canals form gentle curves, and the planes of the anterior and posterior canals nearly form a right angle (Table 3). The planes of the lateral and other two semicircular canals form obtuse angles. The anterior semicircular canal is the longest of the three canals, and the radius of the anterior canal arc is larger than either the lateral or posterior arc canals (Table 4). However, the cross-sectional diameter of the lumen of the posterior canal is larger than the anterior canal, although the largest diameter was measured for the lateral canal. The ratio of the slender portion of a canal over arc radius is greatest for the posterior semicircular canal (5.10). The ratio for the anterior canal is 4.24, and the ratio for the lateral canal is 4.00. The aspect ratio of the anterior semicircular canal arc is higher than the ratio of the arcs of either the lateral or posterior canal (Table 5). This result signifies that the arc of the anterior semicircular canal is more circular than the other canal arcs (ac in Figure 12C).

None of the semicircular canals fit onto a single plane, particularly the anterior canal where a substantial deviation was measured (Table 5). The posterior canal is the most planar of the three, although it is curved along its course and the deviation of the canal is substantial, whereas the deviation of the lateral canal is not (ratios of linear deviation to canal lumen diameter are 1.36 and 0.30 respectively). The course of the anterior limb of the lateral canal undulates as it opens into the vestibule, but the anterior limb is straight. The plane of the lateral semicircular canal is positioned high with respect to the posterior semicircular canal in *Macroscelides* so that the lateral canal divides the space enclosed by the posterior semicircular canal arc when the bony labyrinth is viewed anteriorly (lc in Figure 12A). The sagittal labyrinthine index for *Macroscelides* is greater than that measured for *Chrysochloris* (Table 3). A high index indicates a more dorsal position of the lateral semicircular canal. However, the lateral canal does not extend posterior to the plane of the posterior semicircular canal (lc and pc in Figure 12B–C), and the lateral extent of both canals is equivalent (lc and pc in Figure 12A–B).

Although *Macroscelides* holds a sistergroup relationship with afrosoricids in the supertrees reconstructed by Bininda-Emonds and others [66], there are no unambiguous otic synapomorphies uniting the clade. The lateral semicircular canal is derived relative to the ancestral eutherian state in that it takes a high position relative to the posterior canal, as is observed in *Chrysochloris* and *Hemicentetes*. The afrosoricids and *Macroscelides* lack secondary common crura, which are present in Cretaceous eutherians [37], [83]. Absence of a secondary common crus is derived for Placentalia, and therefore a feature that the clade consisting of Macroscelidea and Afrosoricida inherited from its placental ancestor. The anterior semicircular canal is the largest in terms of radius, indicating that the bony labyrinth of *Macroscelides* retains the ancestral therian condition in this regard.

#### Tubulidentata

**Figure 14:**
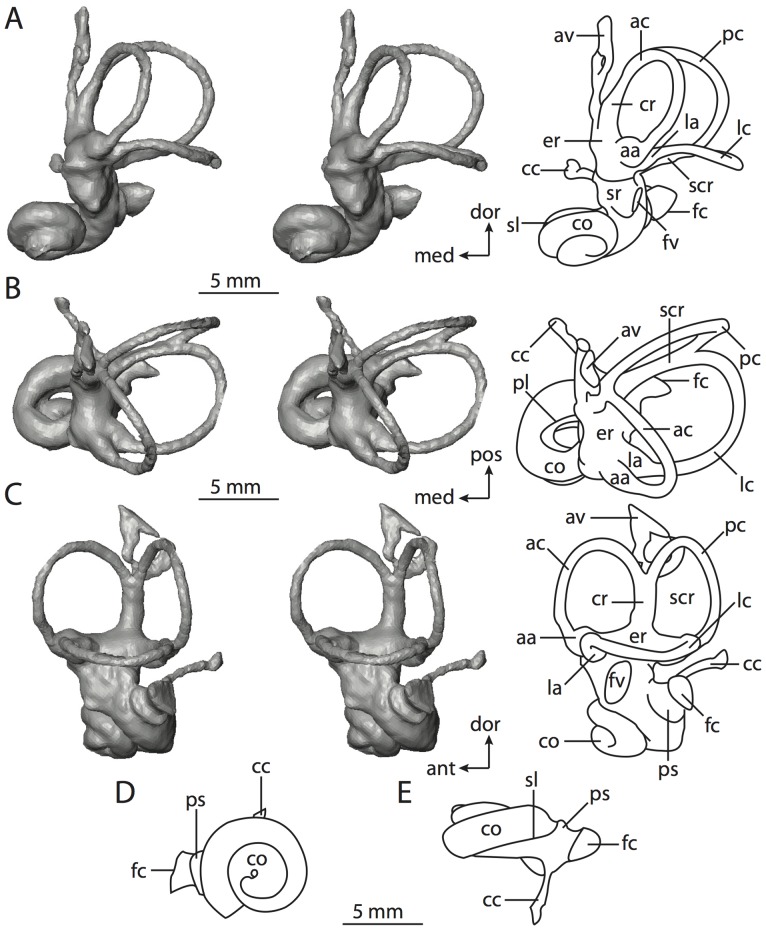
Bony labyrinth of*Orycteropus afer*. **A**, stereopair and labeled line drawing of digital endocast in anterior view; **B**, stereopair and labeled line drawing of digital endocast in dorsal view; **C**, stereopair and labeled line drawing of digital endocast in lateral view; **D**, line drawing of cochlea viewed down axis of rotation to display degree of coiling; **E**, line drawing of cochlea in profile. Abbreviations listed at the end of the Materials and Methods section.

**Figure 15:**
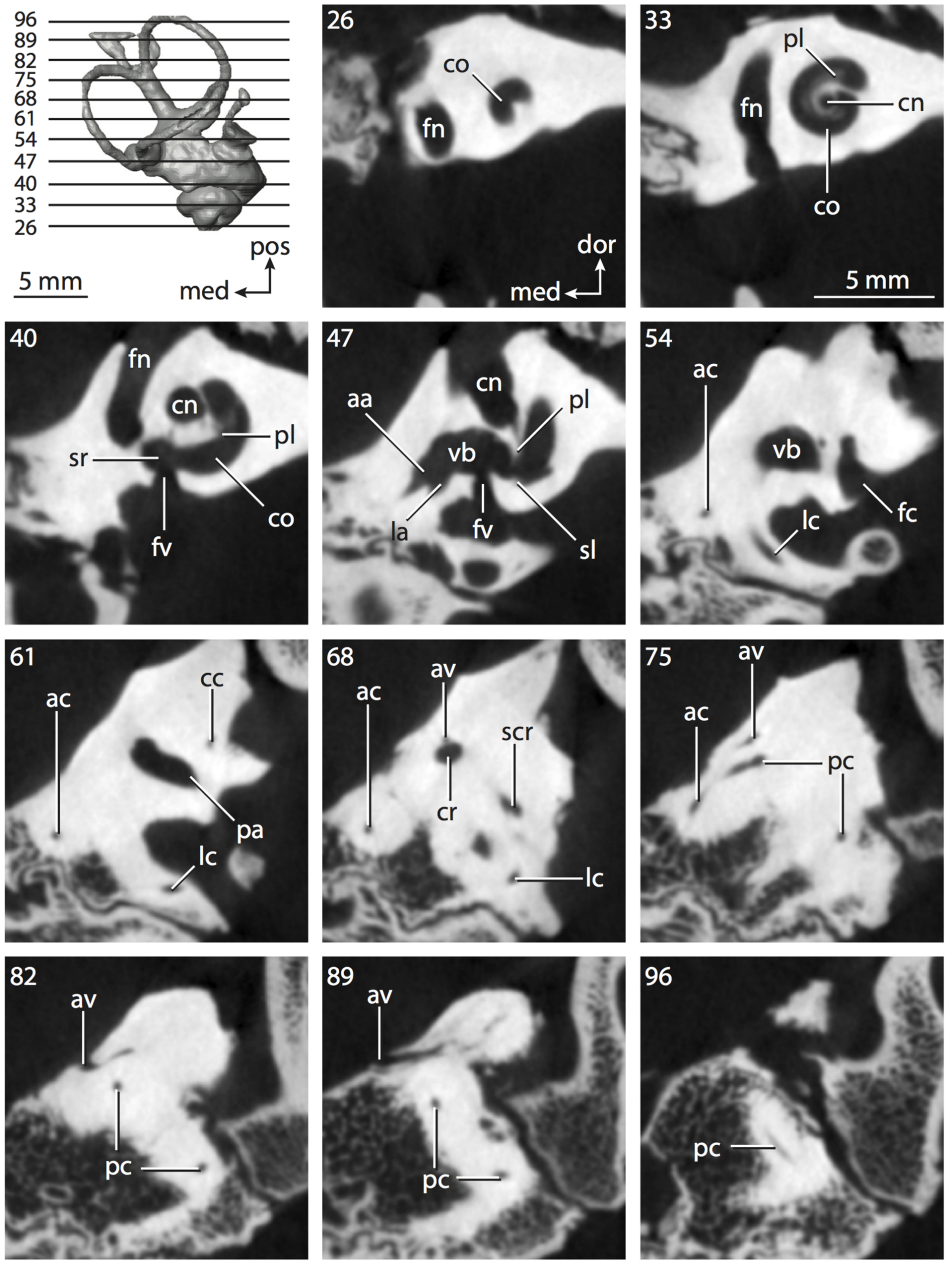
CT slices through ear region of*Orycteropus afer*. Abbreviations listed at the end of the Materials and Methods section.

*Orycteropus afer* (Figures 14 and 15) is the only extant member of Tubulidentata (aardvarks), although two additional genera of aardvarks (*Leptorycteropus* and *Myorycteropus*) were present during the Neogene of sub-Saharan Africa [120]. Morphological data do not support strong systematic placement of Tubulidentata among placental mammals, placing the clade either in a basal polytomy with most placental lineages [33], [102] or with weak associations to ungulates [105], [121]. Molecular evidence suggests a close relationship between aardvarks some ungulates [122], particularly Paenungulata [123], which includes Hyracoidea, Sirenia, and Proboscidea (sensu Simpson [100]). Both Tubulidentata and Paenungulata are included within Afrotheria, although the relationships among these taxa within the African clade are ambiguous [66], [98], [108], [112], [124]–[125].

The average body mass of *Orycteropus afer* is significantly greater than any of the other afrotherians described thus far (Table 1), and the mass of the specimen used in this study (AMNH 51909) is above the species average [89]. The large body size of *Orycteropus* is reflected in the bony labyrinth of the inner ear (Table 1). The cochlea of *Orycteropus* contributes a similar volumetric proportion of the total labyrinth to that calculated for the ancestor of Afrotheria (55% versus 56%), and the spiral of the cochlea is fairly flat (Figure 14E; Table 2). The cochlea completes nearly two turns, but the diameter of the second whorl is smaller than the basal turn, and therefore does not obscure the basal turn when the cochlea is viewed down the axis of rotation (Figure 14D).

The basal plane of the cochlea is rotated from the plane of the lateral semicircular canal (co and lc in Figure 14C), and the fenestra cochleae opens into the tympanic cavity at the end of a short stalk (fc in Figure 14A). A groove is situated behind the fenestra cochleae (expressed on the endocast as a curved ridge) that leads to the bony canaliculus cochleae (cc in Figure 14C and E). The canaliculus, which accommodates the cochlear aqueduct in life, is developed in *Orycteropus* as a slightly curved tube that widens at the tip as best observed in medial view. The secondary bony lamina that supports the basilar membrane in life (sl in Figure 15, slice 47) extends between one half and three quarters of the basal turn of the cochlear canal (Table 2).

A gentle constriction of the vestibule medial to the elliptical fenestra vestibuli (Table 3) divides the spherical and elliptical recesses, although the elliptical recess within the vestibule of *Orycteropus* is not divided into anterior and posterior chambers as is observed within the labyrinth of *Chrysochloris*. One striking feature of the vestibular apparatus of *Orycteropus* is that the lateral and posterior semicircular canals are joined to form a secondary common crus (scr in Figure 14B; Figure 15, slice 68), a structure that is present in *Didelphis* (scr in Figure 4B and F). The secondary common crus is flattened, and bony ridges (expressed as thin grooves on the endocast) divide the crus into a section for the lateral semicircular canal anteriorly, and the posterior canal posteriorly, although the two sections are continuous. The lateral canal does not extend posterior to the plane of the posterior canal, and the lateral extent of both canal arcs are equivalent.

The common crus between the anterior and posterior canals in *Orycteropus* (cr in Figure 14A) appears squat in relation to the semicircular canal arcs when compared to the labyrinth of *Macroscelides proboscideus* (cr in Figure 12). The bony channel for the vestibular aqueduct exits medial to the vestibular aperture for the common crus. The channel forms a straight tube for a little over half of its length, whereupon it bifurcates into an anterior and a posterior projection (av in Figures 14C and 15, slices 68 through 89). The anterior projection is wider than the posterior. The bifurcation of the channel for the vestibular aqueduct is unique to *Orycteropus* among all of the mammals considered in this study, and no mammal possesses a bifurcated membranous aqueduct [30]–[31]. It is unlikely that the soft-tissue aqueduct of *Orycteropus* is forked, although the membranous labyrinth of the species is poorly known.

The planes between the anterior and posterior semicircular canals approach a right angle, and the angle between the posterior and lateral canals are not far from 90° (Table 3). The angle between the anterior and lateral semicircular canal planes is noticeably acute. The posterior semicircular canal is larger than the anterior and lateral in terms of the length of the slender portion of the canal, diameter of canal in cross-section, and arc radius of curvature (Table 4). This pattern is not observed in either the afrosoricids or *Macroscelides*, in which the anterior canal tends to be the largest. The dimensions of the anterior semicircular canal of *Orycteropus* are the smallest among the three canals, including the aspect ratio of the anterior semicircular canal arc (Table 5). The arc of the lateral semicircular canal approaches a circle, and the height of the posterior semicircular canal arc is greater than the width. The ratio between the length of the slender portion of the semicircular canal and arc radius for the anterior, lateral, and posterior semicircular canals are 4.96, 5.03, and 5.39 respectively.

The lateral semicircular canal is the most planar among the three (Table 5) with a ratio of the total linear deviation over cross-sectional diameter of the canal of 0.78. However, the anterior limb of the lateral canal is undulated as it enters the anterior ampulla. The anterior canal deviates from its plane more than the posterior canal. The posterior semicircular canal curves anteriorly along its course (pc in Figure 14C). The deviation of both the anterior and posterior semicircular canals are substantial with ratios of linear deviation over canal diameter of 1.82 and 1.06 respectively.

The bony labyrinth of *Orycteropus* possesses two features that are observed in *Kulbeckia* and other Cretaceous eutherians [37], [83], which are the secondary common crus and the low position of the lateral semicircular canal relative to the posterior canal. Such plesiomorphic features support a basal position of Tubulidentata within Afrotheria as recovered using molecular data [124], [126].

If *Orycteropus* is the sister taxon to all other afrotherians, then absence of the secondary common crus (or separate openings for the lateral and posterior semicircular canals into the vestibule) might be a synapomorphy uniting the remaining afrotheres. However, the absence of the secondary common crus is reconstructed as a synapomorphy for all of Placentalia based on the phylogeny used here (Figure 2). Thus, the presence of the secondary crus is an autapomorphy for *Orycteropus*. It is interesting, however, that the bony labyrinth of *Orycteropus* possesses a few apomorphies (when compared to stem eutherians). For example, the cochlea completes over one and a half turns (almost two), and the posterior semicircular canal has the largest radius, rather than the anterior canal.

#### Hyracoidea

A close relationship between Hyracoidea (hyraxes) and ungulates, particularly either Perissodactyla or Tethytheria (Sirenia+Proboscidea), is a classical hypothesis. Although some morphological data support a sistergroup relationship between Hyracoidea and Perissodactyla [101], [127]–[128], the majority of morphological [33], [102], [104]–[105], [129]–[130] and molecular data [66], [98], [109]–[110], [112]–[113], [130] support a pairing of Hyracoidea and Tethytheria within the group Paenungulata. The results of most phylogenetic analyses recover a sistergroup relationship between Hyracoidea and a monophyletic Tethytheria, but the results of a few recent analyses, including the supertree used here [66], support a closer relationship between Hyracoidea and Sirenia, rendering Tethytheria paraphyletic.

**Figure 16:**
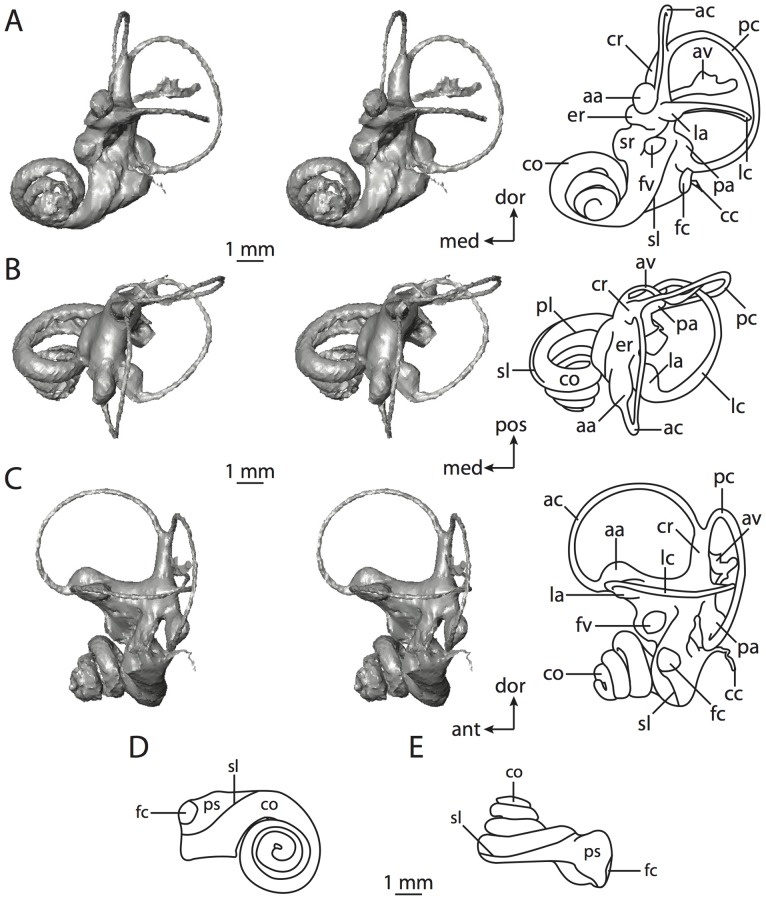
Bony labyrinth of*Procavia capensis*. **A**, stereopair and labeled line drawing of digital endocast in anterior view; **B**, stereopair and labeled line drawing of digital endocast in dorsal view; **C**, stereopair and labeled line drawing of digital endocast in lateral view; **D**, line drawing of cochlea viewed down axis of rotation to display degree of coiling; **E**, line drawing of cochlea in profile. Abbreviations listed at the end of the Materials and Methods section.

A digital endocast of *Procavia capensis* was constructed (Figure 16) to examine the labyrinth of Hyracoidea. *Procavia* is a little larger than a house cat in overall body mass [89],. and the cochlea contributes 48% of the total volume of the inner ear cavities. The percentage of volume of the bony labyrinth that is made up by the cochlea is relatively low among the afrotherians investigated, although larger than that of the elephantimorph (the cochlea of which comprises 31%, as described below).

The aspect ratio of the cochlear spiral is large when compared to most of the other afrotherians examined (Figure 16E), although the aspect ratio of *Macroscelides proboscideus* is larger (Table 2). However, the overall shape of the cochlea is different between *Procavia* and *Macroscelides*. Whereas the apical turn sits upon the basal turn of the cochlea in *Macroscelides*, the apical turns of *Procavia* fit within the arc formed by the basal turn (Figure 16D). Such morphology gives the cochlea of *Procavia* a conical appearance, rather than the cylindrical shape of *Macroscelides* and *Chrysochloris*.

The cochlea of *Procavia* completes over three and three quarters turns (Table 2), a greater degree than in any other afrotherian. The secondary bony lamina of *Procavia* (sl in Figure 16) is not as well developed as the structure is in *Macroscelides*, and only extends a short distance past the first half of the basal turn of the cochlea (sl in Figure 16A and B). The plane of the basal turn deviates from that of the lateral canal to a greater degree than all other afrotherians except the elephantimorph (Table 2). The degree of rotation exhibited by the basal plane of the cochlea might be a synapomorphy for Paenungulata, although the cochlea of *Trichechus* only deviates from the plane of the lateral canal by a small amount. The bony canaliculus cochleae for transmission of the cochlear aqueduct isslender and is not observed easily in the CT slices, having a maximum diameter of a single voxel (around 0.07 mm) at several points along its path. The canaliculus is not a straight tube, but rather hooks laterally. The canaliculus cochleae exits the bony labyrinth from a bulge posteromedial to the fenestra cochleae (cc in Figure 16C).

The spherical recess of the vestibule is distinguished easily from the elliptical recess particularly at the anterior aspect of the vestibule (sr and er in Figure 16A), dorsal to the elliptical fenestra vestibuli (stapedial ratio in Table 3). Despite its name, the spherical recess is ovoid in shape. However, the elliptical recess is much more elongated, and it gently curves laterally. The anterior end of the elliptical recess opens into the anterior and lateral ampullae, and the posterior end of the recess leads to the vestibular apertures of the posterior ampulla, common crus, and posterior limb of the lateral semicircular canal. The lateral canal enters the vestibule closer to the common crus than it does to the posterior ampulla, which causes the lateral semicircular canal to divide the space enclosed by the arc of the posterior semicircular canal into dorsal and ventral regions in anterior view (lc in Figure 16A). Furthermore, the lateral semicircular canal extends posterior to the plane of the posterior canal (lc and pc in Figure 16B and C), but the lateral canal does not extend as far laterally as its posterior counterpart (lc and pc in Figure 16A and B). The sagittal labyrinthine index for *Procavia* is over twice that observed in *Chrysochloris* and greater than that in *Macroscelides* (Table 3).

**Figure 17:**
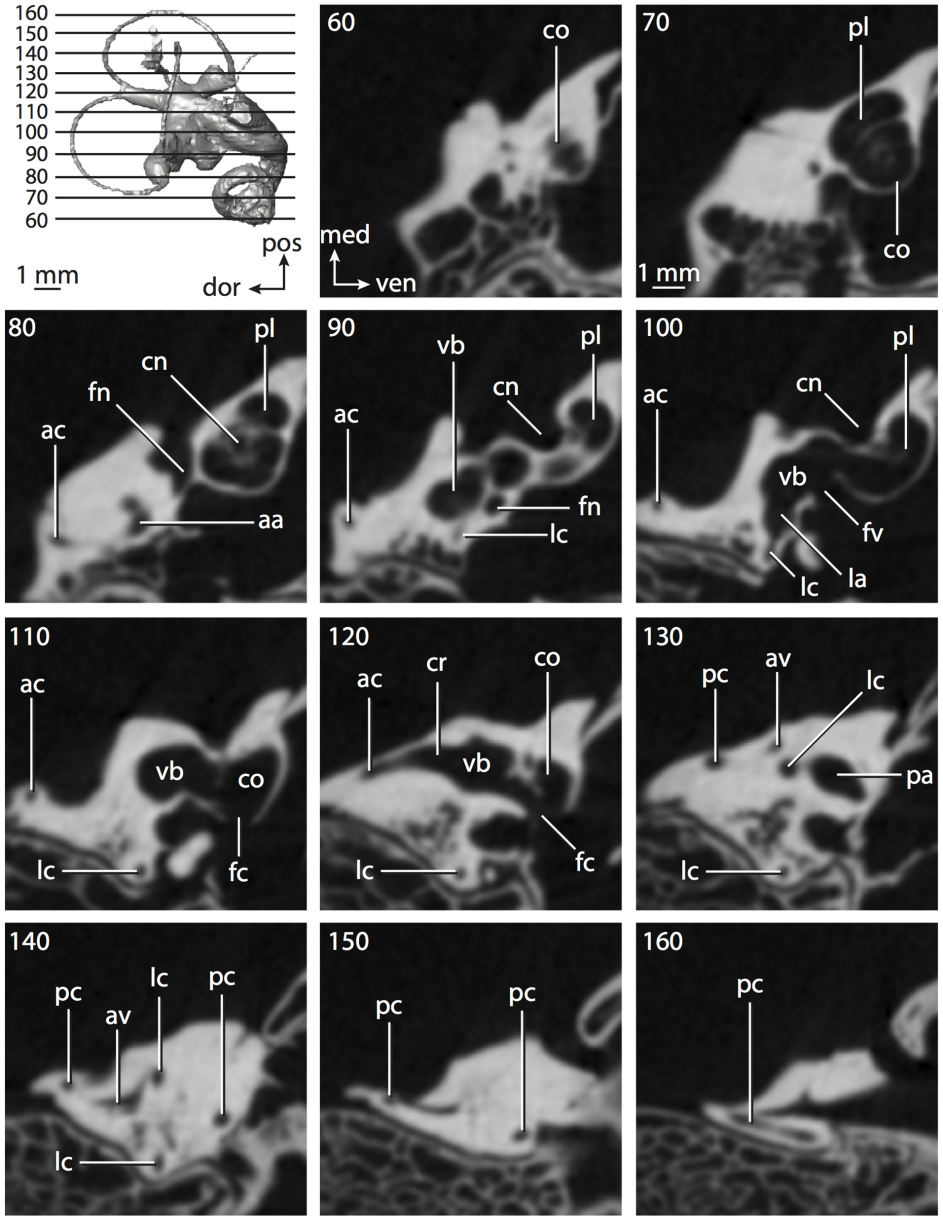
CT slices through ear region of*Procavia capensis*. Abbreviations listed at the end of the Materials and Methods section.

The bony channel for the vestibular aqueduct exits medial and ventral to the vestibular aperture of the common crus (cr and av in Figure 17, slice 120 through 130), and it is almost three times longer than the canaliculus cochleae (Tables 2 and 3). The channel curves laterally along the posterior border of the base of the common crus (av in Figure 16B), but the aqueduct does not cross the rise of the common crus when the bony labyrinth is viewed medially. The channel for the vestibular aqueduct is a uniformly subcircular tube in cross-section, until it flares and flattens into a fissure nearly on the plane of the posterior semicircular canal arc (av in Figure 17, slice 140).

The greatest angle between the planes of two semicircular canals in *Procavia* was measured between the anterior and posterior canals (Table 3). The angle between the anterior and lateral canals is almost as acute as the angle between the lateral and posterior canals is obtuse. As in *Orycteropus afer*, the posterior semicircular canal of *Procavia* is the most voluminous (0.41 mm^3^), as well as the longest of the three canals, and the arc radius of curvature of the posterior semicircular canal arc is greater for the posterior canal than for the others (Table 4). Both the anterior and lateral semicircular canals have a volume of 0.37 mm^3^ each. Among afrotherians, the posterior canal is the largest only in *Procavia* and *Orycteropus* and is a potential synapomorphy uniting the two taxa. A sistergroup relationship between aardvarks and hyraxes has not been proposed, although Hyracoidea has been placed in a polytomy along with Tubulidentata and Sirenia (sister taxon to polytomy is Proboscidea [123]). The arcs of the three semicircular canals are wider than they are high, and the aspect ratio of the arcs of the anterior and lateral canals are similar (Table 5). The ratio of the length of the slender portion of the canal over semicircular canal arc radius is greatest for the anterior canal, at 5.14 (lateral equals 4.28; posterior equals 4.90).

No semicircular canal fits onto a single plane in *Procavia*, although the angular deviation is not great for any canal. The anterior canal shows the largest angular deviation of a canal from its plane, although this is not much different than the angular deviations of the lateral and posterior canals (Table 5). Even so, only the deviation of the anterior canal is considered substantial, with the ratio of total linear deviation of the anterior canal over the cross-sectional diameter of the canal equaling 1.28 (ratios for the lateral and posterior canals are 0.54 and 0.88 respectively). The arc of the posterior semicircular canal curves posteriorly (pc in Figure 16C), and the anterior limb of the lateral canals takes a slightly undulating course as it joins with the lateral ampulla.

There is no unambiguous support for paenungulate monophyly in the bony labyrinth. Hyraxes retain the ancestral placental condition in that the secondary common crus is absent, but are derived from the placental ancestor in that the posterior canal is largest in terms of arc radius, rather than the anterior canal. Among the remaining afrotherians, only *Orycteropus* shares the condition of having the largest arc in the posterior semicircular canal. The labyrinth of *Procavia* is derived from the ancestral eutherian condition in that the lateral semicircular canal is positioned dorsal with respect to the ampullar entrance of the posterior semicircular canal.

#### Sirenia

Dugongs and manatees comprise the clade Sirenia. Sirenia and Cetacea are the only two exclusively aquatic groups of extant mammals, although they are not closely related despite similar lifestyles and superficial resemblances (e.g., fusiform body and short neck). Rather, there is a closer connection between Sirenia and Proboscidea, which is a relationship that has been recognized for several centuries [100]–[101], [131].

Monophyly of Tethytheria, which is the clade that includes Sirenia, Proboscidea, as well as the extinct groups Desmostylia, “Anthrocobunidae”, and Embrithopoda [100], [132], is supported by more recent morphological [33], [102], [128], [133] and molecular evidence [112], [134]. However, the results of a few recent molecular analyses [66], [68], [98] refute tethytherian monophyly, while still recovering a close relationship between Sirenia and Proboscidea within Paenungulata.

**Figure 18:**
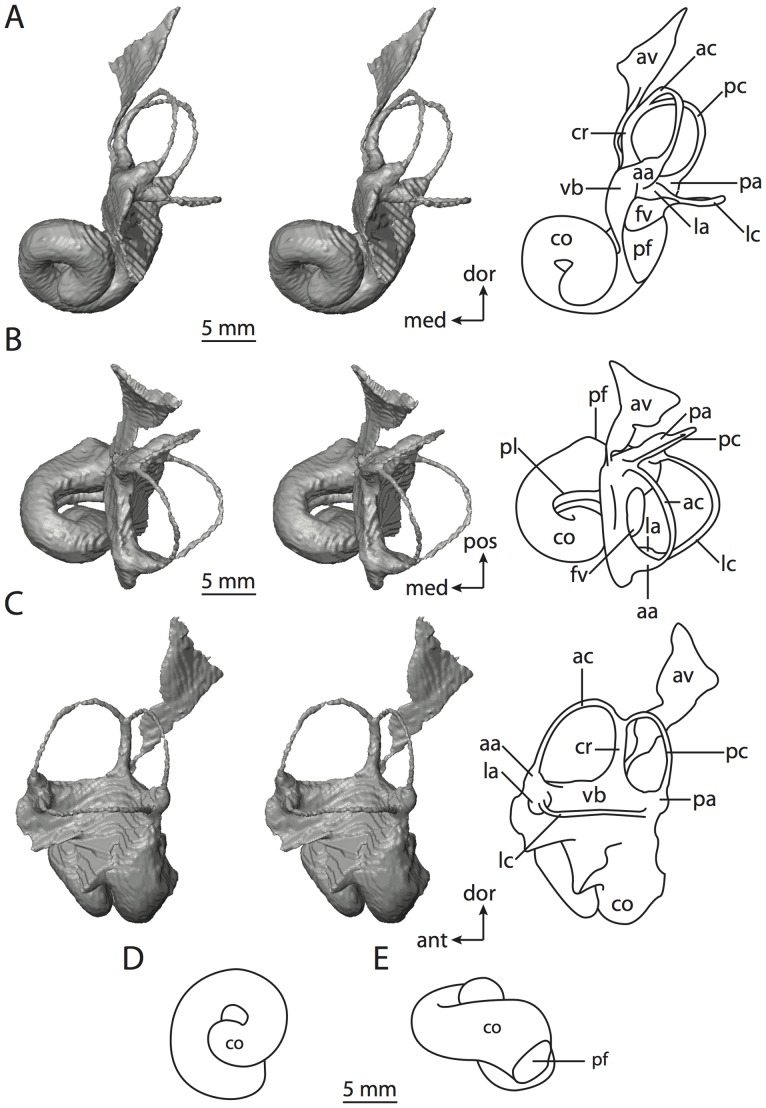
Bony labyrinth of*Trichechus manatus*. **A**, stereopair and labeled line drawing of digital endocast in anterior view; **B**, stereopair and labeled line drawing of digital endocast in dorsal view; **C**, stereopair and labeled line drawing of digital endocast in lateral view; **D**, line drawing of cochlea viewed down axis of rotation to display degree of coiling; **E**, line drawing of cochlea in profile. Abbreviations listed at the end of the Materials and Methods section.

**Figure 19:**
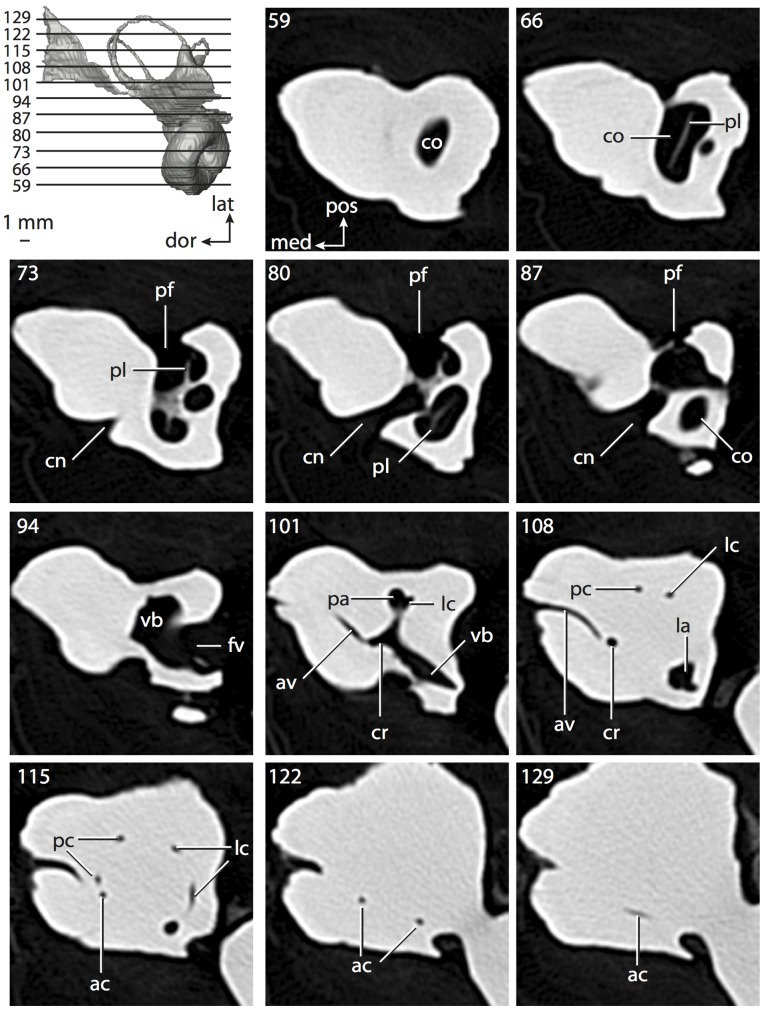
CT slices through ear region of*Trichechus manatus*. Abbreviations listed at the end of the Materials and Methods section.

The Florida manatee, *Trichechus manatus*, represents Sirenia. The most notable feature observable on the digital endocast of *Trichechus* is the absence of the bony canaliculus cochleae for transmission of the cochlear aqueduct (Figures 18 and 19). Rather, the canaliculus and fenestra cochleae are fused to form an undivided perilymphatic foramen (pf in Figure 18A and E; Figure 19, slices 73 through 87), which is unique to Sirenia, Proboscidea, and gray whales among extant mammals. Although the three living species of manatees and the dugong possess an undivided perlymphatic foramen, the bony canaliculus cochleae is separate from the fenestra cochleae in the Eocene sirenian *Prorastamus*, suggesting that the undivided perilymphatic foramen either evolved independently in Sirenia and Proboscidea [133], [135], or it is a reversal in the Eocene sirenian.

The cochlea is large with respect to the entire bony labyrinth, contributing 71% of the entire labyrinthine volume. Although this is a greater contribution than the cochleae of either the ancestral afrotherian (56%) or paenungulate (48%), the value is not much different from the volumetric percentages calculated for *Chrysochloris* sp. (71%) and *Macroscelides proboscideus* (72%). However, the aspect ratio of the spiral of the cochlea is low in *Trichechus* (Figure 18E) relative to the cochlea of either *Chrysochloris* or *Macroscelides* (Table 2). The cochlea of *Trichechus* completes just over a single turn, which is less than any other afrotherian examined, and almost a complete turn lower than that calculated for the ancestor of Afrotheria (751°). A low degree of coiling may be a synapomorphy for Sirenia, given that a similar degree of coiling is observed in the fossil *Hydrodamalis gigas* [136]. The length of the canal from the base to the apex is longer in *Trichechus* than in other afrotherians excepting proboscideans (Table 2). As in proboscideans (described below), the secondary bony lamina is not present in *Trichechus* (Figure 19).

The fenestra vestibuli has a low aspect (stapedial) ratio (Table 3), which signifies a less elliptical window than other afrotherians. The spherical recess of the vestibule, which communicates with the tympanic cavity via the fenestra vestibuli, is poorly developed, and not distinguishable from the elliptical recess. In fact, the vestibule as a whole is mediolaterally compressed as can be seen when the bony labyrinth is in anterior view (vb in Figures 18A and 19, slice 101). The thickest part of the vestibule is at the anterior end, where a slight laterodorsal projection leads to the anterior and lateral ampullae. The ampullae of the semicircular canals are proportionately smaller in *Trichechus* than in other taxa examined, such as *Macroscelides* and *Procavia*.

Apertures for the posterior ampulla, common crus, and the posterior limb of the lateral semicircular canal are situated at the posterior end of the vestibule, with the common crus as the medial-most opening (cr in Figure 18C). The bony channel for the vestibular aqueduct exits the bony labyrinth ventromedial to the vestibular aperture of the common crus. The channel for the aqueduct extends from the vestibule as a round tube for a very short distance before opening into a broad fissure that flares posterodorsally. The bony channel is nearly two thirds as long as the total length of the bony labyrinth (Tables 1 and 2).

The vestibular aperture of the lateral semicircular canal opens near the base of the posterior ampulla in *Trichechus*, similar to the state observed in *Macroscelides*. However, the lateral canal enters the vestibule lateral and ventral to the posterior ampulla in *Trichechus*, which is on the opposite side of the posterior ampulla from *Macroscelides* and other taxa where the opening for the canal is well separated from the ampulla, such as *Procavia*. Even in *Orycteropus*, where the lateral and posterior canals fuse to form a secondary common crus, the lateral canal is situated dorsal and slightly medial to the posterior canal. The morphology observed in *Trichechus* places the plane of the lateral semicircular canal relatively low on the vestibule, and the lateral canal does not extend posterior to the plane of the posterior canal. However, the lateral canal extends further laterally than the arc of the posterior canal.

All of the planes of the semicircular canals of *Trichechus* form acute angles with each other. The angle between the planes of the anterior and lateral semicircular canals is the smallest angle measured between any two canals in any afrotherian specimen (Table 3). Within *Trichechus*, the only canals that approach a right angle are the anterior and posterior canals. No single semicircular canal within the bony labyrinth of *Trichechus* is the greatest in all dimensions measured (Table 4). The radius of the arc of the lateral semicircular canal is larger than the arc of the posterior canal by nearly a millimeter, but only slightly larger than the arc of the anterior canal. Both the diameter of the lumen of the lateral semicircular canal, as well as the volume of the canal (2.5 mm^3^) are the largest among the three canals. However, the length of the slender portion of the lateral semicircular canal is noticeably smaller than both the anterior) and posterior semicircular canals (Table 4).

The posterior limbs of the anterior and lateral semicircular canals form steeper slopes than the anterior limbs (ac and lc in Figure 18B and C). Although the anterior canal is curved along its entire course, the anterior limb of the lateral canal is flattened, giving the arc of the canal an angular appearance at its midpoint, before the posterior limb curves gradually to meet the vestibule. The arc of the posterior semicircular canal is noticeably higher than the other two canals, with an aspect ratio of 1.18. The aspect ratio of the anterior canal arc is similar to that of the lateral canal (Table 5). The ratio of the length of the slender portion of the canal over semicircular canal arc radius is greatest for the posterior canal (4.67), followed by the anterior canal (4.02). The ratio for the lateral canal equals 3.18. The posterior semicircular canal does not deviate from its plane, and the anterior canal is more planar than the lateral canal (Table 5). The deviations exhibited by both the anterior and lateral semicircular canals are substantial (ratios of total linear deviation over cross-sectional diameter are 1.17 and 1.33 respectively), and the anterior limb of the lateral canal takes an undulating course into the lateral ampulla (lc in Figure 18A).

Although the results of several recent molecular analyses do not support the monophyly of Tethytheria, the structure of the inner ear supports a sistergroup relationship between Sirenia and Proboscidea among the paenungulates. Notable labyrinthine features that are shared by Sirenia and Proboscidea within Paenungulata are a low position of the lateral semicircular canal compared to the posterior canal (the lateral canal is high in *Procavia*), and a low cochlear spiral. The large radius of the lateral semicircular canal is an autapomorphy for *Trichechus* compared to all other afrotherians. The bony labyrinth of *Trichechus* retains the ancestral placental condition of the lateral semicircular canal opening directly into the vestibule in the absence of a secondary common crus.

#### Proboscidea

The bony labyrinth of a specimen of an extinct elephantimorph (likely *Mammuthus*) was described elsewhere [84], but for the sake of comparison, a brief overview of the inner ear anatomy of Proboscidea is provided here. Not only are proboscideans the largest afrotherians, as is reflected in the volume and length of the inner ear (Table 1), they are the largest extant terrestrial mammal [89]. Because the species of the proboscidean used for this study is not known with certainty [84], the body mass of the individual could not be estimated. Extant Proboscidea is not a taxonomically diverse clade, with no more than three species [137]–[138], but proboscidean diversity was much greater throughout the Tertiary period [107].

**Figure 20:**
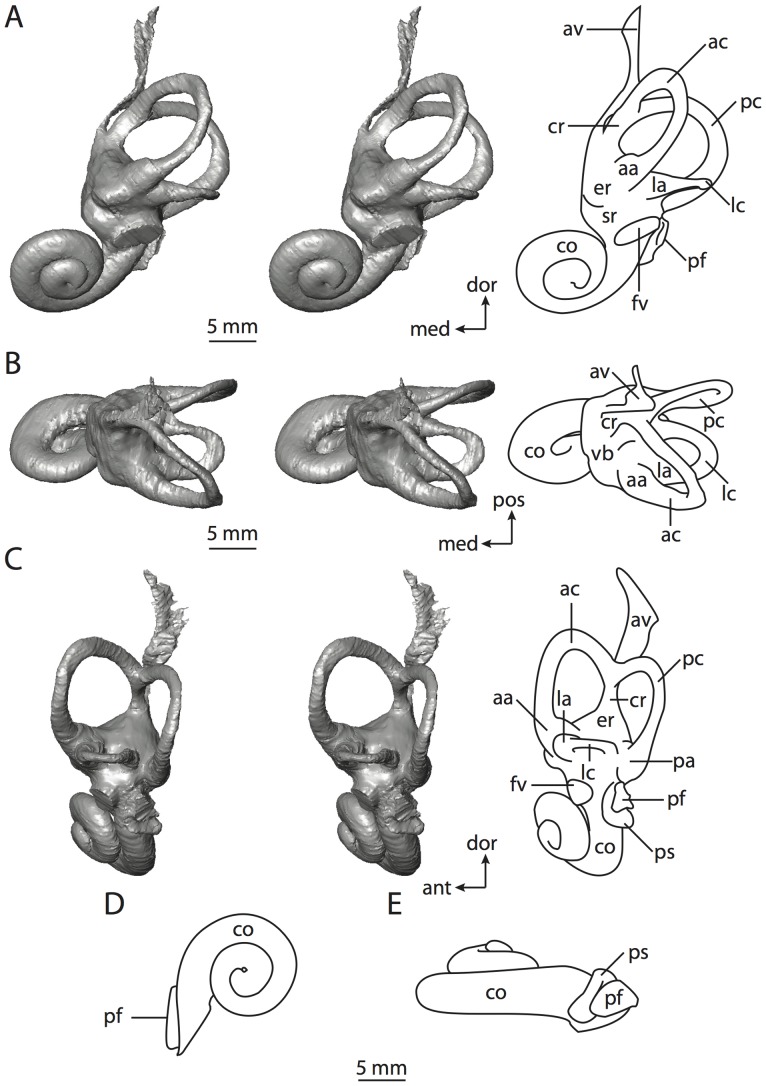
Bony labyrinth of the fossil elephantimorph proboscidean. The endocast is figured in different orientations elsewhere [84]. **A**, stereopair and labeled line drawing of digital endocast in anterior view; **B**, stereopair and labeled line drawing of digital endocast in dorsal view; **C**, stereopair and labeled line drawing of digital endocast in lateral view; **D**, line drawing of cochlea viewed down axis of rotation to display degree of coiling; **E**, line drawing of cochlea in profile. Abbreviations listed at the end of the Materials and Methods section.

**Figure 21:**
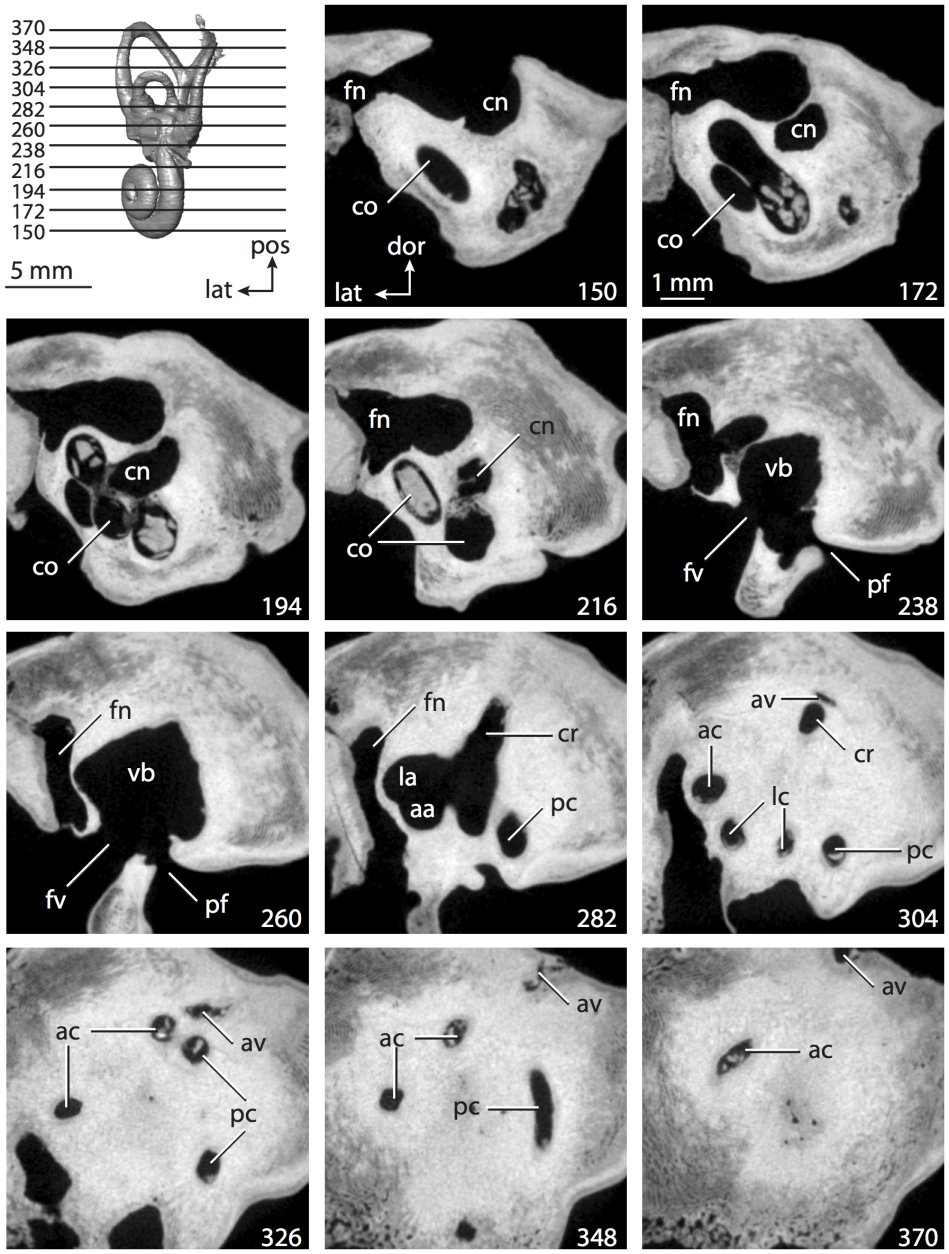
CT slices through ear region of the fossil elephantimorph proboscidean. Anatomy of the inner ear of this specimen was described elsewhere [84]. Abbreviations listed at the end of the Materials and Methods section.

As mentioned in the description of the bony labyrinth of the sirenian *Trichechus manatus*, the canaliculus cochleae for the cochlear aqueduct is absent in the elephantimorph (Figures 20 and 21), which is an apomorphic condition for Tethytheria. Rather, both the elephantimorph and *Trichechus* share a secondarily undivided perilymphatic foramen in lieu of a fenestra cochleae (pf in Figure 20C and E; Figure 21, slices 238 through 260), although this condition may have an independent derivation in both clades [133], [135]. The stapedial ratio measured from the fenestra vestibuli of the elephantimorph is is similar to that calculated for *Trichechus* (Table 3). A round fenestra also is characteristic of the extinct embrythopod *Arsinotherium*, which is closely related to Tethytheria [107], [132], if not within Tethytheria itself as the sister taxon of Proboscidea [135].

The cochlea of the elephantimorph completes a little over two whorls (Table 2) and contributes only 31% of the total volume of the inner ear, which is the smallest contribution observed among the afrotherian sample investigated. Although the total volume of the inner ear is greater in the elephantimorph than any of the other afrotherians, the volume of the cochlea is less than that measured for *Trichechus* (Table 2). The secondary bony lamina is not developed in Proboscidea (Figure 21), and the cochlea is fairly planispiral in the elephantimorph with a low aspect ratio (Figure 20E). The only other afrotherian to have a lower aspect ratio is *Hemicentetes* (Table 2).

The vestibular aperture of the posterior limb of the lateral semicircular canal is situated anterior to the posterior ampulla in the elephantimorph. The bony channel for the vestibular aqueduct leaves the vestibule medial to the common crus, and the aqueduct exits the petrosal via a fissure on the endocranial surface of the bone. The posterior limb of the lateral semicircular canal enters the vestibule separately from the posterior ampulla along a slightly undulating course (the anterior limb is straight as it enters the lateral ampulla). The lateral semicircular canal does not extend as far laterally as the posterior canal (lc and pc in Figure 20A and B), nor does it extend posterior to the posterior canal arc (lc and pc in Figure 20B and C).

The angle between the planes of the basal turn of the cochlea and the lateral semicircular canal is greater for the elephantimorph than any other afrotherian (Table 2). The most acute angle between the planes of two semicircular canals is between the anterior and lateral canals, and the most obtuse angle was measured between the posterior and lateral canals (Table 3). The arc radius of the posterior semicircular canal is larger than both the anterior and lateral canals, as is the diameter of the posterior canal in cross-section (Table 4). However, the length of the slender portion of the anterior semicircular canal is greater than either of the other canals.

The arcs of the lateral and posterior semicircular canals are higher than they are wide in the elephantimorph (Tabe 5). The ratios between the length of the slender portion of the canal and arc radius of the anterior canal is 4.93, which is the largest ratio among the three canals, and 4.41 for the posterior canal, which is the smallest value. The ratio for the lateral canal is 4.70. The canals do not deviate from their planes substantially (ratio of total linear deviation over cross-sectional area for the anterior canal equals 0.87; lateral canal equals 0.08; posterior canal equals 0.71; Table 5); however the posterior semicircular canal takes a sigmoidal course (pc in Figure 20B).

The bony labyrinth of the elephantimorph retains the primitive eutherian morphology observed in *Kulbeckia*, although the proboscidean inner ear is derived in the absence of the secondary common crus. There are no unambiguous characters within the bony labyrinth to support monophyly of Tethytheria to the exclusion of all other afrotherians, although among the paenungulates, both the elephantimorph and *Trichechus* share a flattened cochlea and a low position of the lateral semicircular canals (which are both the ancestral condition for Eutheria). The ancestral paenungulate state for both of those characters is equivocal.

#### Xenarthra

There are two major groups of xenarthrans, the armadillos and extinct glyptodonts that belong to Cingulata, and the anteaters and sloths, which make up the clade Pilosa [107]. Xenarthra often occupies a basal position in placental mammal phylogenies reconstructed using both morphological [102] and molecular analyses [98], [112]. A close relationship between Xenarthra and Pholidota (pangolins) within a group called Edentata has been proposed based on morphology (as recently as the mid-1980’s [33]), but such anatomical similarities, which include adaptations for a fossorial lifestyle and a reduction in teeth, are considered homoplastic in light of more recent phylogenetic analyses and evolutionary discussions [66], [139]. Further, nearly all molecular analyses group Pholidota with other placental clades separate from Xenarthra [66], [68], [98], [108], [112]–[113], [121]–[122], [140]–[141]. At least one exception is a study using protein sequences that recovered a sistergroup relationship between Xenarthra and Pholidota [142].

**Figure 22:**
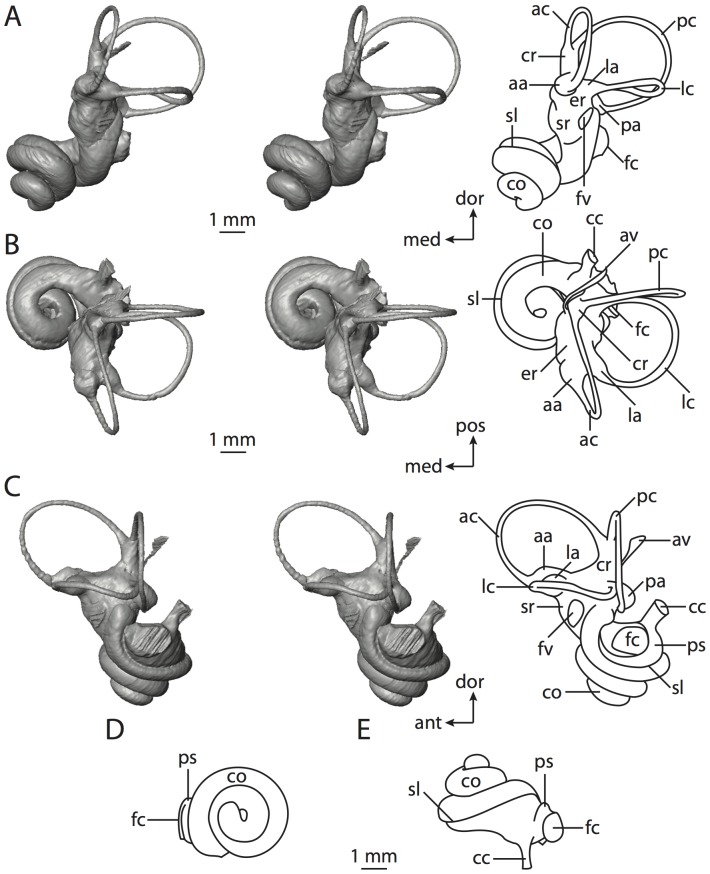
Bony labyrinth of*Dasypus novemcinctus*. **A**, stereopair and labeled line drawing of digital endocast in anterior view; **B**, stereopair and labeled line drawing of digital endocast in dorsal view; **C**, stereopair and labeled line drawing of digital endocast in lateral view; **D**, line drawing of cochlea viewed down axis of rotation to display degree of coiling; **E**, line drawing of cochlea in profile. Abbreviations listed at the end of the Materials and Methods section.

**Figure 23:**
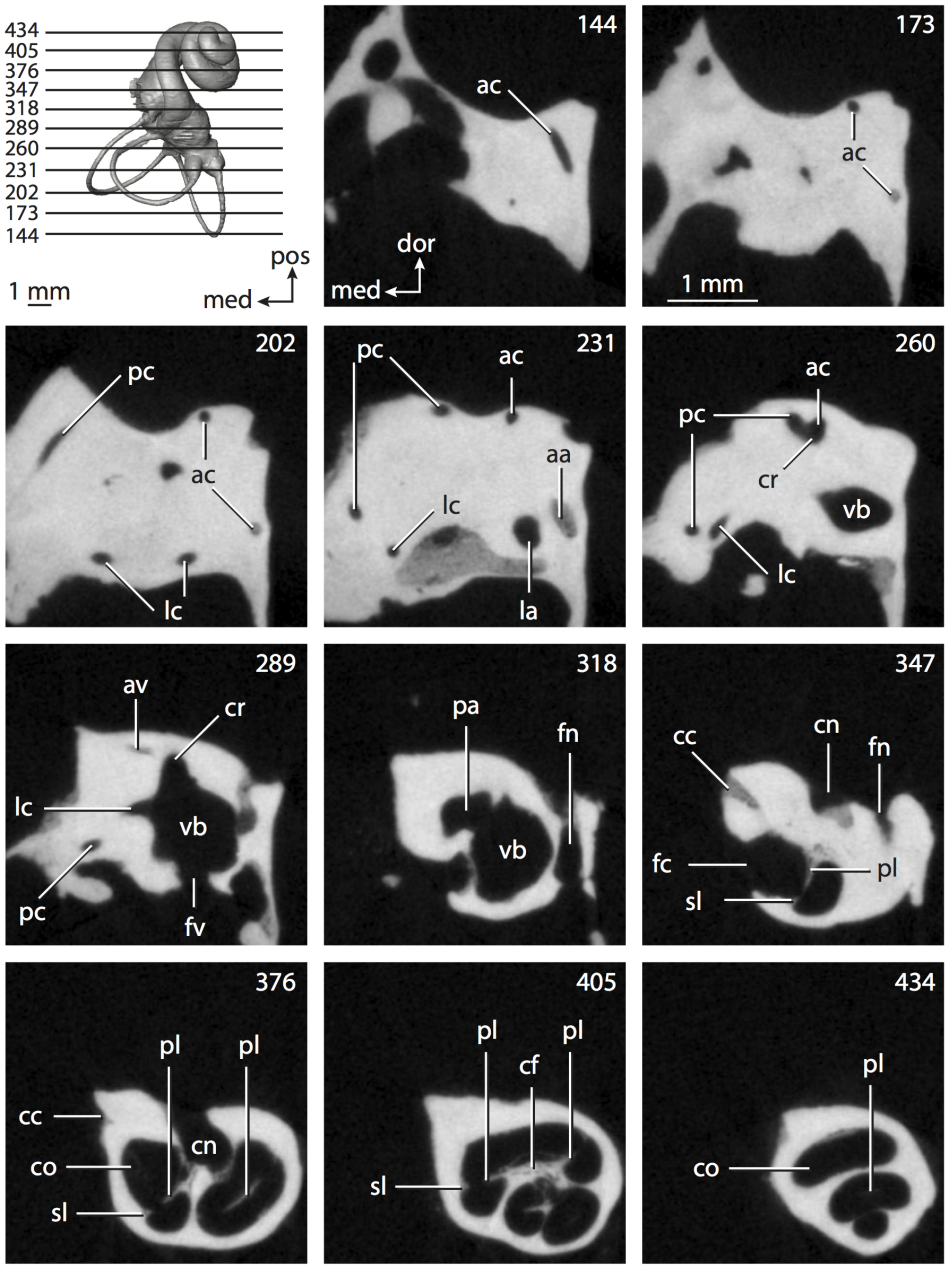
CT slices through ear region of*Dasypus novemcinctus.* Abbreviations listed at the end of the Materials and Methods section.

The nine-banded armadillo, *Dasypus novemcinctus*, which is the only xenarthran found in the United States, represents Xenarthra in this study. *Dasypus* as a genus is known from the Pliocene to Recent in North, Central, and South America [107], and *D. novemcinctus* itself has the largest biogeographical distribution of any xenarthran species [143]. Intraspecific variation within the inner ear of *D. novemcinctus* was discussed previously [83], [144], and a more thorough description of the bony labyrinth of this species is provided here (Figures 22–23). General dimensions of the bony labyrinth of *Dasypus* are provided in Table 1, including total length and the volume. The cochlea itself contributes 66% of the volume of the bony labyrinth (see Table 2), which is larger than that reconstructed for the ancestors of both Placentalia (58%) and Afrotheria (56%). Further dimensions of the cochlea are provided in Table 2, and dimensions and orientations of the semicircular canals are reported in Tables 3–5.

The cochlea completes nearly two and a quarter turns, and the diameters of the apical whorls of the cochlea are smaller than the basal turn of the cochlea (unlike the condition observed in *Macroscelides*), although the successive whorls sit upon the basal turn (Figure 22D). The aspect ratio of the cochlear spiral in profile is 0 the same that was calculated for the afrosoricid *Chrysochloris* (Table 2). As was observed within the cochlea of *Macroscelides*, the secondary bony lamina is well developed in *Dasypus* (sl in Figure 23, slices 347 through 405), and the structure extends past the basal turn (Table 2). The lamina is expressed as the distinct groove along the radial wall of the digital endocast (sl in Figure 22A through C and E), and the lamina curves around the dorsal border of the fenestra cochleae to define the posterior border of an inflation of the scala vestibuli between the fenestra cochleae and fenestra vestibuli. A secondary bony lamina is evident but unlabeled on a published endocast of the southern tamandua *Tamandua tetradactyla*, but the secondary lamina appears to be absent in the three-toed sloth *Bradypus tridactylus* [144]. Medial to the fenestra cochleae in *Dasypus*, an outpocketing of the scala tympani for the perilymphatic duct (ps in Figure 22C and E) leads to a robust canaliculus cochleae for transmission of the cochlear aqueduct (cc in Figure 22B, C, and E; Figure 23, slices 347 through 346).

The angle between the plane of the basal turn of the cochlea and the lateral semicircular canal is low for *Dasypus* when compared to other taxa that are described above (Table 2; lc in Figure 22C). The angle between the lateral semicircular canal and cochlea does not express much intraspecific variation in *D. novemcinctus* [83], [144], although the orientation of the canal is apparently quite variable in other xenarthrans, including *Bradypus* [144]. The spherical recess of the vestibule is distinguishable from the elliptical recess as the former bulges medially toward the axis of rotation of the cochlea (sr in Figure 22A). The anterior and lateral ampullae open into a small anterior chamber of the elliptical recess (expressed on the endocast as a short pedestal; Figure 22B and C). The posterior limb of the lateral semicircular canal takes an undulating course as it opens into the vestibule dorsal to the posterior ampulla (lc in Figure 22C), which gives the lateral canal a high position with respect to the rest of the vestibule. The lateral canal divides the space enclosed by the arc of the posterior semicircular canal when the labyrinth is in anterior view (lc in Figure 22A), and the sagittal labyrinthine index is slightly larger than that calculated for *Chrysochloris* (Table 3). The arc of the lateral semicircular canal does not extend posterior to that of the posterior canal (lc and pc in Figure 22B and C), nor does it extend as far laterally (lc and pc in Figure 22A and B).

The common crus appears stout with respect to the arcs of the semicircular canals (cr in Figure 22A and C). In that respect, the common crus of *Dasypus* more closely resembles *Bradypus* than *Tamandua* among other xenarthrans [144]. The bony channel for the vestibular aqueduct exits the labyrinth from a triangular excavation on the medial wall of the spherical recess, medioventral to the common crus. The canal for the vestibular aqueduct is longer than the canaliculus cochleae for the cochlear aqueduct (Tables 2 and 3), but is a more delicate structure overall (av in Figure 22B). The vestibular aqueduct only crosses the base of the common crus on its posterodorsal course.

All of the semicircular canal planes of *Dasypus* form acute angles with each other, although the angle between the planes of the posterior and lateral canals approaches a right angle (Table 3).

The posterior semicircular canal is the most planar of the three, although its inferior limb is slightly curved anteriorly, with a total angular deviation of 7.8° from its plane (pc in Figure 22C). The lateral canal is the least planar (18.1°) and takes an overall sigmoidal course (lc in Figure 22C). The angular deviation of the anterior semicircular canal from its plane is 13.0°, and the ratio of the total linear deviation over cross-sectional diameter is substantial for all three canals (anterior is 1.68; lateral is 2.13; posterior is 1.18). The posterior semicircular canal is the largest of the three canals in terms of length of the slender portion of the canal and arc radius of curvature (Table 4). However, the diameter of the lumen of the lateral semicircular canal is greater than either the anterior or posterior canal.

The area enclosed by the arc of the anterior semicircular canal is elliptical, as expressed by the aspect ratio of the arc (Table 5; ac in Figure 22C), whereas the area enclosed by the arc of the posterior semicircular canal is circular (pc in Figure 22A). The aspect ratio of the arc of the lateral semicircular inidcates that the height of the arc is greater than the width. The ratio of the length of the slender portion of the semicircular canal over the arc radius is 5.91 for the anterior semicircular canal, 4.63 for the lateral semicircular canal, and 5.88 for the posterior semicircular canal.

The bony labyrinth of *Dasypus* is derived in all respects to that of the ancestral eutherian, but retains the direct vestibular entry of the lateral semicircular canal from its placental ancestor. The plane of the lateral canal is high relative to the ampullar entry of the posterior canal, which is derived with respect to Eutheria, but the ancestral placental condition is unknown. Furthermore, the posterior semicircular canal of *Dasypus* is largest in terms of arc radius, rather than the anterior canal arc, and the aspect ratio of the cochlear spiral is high, giving the cochlea a high or “sharp-pointed” appearance. The aspect ratio of the cochlear spiral is conservative within *Dasypus* [83], [144], but the shape expresses substantial variation in *Bradypus* [144].

Although the labyrinth of *Dasypus* is derived with respect to the eutherian ancestral condition, there are no unambiguous characters within the labyrinth to support a closer relationship between Xenarthra and either Afrotheria or Boreoeutheria. The cochlea of *Dasypus* coils to a greater degree than the ancestor of Placentalia (816° versus 738°), and the cochlea contributes a larger percentage of the total inner ear volume than the placental ancestor (66% versus 58%).

#### Boreoeutheria

The non-afrotherian and non-xenarthran placentals, or Boreoeutheria, are divided into two sister clades, the Euarchontoglires and Laurasiatheria (Figure 2). The lateral semicircular canal of the ancestral boreoeutherian entered the vestibule directly without forming a secondary common crus with the posterior semicircular canal (a state inherited from the placental ancestor), and the arc of the anterior semicircular canal was the largest among the three arcs, which is a state retained from the therian ancestor. The plane of the lateral semicircular canal was positioned high compared to the ampullar opening of the posterior semicircular canal, which is derived from the ancestors of both Theria and Eutheria, although the state in the placental ancestor is unknown. A high position of the lateral canal in Boreoeutheria is shared with *Dasypus*, which might support a sistergroup relationship between Xenarthra and Boreoeutheria, but the ancestral state in Afrotheria could not be reconstructed unequivocally. Owing to variation of the aspect ratio of the cochlear spiral within Laurasiatheria and Euarchontoglires, the condition for the ancestor of Boreoeutheria is equivocal between the high and low conditions.

The degree of coiling of the ancestor of Boreoeutheria (815°) is almost identical to that of *Dasypus* (816°), both of which are greater than that reconstructed for Afrotheria (751°). Such a degree of coiling might support a Xenarthra plus Boreoeutheria pairing. However, the volumetric contribution of the cochlea to the entire labyrinth of Boreoeutheria (55%) nearly is identical that reconstructed for Afrotheria (56%), both of which are less than that calculated for *Dasypus* (66%).

#### Laurasiatheria

Laurasiatheria encompasses a great diversity of placental mammals in terms of body size, ranging from the smallest extant mammal – the hog-nosed bat (*Craseonycteris thonglongyai*) – at around 2 g to the largest – the blue whale (*Balaenoptera musculus*) – at around 150000 kg [89]. Some laurasiatherians are specialized for efficient cursoriality, such as the cheetah (*Acinonyx jubatus*) or Thomson’s gazelle (*Eudorcas thomsoni*), while others are adapted for fossorial lifestyles, such as the European mole (*Talpa europaea*). Furthermore, volant bats and fully aquatic cetaceans are included within Laurasiatheria. As a whole, the clade Laurasiatheria is composed of Cetartiodactyla (represented here by *Sus scrofa*, the extinct *Bathygenys reevesi*, *Tursiops truncatus*, and an extinct member of Balaenopteridae), Perissodactyla (represented by *Equus caballus*), Carnivora (represented by *Canis familiaris*, *Eumetopias jubatus*, and *Felis catus*), Pholidota (represented by *Manis tricuspis*), Chiroptera (represented by *Pteropus lylei*, *Nycteris grandis*, *Rhinolophus ferrumequinum*, and *Tadarida brasiliensis*), and Eulipotyphla (represented by *Atelerix albiventris* and *Sorex monticolus*). General dimensions of the bony labyrinths of laurasiatheres are provided in Table 1, dimensions of the cochlea are provided in Table 2, and dimensions and orientations of the semicircular canals are reported in Tables 3–5.

Most character states reconstructed for the ancestral laurasiatherian were retained from its boreoeutherian ancestor. That is, the lateral semicircular canal entered directly into the vestibule without forming a secondary common crus, the arc of the anterior semicircular canal has the greatest radius, and the plane of the lateral semicircular canal was high compared to the junction of the posterior canal and its ampulla. The state of the aspect ratio of the cochlea was reconstructed as equivocal, as was calculated for Boreoeutheria. The ancestral degree of coiling of the cochlea of Laurasiatheria (751.0°) was less than that reconstructed for Boreoeutheria (815°), but the contribution of the cochlea to the entire labyrinthine volume of Laurasiatheria (55%) was similar to that of the boreoeutherian ancestor (56%).

Within Boreoeutheria, Chiroptera is included in a polytomy with Ferae (Carnivora and Pholidota) and a clade comprising Cetartiodactyla and Perissodactyla (Figure 2). The ancestral states reconstructed for the bony labyrinth of the most recent common ancestor of this polytomy were the same as those calculated for the ancestor of Laurasiatheria. That is, the lateral semicircular canal entered the vestibule directly in the absence of a secondary common crus, the plane of the lateral canal was high compared to the ampullar entry of the posterior semicircular canal, and the arc of the anterior semicircular canal was the largest among the three canal arcs. The ancestral degree of coiling for the ungulate-feran-chiropteran polytomy was 815°, which was identical to that of the ancestral boreoeutherian condition (815°), and the cochlea contributed 56% of the total labyrinthine volume, which also was inherited from the ancestor of Boreoeutheria (56%).

Early systematic analyses of mammals based on morphology group cetartiodactyls (although separated into monophyletic Artiodactyla with the exclusion of Cetacea) and perissodactyls in a group called Ungulata along with Sirenia, Hyracoidea, and Proboscidea (and often Tubulidentata) [33], [104]–[105], [107]. Ungulate monophyly has been brought into question by most recent molecular results that not only separate hyracoids, sirenians, proboscideans, and tubulidentates from the perissodactyls, artiodactyls, and cetaceans into Afrotheria (as discussed above), but some recover a close relationship between the cetartiodactyls and perissodactyls with a Carnivora+Pholidota clade [112], [141], and at times placing Perissodactyla as the sister taxon to either Carnivora [145] or the Carnivora+Pholidota grouping [98], [108]–[109]. However, most molecular analyses recover a Perissodactyla+Cetartiodactyla clade, whether Cetacea falls within Artiodactyla or not [111], [140].

The only state reconstructed for the ungulate ancestor that differs from that of the ancestor of Boreoeutheria was the aspect ratio of the cochlea, which was low in the ancestor of the Perissodactyla+Cetartiodactyla clade. The shape of the boreoeutherian cochlear spiral was reconstructed as equivocal, although the aspect ratio of the cochlea was low in the ancestral therian. The bony labyrinth of the ancestor of the Perissodactyla+Cetartiodactyla clade had a lateral semicircular canal that opened into the vestibule directly (retained from the placental ancestor), a position of the plane of the lateral canal high compared to the posterior canal (retained from the boreoeutherian ancestor), and an anterior semicircular canal arc as the largest of the three arcs (retained from the therian ancestor). The ancestral coiling of the cochlea of the Perissodactyla+Cetartiodactyla clade was 857°, which was greater than that reconstructed for the ancestor of the ungulate-feran-chiropteran polytomy (815°), and the ancestral ungulate cochlea contributed 55% of the total labyrinthine volume, which was a value retained from the boreoeutherian ancestor.

The ancestor of Ferae (Carnivora plus Pholidota as supported by the results of numerous analyses [66], [68], [98], [112]) retained labyrinthine morphology similar to the most recent common ancestor of the Perissodactyla+Cetartiodactyla clade, Ferae, and Chiroptera. The lateral semicircular canal entered the vestibule directly in absence of a secondary common crus, the anterior semicircular canal arc was the largest among the three arcs, and the lateral canal was positioned high compared to the ampullar opening of the posterior semicircular canal. The aspect ratio of the cochlea was equivocal, but the ancestral feran cochlea coiled 888° and contributed 56% of the total labyrinthine volume. The volumetric contribution of the cochlea of Ferae was retained from the boreoeutherian ancestor, but the degree of coiling was greater than that reconstructed for its ancestors within Boreoeutheria.

There are no unambiguous otic synapomorphies that support any relationships between the Perissodactyla+Cetartiodactyla clade, Ferae, and Chiroptera. Ancestral states reconstructed for the ancestors of clades within Cetartiodactyla, Perissodactyla, Ferae, and Chiroptera, as well as the ancestral states for Chiroptera as a whole, are provided in separate sections below.

#### Terrestrial cetartiodactyla

The origins of Cetacea have been mired in controversy (see the brief historical review of cetacean systematics by Gingerich [146]), but most evidence supports a close relationship between cetaceans and even-toed ungulates (traditionally classified as Artiodactyla). Both morphology [2], [67], [107], [147]–[154] and molecules [66], [98], [111]–[112], [155]–[160] have been used to suggest a common origin for cetaceans and their terrestrial hoofed relatives. Although a large amount of the genetic data support a nesting of Cetacea within Artiodactyla, only recently have such relationships found support from morphology [161]–[163]. Because the name Cetartiodactyla is commonly used in scientific literature, including those studies that include Cetacea within Artiodactyla, the name Cetartiodactyla is used throughout the remainder of the present paper.

The terrestrial members of Cetartiodactyla (non-cetacean even-toed ungulates) are divided into the three major extant groups, which are Suiformes (pigs and hippos), Tylopoda (camels and llamas), and Ruminantia (deer and cows) [164]. The extinct oreodont *Bathygenys reevesi* and its closest relative in the sample, the extant pig *Sus scrofa*, represent the terrestrial cetartiodactyls here.

**Figure 24:**
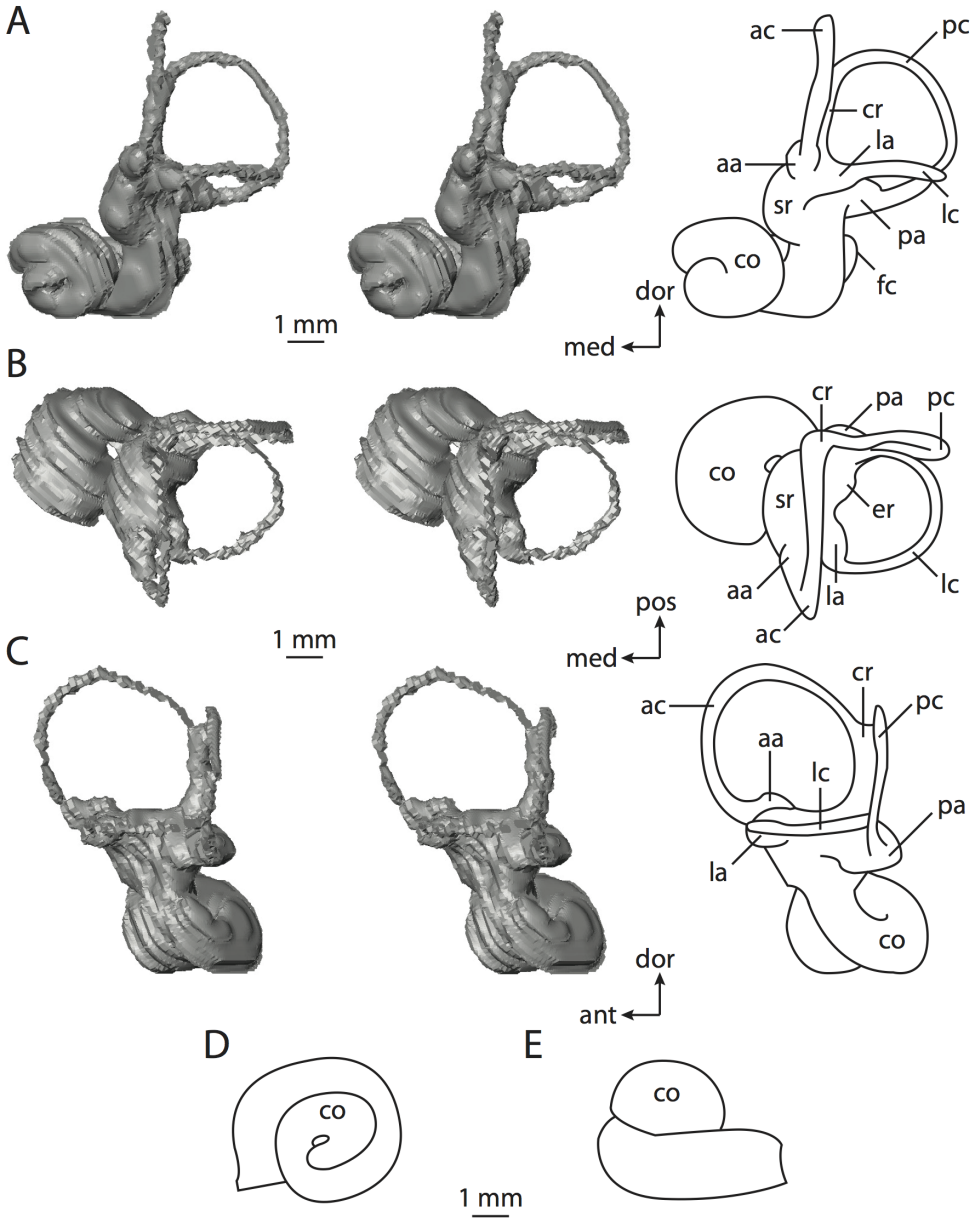
Bony labyrinth of*Bathygenys reevesi*. **A**, stereopair and labeled line drawing of digital endocast in anterior view; **B**, stereopair and labeled line drawing of digital endocast in dorsal view; **C**, stereopair and labeled line drawing of digital endocast in lateral view; **D**, line drawing of cochlea viewed down axis of rotation to display degree of coiling; **E**, line drawing of cochlea in profile. Abbreviations listed at the end of the Materials and Methods section.

**Figure 25:**
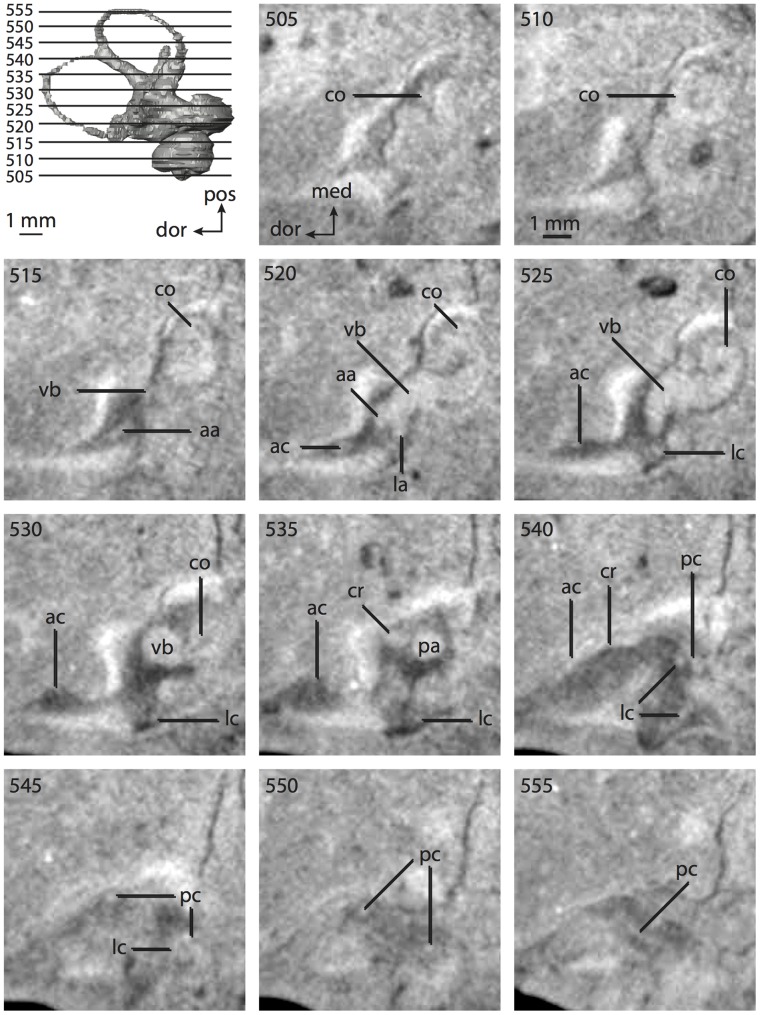
CT slices through ear region of*Bathygenys reevesi*. Abbreviations listed at the end of the Materials and Methods section.

**Figure 26:**
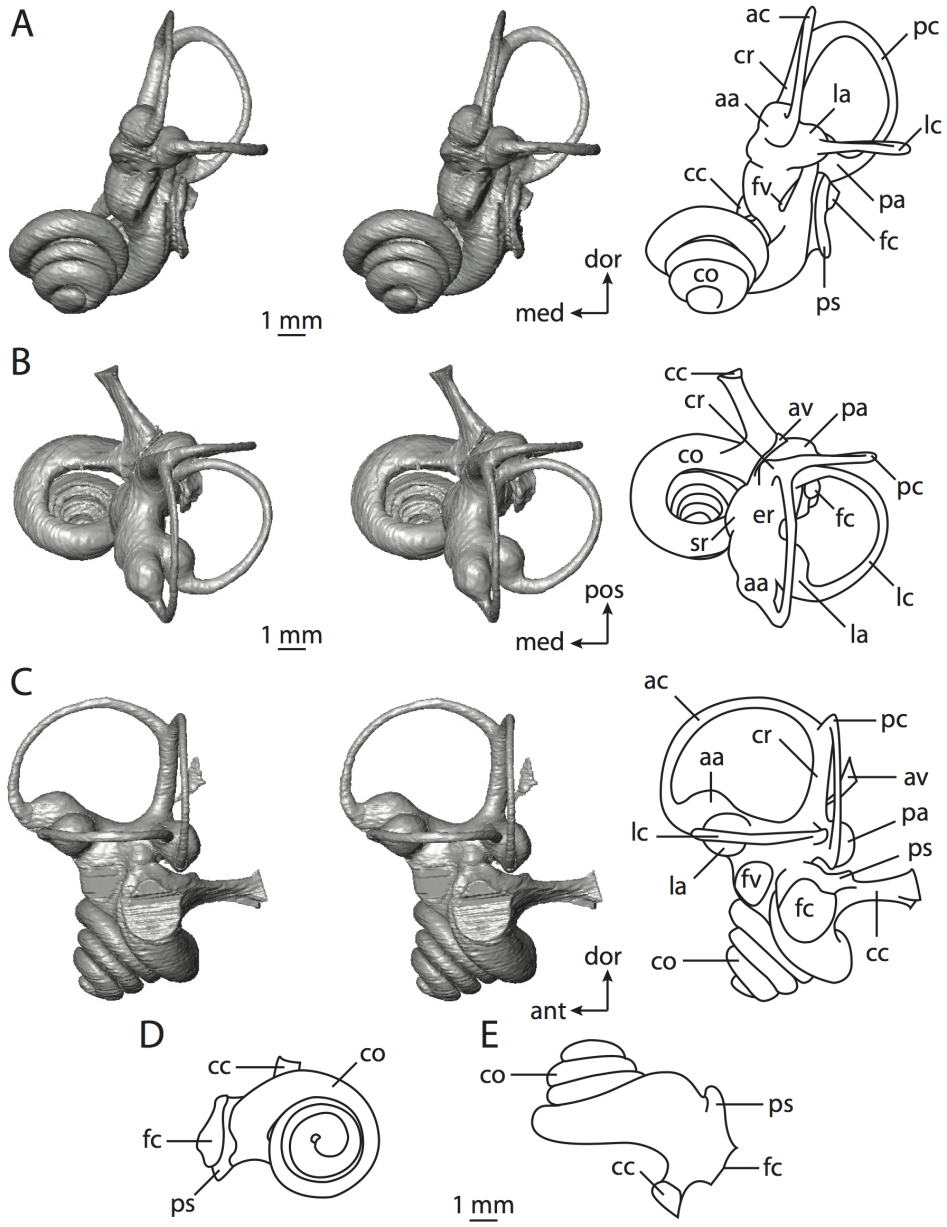
Bony labyrinth of*Sus scrofa*. **A**, stereopair and labeled line drawing of digital endocast in anterior view; **B**, stereopair and labeled line drawing of digital endocast in dorsal view; **C**, stereopair and labeled line drawing of digital endocast in lateral view; **D**, line drawing of cochlea viewed down axis of rotation to display degree of coiling; **E**, line drawing of cochlea in profile. Abbreviations listed at the end of the Materials and Methods section.

**Figure 27:**
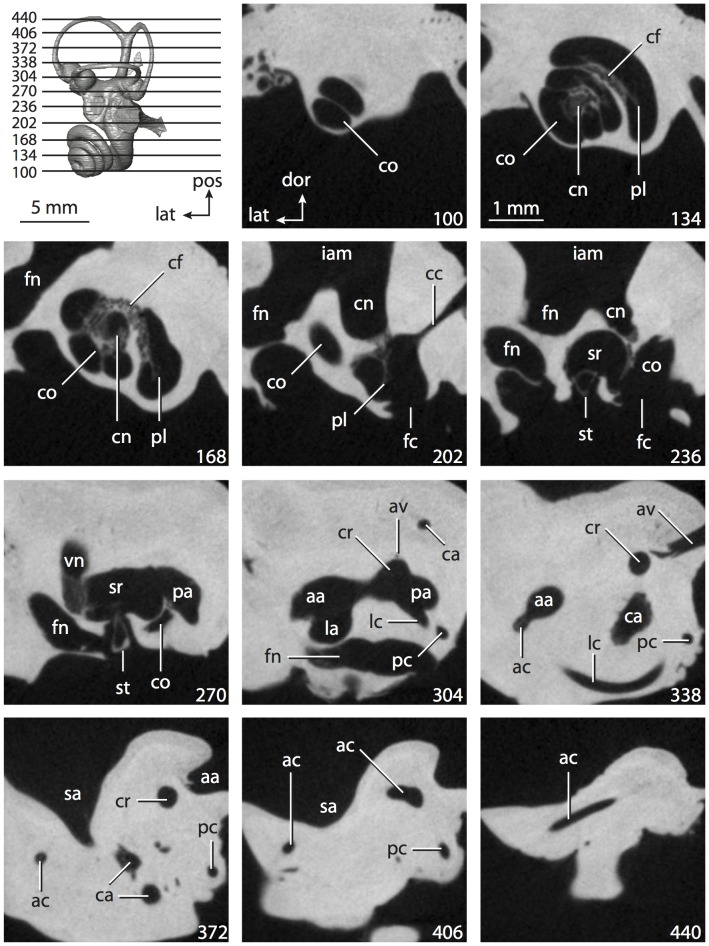
CT slices through ear region of*Sus scrofa*. Abbreviations listed at the end of the Materials and Methods section.

Oreodonts (classified under Tylopoda [164]) were common members of the North American mammal biota during the Tertiary [165]. *Bathygenys reevesi* (Figures 24 and 25) is a small oreodont from the Airstrip and Little Egypt local faunas in the Trans-Pecos region of Texas [166], and inclusion of the ear region of this taxon extends the temporal range of the placental sample into the Oligocene [167]. Preservation of the ear region in the skull of *Bathygenys* was such that the matrix filling the inner ear cavities appears very similar to the bone in the CT scans (Figure 25). Because of this, small structures, such as the bony channels for the cochlear and vestibular aqueducts are not visible in the digital images. Furthermore, the boundaries of the fenestrae cochleae and vestibuli are ill defined, and measurements, such as the stapedial ratio calculated from the dimensions of the fenestra vestibuli, were not taken. However, the gross anatomy of the bony labyrinth of *Bathygenys* was segmented (Figures 24) and described here along with *Sus scrofa* (Figures 26–27).

The bony labyrinth of *Sus* is larger than the labyrinth of *Bathygenys*, both in terms of the length and gross volume of the inner ear cavities (Table 1). Overall, *Sus* is a larger animal than *Bathygenys*, given that the length of the skull of *Sus* is over two and a half times greater than that measured for *Bathygenys* (Table 1). The body mass of *Bathygenys* was not estimated, but the average body mass of *Sus* is 88 kg [89].

Although the cochlea of *Sus* is more voluminous than the cochlea of *Bathygenys* (Table 2), the structure contributes a similar amount to the total volume of the bony labyrinth in both species (54% in *Bathygenys*; 59% in *Sus*). However, this is the only similarity between the cochleae of the two taxa. The cochlear canal is significantly longer (Table 2) and coils to a much greater degree in *Sus* (Figure 26D) than is observed in *Bathygenys* (Figure 24D). Furthermore, the cochlear spiral of *Sus* has a much higher aspect ratio than the cochlea of *Bathygenys* (Table 2; Figure 24E and 26E). The aspect ratio of the cochlea of *Bathygenys* is similar the ratio calculated for *Hemicentetes semispinosum*. The apical whorl of the cochlea of *Bathygenys* sits upon the basal turn, although the diameter of the apical whorl is smaller than the basal, as can be seen when the cochlea is in vestibular view (Figure 24E).

The shape of the cochleae of *Sus* (Figure 26) and *Procavia* (Figure 16) are strikingly similar. The aspect ratios calculated for the cochlear spirals are identical between the two taxa, and both spirals form a pyramid-like structure, where the apical whorls do not sit directly upon the basal whorl, but rather fit within the basal turn when the cochlea is in vestibular view. The diameters of the second and third turns are similar, so that the third whorl shields most of the second from view when the cochlea is viewed down the axis of rotation.

Preservation of the bony labyrinth of *Bathygenys* was such that presence or absence of a secondary bony lamina within the cochlea could not be determined (Figure 25). However, the CT scans through the ear region of *Sus* demonstrate that the secondary lamina was not present in the cochlea of the pig (Figure 27). The scala tympani of *Sus* is expanded posterodorsal to the fenestra cochleae, and a very robust bony canaliculus cochleae that is triangular in cross-section and projects posteromedially from the excavation (cc in Figure 26A and C). As stated above, the canaliculus cochleae was not observed for *Bathygenys*, owing to preservation of the fossil specimen. The canaliculus cochleae likely was present in the taxon, given that the structure is observed in all other cetartiodactyls examined here and elsewhere [30], as well as most placental taxa.

The angles formed between the planes of the basal turn of the cochlea and the lateral semicircular canal are not much different between *Bathygenys* and *Sus* (Table 2). The degree of angular deviation observed in these taxa is similar to that measured in the elephant shrew, *Macroscelides proboscideus*. The stapedial ratio could not be measured for *Bathygenys*, but a low ratio was calculated for *Sus* (Table 3). The spherical recess of the vestibule, through which the labyrinth communicates with the middle ear cavity via the fenestra vestibuli and stapes, is well defined in *Sus* (sr in Figure 26B; Figure 27, slices 236 and 270). The recess in the pig forms a sphere that is bisected, and the fenestra vestibuli is situated on the cut surface. The spherical recess cannot be distinguished easily from the elliptical recess in *Bathygenys*, where the vestibule is developed as a continuous yet irregularly shaped cavity (er in Figure 24B; Figure 25, slices 520 through 530).

The elliptical recess of the vestibule of *Sus* is elongate, with a stout anterior projection that is expressed as a pedestal for the anterior and lateral ampullae on the digital endocast (er in Figure 26B and C). A similar projection is observed in the vestibule of *Bathygenys* (Figure 24B and C). All three ampullae in *Sus* are rounded, teardrop-shaped excavations within the vestibule. The posterior limb of the lateral semicircular canal takes a straight course as it opens directly into the vestibule dorsal to the posterior ampulla in both cetartiodactyls. Because of this, the lateral canal divides the space enclosed by the arc of the posterior semicircular canal when either labyrinth is viewed anteriorly (lc in Figures 24A and 26A). The sagittal labyrinthine index is nearly the same for *Bathygenys* as it is for *Sus* (Table 3), and the arc of the lateral semicircular canal does not extend posterior to that of the posterior canal in either taxon. The lateral extent of both canals is equivalent.

The bony channel for the vestibular aqueduct is not observed in *Bathygenys,* but the channel is a thin canal in *Sus* that exits the vestibule ventromedial and slightly anterior to the vestibular aperture of the common crus (av in Figure 26B). The channel for the vestibular aqueduct is expressed on the endocast as a fine thread before expanding as the aqueduct opens into a fissure near the endocranial surface of the petrosal. Although the channel for the vestibular aqueduct is more delicate than the canaliculus cochleae for the cochlear aqueduct, the bony channel for the vestibular aqueduct is slightly longer.

Although the specific measured values of the angles between the planes of the three semicircular canals are different for *Sus* and *Bathygenys*, the basic pattern is the same for both of these species. For example, the angle between the planes of the anterior and posterior semicircular canals is the greatest for both *Bathygenys* and *Sus*, and the angle between the anterior and lateral canals is the smallest (Table 3). The angles between the planes of the posterior and lateral semicircular canals are the closest to 90° for both taxa.

Both the radius and diameter of the lumen of the anterior semicircular canal arc is the largest in both *Sus* and *Bathygenys* (Table 4). However, this pattern is not observed universally across all dimensions of the semicircular canals. For example, the length of the slender portion of the anterior semicircular canal is the greatest in *Sus*, but the posterior canal is the longest in *Bathygenys*. The aspect ratio of the lateral semicircular canal is the greatest in both *Bathygenys* and *Sus*, although the ratio of the anterior canal was the smallest in *Bathygenys*, whereas the aspect ratio of the posterior canal was the smallest in *Sus* (Table 5). The ratio between the length of the slender portion of the canal and arc radius for the posterior semicircular canal was the greatest for both *Bathygenys* (5.59; anterior equals 5.08; lateral equals 4.68) and *Sus* (4.89; anterior equals 4.86; lateral equals 3.87).

The semicircular canals of *Sus* are more planar (fit better onto a single plane) than the canals of *Bathygenys*. In fact, the anterior semicircular canal is perfectly planar in *Sus*, whereas the anterior canal deviates from its plane in *Bathygenys* (Table 5). The posterior canal is the least planar of the three for both taxa. The posterior canal is curved posteriorly in *Bathygenys* (pc in Figure 24C), although the curvature might be an artifact of preservation. The posterior canal is straight along its course in *Sus* (pc in Figure 26C). Only the posterior semicircular canal deviation of *Bathygenys* is considered substantial (ratio of total linear deviation over cross-sectional diameter is 1.23 versus 0.61 for anterior and 0.64 for lateral; ratios for anterior, lateral, and posterior canals of *Sus* are 0.00, 0.20, and 0.24 respectively).

The bony labyrinth of the ancestor of Cetartiodactyla was similar to that reconstructed for the ancestor of the Perissodactyla+Cetartiodactyla clade. The lateral semicircular canal opened into the vestibule directly in absence of a secondary common crus, the arc of the anterior semicircular canal was the largest among the three, the lateral semicircular canal was positioned high compared to the posterior canal, and the aspect ratio of the cochlea was low. The cochlea of Cetartiodactyla coiled to a lesser degree than the Perissodactyla+Cetartiodactyla clade (846° versus 857°), but the cochlear canal contributed a greater percentage to the overall labyrinthine volume (59% versus 55%).

The labyrinths of the two terrestrial cetartiodactyls retain the ancestral cetartiodactyl condition of the anterior canal possessing the largest arc radius. The cochlea of *Bathygenys* is flattened (low aspect ratio), which is the ancestral condition, although the cochlea of *Sus* has a high profile. Both labyrinths retain the ancestral cetartiodactyl condition of the high position of the lateral semicircular canal as compared to the posterior canal, and a vestibular entrance of the lateral canal, rather than formation of a secondary common crus.

Although the cladogram presented in Figure 2 depicts a closer relationship between *Sus* and Cetacea, there are no unambiguous inner ear synapomorphies supporting this relationship. Both *Sus* and *Bathygenys* share a high position of the lateral semicircular canal that is absent in Cetaceans (discussed in the following section), but this state was ancestral for crown Placentalia as a whole. Nonetheless, the most recent common ancestor of *Sus* and Cetacea possessed a bony labyrinth with the lateral semicircular canal opening directly into the vestibule, the anterior semicircular canal arc with the largest radius among the three canals, a high position of the lateral semicircular canal compared to the posterior canal, and a low aspect ratio of the cochlea in profile. The ancestral cochlear coiling of *Sus* and Cetacea was 1013°, which appears to be a factor of the high degree of coiling in *Sus* given that the cochleae of the cetaceans do not coil nearly as much (see below). However, the ruminants and tylopods studied by Gray [30] possess cochleae that complete between two and one half and two and three quarter turns. The contribution of the cochlea to the entire labyrinth is 67% for the ancestor of *Sus* and Cetartiodactyla, which likely is an inflated estimation given the disproportionately large cochlea of the cetaceans.

#### Cetacea

With the exception of Sirenia (the bony labyrinth of which was described above in the Afrotheria section), cetaceans are the only fully aquatic extant mammals. Two major cetacean clades recognized are the baleen whales, or Mysticeti, which includes the largest living mammal (*Balaenoptera musculus*), and the toothed whales, Odontoceti, which includes porpoises and dolphins such as *Tursiops truncatus*. The bony labyrinth of the bottlenose dolphin *Tursiops* is described, along with the labyrinth of a fossil member of Balaenopteridae (Mysticeti). The balaenopterid fossil used (TMM 42958-35) was collected from Pliocene deposits of the Yorktown Formation at the Lee Creek Mine in Aurora County, North Carolina. Morphologically, the isolated petrosal can be identified as Balaenopteridae following the key and descriptions of extant mysticetes by Ekdale and others [7]. A more specific identification could not be made. The general structure of the bony labyrinth of the fossil does not depart substantially from that described for other extinct [1], [9] and extant [31], [48], [50], [168] mysticetes.

**Figure 28:**
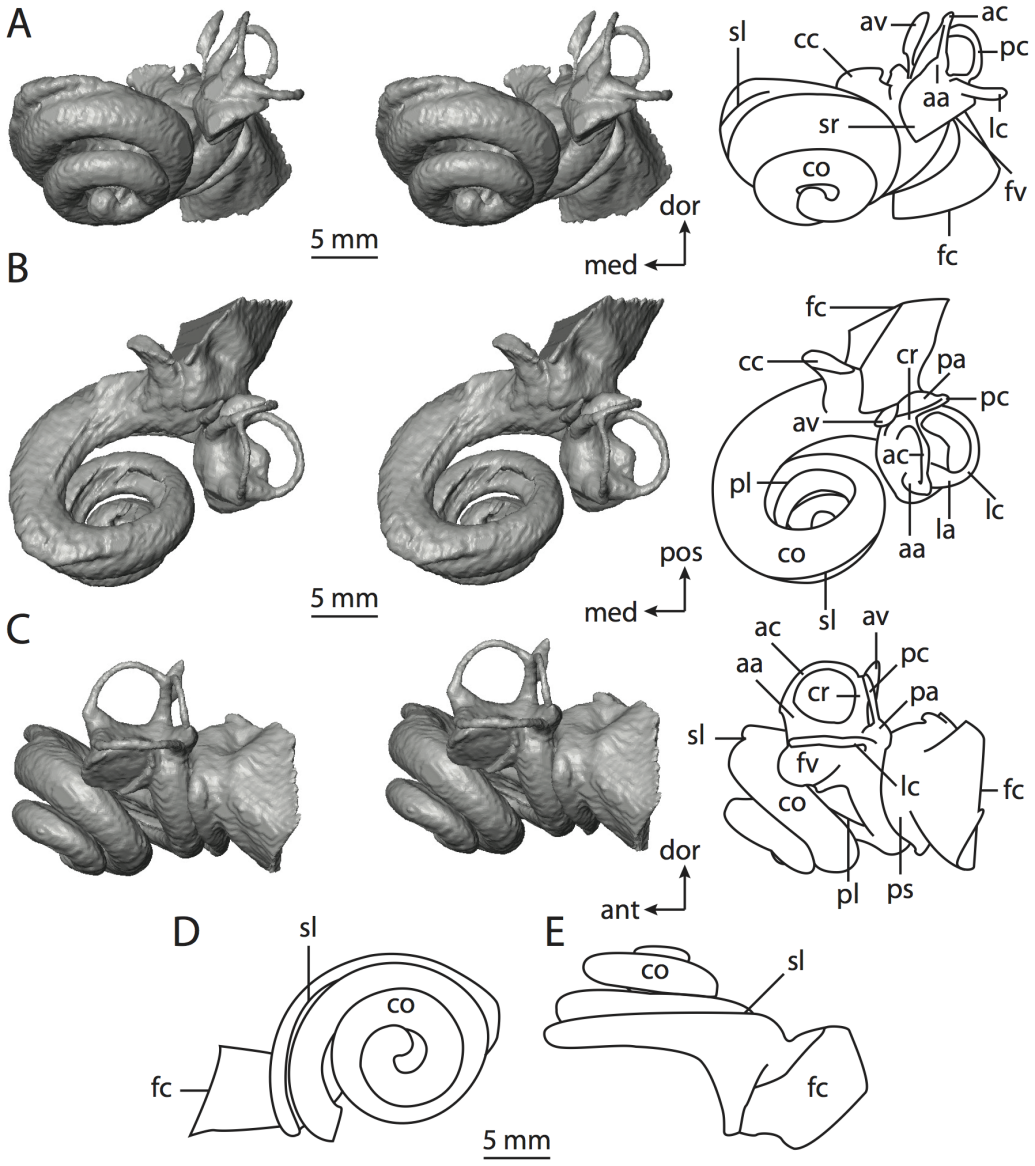
Bony labyrinth of fossil Balaenopteridae. **A**, stereopair and labeled line drawing of digital endocast in anterior view; **B**, stereopair and labeled line drawing of digital endocast in dorsal view; **C**, stereopair and labeled line drawing of digital endocast in lateral view; **D**, line drawing of cochlea viewed down axis of rotation to display degree of coiling; **E**, line drawing of cochlea in profile. Abbreviations listed at the end of the Materials and Methods section.

**Figure 29:**
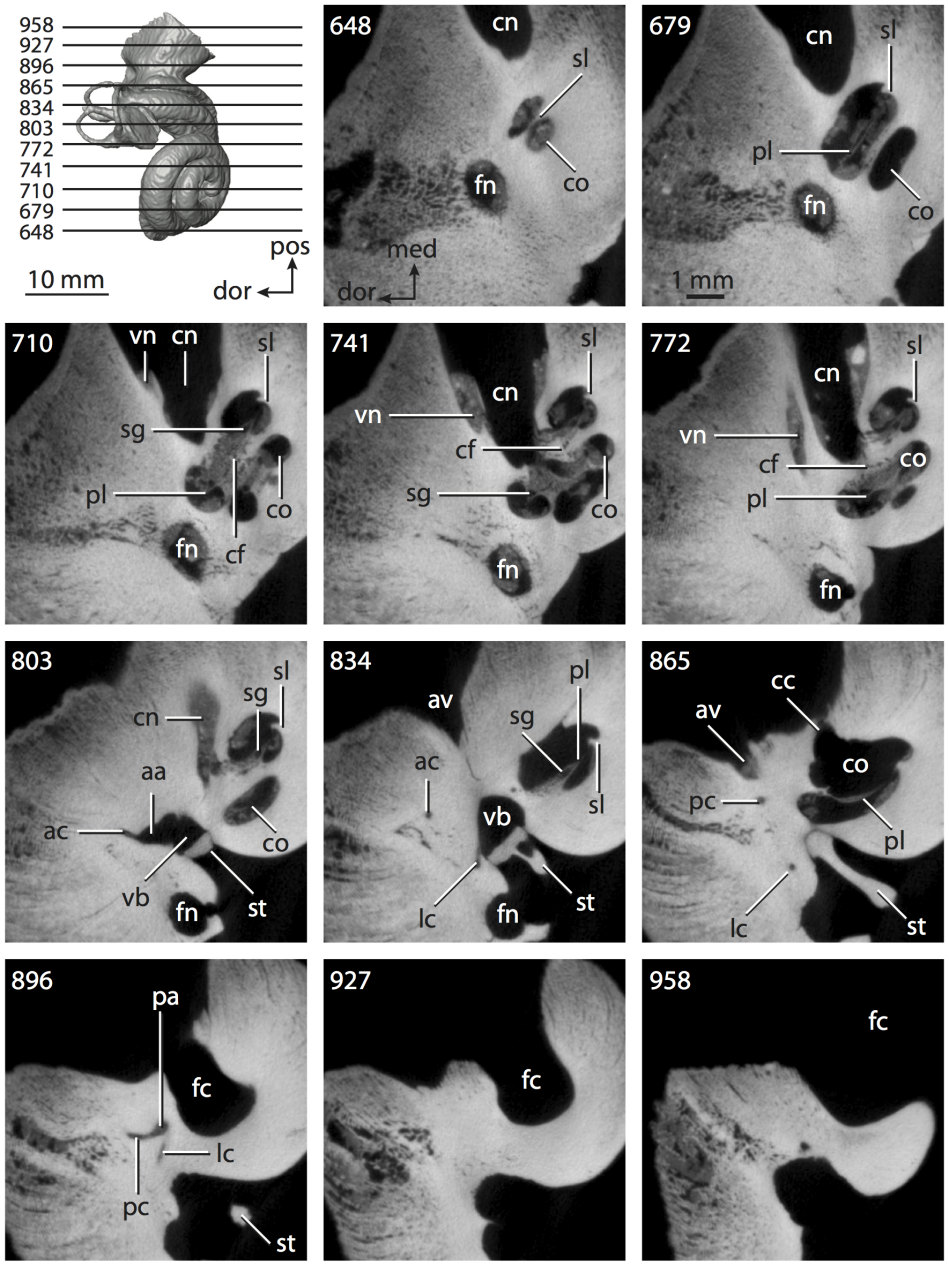
CT slices through ear region of fossil Balaenopteridae. Abbreviations listed at the end of the Materials and Methods section.

**Figure 30:**
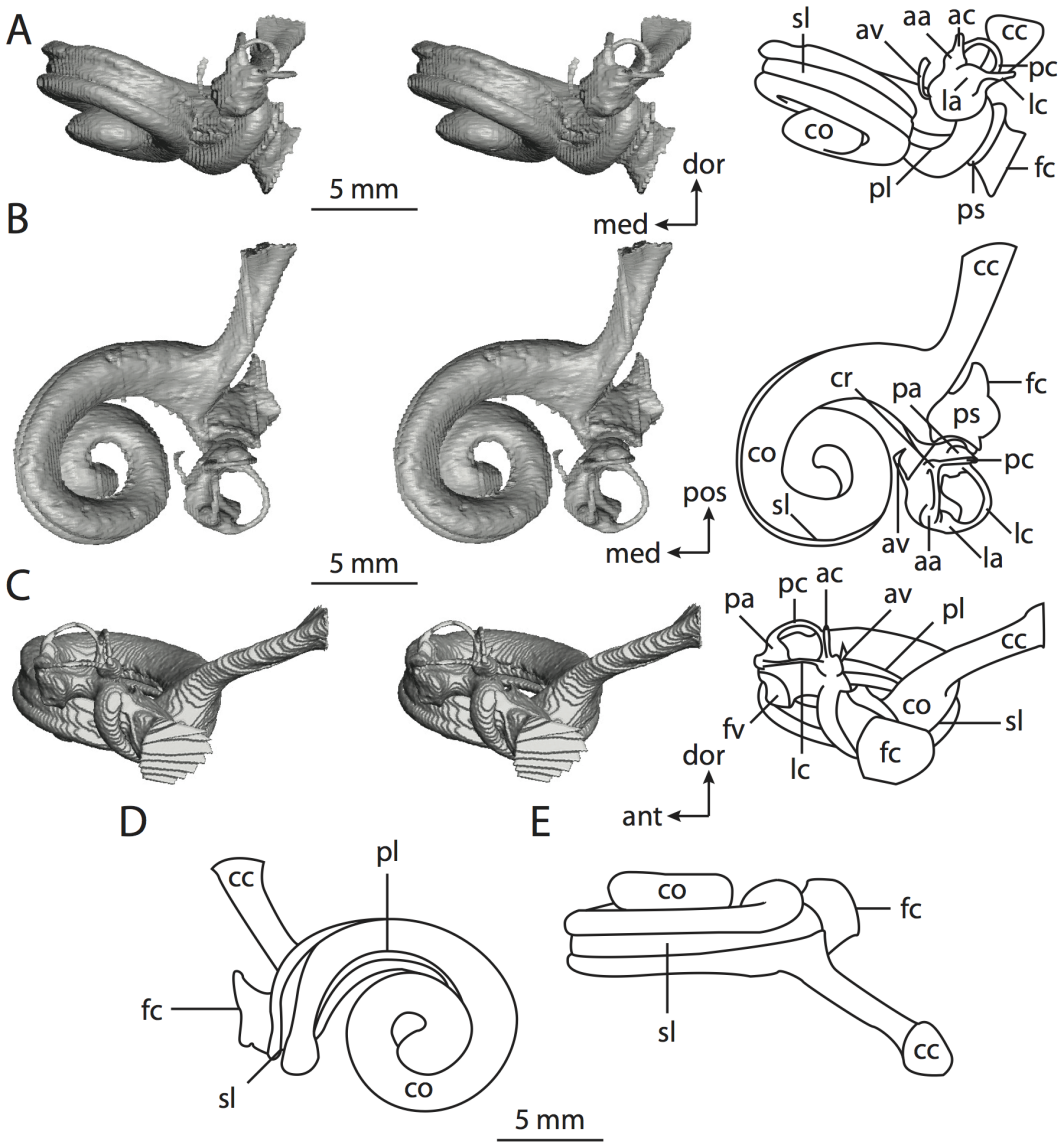
Bony labyrinth of*Tursiops truncatus* (images reversed). **A**, stereopair and labeled line drawing of digital endocast in anterior view; **B**, stereopair and labeled line drawing of digital endocast in dorsal view; **C**, stereopair and labeled line drawing of digital endocast in lateral view; **D**, line drawing of cochlea viewed down axis of rotation to display degree of coiling; **E**, line drawing of cochlea in profile. Abbreviations listed at the end of the Materials and Methods section.

**Figure 31:**
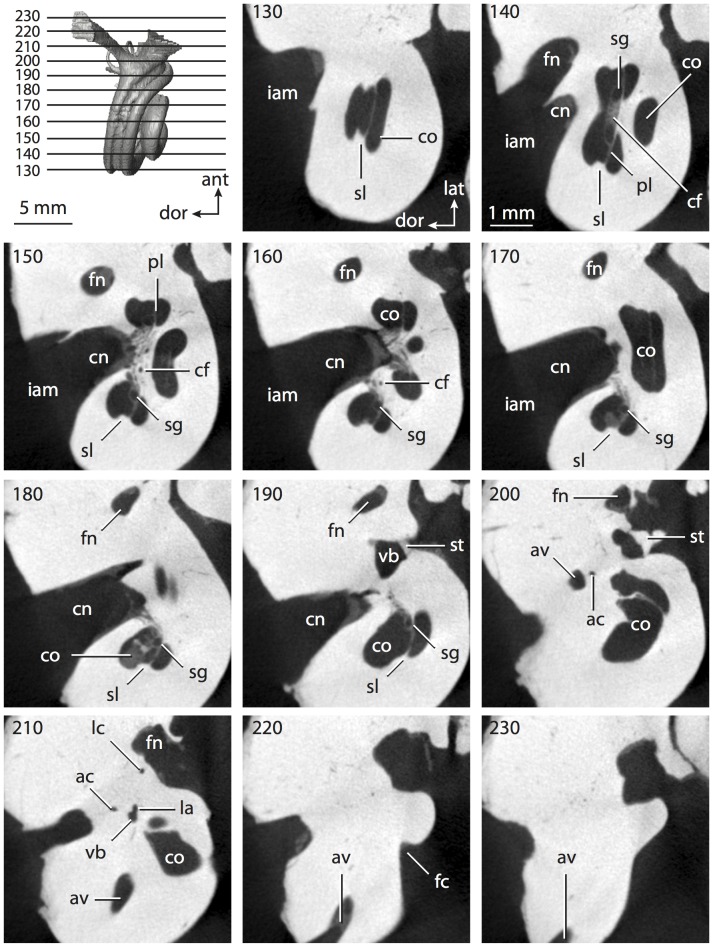
CT slices through ear region of*Tursiops truncatus*. Abbreviations listed at the end of the Materials and Methods section.

The bony labyrinth of the extinct balaenopterid (Figures 28–29) is larger than that of *Tursiops* (Figures 30–31) both in terms of anterior-posterior length and gross volume of the inner ear cavities (Table 1). The greater dimensions of the mysticete labyrinth likely reflects body size differences between balaenopterids and *Tursiops*, with the average body mass of most mysticete species being several orders of magnitude greater than that of the bottlenose dolphin [89]. Average body mass for *Tursiops* is 179.5 kg, whereas the smallest extant balaenopterid is 4,000 kg (*Balaenoptera acutorostrata*) [89].

The cochlea of the balaenopterid is larger than *Tursiops* in all dimensions, including volume, cochlear canal length, degree of coiling, and even aspect ratio, although to a lesser extent (Table 2). The volumetric contribution of the cochlea to the total inner ear volume is greater for *Tursiops* (94%) than for the balaenopterid (91%), although the value for the balaenopterid is exceptionally high. The significant contribution of the total volume by the cochlea is higher for the two cetacean taxa than any other mammal investigated here, including the afrotherians *Chrysochloris, Macroscelides*, and *Trichechus* (each with a cochlear contribution around 71–72%). The other extreme is the cochlea of the elephantimorph proboscidean, which only contributes 31% of the total volume of the bony labyrinth (see above).

As is evident with the aspect ratios reported in Table 2, the cochleae of both cetaceans are low-spired, and the apical turns of the cochlea are smaller in diameter than the basal whorl (Figures 28D and 30D). A well-developed secondary bony lamina is present in both labyrinths (sl in Figures 28 through 31), extending from a point anterior to the fenestra cochleae for a considerable distance along the radial wall of the cochlear canal. The secondary lamina, which is expressed as a distinct groove on the endocast, is present for around two thirds of the basal turn in the balaenopterid, but it persists for a short distance beyond the basal turn in *Tursiops* (Table 2).

An anteriorly oriented excavation of the cochlea (expressed as a flange on the endocast) forms a tympanal recess immediately basal to the apical terminus of the secondary bony lamina in the balaenopterid (Figure 28C). A similar structure is observed in the reconstruction of the inner ear of the extinct mysticete *Herpetocetus* [1], and it might be characteristic of the Mysticeti, although such a recess is not observed in the cast of the inner ear created by Hyrtl [48], nor is the extension observed in *Tursiops* or any other mammal studied here.

The bony canaliculus for the cochlear aqueduct is significantly longer in *Tursiops* than in the balaenopterid (Table 2). The canaliculus of *Tursiops* is roughly triangular in cross-section (cc in Figure 30E), whereas the bony passage is flattened in the balaenopterid (cc in Figure 28B). The scala tympani is not inflated near the proximal end of the canaliculus cochleae posterior to the fenestra cochleae in *Tursiops*, but a groove (expressed as a ridge on the endocast) extends from the canaliculus cochleae for a short distance on the tympanal surface of the cochlea in the balaenopterid. The angle between the planes of the basal turn of the cochlea and lateral semicircular canal for the balaenopterid and *Tursiops* are similar to the angles measured for the terrestrial cetartiodactyls *Bathygenys* and *Sus* (see above; Table 2).

The fenestra vestibuli is separated from the fenestra cochleae by a great distance in both cetacean taxa. This likely is the result of a flexure near the junction of the cochlea and vestibule. A cochlear hook ending in the fenestra cochleae is a common feature in the inner ear of cetaceans, primarily odontocetes [9], [32], [169], but the structure is not observed in the extinct mysticete. The stapedial ratios, as calculated from the fenestra vestibuli, are more circular in both cetacean taxa than the ratios calculated for other placental mammals (Table 3).

The entire vestibular apparatus of the cetacean bony labyrinth is significantly smaller than that of other taxa (Figures 28 through 31). This is expressed not only in the images of the endocasts, but also in the volumetric measurements (cochlea of *Tursiops* contributes around 94% of the volume, so vestibular apparatus contributes 6%; vestibular apparatus of the balaenopterid is around 10%). The vestibule of *Tursiops* is bowed medially (Figure 30B) with the fenestra vestibuli opening through an anterior excavation of the cavity (fv in Figure 30C). The vestibule is not curved in the balaenopterid, and the spherical recess is small and is distinguished from the elliptical recess by a gentle constriction behind the fenestra vestibuli (sr in Figure 28A). A similar constriction is observed in *Tursiops* (Figure 30A), but the connection between the anterior aspect of the spherical recess and the cochlea is not distinguishable.

An extension of the elliptical recess adjacent to the constriction between the vestibular compartments leads to the lateral and anterior ampulla (la and aa in Figures 28B and 30B). The posterior limbs of the lateral semicircular canals of both cetaceans do not have separate openings into the vestibule. Rather, the lateral canal empties into the posterior ampulla just above the vestibular aperture of the ampulla (a secondary common crus is not present in either taxon; lc and pa in Figure 28B and C). The posterior limb of the lateral canal takes an undulating course into the vestibule in *Tursiops*, but the limb is straight in the balaenopterid fossil (lc in Figures 28C and 30C). The lateral extents of the posterior and lateral semicircular canal arcs are equivalent in both taxa, and the lateral canal does not extend posterior to the plane of the posterior canal in either cetacean.

The bony channel for the vestibular aqueduct exits the bony labyrinth near the medial edge of the vestibular aperture of the common crus in the balaenopterid. The passage in this taxon is expressed as fine thread that extends to the endocranial aperture of the bony channel (av in Figure 28A). The vestibular aperture for the channel for the vestibular aqueduct of *Tursiops* (av in Figure 30A and B) is separated from the common crus by a relatively greater distance than it is in the balaenopterid (av in Figure 28A and B). In medial view, the massive cochlea shields the channel in *Tursiops*, but is best observed when the bony labyrinth is oriented dorsally (Figure 30B).

The angle between the planes of the anterior and posterior semicircular canals is obtuse in both *Tursiops* and the balaenopterid, but the remaining canal plane angles are acute (Table 3). The lowest angles for each cetacean were measured between the anterior and lateral canals. The radius of the anterior canal arc is the largest in the balaenopterid and the radius of the posterior arc is the smallest in both (Table 4). The radius of curvature of the lateral semicircular canal arc is largest in *Tursiops*. A common pattern is not observed in any of the other dimensions of the semicircular canals. The length of the slender portion of the canal of the anterior semicircular canal of the balaenopterid is the greatest, although the lateral canal is the longest in *Tursiops*. The diameter of the lumen is greatest in the lateral semicircular canal in the balaenopterid, whereas the diameters of the anterior and posterior canals are equal and larger than the value taken for the lateral canal in *Tursiops* (Table 4).

The aspect ratio of the posterior semicircular canal is greatest in both *Tursiops* and the balaenopterid (Table 5). The aspect ratio of the lateral semicircular canal is the smallest in the balaenopterid, and the ratio of the anterior canal is the smallest in *Tursiops*; however, it is not much different than that of the lateral canal. The largest ratio between the length of the slender portion of any canal and arc radius of curvature among the cetaceans examined was calculated for the posterior semicircular canal of *Tursiops*. The ratio calculated for the posterior canal of the balaenopterid was the largest among the three canals in this specimen as well (4.94). Although the ratio calculated for the posterior canal of *Tursiops* was larger than that of the balaenopterid, the ratios for the anterior (4.19) and lateral (4.05) semicircular canals were larger than those calculated for *Tursiops* (3.47 and 3.38 respectively).

Overall, the semicircular canals of *Tursiops* fit onto single planes (the anterior and posterior canals are planar). The only canal of *Tursiops* that deviates from its plane is the lateral semicircular canal (table 5), although deviation is not substantial (ratio of total linear deviation over cross-sectional diameter is 0.85). The angular deviations of the anterior and posterior semicircular canals of the balaenopterid are substantial (ratio of anterior is 1.27; posterior is 1.56), but the lateral semicircular canal of the balaenopterid does not deviate from its plane substantially (ratio is 0.39).

The bony labyrinth of the ancestral cetacean lacked a secondary common crus formed between the lateral and posterior semicircular canal, as did the ancestor of Cetartiodactyla, but the cetacean labyrinth was derived from the ancestral cetartiodactyl condition in that the lateral canal opened into the posterior ampulla, rather than into the vestibule directly. Although this state is a synapomorphy for Cetacea within Cetartiodactyla, the lateral canal also opens into the posterior ampulla in Perissodactyla, Scandentia, some Carnivora, and some Chiroptera (see below). A second otic synapomorphy that separates Cetacea from the terrestrial cetartiodactyls is a low position of the plane of the lateral semicircular canal compared to the ampullar entrance of the posterior semicircular canal. The state is derived with respect to the ancestral cetartiodactyl condition, and it is a reversal to the ancestral therian state.

Additional states reconstructed for the ancestor of Cetacea include the anterior semicircular canal arc as the greatest among the three arcs despite the lateral canal as the largest in *Tursiops*, and a low aspect ratio for the cochlear spiral in profile. The coiling of the cochlea of the ancestral cetacean (853°) was retained from the ungulate ancestor (857°), but the contribution of the ancestral cetacean cochlea to the total labyrinthine volume was greater than that calculated for the Perissodactyla+Cetartiodactyla clade (84% versus 55%). The high contribution of the cochlea to the total volume distinguishes Cetacea from other members of Cetartiodactyla, and likely is greater than that inferred in this study.

#### Perissodactyla

**Figure 32:**
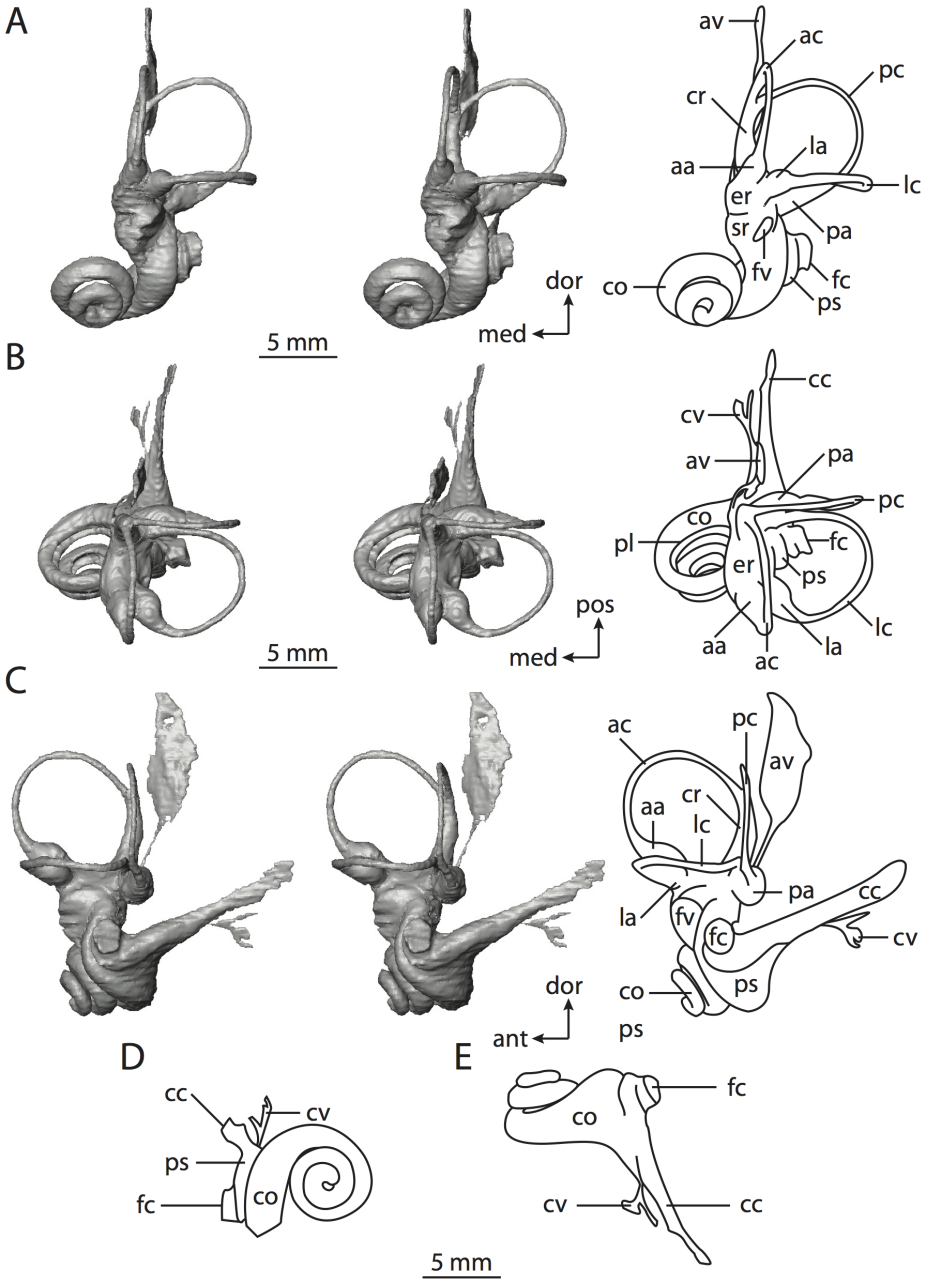
Bony labyrinth of*Equus caballus*. **A** ****, stereopair and labeled line drawing of digital endocast in anterior view; **B**, stereopair and labeled line drawing of digital endocast in dorsal view; **C**, stereopair and labeled line drawing of digital endocast in lateral view; **D**, line drawing of cochlea viewed down axis of rotation to display degree of coiling; **E**, line drawing of cochlea in profile. Abbreviations listed at the end of the Materials and Methods section.

The odd-toed ungulates that make up extant Perissodactyla are divided into Equidae (horses), Tapiridae (tapirs), and Rhinocerotidae (rhinoceroses). Monophyly of Perissodactyla is well supported [66], [99]–[100], [107], [139]–[140], as is a sister taxon relationship between Tapiridae and Rhinocerotidae within the group [61], [70], [84], [170]–[173]. Only the modern horse, *Equus caballus*, was available for examination. Images of the inner ear and an endocast of the bony labyrinth are presented in Figures 32 through 33.

**Figure 33:**
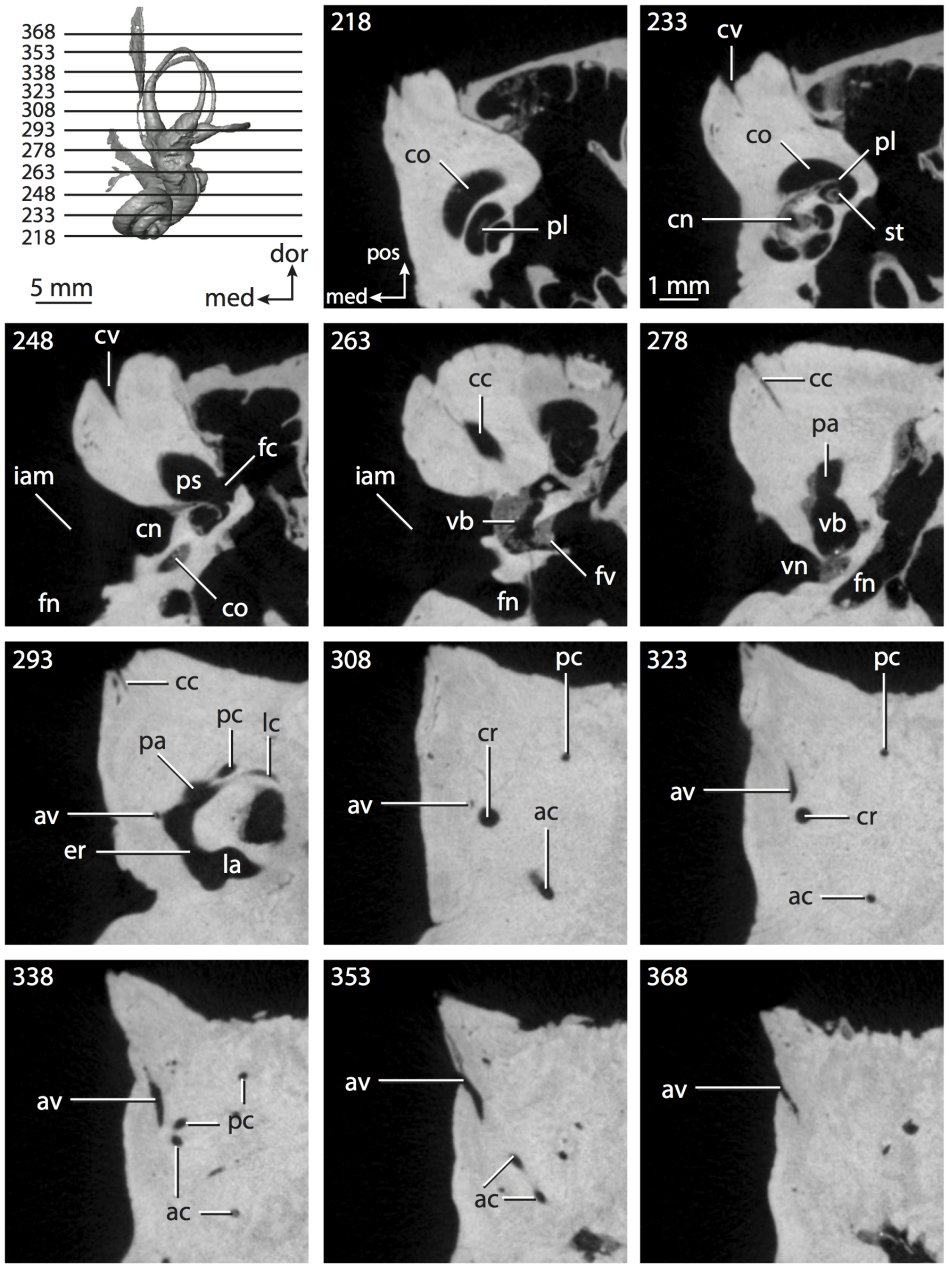
CT slices through ear region of*Equus caballus.* Abbreviations listed at the end of the Materials and Methods section.

The total volume of the inner ear cavities of *Equus* is similar to that of the dolphin *Tursiops truncatus* (Table 1). This is also reflected in the length of the skull, where the skull of *Equus* is slightly shorter than that of *Tursiops*. However, the total length of the inner ear is larger in *Equus* than it is in *Tursiops*, and the horse has a greater average body mass (Table 1; [89]). Although the total volume of the bony labyrinth of *Equus* is similar to that in *Tursiops*, the cochlea of the horse is only half the volume of the cochlea of the dolphin (Table 2). Because of this, the equine cochlea’s total volumetric contribution (51%) is significantly less than that of *Tursiops* (94%), but rather is more in line with the percentage calculated for the terrestrial cetartiodactyl *Bathygenys reevesi* (54%).

The cochlea completes two and a half turns (Table 2), and the spiral is loosely coiled compared to other placentals (Figure 32A and E). The cochlea of *Equus* has a low aspect ratio of (Figure 32E) that is similar to that calculated for the elephantimorph proboscidean and *Orycteropus afer* (Table 2). The total length of the cochlear canal of *Equus* is similar to that measured in the cochlea of *Sus* and *Tursiops*, despite the fact that the volume of the cochlea of the horse falls in between the dolphin and pig (Table 2). A secondary bony lamina, which extends around two fifths of the basal turn (Table 2), is observed in *Equus*, although it is not well-developed. The scala tympani is expanded into a wedge shaped excavation leading to the straight canaliculus cochleae for the cochlear aqueduct posterior to the fenestra cochleae (ps in Figures 32A and 33, slice 248). The canaliculus narrows towards its terminus, and flattens into a fissure near its external aperture (cc in Figure 33, slices 338 through 368), although it retains robusticity along its course. The bony passage is the longest canaliculus measured for any taxon described here (Table 2). A short and delicate secondary passage, likely for a vein, exits the medial side of the canaliculus near the midpoint of the bony channel for the cochlear aqueduct (cv in Figure 32B though E).

The angle between the planes of the basal turn of the cochlea and the lateral semicircular canal in *Equus* is greater than the artiodactyls studied, but intermediate between the carnivorans (see below; Table 2). The spherical and elliptical recesses are separated by a constriction of the vestibule ventral to the ampullae of the semicircular canals (sr and er in Figure 32A and C). The constriction forms a bony ring (expressed as a wide sulcus on the endocast) surrounding the vestibule. The bony ring sits on a plane that nearly is parallel with that of the lateral semicircular canal. The elliptical recess is elongate, and it slightly bows medially, away from the arc of the lateral semicircular canal (er in Figure 32B).

The anterior and lateral ampullae open into a slight excavation at the anterior end of the elliptical recess (aa and la in Figure 32B). At the posterior end of the recess, the undulating posterior limb of the lateral semicircular canal opens into the posterior ampulla, immediately anterolateral to the vestibular aperture of the ampulla. Because of this, the lateral semicircular canal does not have its own opening into the vestibule (also observed in both cetaceans, *Hemicentetes*, and other taxa, including the tree shrew, some carnivorans, and some bats, as described below). Additionally, the plane of the canal is high relative to other vestibular constituents. The elevated lateral semicircular canal divides the space enclosed by the posterior semicircular canal arc when the endocast of the bony labyrinth is viewed posteriorly (lc in Figure 32A), although the sagittal labyrinthine index of *Equus* is the smallest index calculated for any mammal in which the plane of the lateral canal takes a high position described thus far in the paper (Table 3). The lateral extent of the posterior and lateral semicircular canals are equivalent (lc and pc in Figure 32A and B), and the lateral canal does not extend posterior to the posterior semicircular canal plane (lc and pc in Figure 32B and C).

A groove (expressed on the endocast as a low ridge in medial view) extends from the dorsomedial edge of the spherical recess to the vestibular aperture of the bony channel for the vestibular aqueduct, which is situated ventral and medial to the vestibular aperture of the common crus (av in Figure 32B). The channel is developed as a very delicate thread for half of its length in *Equus*, extending posteriorly and crossing the base of the common crus when the endocast of the bony labyrinth is viewed medially. The distal half of the channel is broad and flattened, indicating that the aqueduct enters a fissure before exiting on the endocranial surface of the petrosal (av in Figure 33, slices 308 through 368). The total length of the bony channel for the vestibular aqueduct is a bit larger than the length of the canaliculus cochleae (Tables 2 and 3).

The planes of the posterior and lateral semicircular canals of *Equus* roughly form a right angle with one another, and the angle between the planes of the posterior and anterior canals only is slightly obtuse (Table 3). Both the length of the slender portion and arc radius of the posterior semicircular canal are the greatest among these dimensions measured for the three canals of *Equus* (Table 4). The lateral semicircular canal exhibited the smallest value of these two dimensions. The largest semicircular canal lumen diameter in cross-section was measured for the anterior semicircular canal (Table 4), and the largest volume was measured for the lateral semicircular canal (2.57 mm^3^; anterior equals 2.19 mm^3^; posterior equals 2.32 mm^3^).

The aspect ratios of the anterior and lateral semicircular canal arcs are similar, and both are lower than the aspect ratio of the posterior canal (Table 5). The high aspect ratio of the posterior semicircular canal indicates that the height of the canal arc is greater than the width. The greatest ratio of the length of the slender portion of the canal to arc radius was calculated for the posterior semicircular canal (5.17), and the ratio for the anterior (3.46) and lateral (3.38) semicircular canals are not significantly different. The semicircular canals themselves are fairly planar, especially the anterior canal (Table 5). The degree of deviation is not substantial for any canal, where the ratios of total linear deviation over cross-sectional diameter of the canal for the anterior, lateral, and posterior canals are 0.27, 0.64, and 0.70 respectively. However, the posterior canal is slightly curved anteriorly along its dorsal course, and the lateral canal is sigmoidal (pc in Figure 32C).

The cochlear spiral of *Equus* possesses the ancestral ungulate state of a low aspect ratio, giving the cochlea a flattened appearance. The pattern of semicircular canal arc radii in *Equus*, with the largest radius being the anterior, is inherited from its boreoeutherian ancestor, although the entry of the lateral semicircular canal into the posterior ampulla is derived and shared with Cetacea. The high position of the lateral semicircular canal with respect to the ampullar opening of the posterior canal is retained from the ancestor of Boreoeutheria.

#### Carnivora

Extant carnivorans belong to two phylogenetically distinct clades, Caniformia (dogs, bears, raccoons, weasels, and pinnipeds) and Feliformia (cats, hyenas, mongooses, and viverrids). Monophyly of Pinnipedia (within Caniformia) has been questioned in the past [174]–[175], although most recent data are in support of a single origin for seals, sea lions, and walruses [66], [176]–[178]. Most, if not all, carnivoran classifications include pinnipeds with caniforms [100], [107], [178]–[179].

The carnivorans examined here include two common terrestrial species (*Canis familiaris* and *Felis catus*), as well as the aquatic Stellar sea lion, *Eumetopias jubatus* (Pinnipedia). The dog that was used was a particularly small breed (a Chihuahua). Although the cranium varies to extreme degrees among domestic dog breeds, the vast majority of variation is restricted to the craniofacial region rather than the basicranium [180]–[182]. Furthermore, the bony labyrinth morphology of the specimen studied here does not depart substantially from that of other domestic dogs [72].

**Figure 34:**
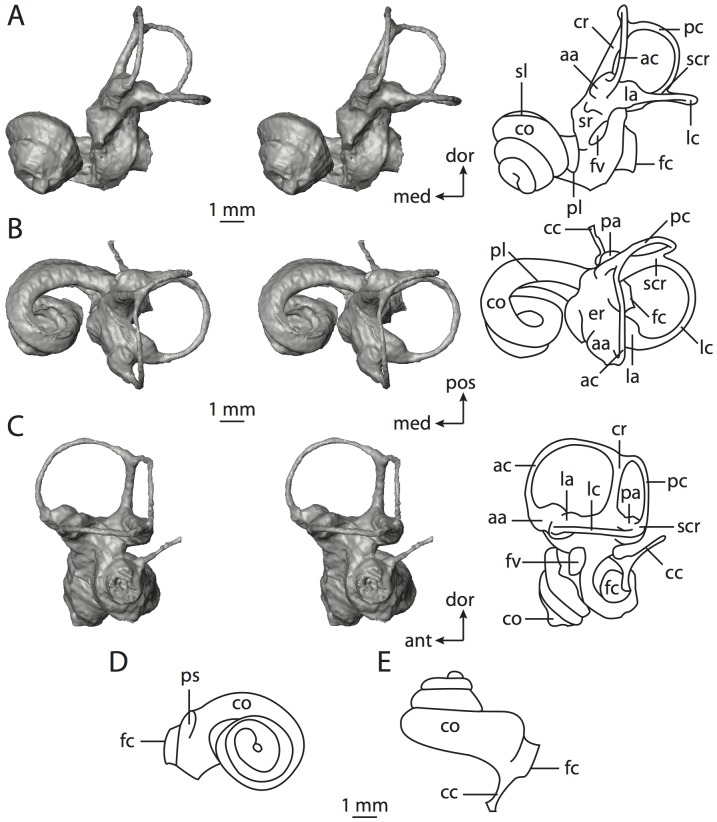
Bony labyrinth of*Canis familiaris*. **A** ****, stereopair and labeled line drawing of digital endocast in anterior view; **B**, stereopair and labeled line drawing of digital endocast in dorsal view; **C**, stereopair and labeled line drawing of digital endocast in lateral view; **D**, line drawing of cochlea viewed down axis of rotation to display degree of coiling; **E**, line drawing of cochlea in profile. Abbreviations listed at the end of the Materials and Methods section.

**Figure 35:**
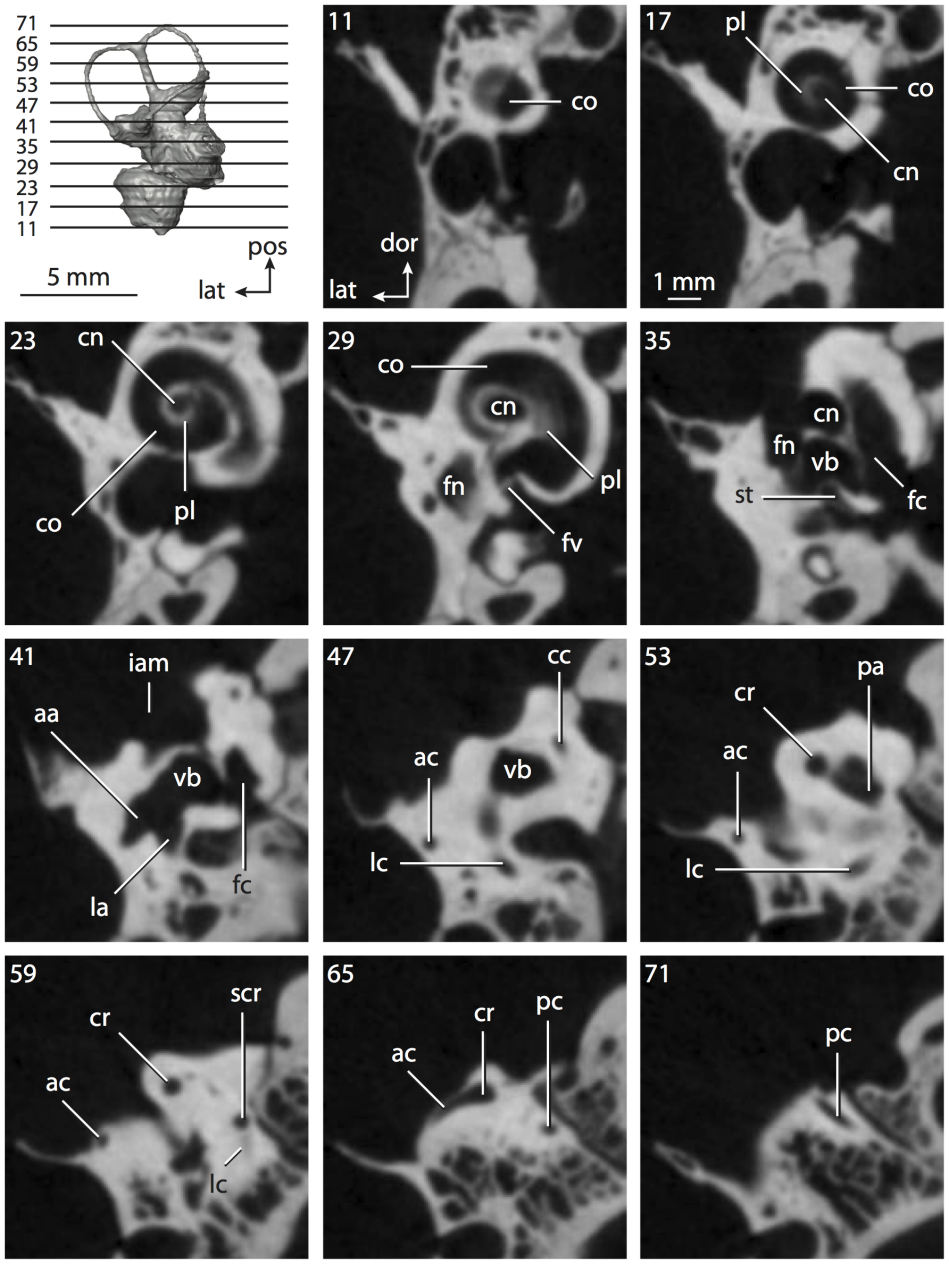
CT slices through ear region of*Canis familiaris*. Abbreviations listed at the end of the Materials and Methods section.

**Figure 36:**
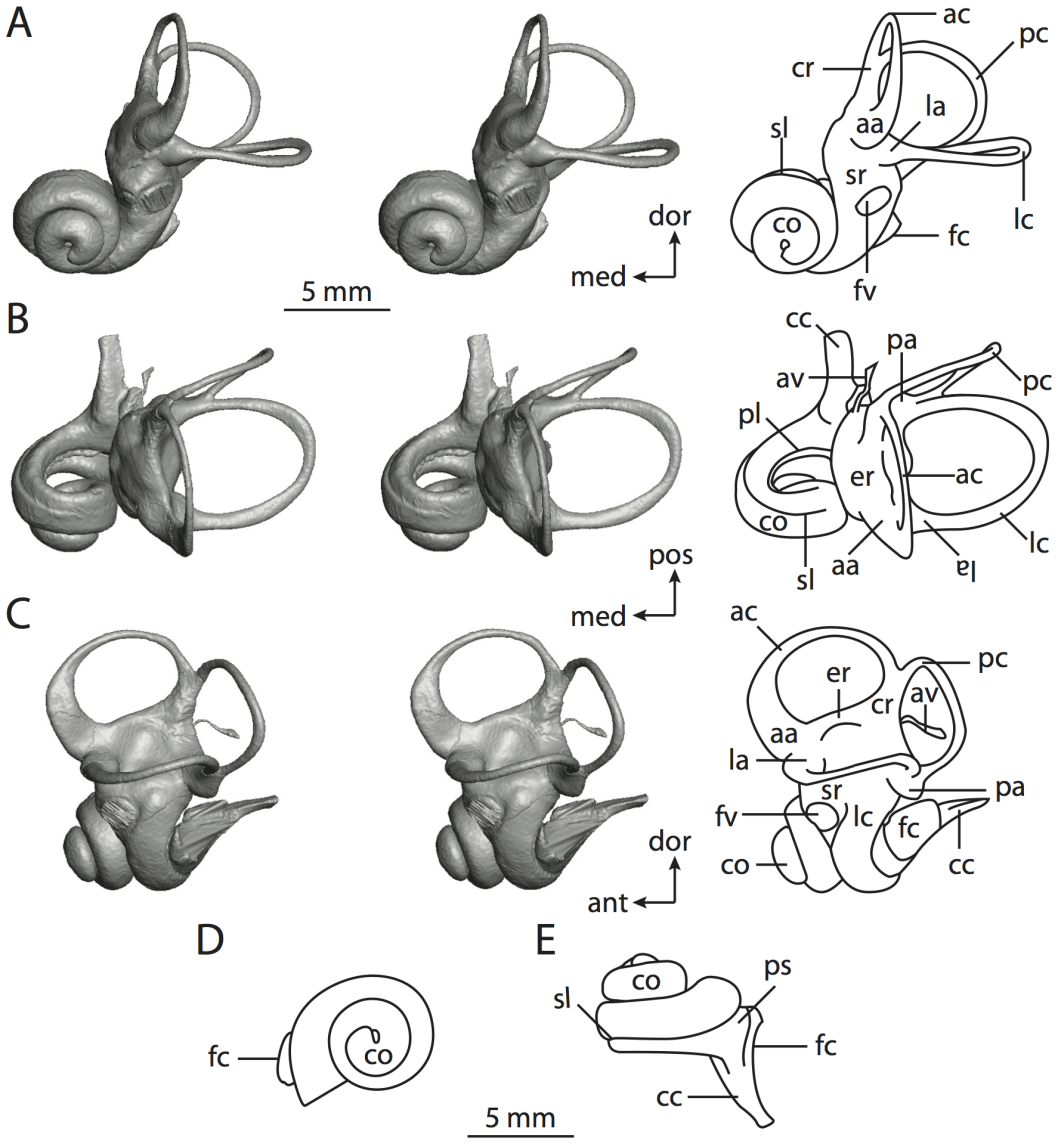
Bony labyrinth of*Eumetopias jubatus* (images reversed). **A**, stereopair and labeled line drawing of digital endocast in anterior view; **B**, stereopair and labeled line drawing of digital endocast in dorsal view; **C**, stereopair and labeled line drawing of digital endocast in lateral view; **D**, line drawing of cochlea viewed down axis of rotation to display degree of coiling; **E**, line drawing of cochlea in profile. Abbreviations listed at the end of the Materials and Methods section.

**Figure 37:**
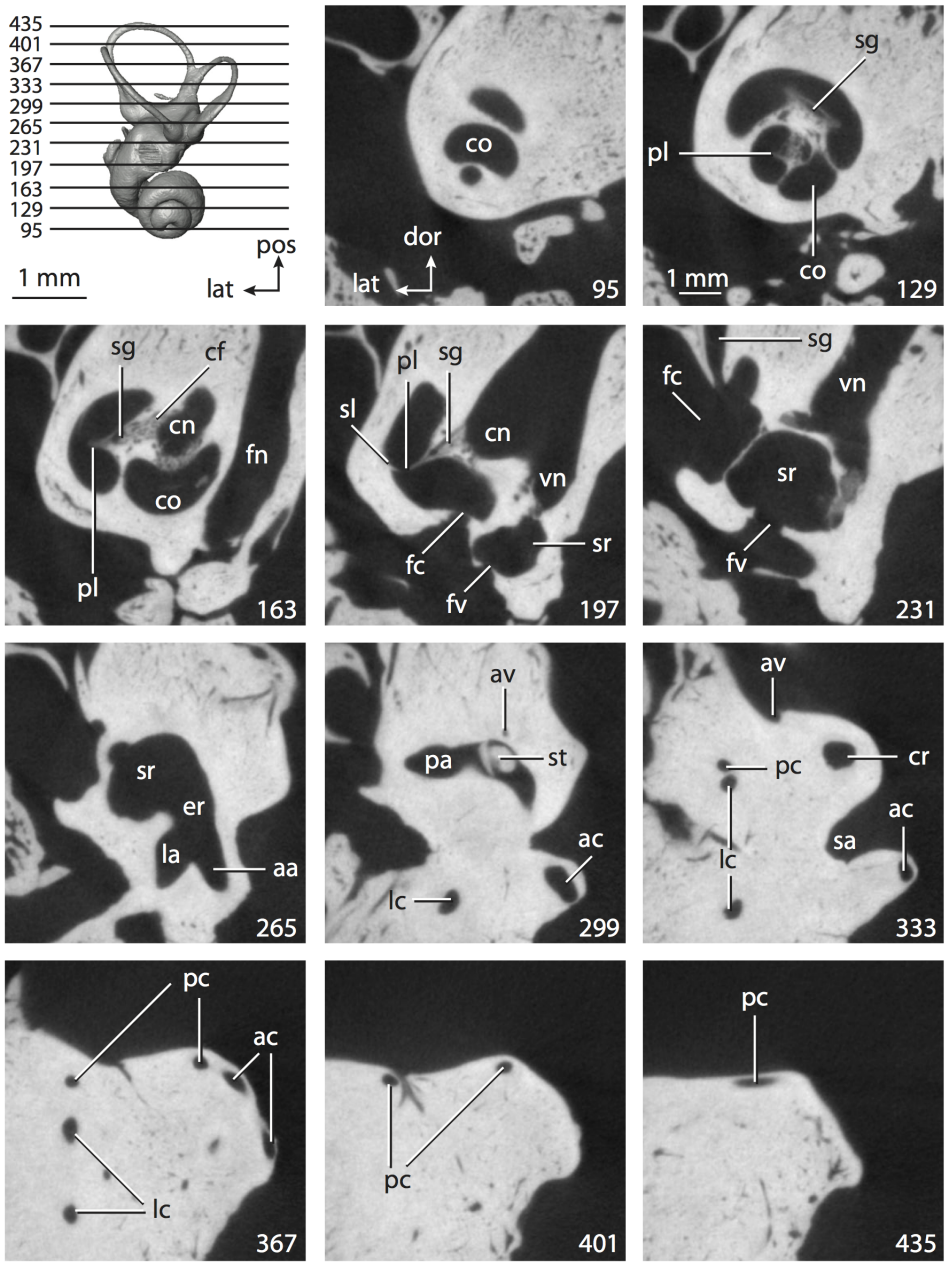
CT slices through ear region of*Eumetopias jubatus*. Abbreviations listed at the end of the Materials and Methods section.

**Figure 38:**
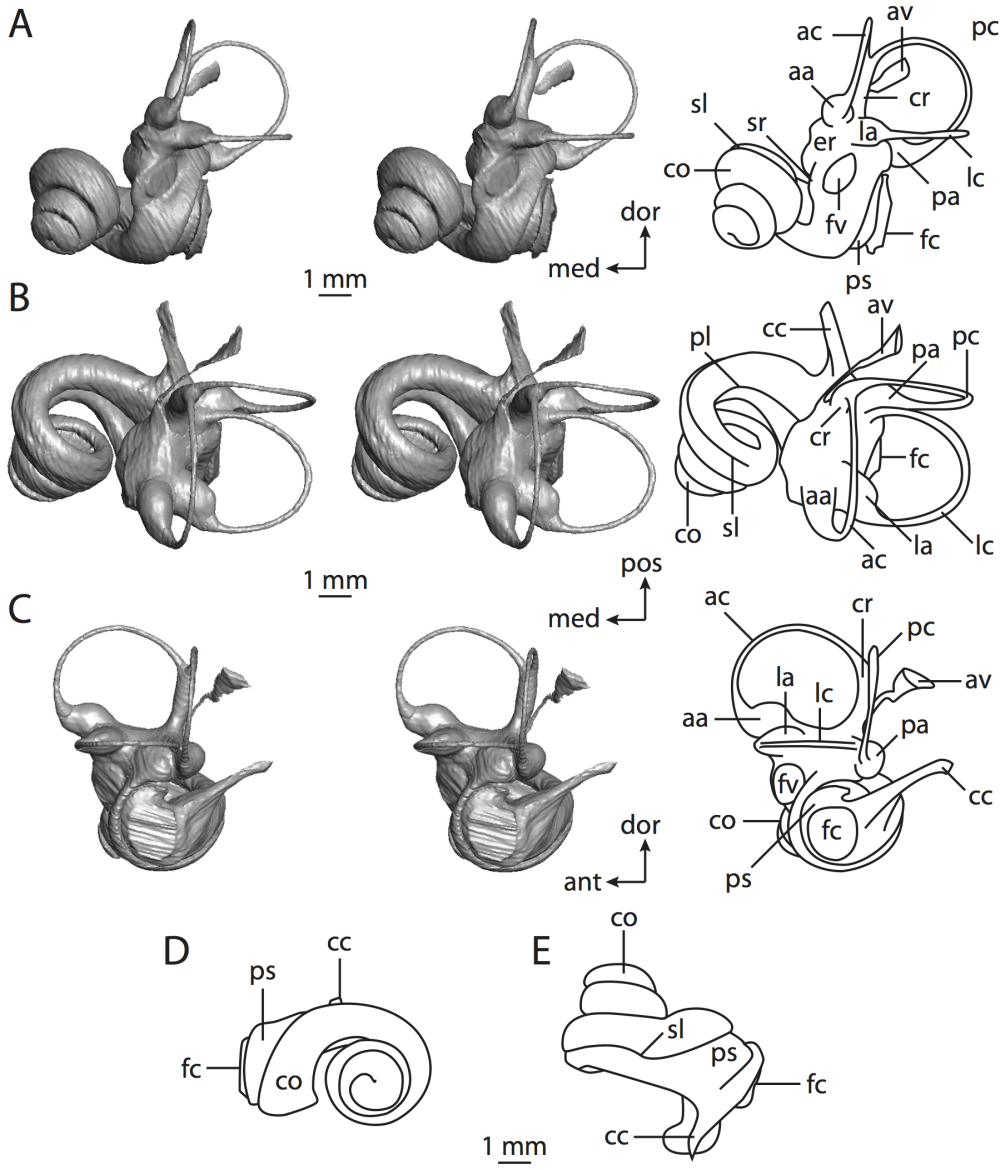
Bony labyrinth of*Felis catus* (images reversed). **A**, stereopair and labeled line drawing of digital endocast in anterior view; **B**, stereopair and labeled line drawing of digital endocast in dorsal view; **C**, stereopair and labeled line drawing of digital endocast in lateral view; **D**, line drawing of cochlea viewed down axis of rotation to display degree of coiling; **E**, line drawing of cochlea in profile. Abbreviations listed at the end of the Materials and Methods section.

**Figure 39:**
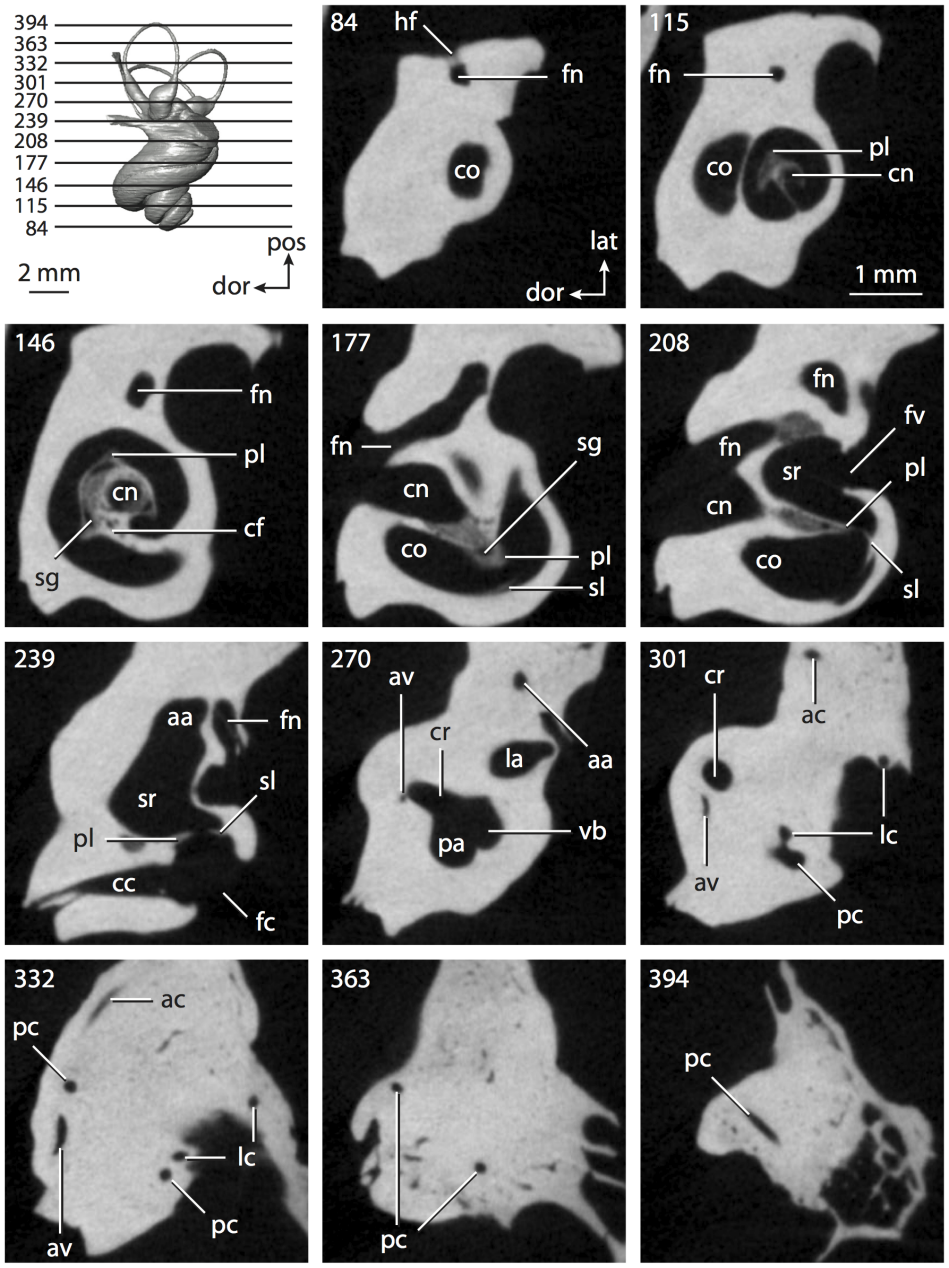
CT slices through ear region of*Felis catus*. Abbreviations listed at the end of the Materials and Methods section.

The sea lion is a much larger animal than the terrestrial carnivorans, with an average body mass of 735 kg [90], than either the cat (3.4 kg) [89] or dog (upwards of 31 kg) [92]. The number of CT slices obtained through the ear regions of *Felis* (627 slices) and *Eumetopias* (498 slices) is significantly greater than the number obtained for *Canis* (92 slices). Because of this, the CT data through the ear region of *Canis* (Figures 34 and 35) are of a lower resolution than those of *Eumetopias* (Figures 36 and 37) and *Felis* (Figures 38 and 39), and minute features of the inner ear of the dog are not discernable (such as the bony channel for the cochlear aqueduct).

The total volume of the inner ear cavities of the *Canis* specimen used in this study is less than that computed for both the *Felis* and *Eumetopias* specimens (Table 1). In fact, the volume of the cochlea of the cat is nearly the same as the volume of the bony labyrinth of the dog as a whole (Tables 1 and 2). The specimen of *Canis* used is from a small dog, which likely explains the size difference. Even so, the percent of the total inner ear volume that is the cochlea is similar between the two terrestrial carnivorans, as the cochlea of *Felis* contributes 68% of the volume, and the cochlea of *Canis* contributes 66%. The cochlea of *Eumetopias* contributes less volume to the labyrinth (54%). Similarly, the length of the inner ear cavity of the dog is not much different than that measured for the cat, and the length of the bony labyrinth of *Eumetopias* is substantially greater than either of the other species, owing to large body size (Table 1).

The length of the cochlear canal in *Eumetopias* is greater than either the dog or the cat (Table 2), and the cochlea of the dog completes a greater degree of coiling (Figure 34D) than the cat (Figure 38D) and especially the sea lion (Figure 36D). A secondary bony lamina is observed extending along the radial wall of the cochlea in all three taxa, although the lamina persists for a greater relative distance in *Eumetopias* and *Felis* than what was measured for *Canis* (Table 2). The aspect ratios of the cochleae of carnivorans are high relative to other species described above. The ratio of the cat (Figure 38E) is higher than that of the dog (Figure 34E), and the ratio calculated for the sea lion is intermediate (Figure 36E). Other species with high aspect ratios include *Macroscelides* and *Sus* (Table 2).

The basal whorl of each carnivoran cochlea is separated from the apical turns, where the apical turns fit within the arc created by the basal turn when the cochlea is in vestibular view (Figures 34D, 36D, and 38D). The apical turns sit upon one another in all three taxa, but each successive whorl is smaller than the turn immediately basal to it, forming a pointed cone beyond the basal whorl. The scala tympani is expanded posterior to the fenestra cochleae in all carnivoran taxa. The expansion of the scala tympani leads to the bony canaliculus cochleae for the cochlear aqueduct (cc in Figures 34 through 36 and 38 through 39). The canaliculus is longer and more robust in *Felis* than in *Canis*, although the longest canaliculus was measured for *Eumetopias* (4.16 mm).

The angle between the planes of the basal turn of the cochlea and the lateral semicircular canal in *Canis* is similar to that measured for the cetaceans and *Sus* (Table 2). However, the angle is greater in *Felis*, being more similar to the hyrax *Procavia capensis* than to the dog. The angle formed between the cochlea and lateral canal of *Eumetopias* is intermediate between the dog and cat, most closely resembling *Orycteropus* (Table 2). The scalae tympani and vestibuli bend around the dorsal border of the fenestra cochleae in the carnivorans. The fenestra vestibuli of *Felis* is elliptical (stapedial ratio in Table 3), although the fenestra of *Canis* is distinctly more circular. The fenestra vestibuli of *Eumetopias* is intermediate between the other carnivorans (Table 3).

The spherical recess of the vestibule is separated from the elliptical recess by a constriction of the vestibule in the carnivoran species. The elliptical recess is gently curved, forming a secondary excavation at its anterior end for the vestibular apertures of the anterior and lateral ampullae in *Canis* and *Felis* (aa and la in Figures 34B and 38B), but not in *Eumetopias* (Figure 36B). Rather, the elliptical recess of *Eumetopias* is concave laterally (er in Figure 36B and C). The anterior excavation of the elliptical recess of the vestibule in both *Canis* and *Felis* is expressed as a pedestal for the ampullae in the digital endocasts. The anterior ampulla of *Eumetopias* forms a teardrop-shaped structure, although the lateral ampulla is deflated and dorsoventrally compressed in this taxon.

The bony channel for the vestibular aqueduct was not observed in *Canis*, owing to the inadequate resolution of the CT data (Figure 35). The structure is observed in both *Eumetopias* and *Felis* (av in Figures 37, slice 299 and 39, slices 270 and 301), in which the bony channel opens ventral to the medial edge of the vestibular aperture of the common crus in both taxa, and bends laterally along its course. As is the case in many of the species described here, the channel for the aqueduct ends in a flattened fissure. The length of the channel is longer in *Felis* than that measured for *Eumetopias* (Table 3).

The straight posterior limb of the lateral semicircular canal opens into the posterior ampulla, rather than the vestibule itself, dorsal to the anterior edge of the vestibular aperture of the posterior ampulla in *Felis*. The position of the lateral semicircular canal in *Felis* is high relative to the other vestibular components. When the endocast of the bony labyrinth of *Felis* is viewed anteriorly, the lateral canal crosses the space enclosed by the posterior semicircular canal (sagittal labyrinthine index in Table 3; lc in Figure 38A). The lateral semicircular canal of *Canis* is situated in a lower position than in *Felis*, and the plane of the canal does not cross the space enclosed by the posterior canal in anterior view (lc in Figure 34A). The lateral semicircular canal of *Eumetopias* empties into the posterior ampulla, as in the cat, but the plane of the canal does not take a high position in the sea lion as it does in the cat (lc in Figure 36A). In this manner, the vestibular apparatus of *Eumetopias* appears more similar to *Canis* among the carnivorans examined here. The lateral extents of the posterior and lateral semicircular canals are equivalent in all three taxa, and none of the carnivoran lateral canals extend posterior to the plane of the posterior canal.

As in the cat and sea lion, the posterior limb of the lateral semicircular canal does not open into the vestibule in *Canis*. Unlike the other taxa, the lateral canal of *Canis* does not open into the posterior ampulla directly either, but rather the lateral canal is fused with the posterior semicircular canal to form a secondary common crus (scr in Figures 34A through C, and 35, slice 59). A secondary crus is developed in the aardvark (*Orycteropus afer*) and also in non-placental mammals.

The semicircular canals of both terrestrial taxa form graceful curves along their courses. The greatest angle between the planes of any two semicircular canals in *Eumetopias* was measured between the anterior and posterior semicircular canals (Table 3). The angle between the canals is also obtuse in *Canis*, but the angle between the anterior and posterior canals in *Felis* is closer to a right angle, although the posterior and lateral semicircular canal planes nearly are perpendicular in *Canis* and *Eumetopias* (Table 3). The angle between the planes of the posterior and lateral semicircular canals of *Felis* is the greatest angle between any two canals in the cat. As with most dimensions within the bony labyrinth, the semicircular canals of *Felis* are larger than the canals of *Canis* (Table 4), although a common pattern across the canal arc radii is observed in both taxa. Namely, the radius of the arc of the anterior semicircular canal is the greatest for both *Canis* and *Felis*. However, the lateral semicircular canal is the largest in terms of radius in *Eumetopias* (Table 4).

The posterior semicircular canal of the cat is the longest of all of the canals in this species, and the lateral canal is the shortest. Likewise, the lateral semicircular canal of *Canis* is the shortest of its canals, but its anterior canal is the longest, rather than its posterior canal as was observed in *Felis* (Table 4). Unlike the terrestrial carnivorans, the lateral canal of *Eumetopias* is the longest. The lateral semicircular canal of *Canis* may be the shortest of the three canals in this species, but the lumen of the lateral canal has the greatest cross-sectional diameter in the dog (Table 4). Similarly, the lateral canal has the greatest diameter in *Eumetopias*. All of the canals of *Felis* were equal in cross-sectional diameter. The aspect ratio of the arc of the lateral semicircular canal is highest in both *Canis* and *Felis*, and the aspect ratio is the smallest for the anterior canal arc for both species (Table 5). The aspect ratios of all three canal arcs are larger in *Eumetopias* than those measured for the terrestrial carnivorans. The ratio of the length of the slender portion over arc radius of the posterior semicircular canal was the greatest among the three canals in *Canis* (5.14; anterior equals 4.97; lateral equals 4.45), *Eumetopias* (4.92; anterior equals 4.33; lateral equals 4.72), and *Felis* (4.93; anterior equals 4.57; lateral equals 4.45).

The lateral semicircular canal is the least planar of the three canals in *Eumetopias* (Table 5). In fact, the lateral canal of *Eumetopias* is the least planar of any semicircular canal measured for any carnivoran specimen examined here. The posterior semicircular canal of *Canis* is the least planar of its three canals. The posterior canal of *Felis* does not deviate from its plane in any substantial manner. The angular deviation of the anterior canal is smaller in *Felis* than in *Canis*, but not by much (Table 5). The anterior semicircular canal of *Eumetopias* deviates by a miniscule amount. None of the canals in either of the terrestrial species deviate substantially from their respective planes. The ratios of the total linear deviation over cross-sectional diameter of the anterior, lateral, and posterior semicircular canals of *Canis* are 0.59, 0.40, and 0.94 respectively, and the ratios for the same canals in *Felis* are 0.57, 0.51, and 0.00 (planar). The anterior canal of *Eumetopias* does not deviate from its plane substantial (linear deviation to lumen diameter ratio equals 0.11), although the lateral and posterior semicircular canals of the sea lion deviate by a substantial amount (ratio of lateral equals 1.70; posterior equals 1.15).

Two labyrinthine characters are synapomorphies for Carnivora within Ferae. The first is the higher aspect ratio of the carnivoran cochlea in profile that gives the cochlear spiral a “sharp-pointed” profile [30]–[31]. The second synapomorphy is the entrance of the lateral canal into the posterior ampulla, which is observed in *Eumetopias* and *Felis*. The secondary common crus observed in *Canis* is an apomorphic reversal to the ancestral therian condition, and it also is observed in *Orycteropus* among crown placentals. The ancestral coiling of the cochlea of Carnivora is over a quarter of a turn greater than that reconstructed for the ancestor of Ferae (987° versus 888°), and the carnivoran cochlea contributes 5% more to the total labyrinthine volume than that of the feran ancestor (61% versus 56%). The position of the lateral canal is reconstructed as high relative to the vestibule for the ancestral carnivoran, despite the low position in caniforms. In addition, the anterior semicircular canal is the largest in ancestral Carnivora.

A single character from the bony labyrinth, reversal to a low position of the lateral semicircular canal in relation to the ampullar entrance of the posterior canal, is optimized as a synapomorphy for Caniformia. The lateral canal of *Felis* is positioned high, which is derived from the ancestral eutherian condition, but is plesiomorphic for Carnivora as a whole. The low position of the lateral canal is a reversal for Caniformia. The lateral semicircular canal enters the posterior ampulla in the ancestral caniform (even though a secondary common crus is present in *Canis*), and the arc of the anterior semicircular canal is the largest of the three canal arcs (even though the lateral canal arc is the largest in *Eumetopias*). The ancestral labyrinth of Caniformia possesses a cochlea with a high aspect ratio that coiled 979° and contributed 60% of the total labyrinthine volume.

#### Pholidota

Although extant species of pangolins are known only from Africa and Asia, fossils of Pholidota have been recovered from Tertiary deposits of Europe and North America [109], [139]. Pangolins have not contributed greatly to the mammalian biota throughout time [139], nor is Pholidota a taxonomically diverse group at present, with only eight species recognized within the single genus *Manis* [138].

**Figure 40:**
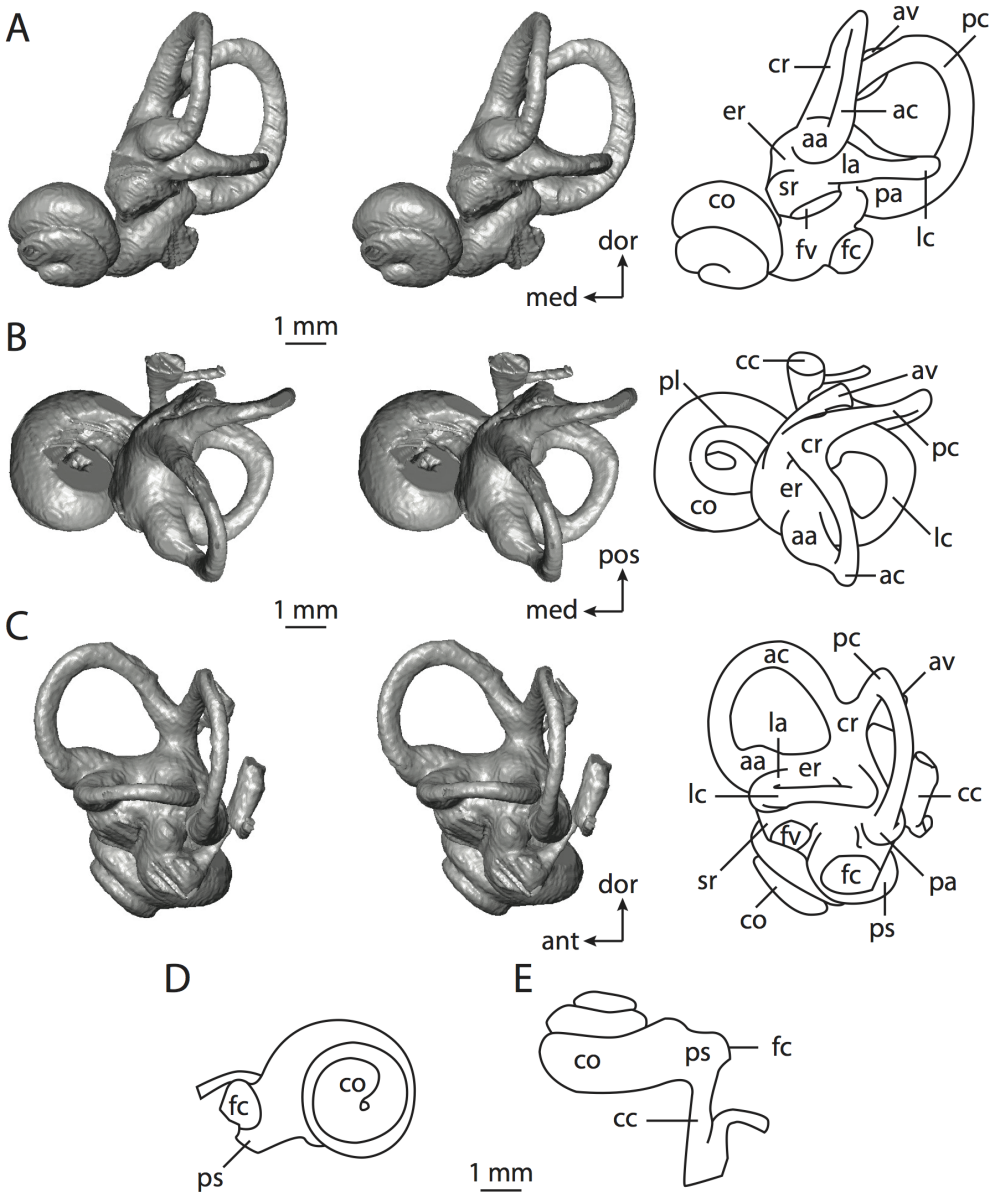
Bony labyrinth of*Manis tricuspis*. **A**, stereopair and labeled line drawing of digital endocast in anterior view; **B**, stereopair and labeled line drawing of digital endocast in dorsal view; **C**, stereopair and labeled line drawing of digital endocast in lateral view; **D**, line drawing of cochlea viewed down axis of rotation to display degree of coiling; **E**, line drawing of cochlea in profile. Abbreviations listed at the end of the Materials and Methods section.

**Figure 41:**
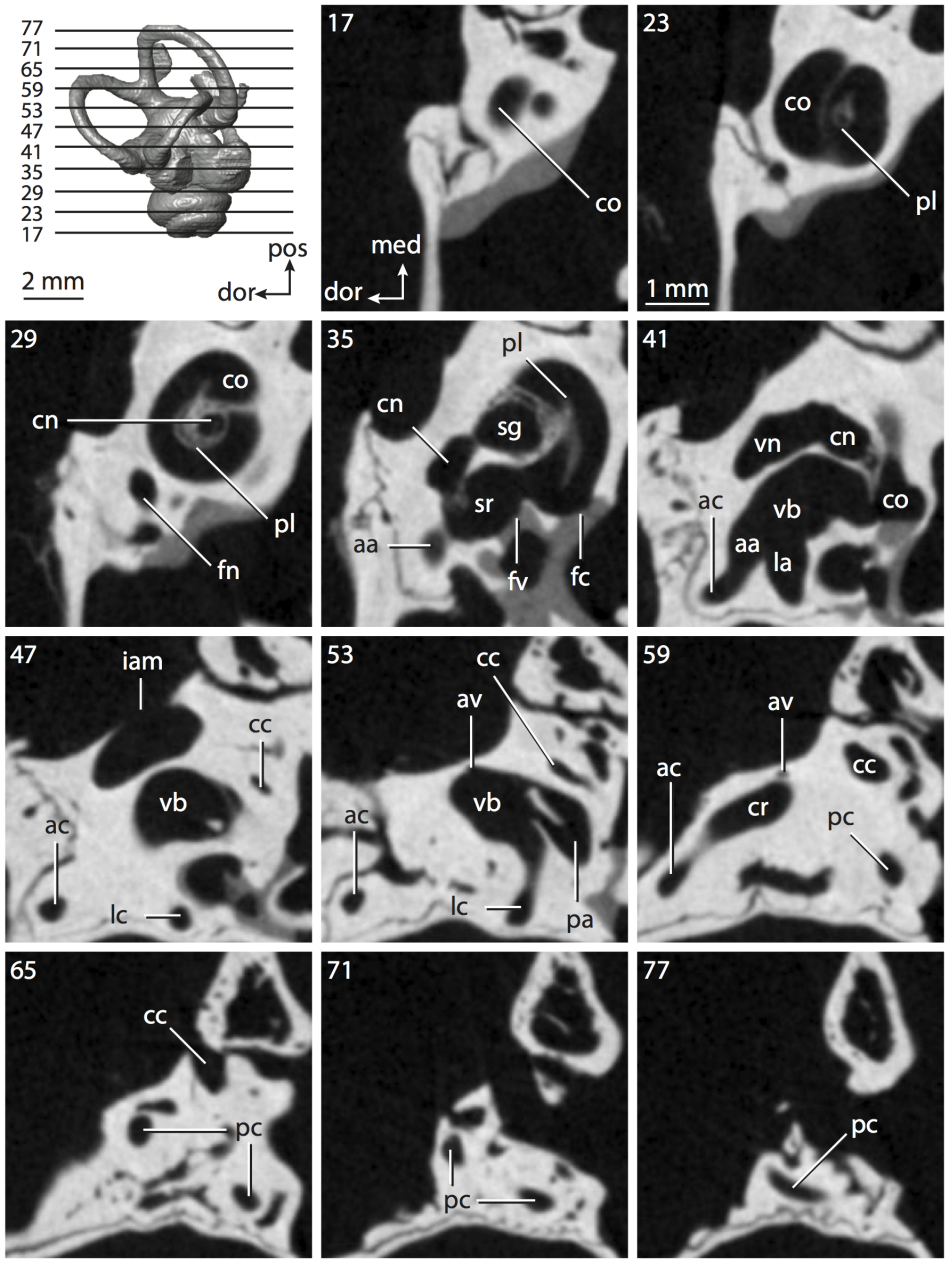
CT slices through ear region of*Manis tricuspis*. Abbreviations listed at the end of the Materials and Methods section.

The gross volume of the inner ear cavities of the pangolin, *Manis tricuspis* (Figures 40 and 41), examined in this study is similar to that of *Bathygenys reevesi* (29.8 mm^3^) and *Dasypus novemcinctus* (Table 1). Likewise, the lengths of the bony labyrinth are not vastly different. The overall body size of *Manis* is similar to *Dasypus* [89], which is reflected in the dimensions of the inner ear (Table 1). The cochlea in *Manis* contributes 49% of the total volume of the bony labyrinth and completes over two and one third turns (Table 2). The apical whorls of the cochlea sit upon the basal turn, rather than fit within the basal turn (Figure 40D) as is observed in cetaceans. A secondary bony lamina is not developed within the cochlea of *Manis* (Figures 40 and 41) as was observed in the terrestrial cetartiodactyls *Bathygenys* and *Sus* (described above) and the xenarthran *Bradypus* [144], but present in other ferans (see above).

The scala tympani of the cochlea is expanded internal to the fenestra cochleae (ps in Figure 40C). The excavation of the scala tympani leads to a robust canaliculus cochleae for the cochlear aqueduct (cc in Figure 40B and C; Figure 41, slices 47 through 65). The canaliculus is oriented dorsally and takes a straight course as it extends to the external surface of the petrosal (2.85 mm in length). The external aperture for the cochlear aqueduct is shared by a second canal, which empties into the lateral aspect of the canaliculus cochleae proximal to the midpoint of the canaliculus (cc in Figure 40B, C, and E). The second canal is not straight but rather hooks dorsally to join the canaliculus cochleae after opening lateral to the canaliculus. A curved canal that fuses to the canaliculus cochleae is not observed in any other mammal. A slight constriction of the vestibule dorsal to the elliptical fenestra vestibuli is the only separation between the spherical and elliptical recesses. Otherwise, the vestibule is a single and undivided unit. The bony channel for the vestibular aqueduct exits the vestibule medial to the vestibular opening of the short and stout common crus (Figures 40B and 41, slices 53 and 59).

The three semicircular canals are especially thick relative to the size of the labyrinth, including the lateral canal, which makes the high position of the canal plane difficult to observe (Figure 40). The posterior limb of the lateral semicircular canal opens into the vestibule anterodorsal to the vestibular aperture of the posterior ampulla (lc in Figures 40C and 41, slice 53). Because the lateral canal empties directly into the vestibule at its posterior end, a secondary common crus is not developed between the lateral and posterior semicircular canals. The position of the plane of the lateral semicircular canal, intersecting points along the lumen of the canal, is high relative to the rest of the vestibular elements (lc in Figure 40A; sagittal labyrionthine index in Table 3). The robusticity of the lateral semicircular canal makes the high position of the canal plane difficult to observe (Figure 40A).

The angle between the planes of the posterior and lateral semicircular canals approach a right angle, and the other angles between canal planes are acute (Table 3). The radius and length of the posterior semicircular canal are largest in *Manis* (Table 4). As stated above, the semicircular canals of *Manis* are thick relative to the total size of the bony labyrinth. As a comparison, the total length of the bony labyrinth of *Manis* is not much less than that of the oreodont *Bathygenys* (Table 1), although the cross-sectional diameter of the anterior, lateral, and posterior semicircular canals of the pangolin aregreater than that measured for the oreodont (Table 4).

The lowest aspect ratio of a semicircular canal arc was calculated for the anterior canal (Table 5). The ratio of the length of the slender portion of the canal to the arc radius is largest for the anterior semicircular canal (4.52). The ratio for the posterior canal is 4.23, and the ratio for the lateral canal in *Manis* is 3.49. The lateral semicircular canal of *Manis* does not deviate from its average plane by any substantial degree (Table 5), nor are the degrees of angular deviation measured for both the anterior and posterior semicircular canals substantial (ratios of total linear deviation over cross-sectional diameter are 0.31 and 0.40 respectively).

The bony labyrinth of *Manis* inherited a direct entry of the lateral semicircular canal into the vestibule from the ancestral placental, and the high position of the plane of the lateral semicircular canal compared to the posterior canal is retained from the ancestor of Boreoeutheria. The low aspect ratio of the cochlea observed in *Manis* is the same as that reconstructed for the ancestor of Eutheria. Because the state of the cochlea could not be reconstructed for the ancestor of Placentalia, the condition in *Manis* is either a primitive retention or a secondary reversal. The arc of the posterior semicircular canal of *Manis* is the largest among the three arcs, which is derived relative to both the ancestor of Boreoeutheria, as well as the most recent common ancestor of Pholidota and Carnivora (for which the anterior arc is the largest).

There are no unambiguous synapomorphies within the inner ear that support an exclusive Carnivora plus Pholidota clade (Ferae). The ancestor of the clade retained features that were present in the ancestor of Placentalia, including entry of the lateral semicircular canal into the vestibule directly, and an anterior semicircular canal arc that was the largest among the three arcs (also present in the ancestor of Theria). The plane of the lateral semicircular canal of the ancestor of Ferae was high compared to the ampullar entrance of the posterior canal, which was the state reconstructed for the ancestor of Boreoeutheria. The state of the aspect ratio of the cochlea was equivocal as reconstructed for the feran ancestor.

#### Chiroptera

**Figure 42:**
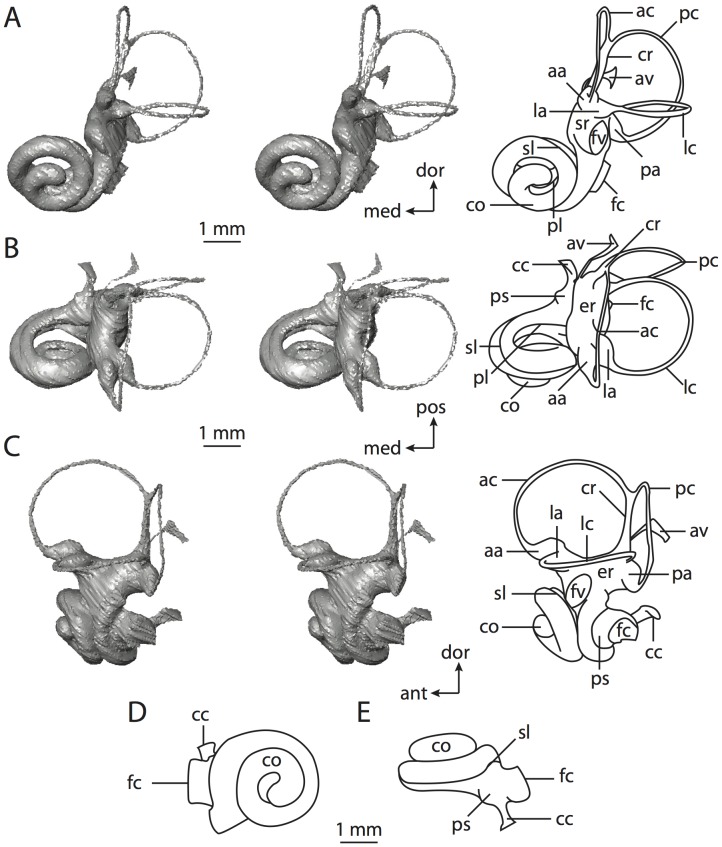
Bony labyrinth of*Pteropus lylei*. **A** ****, stereopair and labeled line drawing of digital endocast in anterior view; **B**, stereopair and labeled line drawing of digital endocast in dorsal view; **C**, stereopair and labeled line drawing of digital endocast in lateral view; **D**, line drawing of cochlea viewed down axis of rotation to display degree of coiling; **E**, line drawing of cochlea in profile. Abbreviations listed at the end of the Materials and Methods section.

**Figure 43:**
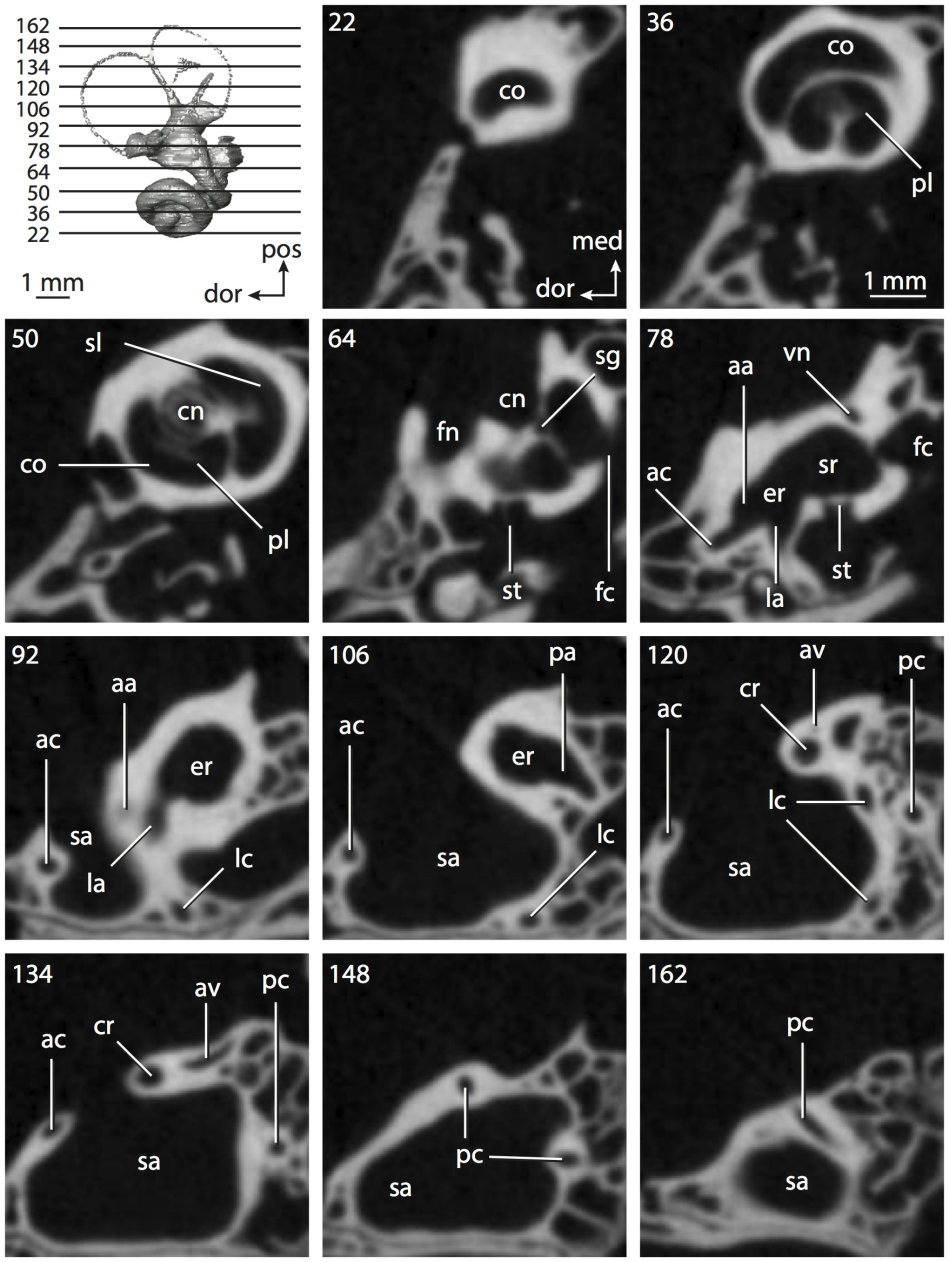
CT slices through ear region of*Pteropus lylei*. Abbreviations listed at the end of the Materials and Methods section.

Chiroptera (bats) is the only group of truly volant mammals, and with over 1,000 species, it forms the second most speciose group of mammals (second only to rodents) [139]. Bats traditionally are separated into Megachiroptera, which includes non-echolocating bats and potentially one group of echolocators (see below), and Microchiroptera, which includes echolocating bats only. The results of several recent molecular studies support a closer relationship between Pteropidae (of which *Pteropus lylei* is used as a representative; Figures 42–43) and the echolocating Rhinolophidae (of which *Rhinolophus ferrumequinum* was examined here) than between Rhinolophidae and other echolocating bats, which are represented here by the Nycteridae species *Nycteris grandis* and the Molossidae species *Tadardia brasiliensis* [183]–[185]. However, the supertree results of some studies [66] separate Pteropidae as the sister taxon to all other bats. Because the morphological descriptions of the bony labyrinth are organized based on the relationships recovered by Bininda-Emonds and others [66] in the present study, description of the inner ear of *Rhinolophus* is included with *Nycteris* and *Tadarida*.

#### Megachiroptera

*Pteropus* includes the bats with the largest body sizes [89], and the average body mass of *Pteropus lylei* is an order of magnitude larger than that of the microchiroperan species examined (see Table 1). The cochlea contributes 59% of the total labyrinthine volume and completes over one and three quarters turns (Table 2; Figure 42D). The secondary bony lamina is present (sl in Figures 42 and 43, slice 50) and persists for nearly the entire basal turn. The aspect ratio of the cochlea in *Pteropus* is high (Table 2) and the cochlear canal itself appears narrow compared to the labyrinth as a whole (Figures 42A and 43, slices 22 through 50). The apical turnings of the cochlea fit within the basal whorl when the cochlea is viewed down its axis of rotation (Figure 42D). The canaliculus cochleae for the cochlear aqueduct is stout and slightly curved.

The spherical and elliptical recesses are undivided, although an excavation is present at the anterior end of the vestibule, which is expressed as a pedestal for the anterior and lateral ampullae on the endocast (aa and la in Figure 42B and C). The common crus and semicircular canals are delicate and form graceful curves in *Pteropus*. The bony channel for the vestibular aqueduct exits the inner ear cavities medial to the vestibular aperture of the common crus. The channel extends posteriorly before terminating in a triangular-shaped fissure. The posterior limb of the lateral semicircular canal enters the vestibule dorsal to the vestibular aperture of the posterior ampulla (lc in Figure 43, slices 92 through 120), giving the plane of the lateral semicircular canal a relatively high position (lc in Figure 42A).

The planes of the posterior and lateral semicircular canals essentially form a right angle, while the angle between the anterior and lateral canals is acute and the angle between the anterior and posterior canal is obtuse (Table 3). The radius of the arc of the anterior semicircular canal is greater than that measured for the lateral and posterior canals (Table 4). This differs from the other dimensions of the semicircular canals in *Pteropus*. For example, the length of the slender portion of the posterior semicircular canal is greater than either the anterior or lateral canal. Although the lateral semicircular canal is the smallest of the three in terms of length of the slender portion of the canal and arc radius, the cross-sectional diameter of the lateral canal is greater than that measured for either the anterior or posterior semicircular canal (Table 4).

None of the semicircular canals fit on a single plane, although the total angular deviation of the posterior canal (Table 5) is not substantial (ratio of total linear deviation over cross-sectional diameter is 0.52). On the other hand, the anterior and lateral semicircular canals of *Pteropus* deviate from their average planes by a substantial amount (ratios of linear deviation over canal diameter are 1.63 and 1.35 respectively). The aspect ratio of the lateral semicircular canal arc of *Pteropus* is the highest among the three canals (Table 5). The high ratio of the lateral semicircular canal arc indicates that the height and width of the arc are nearly identical. The ratio of the length of the slender portion of the canal to the canal arc radius of the posterior semicircular canal is the greatest (5.20; ratio of anterior equals 4.37; ratio of lateral equals 4.56).

The ancestor of Chiroptera retained ancestral placental features, including a lateral semicircular canal that was positioned high compared to the posterior canal and that opened into the vestibule directly, as well as the anterior semicircular canal arc as the largest among the three arcs. The ancestral chiropteran cochlea had a high aspect ratio (a condition shared with Carnivora), coiled 764°, and contributed 61% to the overall volume of the inner ear cavities (also shared with Carnivora). The labyrinth of *Pteropus* retains all discrete character states from its chiropteran ancestor, but the cochlea of *Pteropus* coils to a lesser degree (656°).

#### Microchiroptera

**Figure 44:**
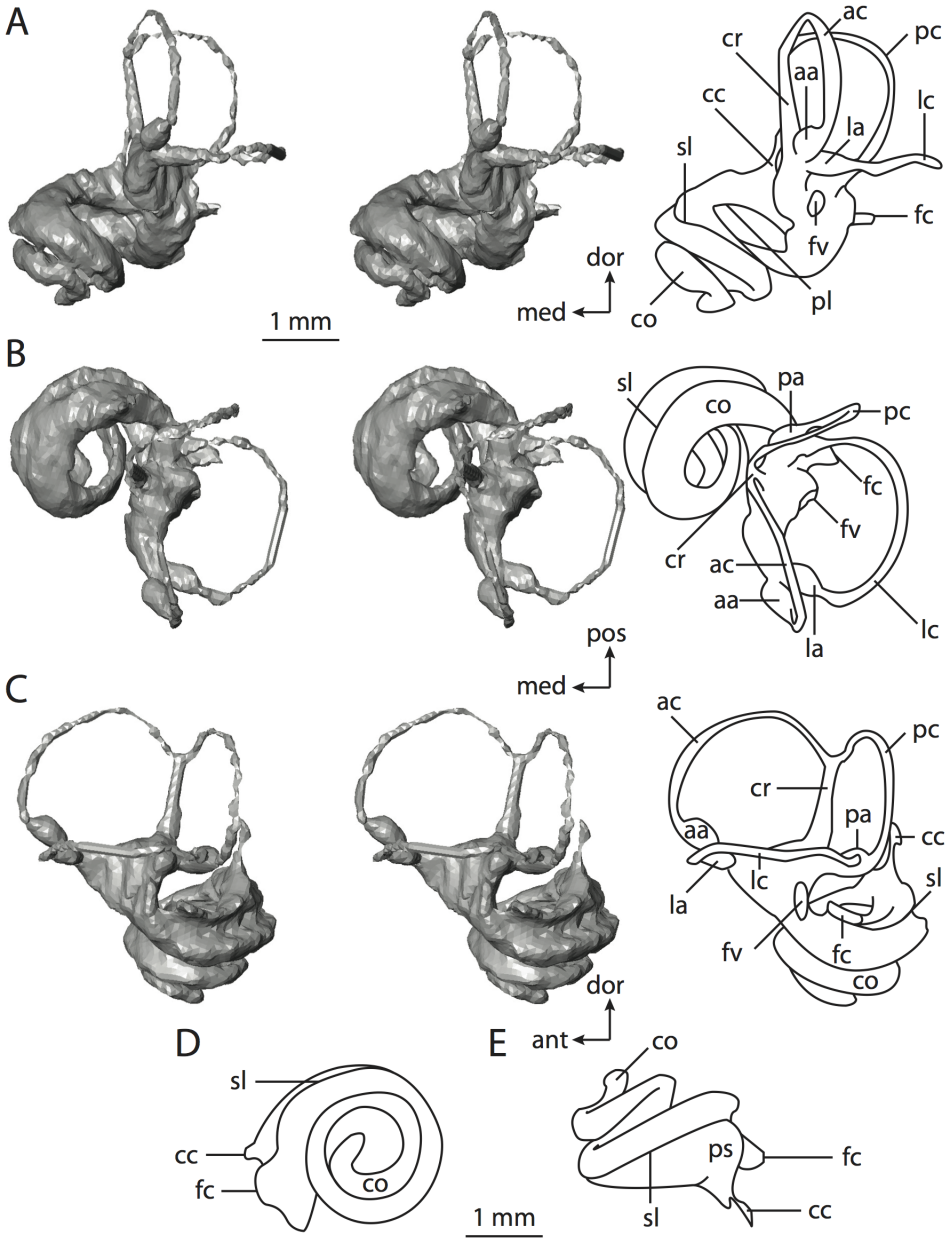
Bony labyrinth of*Nycteris grandis*. **A**, stereopair and labeled line drawing of digital endocast in anterior view; **B**, stereopair and labeled line drawing of digital endocast in dorsal view; **C**, stereopair and labeled line drawing of digital endocast in lateral view; **D**, line drawing of cochlea viewed down axis of rotation to display degree of coiling; **E**, line drawing of cochlea in profile. Abbreviations listed at the end of the Materials and Methods section.

**Figure 45:**
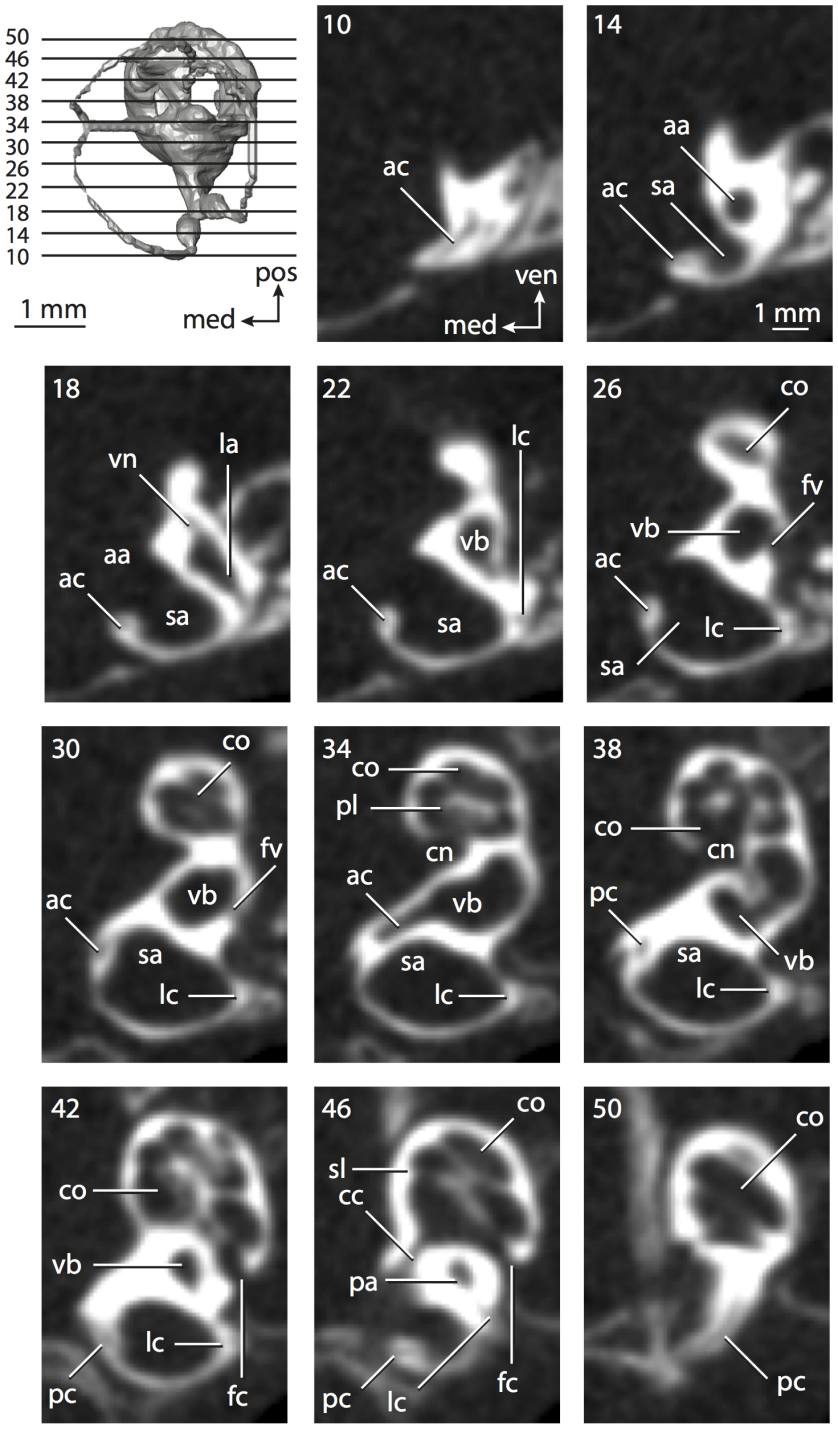
CT slices through ear region of*Nycteris grandis*. Abbreviations listed at the end of the Materials and Methods section.

**Figure 46:**
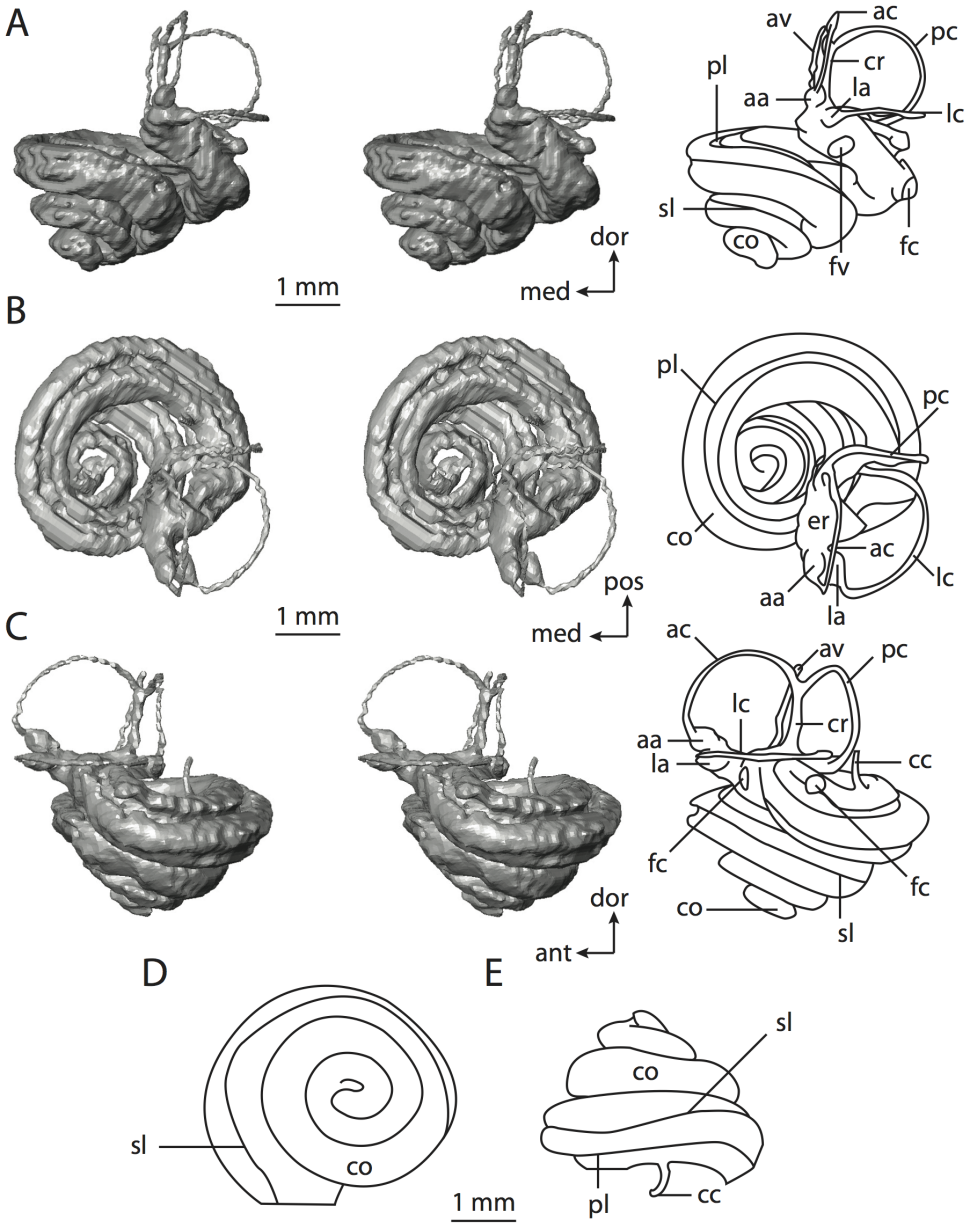
Bony labyrinth of*Rhinolophus ferrumequinum*. **A**, stereopair and labeled line drawing of digital endocast in anterior view; **B**, stereopair and labeled line drawing of digital endocast in dorsal view; **C**, stereopair and labeled line drawing of digital endocast in lateral view; **D**, line drawing of cochlea viewed down axis of rotation to display degree of coiling; **E**, line drawing of cochlea in profile. Abbreviations listed at the end of the Materials and Methods section.

**Figure 47:**
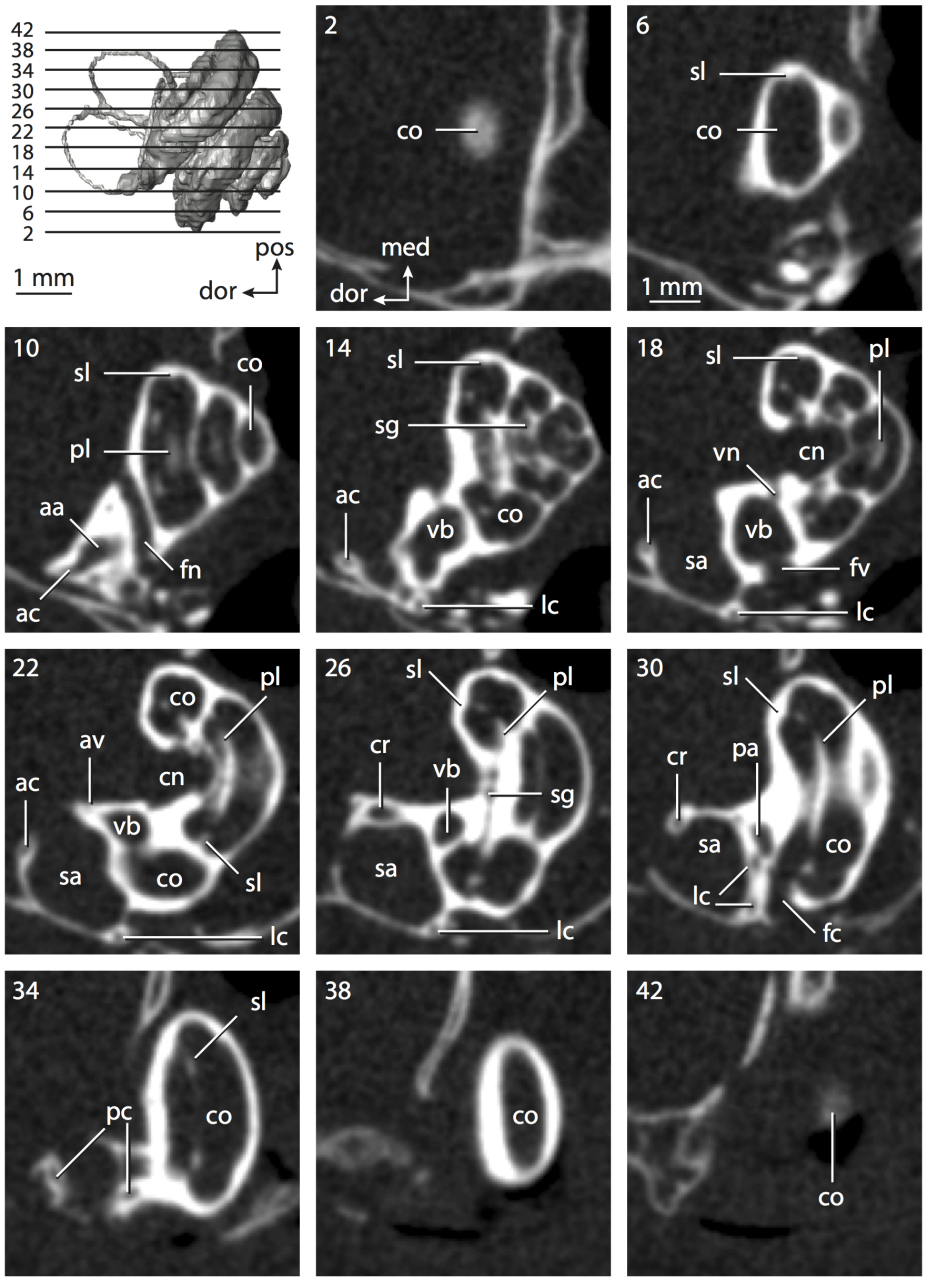
CT slices through ear region of*Rhinolophus ferrumequinum.* Abbreviations listed at the end of the Materials and Methods section.

**Figure 48:**
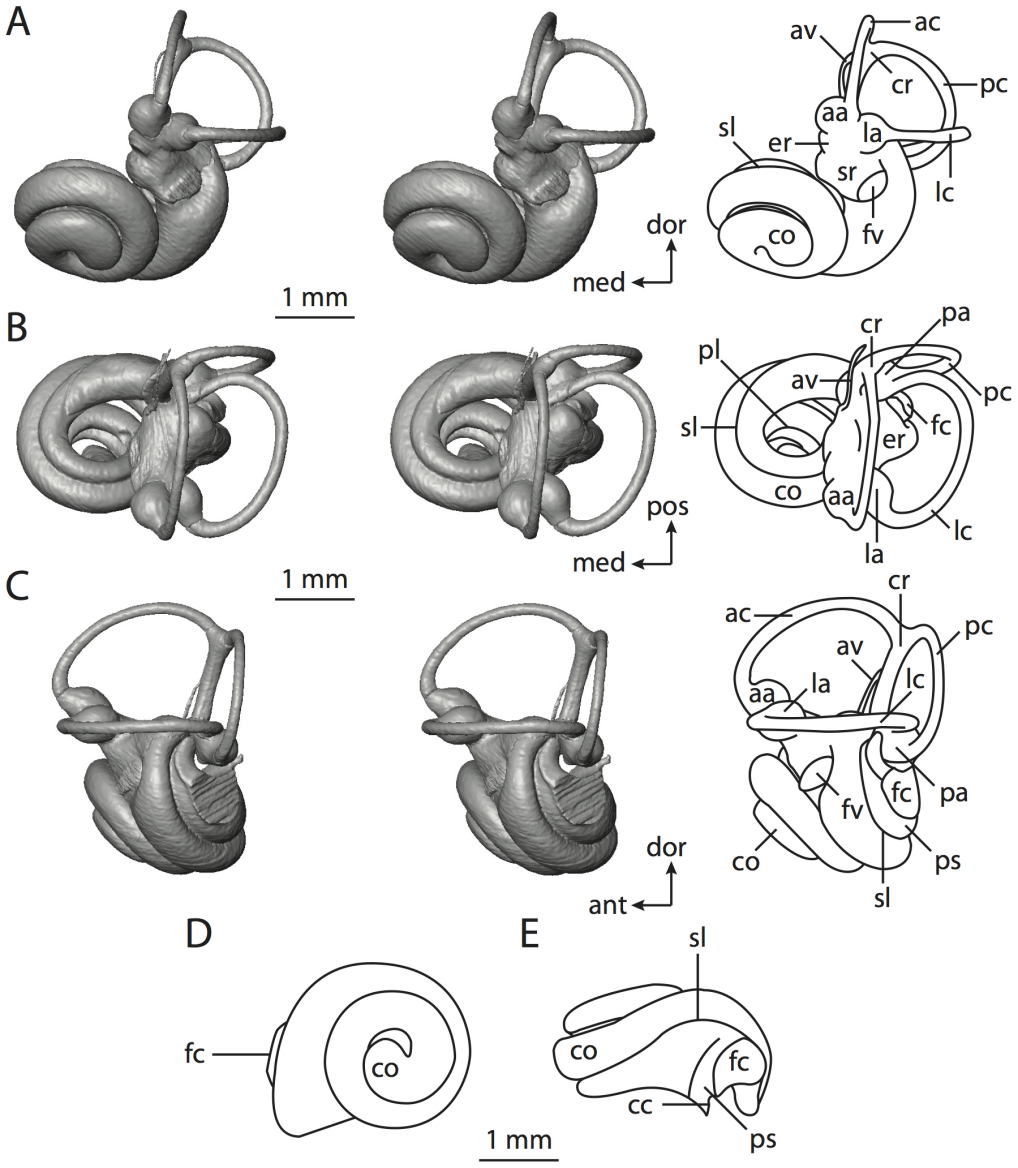
Bony labyrinth of*Tadarida brasiliensis*. **A**, stereopair and labeled line drawing of digital endocast in anterior view; **B**, stereopair and labeled line drawing of digital endocast in dorsal view; **C**, stereopair and labeled line drawing of digital endocast in lateral view; **D**, line drawing of cochlea viewed down axis of rotation to display degree of coiling; **E**, line drawing of cochlea in profile. Abbreviations listed at the end of the Materials and Methods section.

**Figure 49:**
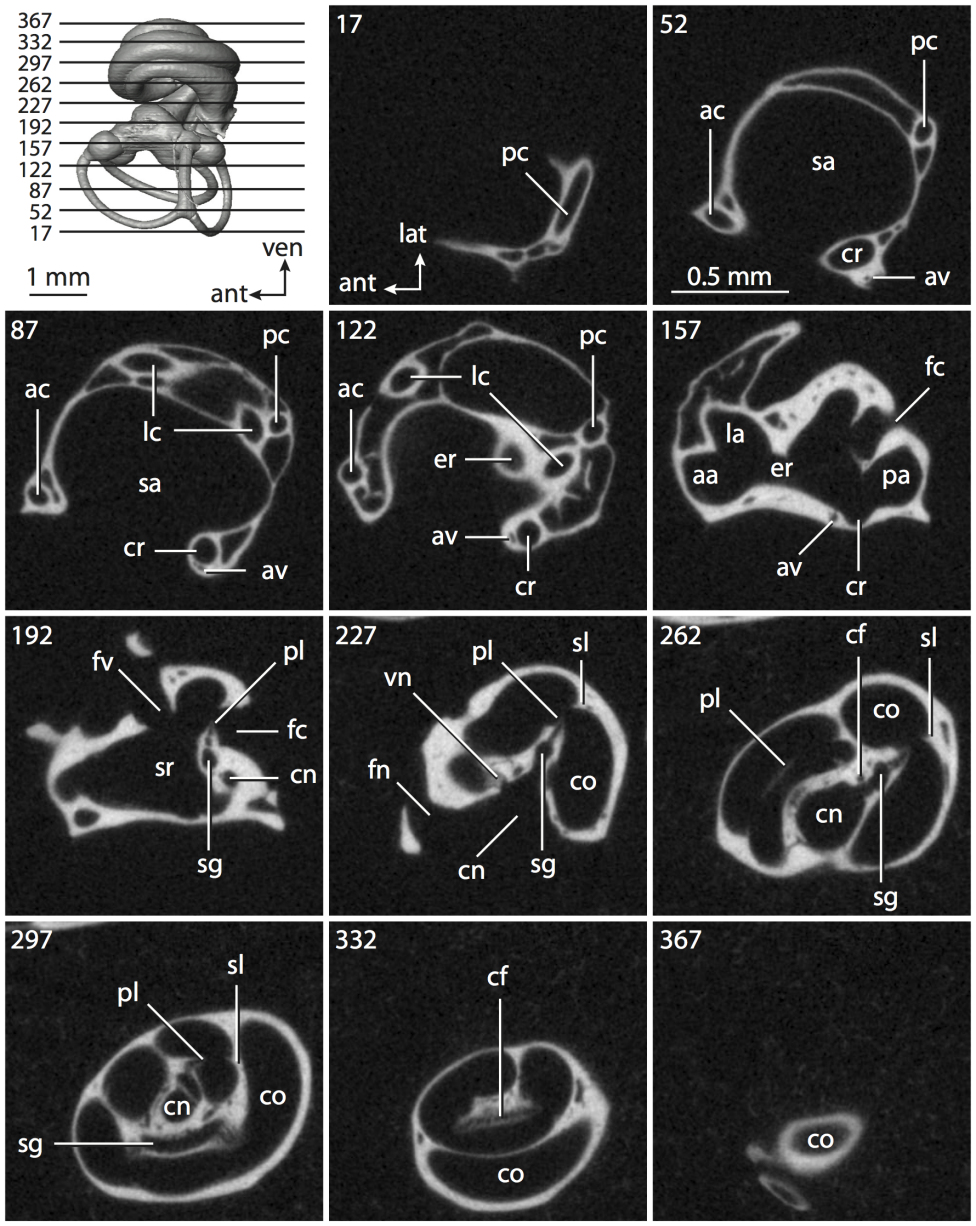
CT slices through ear region of*Tadarida brasiliensis*. Abbreviations listed at the end of the Materials and Methods section.

Among the microchiropteran species examined in the present study, *Nycteris grandis* (Figures 44–45), *Rhinolophus ferrumequinum* (Figures 46–47), and *Tadarida brasiliensis* (Figures 48–49), the species with the largest body mass is *Nycteris* [89]. However, the bony labyrinth of *Rhinolophus* is the largest, both in terms of gross volume of the inner ear cavities as well as the total length of the labyrinth (Table 1). Likewise, the volume of the cochlea of *Rhinolophus* is greater than that measured for either *Nycteris* or *Tadarida* (Table 2). The cochleae of the three species comprise over half of the total inner ear volume. The cochlea of *Nycteris* contributes 67% of the total volume, which is similar to the percentage calculated in *Canis* (66%) and *Felis* (68%). The cochlea comprises 73% of the labyrinthine volume in *Tadarida*, which is similar to the percentage calculated in the afrotherians *Chrysochloris* (71%), *Macroscelides* (72%), and *Trichechus* (71%). The largest volumetric contribution among the bats examined was calculated for *Rhinolophus* (89%). The only other mammals that have a larger contribution than *Rhinolophus* are the cetaceans (contribution in *Tursiops* equals 94%; contribution in the balaenopterid equals 91%).

The cochlea of *Rhinolophus* (co in Figure 46D) completes just over three complete turns, whereas the cochlea of *Nycteris* (Figure 44D) and *Tadarida* (Figure 48D) complete around two to two and one quarter turns (Table 2). Likewise, the length of the cochlear canal of *Rhinolophus* is greater than that measured in either *Nycteris* or *Tadarida*. A secondary bony lamina is present in each of the chiropteran taxa examined. The secondary lamina of *Rhinolophus*, which is expressed as a groove on the endocast (e.g., sl in Figure 47, slices 6–34), persists for the greatest relative distance along the radial wall of the cochlear canal and the least in *Nycteris* (Figure 45, slice 46). The extension of the secondary bony lamina of *Tadarida* (sl in Figure 49, slices 227–297) is intermediate between the other two microchiropterans (Table 2). The secondary bony laminae of *Rhinolophus* and *Tadarida* are the only secondary laminae examined so far to extend beyond the basal turn, with the exception of the secondary lamina in *Tursiops*. The secondary lamina of *Rhinolophus* completes more turns than that of any other mammal examined in this study (Table 2; see also the work of Staněk [186]).

The aspect ratio of the cochlear spiral in profile is the smallest for *Tadarida* (Table 2; Figure 48E; *Nycteris*, Figure 44E *Rhinolophus*, Figure 46E). The apical whorls of the cochlea of *Tadarida* sit upon the basal turn (Figure 48D), whereas the apical whorls fit within the more basal turns in both *Rhinolophus* and *Nycteris* (Figures 44D and 46D). A groove, which is expressed as a ridge on the endocast, situated on the axial wall of the cochlear canal opposite the fenestra cochleae, leads to the short canaliculus cochleae for the cochlear aqueduct in *Tadarida* (cc in Figure 48E). A canaliculus is observed in both *Rhinolophus* (cc in Figure 46C and E) and *Nycteris* (cc in Figure 44C and E), although the groove opposite the fenestra cochleae is not observed in these latter species. The canaliculus is very straight in *Rhinolophus*, and it is oriented nearly perpendicular to the plane of the basal turn of the cochlea (cc in Figure 46C and E).

The plane of the basal turn of the cochlea of *Rhinolophus* forms an angle with the plane of the lateral semicircular canal (lc in Figure 44A) that is the smallest measured between the structures of any mammal described here (Table 2). The angle in *Nycteris* is similar to that measured in *Felis* and the elephantimorph, and the angle in *Tadarida* is not much different from that observed in *Eumetopias* and *Orycteropus*. The fenestra vestibuli is elliptical in *Tadarida*, although the aspect (stapedial) ratio calculated for *Nycteris* is 1.0, indicating a circular fenestra (Table 3). The stapedial ratio, as measured from the fenestra vestibuli, of *Rhinolophus* is low as well. The spherical recess of *Tadarida* is separated from the elliptical recess by a mild constriction of the vestibule (sr and er in Figure 48A). The elliptical recess is elongated, with extensions at its anterior and posterior ends. The recesses are not distinguishable within the vestibule of either *Nycteris* or *Rhinolophus* (Figures 44 and 46 respectively, but as in *Tadarida*, the vestibule of the other two species possess an anterior excavation for the anterior and lateral ampullae, as well as a posterior excavation for the posterior ampulla and common crus. The posterior excavation is best developed in *Rhinolophus* (pa and cr in Figure 46B and C).

The common crura of all three bats are tall and especially slender in *Rhinolophus* (cr in Figure 46) and *Nycteris* (cr in Figure 44). The bony channel for the vestibular aqueduct leaves the inner ear medial and anterior to the vestibular aperture of the common crus in *Tadarida* (av in Figures 48B and 49, slice 157) and *Rhinolophus* (av in Figure 47, slices 22–26), rather than directly medial to the crus as in *Pteropus* (av in Figure 43, slice 120). The channel is straight in *Rhinolophus* and opens on the surface of the petrosal near the apex of the common crus (av in Figure 46C), but the channel of *Tadarida* curves gently towards the posterior end of the labyrinth (av in Figure 48B and C). The presence of a bony channel for the vestibular aqueduct could not be determined for *Nycteris*, because the data were not adequate to resolve the structure (only 70 slices with an interpixel spacing of 0.0654 mm in *Nycteris* versus 380 slices with and interpixel spacing of 0.0097 mm in *Tadarida*; see Table S1). However, the CT dataset of the ear region of *Rhinolophus* contains fewer slices (45) than that of *Nycteris*, yet the channel for the vestibular aqueduct is observed, perhaps because the slices were of a higher resolution (interpixel spacing of 0.043 mm).

The posterior limb of the lateral semicircular canal opens into the posterior ampulla in *Nycteris* (lc in Figure 44B) and *Rhinolophus* (lc in Figure 46B and 47, slice 30), but the canal opens directly into the vestibule anterior to the posterior ampulla in *Tadarida* (lc in Figure 48B). The lateral canals of *Tadarida* and *Rhinolophus* are positioned high with respect to the other vestibular constituents (sagittal labyrinthine indices in Table 3; lc in Figures 46A and 48A), but the canal is comparatively lower in *Nycteris*, in which the lateral canal does not cross the space enclosed by the posterior semicircular canal (lc in Figure 44A).

The angle between the planes of the posterior and anterior semicircular canals is the largest measured in each of the microchiropteran taxa, especially in *Nycteris* (Table 3). The anterior and lateral semicircular canals express the smallest angle in all of the species, especially *Tadarida*. The radius of the arc of the anterior semicircular canal is greater than the other canal radii in the three microchiropteran taxa examined here (Table 4). The smallest arc radius was measured for the lateral semicircular canal arc in *Rhinolophus*. The semicircular canals themselves are longer for *Nycteris* than either *Rhinolophus* or *Tadarida* (Table 4). However, the canals of *Tadarida* are larger in terms of cross-sectional diameter.

The aspect ratio of the arc of the lateral semicircular canal is the lowest among the three canals for the microchiropterans, particularly for *Rhinolophus* (Table 5). Only the aspect ratio of the lateral canal in the balaenopterid is smaller than that calculated for *Rhinolophus*. The highest aspect ratio among the microchiropteran canal arcs was measured for the posterior canal of *Rhinolophus*. The ratio of the length of the slender portion of the canal to arc radius for the posterior semicircular canal was the greatest ratio in *Nycteris* (5.51; anterior ratio equals 4.48; lateral ratio equals 3.91), *Rhinolophus* (5.25; anterior ratio equals 4.25; lateral equals 4.64), and *Tadarida* (4.88; anterior equals 4.62; lateral equals 4.45).

The semicircular canals of *Tadarida* are the most planar among the microchiropterans (Table 5), and none of them deviate substantially from their planes. The posterior canal does not deviate substantially from its plane in *Tadarida*, although the canal is the least planar in both *Rhinolophus* and *Nycteris*. The greatest total angular deviation in *Tadarida* was measured for the lateral semicircular canal (Table 5). The degree of angular deviation of the anterior semicircular canal is substantial in *Rhinolophus* (ratio equals 1.66), but the canal does not deviate substantial in *Nycteris* (ratio equals 0.58).

There are no unambiguous synapomorphies within the bony labyrinth uniting Chiroptera as a whole, nor is there evidence from the inner ear that *Rhinolophus* shares a more recent common ancestor with *Pteropus* than the definitive microchiropterans *Nycteris* and *Tadarida*. However, the lateral semicircular canals of both *Nycteris* and *Rhinolophus* empty into the posterior ampulla, whereas the lateral canals of *Pteropus* and *Tadarida* open into the vestibule directly.

A secondary common crus is not observed in any of the bats examined. In this regard, the bony labyrinth of Chiroptera is derived from that of the ancestral eutherian, but retains this morphology from the ancestral placental. Most of the bats are derived from the ancestral eutherian condition in the position of the lateral semicircular canal in relation to the ampullar opening of the posterior canal, although *Nycteris* retains the ancestral condition. Because of this, the state in the ancestor of Microchiroptera is equivocal as reconstructed. *Tadarida* retains the ancestral therian state of a flattened cochlea, whereas the cochleae of all other bats have a high aspect ratio. Nonetheless, the ancestral microchiropteran condition is a cochlea with a high aspect ratio, which is retained from the ancestor to all of Chiroptera. The largest semicircular canal arc is observed in the anterior canal in all of the bats, although this feature is plesiomorphic and shared with most therian taxa. The cochlea of the microchiropteran ancestor coils 820° and contributes 68% of the total labyrinthine volume, both of which are greater values than those reconstructed for the ancestor of Chiroptera (764°; 61%).

The most recent common ancestor of *Rhinolophus* and *Tadarida* possessed a cochlea with a high aspect ratio, a lateral semicircular canal positioned high compared to the posterior canal, and an anterior semicircular canal arc that was the largest among the three arcs. All of these states also are present in the ancestor of Chiroptera. Because the lateral semicircular canal opened into the vestibule in *Tadarida* and into the posterior ampulla in *Rhinolophus*, the state in the most recent common ancestor of these taxa was reconstructed as equivocal. The cochlea of this ancestor coiled 896°, and contributed 76% of the entire labyrinthine volume. Although the cochlea contributed a great amount of the labyrinthine volume, it was not as great as that reconstructed for Cetacea (84%).

#### Eulipotyphla

The sister taxon to the Perissodactyla+Cetartiodactyla clade, Ferae, and Chiroptera polytomy is Eulipotyphla. The constituents of Eulipotyphla are Erinaceidae (hedgehogs), Soricidae (shrews), Talpidae (moles), *Solenodon*, and the extinct genus *Nesophontes* [187]. These taxa traditionally were grouped with the afrosoricid Tenrecidae and Chrysochloridae within the paraphyletic Lipotyphla [107], which in turn was a subset of Insectivora [100], that also included Macroscelidea. Although most recent phylogenetic analyses fail to support insectivoran or lipotyphlan monophyly, a monophyletic Eulipotyphla often is recovered [66], [98]–[99], [112], [118], [188]–[189]. However, eulipotyphlan monophyly is not always found, even among molecular studies that group the afrosoricids with other afrotherians [190]–[191].

**Figure 50:**
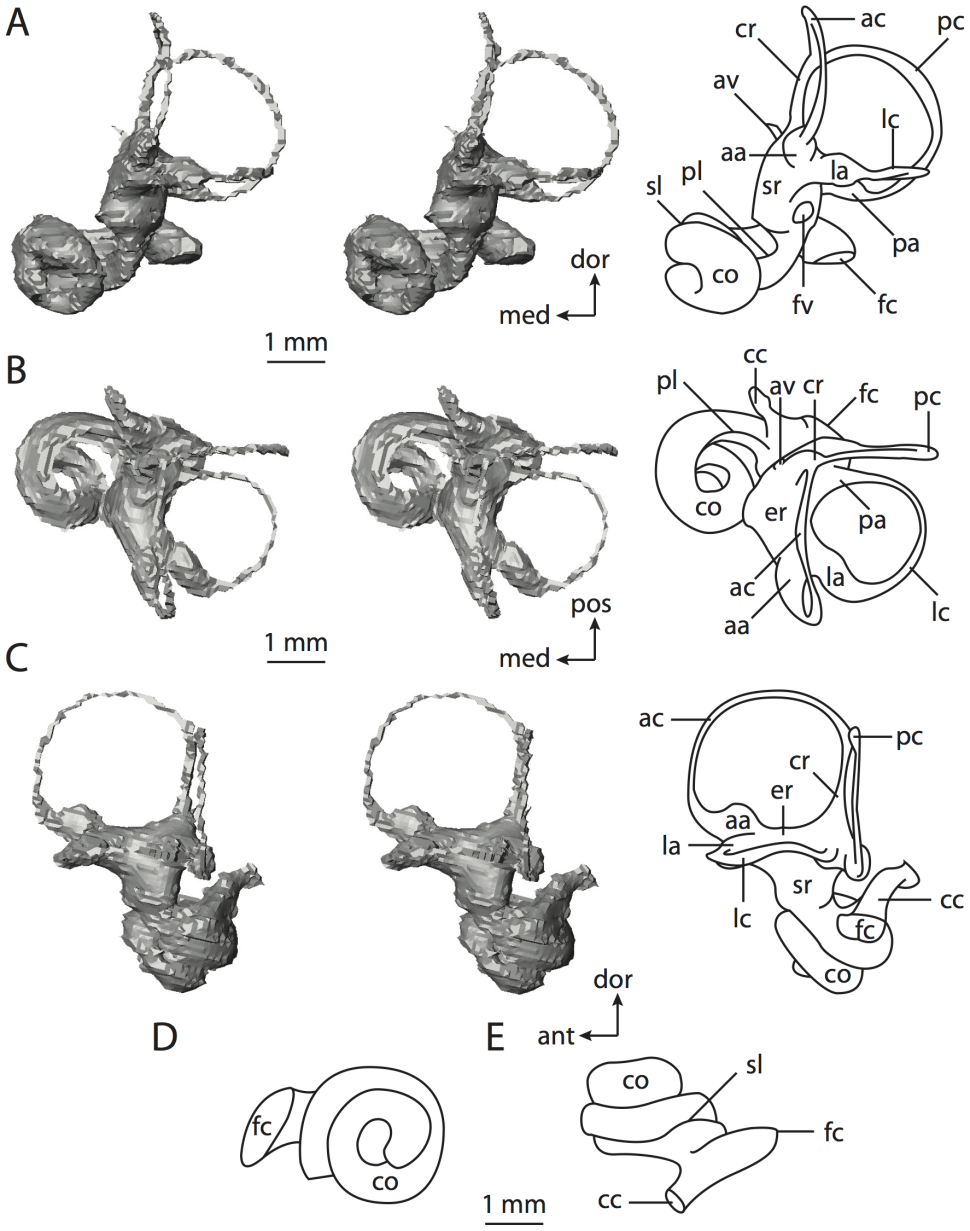
Bony labyrinth of*Atelerix albiventris*. **A**, stereopair and labeled line drawing of digital endocast in anterior view; **B**, stereopair and labeled line drawing of digital endocast in dorsal view; **C**, stereopair and labeled line drawing of digital endocast in lateral view; **D**, line drawing of cochlea viewed down axis of rotation to display degree of coiling; **E**, line drawing of cochlea in profile. Abbreviations listed at the end of the Materials and Methods section.

**Figure 51:**
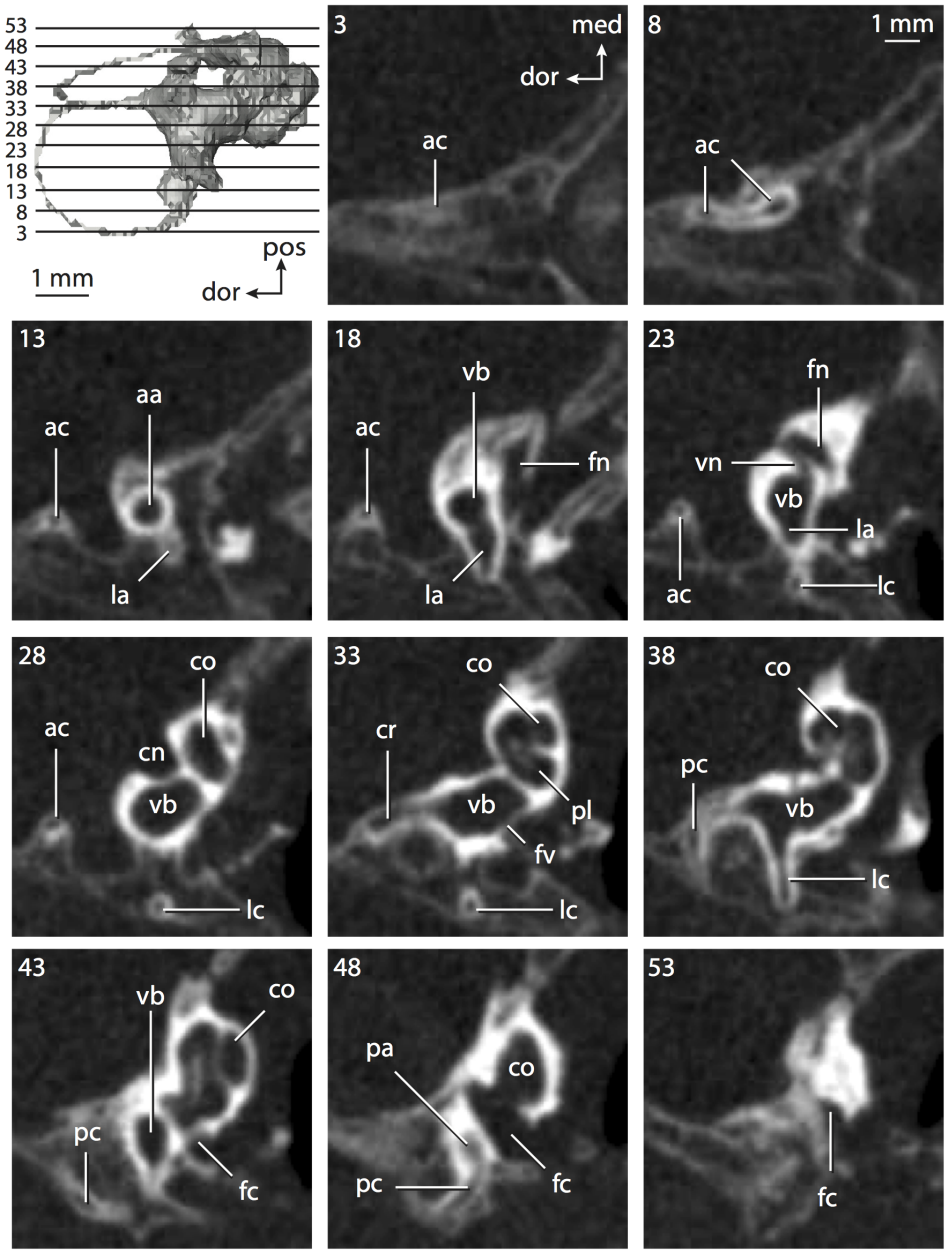
CT slices through ear region of*Atelerix albiventris*. Abbreviations listed at the end of the Materials and Methods section.

**Figure 52:**
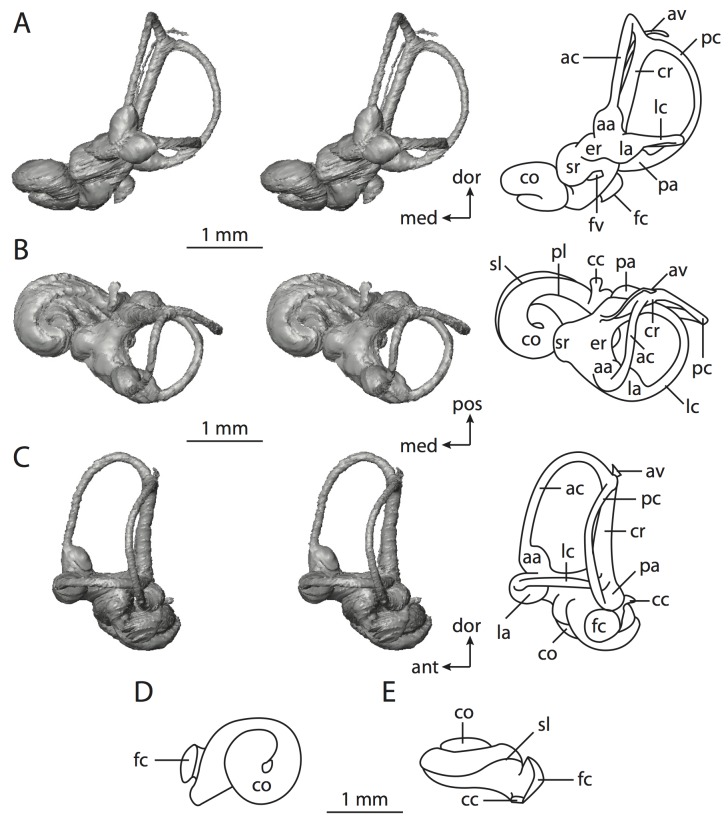
Bony labyrinth of*Sorex monticolus*. **A** ****, stereopair and labeled line drawing of digital endocast in anterior view; **B**, stereopair and labeled line drawing of digital endocast in dorsal view; **C**, stereopair and labeled line drawing of digital endocast in lateral view; **D**, line drawing of cochlea viewed down axis of rotation to display degree of coiling; **E**, line drawing of cochlea in profile. Abbreviations listed at the end of the Materials and Methods section.

**Figure 53:**
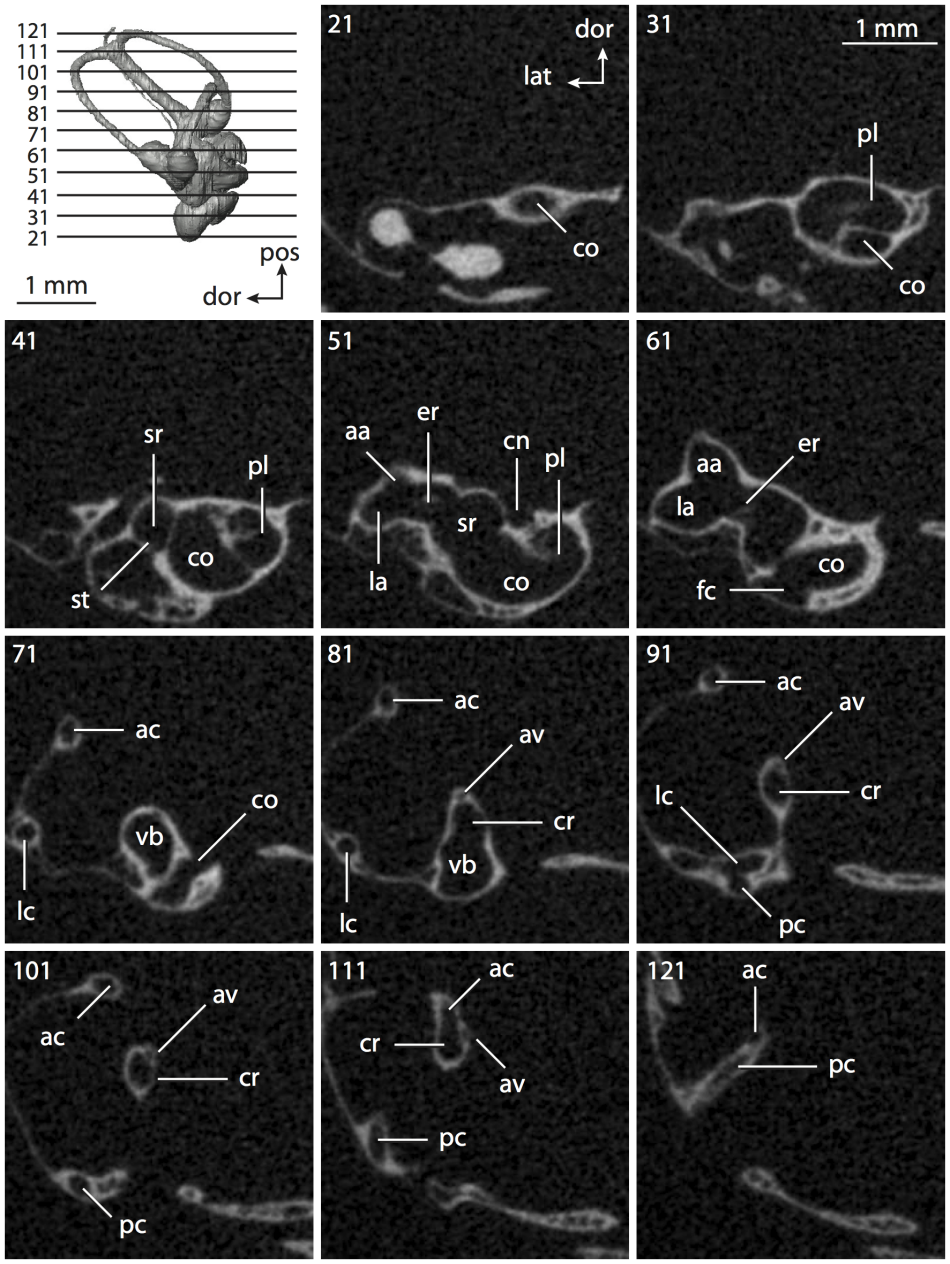
CT slices through ear region of*Sorex monticolus*. Abbreviations listed at the end of the Materials and Methods section.

The eulipotyphlans form the third most speciose clade of placental mammals [137]–[138], most of which belong to the subclade Soricomorpha (shrews, moles, solenodons, and nesophontids). The sister taxon to the soricomorphs are the hedgehogs, which belong to the group Erinaceomorpha. Both major subclades of eulipotyphlan are represented - the hedgehog *Atelerix albiventris* (Figures 50–51) and the shrew *Sorex monticolus* (Figures 52–53).

*Atelerix* is significantly larger bodied than *Sorex* [89] and the bony labyrinth of *Atelerix* is larger, both in terms of length and volume (Table 1). The volume of the cochlear canal of *Atelerix* is greater than that measured for *Sorex* (Table 2), although the respective contributions of the cochlea to total inner ear volume (50% versus 46%) are similar. Not only is the length of the cochlear canal of *Atelerix* longer than that of *Sorex* (Table 2), the cochlea of the hedgehog completes a greater degree of coiling (Figure 50D) than the shrew (Figure 52D). The secondary bony lamina of *Sorex* persists for the first half of the basal turn of the cochlea, whereas the secondary lamina persists for two thirds of the basal turn in *Atelerix*. The aspect ratio of the spiral of the cochlea in profile of *Atelerix* (Figure 50E) is greater than the ratio calculated for *Sorex* (Figure 52E).

The plane of the basal turn of the cochlea forms a larger angle with the plane for the lateral semicircular canal in *Atelerix* than that measured for *Sorex* (Table 2). The canaliculus cochleae for the cochlear aqueduct is longer in *Atelerix* versus *Sorex* as well (Table 2). The scala tympani of the cochlea is expanded internal to the fenestra cochleae, leading to the canaliculus in both eulipotyphlan taxa. The expansion in *Atelerix* is elongated, and curves ventrally, forming a hook on the endocast that terminates with the fenestra cochleae (fc in Figure 50A and D). Elongation of the expansion is not observed in *Sorex* (fc in Figure 52).

The fenestra vestibuli of both eulipotyphlan taxa are elliptical (stapedial ratio in Table 2). The vestibules of both taxa are constricted, separating the spherical and elliptical recesses, the latter with anterior and posterior excavations in both *Atelerix* and *Sorex*. The bony channel for the vestibular aqueduct opens medial to the vestibular aperture of the common crus in *Atelerix*, but the channel exits the inner ear cavities anterior to the crus in *Sorex*. The relative lengths of the channels are different between the eulipotyphlans (Table 3). The channel is very short and straight in *Atelerix* (av in Figure 50A), although the channel extends for a greater distance in *Sorex*, where the delicate canal parallels the common crus for much of its length before turning posteriorly to open on the external surface of the petrosal (av in Figures 53 and 53, slices 81 though 101).

The posterior limb of the lateral semicircular canal opens directly into the vestibule in both taxa (lc in Figure 51, slice 38 for *Atelerix*; Figure 53, slice 91 for *Sorex*), although the vestibular aperture of the canal of *Atelerix* is further separated from the base of the posterior ampulla than the canal in *Sorex*. The lateral canal is positioned high relative to the posterior semicircular canal in both the hedgehog and shrew, particularly in *Atelerix* (lc in Figure 50A and 52A). The angle between the planes of the posterior and lateral semicircular canals is the greatest between canals in *Atelerix*, although the angle between the anterior and posterior canals in the hedgehog labyrinth is not much different (Table 3). The angle between the anterior and lateral canals is significantly more acute. The greatest angle in *Sorex* was measured between the anterior and posterior canals, but as was measured for *Atelerix*, the angle between the posterior and lateral canals is similar.

The arc radius of curvature of the anterior semicircular canal is largest in both *Atelerix* and *Sorex*, although the radius of the posterior arc is similar to that of the anterior in both taxa (Table 4). The anterior semicircular canal of *Atelerix* is longer than either the lateral or posterior canals, although the longest canal in *Sorex* is the posterior semicircular canal. The anterior and posterior semicircular canal volumes are the same in *Atelerix* (0.04 mm^3^ each), which is a greater value than the lateral canal (0.03 mm^3^). The volumes of all of the canals in *Sorex* are identical (0.02 mm^3^). The cross-sectional diameters of the anterior and posterior semicircular canals are the same in *Sorex*, which is a smaller value than that measured for the lateral canal (Table 4). The diameter of the anterior canal is largest in *Atelerix*.

The lateral and posterior semicircular canal arcs of *Atelerix* approach perfect circles (height and width nearly identical) with respective aspect ratios close to 1.0 (Table 5; lc and pc in Figures 50 and 52). The ratio of length of the slender portion of the posterior semicircular canal to its arc radius is the greatest in *Sorex* (5.44), and the ratio of the lateral canal is the smallest in the shrew (3.38). The ratio of the lateral canal in *Atelerix* is the smallest (4.15), and the ratio is identical for the anterior and posterior canals (4.74).

Among the semicircular canals of both eulipotyphlan taxa, the posterior canal *Sorex* deviates the most from its plane (pc in Figures 52C and Figure 50C). The least planar canal of *Atelerix* is the lateral semicircular canal (Table 5), which does not deviate substantially from its plane in the shrew. The degrees of deviation for the anterior, lateral, and posterior semicircular canals are substantial for *Atelerix* (ratios of total linear deviation over cross-sectional diameter equal 1.40, 2.00, and 2.22 respectively), but only the posterior canal of *Sorex* exhibits substantial deviation (ratio is 1.87; anterior is 0.78; lateral is 0.00).

No features of the bony labyrinth support monophyly of Eulipotyphla, nor are there any unambiguous characters that unite the eulipotyphlans with the afrosoricids (*Chrysochloris* and *Hemicentetes*). Both *Sorex* and *Atelerix* are derived from the ancestral eutherian condition in that the lateral semicircular canal enters the vestibule directly rather than forming a secondary common crus with the posterior canal, as well as a high position of the plane of the lateral canal in relation to the ampullar opening of the posterior semicircular canal. Vestibular entry of the lateral canal is inherited from the ancestor of Placentalia. The cochlea of *Atelerix* is derived from the ancestral eutherian in that the aspect ratio of the spiral is high, whereas the cochlea of *Sorex* retains the primitive flattened condition reconstructed for the ancestor of Theria.

The ancestral states of Eulipotyphla include a lateral semicircular canal that opens into the vestibule directly (retained from placental ancestor) and is positioned high compared to the ampullar entrance of the posterior canal (retained from boreoeutherian ancestor), and an anterior semicircular canal arc with the largest radius among the three arcs (retained from therian ancestor). The state of the aspect ratio of the cochlea is reconstructed as equivocal for the ancestor of Eulipotyphla. The cochlea of the most recent common ancestor of *Atelerix* and *Sorex* contributes 50% of labyrinthine volume and the cochlear canal coils 623°. The low degree of coiling of the ancestral eulipotyphlan either is a retention from its therian ancestor, or else a reversal to a more ancestral morphology.

#### Euarchontoglires

Euarchontoglires contains the remaining placental mammal clades. Among these are the highly speciose Rodentia, Lagomorpha, Primates, Dermoptera, and Scandentia. Gross dimensions of the bony labyrinths of Euarchontoglires are provided in Table 1. Dimensions of the cochlea are provided in Table 2, and dimensions and orientations of the semicircular canals are reported in Tables 3–5.

The states reconstructed for the bony labyrinth of the most recent common ancestor of Euarchontoglires are the same as those for Boreoeutheria. That is, the lateral semicircular canal opens into the vestibule directly in the absence of a secondary common crus, the lateral semicircular canal is positioned high compared to the posterior semicircular canal, and the anterior canal arc is the largest in terms of radius of curvature among the three arcs. The cochlea of the ancestral euarchontoglire coils 957°, which is over a quarter of a turn greater than that reconstructed for the ancestral boreoeutherian (815°), and the cochlea of Euarchontoglires contributes 53% of the total inner ear volume (retained from the ancestor of Boreoeutheria, which had a cochlea contributing 55%). An unequivocal state of the aspect ratio of the cochlea could not be reconstructed from the data provided here.

Recognition of a close relationship between rodents and lagomorphs can be traced back to the seminal classification of Linnaeus [131], in which he also included the rhinoceros (although before 1758, he restricted Glires to rodents and lagomorphs [192]). Monophyly of Glires persisted in several later classifications [99]–[100], [107], [193], but monophyly has not been free from controversy [193]–[195]. Both morphological [101], [194], [196]–[198] and molecular investigations [199]–[204] have either allied Rodentia or Lagomorpha with various other placental mammal taxa, or else rendered the groups within Glires paraphyletic with varying levels of robusticity. Despite the ambiguity of rodent and lagomorph affinites in earlier studies, a unified Glires is supported by many recent phylogenetic analyses [6], [66], [69], [98], [112], [141], [205]–[212].

The most recent common ancestor of Rodentia and Lagomorpha (Glires) retained a lateral semicircular canal that opened into the vestibule directly in absence of a secondary common crus from the most recent common ancestor of Placentalia, a position of the lateral canal high compared to the posterior canal from the ancestor of Boreoeutheria, and the highest arc radius of curvature measured for the anterior semicircular canal arc from the ancestor of Theria. Although the euarchontoglire ancestral state of the aspect ratio of the cochlea was equivocal, the ancestral glire possessed a cochlea with a high aspect ratio, which was shared with Scandentia among the members of Euarchontoglires. The cochlea of the ancestral Glires coiled 924°, and the cochlea contributed 55% of the total labyrinthine volume, which was inherited from the ancestral boreoeutherian.

Primates, dermopterans, and scandentians together form the clade Euarchonta [213]–[214]. However, the results of Bininda-Emonds and others [66] do not recover a monophyletic Euarchonta. Rather, Scandentia is placed in a polytomy with Glires and a Dermoptera plus Primates clade, which is referred to as Primatomorpha (Figure 2). The ancestral labyrinth of Primatomorpha retained an anterior semicircular canal with the greatest arc radius of curvature among the three canal arcs from the ancestral therian, a high position of the lateral semicircular canal from the ancestral boreoeutherian, and a direct vestibular entrance of the lateral semicircular canal from the ancestral placental. The aspect ratio of the cochlea was low for Primatomorpha, which is a unique state within Euarchontoglires.

#### Rodentia

Rodents make up the most speciose clade of mammals, contributing over 40% of all named extant mammal species [138]. The supertree presented here (Figure 2) depicts Rodentia as a natural clade, although rodent monophyly has been questioned. The results of some phylogenetic analyses based on molecular sequence data support the hypothesis that guinea pigs do not share a common ancestry with other rodent taxa, but rather with Primates and ungulates [215]–[219]. Despite these outdated analyses, the results of most if not all recent morphological [118], [208], [220] and molecular [98], [112], [221]–[226] analyses support a monophyletic Rodentia.

**Figure 54:**
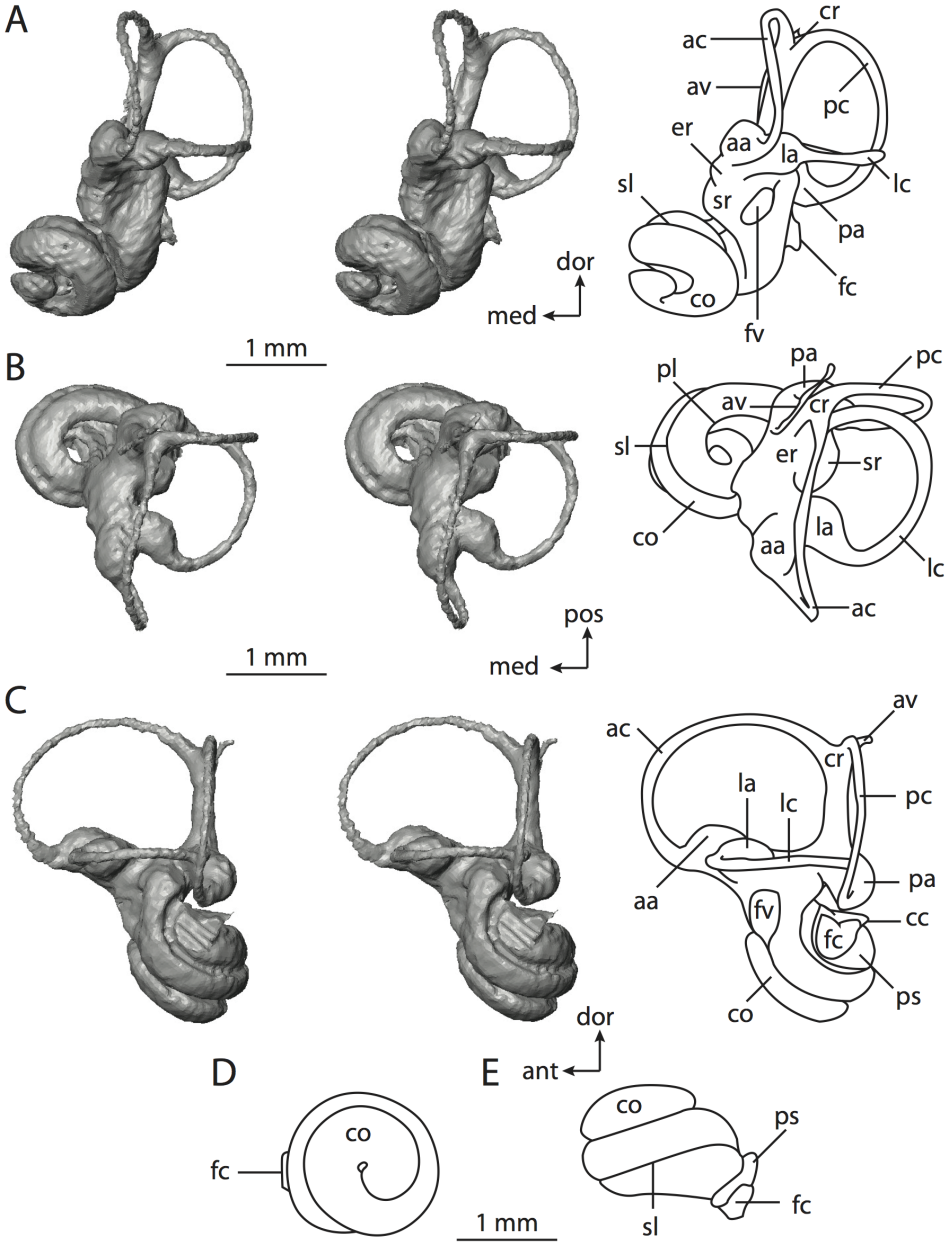
Bony labyrinth of*Mus musculus*. **A**, stereopair and labeled line drawing of digital endocast in anterior view; **B**, stereopair and labeled line drawing of digital endocast in dorsal view; **C**, stereopair and labeled line drawing of digital endocast in lateral view; **D**, line drawing of cochlea viewed down axis of rotation to display degree of coiling; **E**, line drawing of cochlea in profile. Abbreviations listed at the end of the Materials and Methods section.

**Figure 55:**
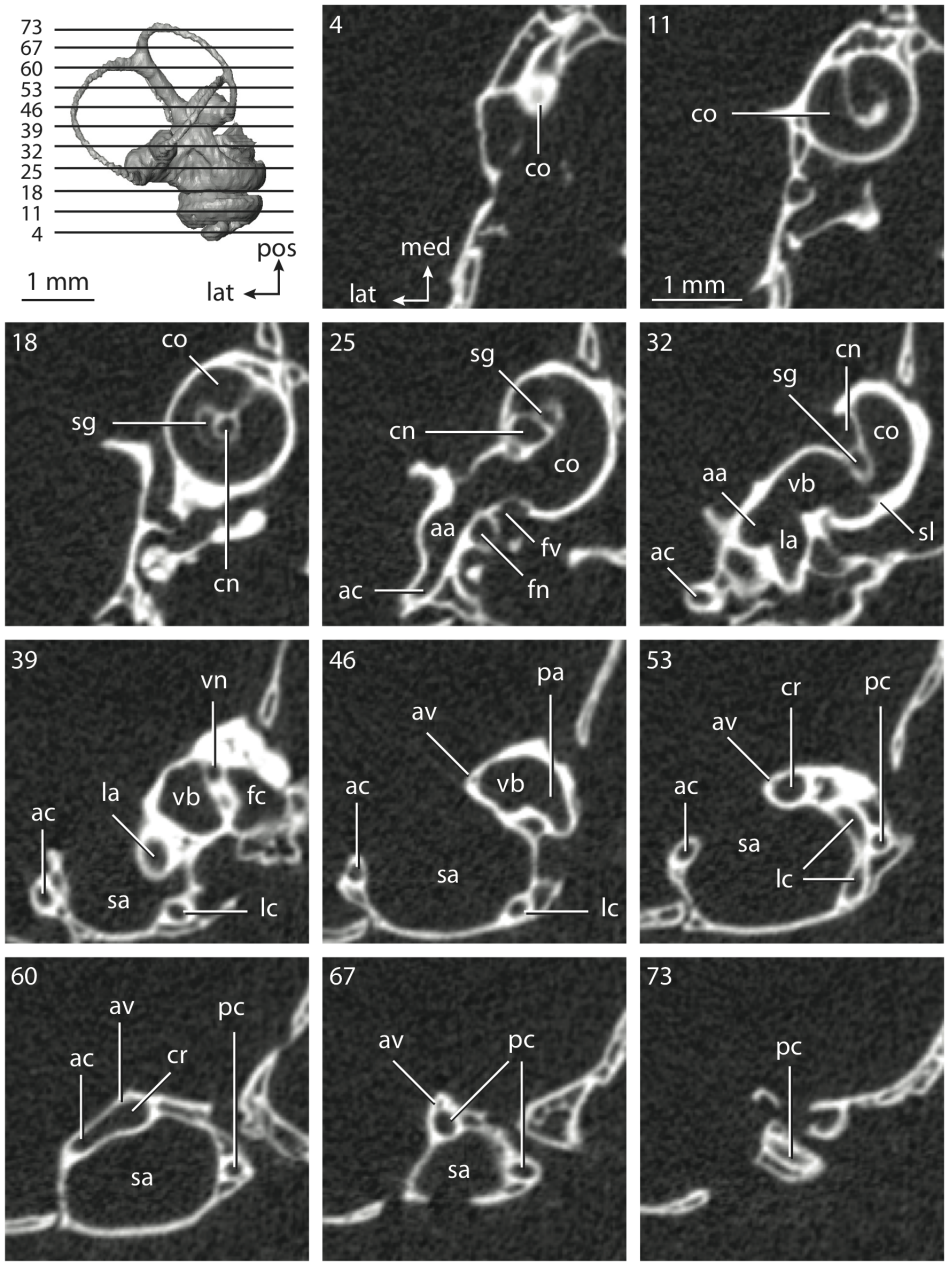
CT slices through ear region of*Mus musculus*. Abbreviations listed at the end of the Materials and Methods section.

**Figure 56:**
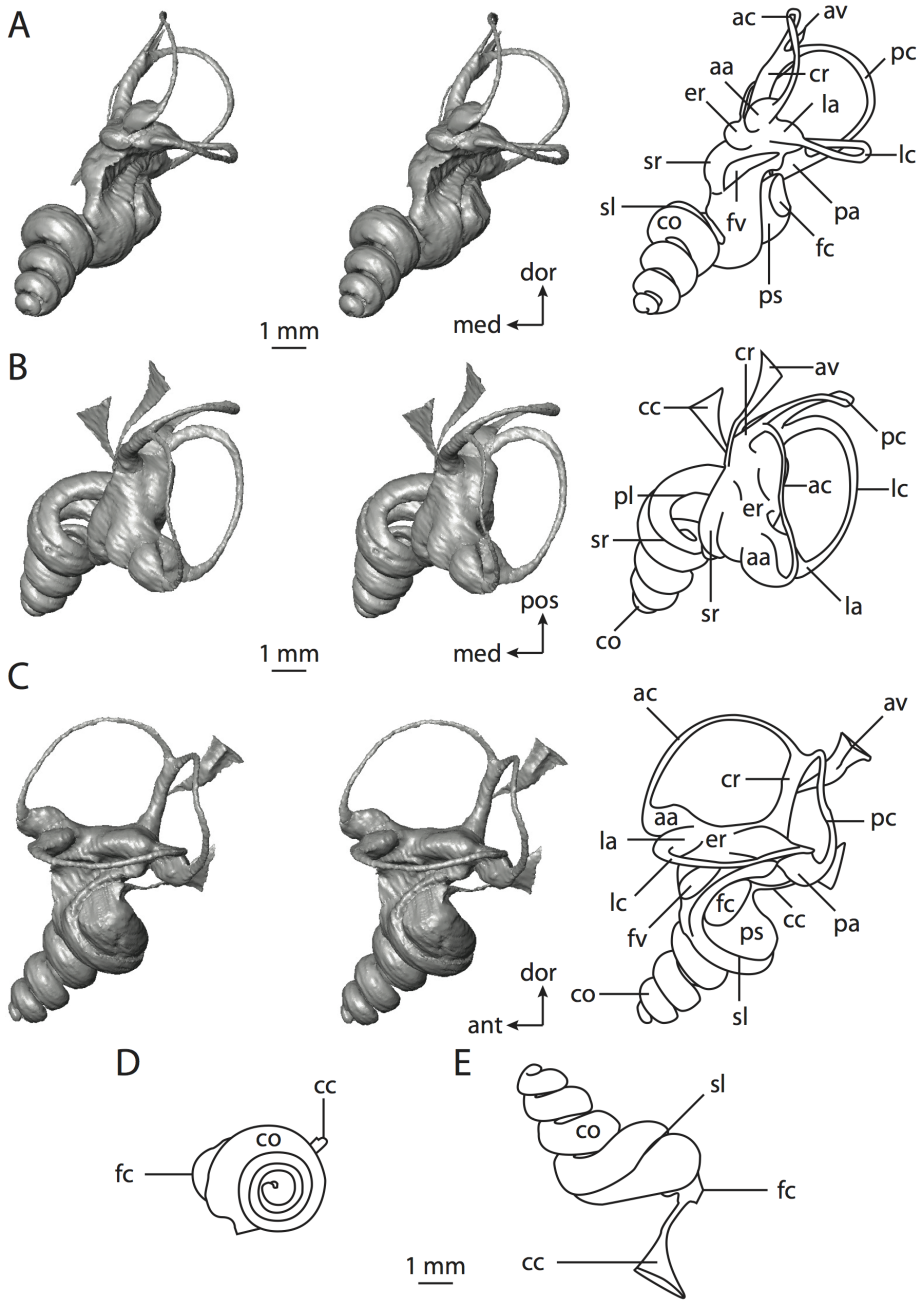
Bony labyrinth of*Cavia porcellus*. **A**, stereopair and labeled line drawing of digital endocast in anterior view; **B**, stereopair and labeled line drawing of digital endocast in dorsal view; **C**, stereopair and labeled line drawing of digital endocast in lateral view; **D**, line drawing of cochlea viewed down axis of rotation to display degree of coiling; **E**, line drawing of cochlea in profile. Abbreviations listed at the end of the Materials and Methods section.

**Figure 57:**
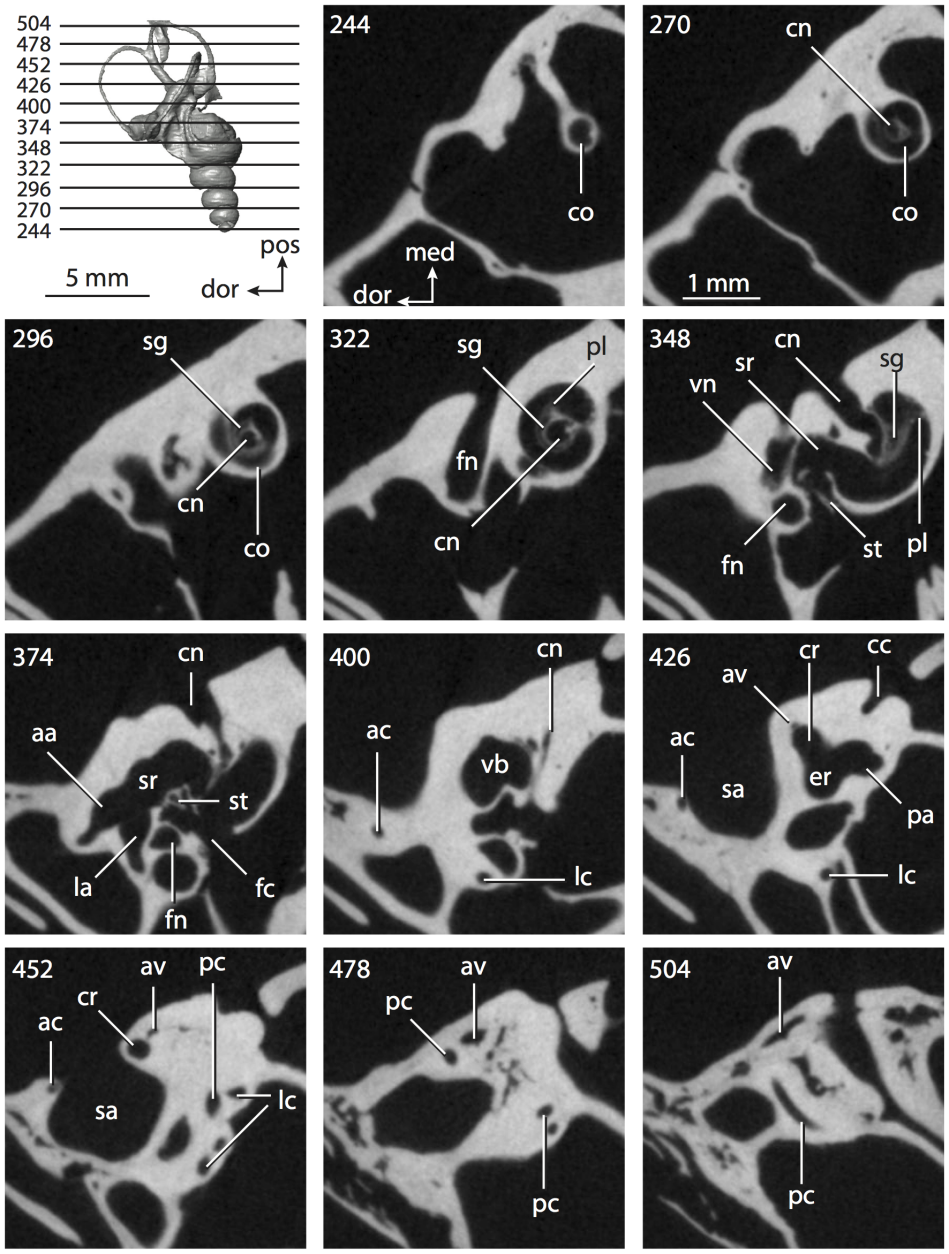
CT slices through ear region of*Cavia porcellus*. Abbreviations listed at the end of the Materials and Methods section.

The rodents examined in this study are the mouse *Mus musculus* (Figures 54–55) and the guinea pig *Cavia porcellus* (Figures 56–57). The bony labyrinth of *Cavia* is larger than that of *Mus*, both in terms of labyrinthine length and volume (Table 1). The dimensions of the inner ear cavities are mirrored by the average body masses of the two species [89]. The volume of the cochlea of *Cavia* is significantly greater than that measured for *Mus* (Table 2), although the cochlea forms a greater proportion of the bony labyrinth in the mouse (59%) than in the guinea pig (55%).

The most noticeable aspect of the bony labyrinth of *Cavia* is the sharp cone formed by the cochlea (Figure 56). Not only is the aspect ratio of the cochlea twice that observed in *Mus* (Table 2), the ratio of the guinea pig is greater than that calculated for any other mammal discussed here (closest is *Macroscelides*, which is still 60% of that calculated for *Cavia*). Similarly, the cochlea of *Cavia* coils to a much greater degree than any other mammal studied here (Figure 56D), completing over four whorls (Table 2). Even the highly coiled cochlea of *Procavia capensis* only coils three and three quarters turns (see Figure 16). The cochlea of *Mus* completes half as many coils (Figure 54D; Table 2).

The scala tympani is expanded interior to the fenestra cochleae in both taxa. The expansion leads to the canaliculus cochleae for the cochlear aqueduct. The canaliculus is a short and straight tube in *Mus* (cc in Figure 54C), but the slender portion of the canal of *Cavia* is gently curved and ends in a triangular shaped fissure (cc in Figure 56). The plane of the basal turn of the cochlea deviates from the plane of the lateral semicircular canal to a greater degree in *Cavia* than in *Mus* (Table 2).

The bony vestibule of *Mus* is not divided into the spherical and elliptical recesses, although an excavation at the anterior end of the vestibule, which is expressed as a bulbous pedestal in the endocast, for the posterior ampulla, common crus, and posterior limb of the lateral semicircular canal is present in the mouse (er in Figure 54B). The vestibule is subdivided into the spherical and elliptical recesses by a constriction interior to the fenestra vestibuli in *Cavia* (sr and er Figure 56A and B). The fenestra vestibuli is elliptical in the guinea pig (more circular in *Mus* equals; Table 2), which is expressed as an oblong depression in the spherical recess on the endocast. Both the spherical and elliptical recesses are elongate cavities.

Unlike *Mus*, the common crus and posterior ampulla of *Cavia* do not empty into a posterior extension of the vestibule (er in Figure 56B). The bony channel for the vestibular aqueduct exits the inner ear anterior to the medial edge of the vestibular aperture of the common crus in both taxa. The posterior limb of the lateral semicircular canal opens directly into the vestibule anterior to the posterior ampulla in both rodents. The plane of the lateral canal is positioned dorsal relative to the posterior semicircular canal, in both *Cavia* and *Mus* with similar sagittal labyrinthine indices (Table 3).

The semicircular canals are slender in all rodents examined here. The planes of the anterior and posterior semicircular canals in *Cavia* form an angle that is the greatest measured between any two canals measured for either rodent species (Table 3). The planes of the anterior and lateral semicircular canals in *Cavia* form an angle that is the smallest angle measured between any two canals measured for both rodents. The anterior semicircular canal is the largest in terms of arc radius of curvature and length of the slender portion of the canal for both rodents (Table 4), and the lateral canal is the smallest for the same dimensions. The cross-sectional diameter of the anterior semicircular canal of *Mus* is greater than the lateral and posterior canals, but the diameter of the lateral canal of *Cavia* is greater than the anterior and posterior semicircular canals (Table 4).

Both the largest and smallest semicircular canal arc aspect ratios among rodents were measured for the arcs of *Cavia* (Table 5). The largest semicircular canal arc aspect ratio in *Cavia* is observed in the posterior canal, and the smallest ratio is observed in the lateral semicircular canal for the guinea pig. The ratio of the slender portion of the posterior semicircular canal length to its arc radius is the greatest for both species (5.02 for *Cavia*; 5.39 for *Mus*), and the ratio is the smallest in the lateral canal (4.13 for *Cavia*; 4.12 for *Mus*). The canal length to arc radius ratio of the anterior semicircular canal is 4.79 for *Cavia* and 4.98 for *Mus*.

The semicircular canals of *Cavia* are less planar than the canals of *Mus* (Table 5), especially the posterior canal (ratio of total linear deviation over cross-sectional diameter is 3.13; 1.16 in *Mus*). The lateral semicircular canal of *Mus* is the most planar canal in either taxon (ratio is 0.13; 1.49 for *Cavia*). The linear deviation to cross-sectional diameter ratio is 0.31 for the anterior semicircular canal in *Cavia* and 3.10 for *Mus*.

The labyrinths of *Cavia* and *Mus* retain the ancestral condition reconstructed for Theria in that the largest semicircular arc radius is observed in the anterior canal. Further, the labyrinth of the ancestor of Rodentia retained the ancestral placental entry of the lateral canal (into the vestibule directly), the ancestral boreoeutherian position of the lateral semicircular canal (high compared to the posterior canal), and the ancestral glire cochlear aspect ratio (high). The cochlea of the rodent ancestor coiled 1003° (close to 1013° reconstructed for the most recent common ancestor of Cetacea plus *Sus*) and contributed 56% of the total labyrinthine volume (close to 55% contribution of the cochlea of Boreoeutheria).

#### Lagomorpha

Lagomorphs (hares, rabbits, and pikas) are classically allied with rodents (proposed as far back as 1748 [192]) and the majority of recent phylogenetic analyses support this hypothesis [6], [98], [112], [141], [208]. However, Lagomorpha has been united with ungulates [194], [196], [199], elephant shrews [101], and other placental clades [204]. Given the predominance of data supporting a clade exclusive to rodents and lagomorphs (Glires), such a relationship is accepted here.

**Figure 58:**
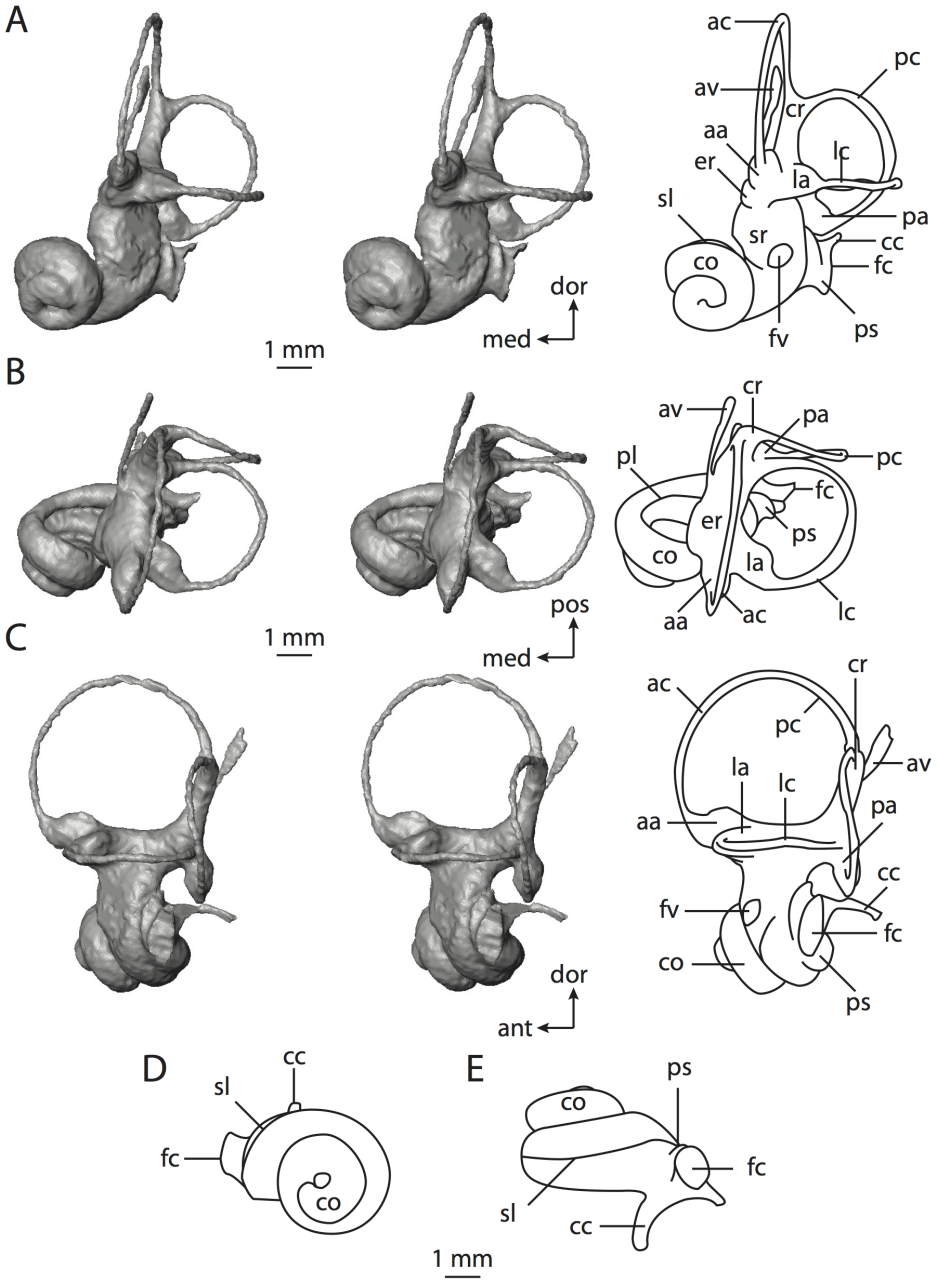
Bony labyrinth of*Lepus californicus*. **A**, stereopair and labeled line drawing of digital endocast in anterior view; **B**, stereopair and labeled line drawing of digital endocast in dorsal view; **C**, stereopair and labeled line drawing of digital endocast in lateral view; **D**, line drawing of cochlea viewed down axis of rotation to display degree of coiling; **E**, line drawing of cochlea in profile. Abbreviations listed at the end of the Materials and Methods section.

**Figure 59:**
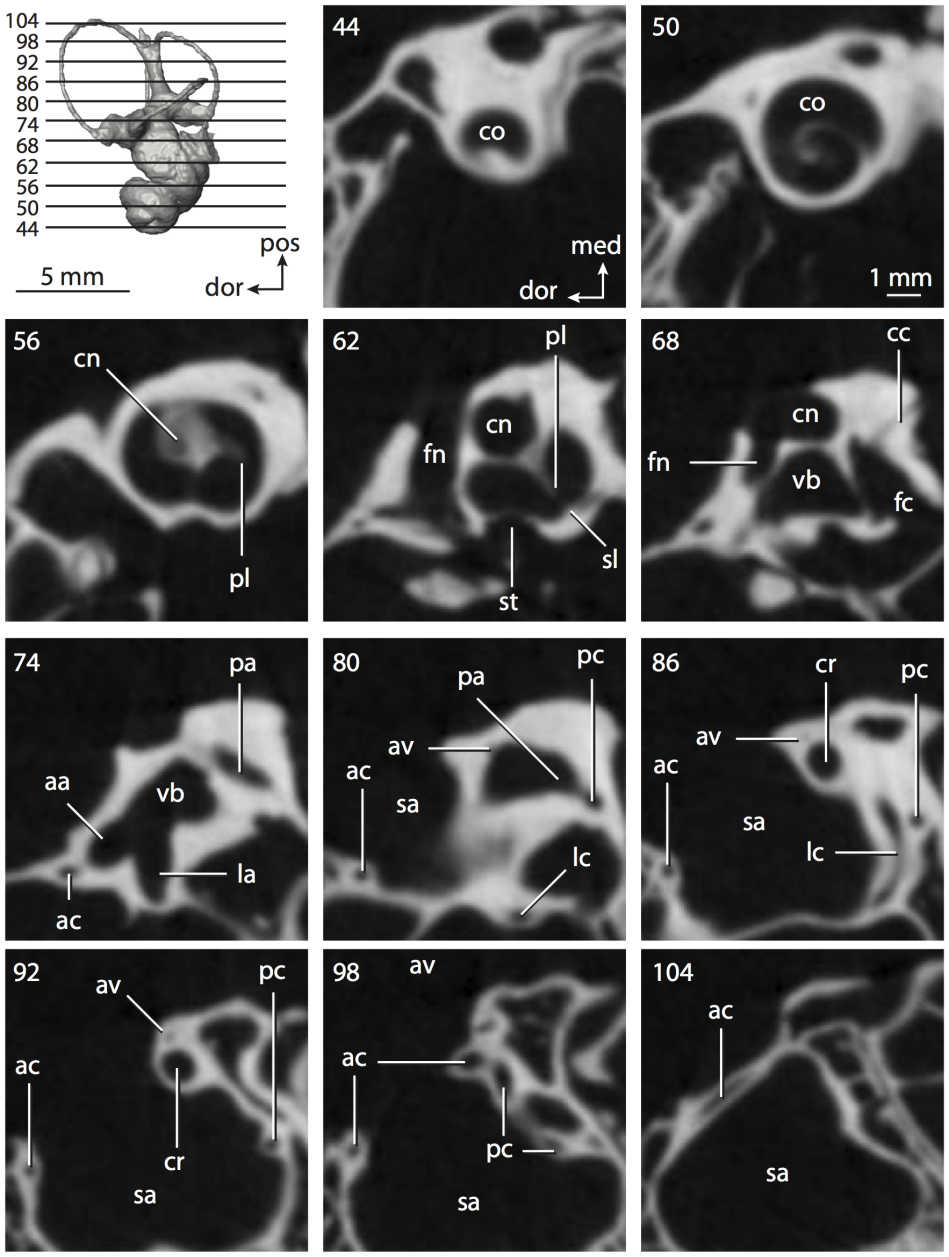
CT slices through ear region of*Lepus californicus*. Abbreviations listed at the end of the Materials and Methods section.

**Figure 60:**
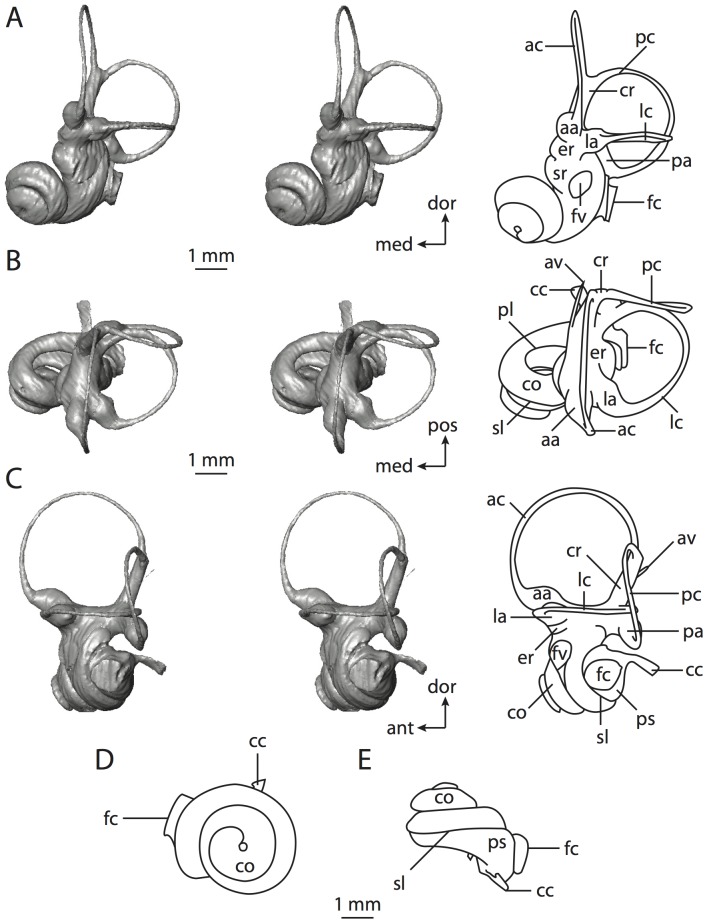
Bony labyrinth of*Sylvilagus floridanus*. **A**, stereopair and labeled line drawing of digital endocast in anterior view; **B**, stereopair and labeled line drawing of digital endocast in dorsal view; **C**, stereopair and labeled line drawing of digital endocast in lateral view; **D**, line drawing of cochlea viewed down axis of rotation to display degree of coiling; **E**, line drawing of cochlea in profile. Abbreviations listed at the end of the Materials and Methods section.

**Figure 61:**
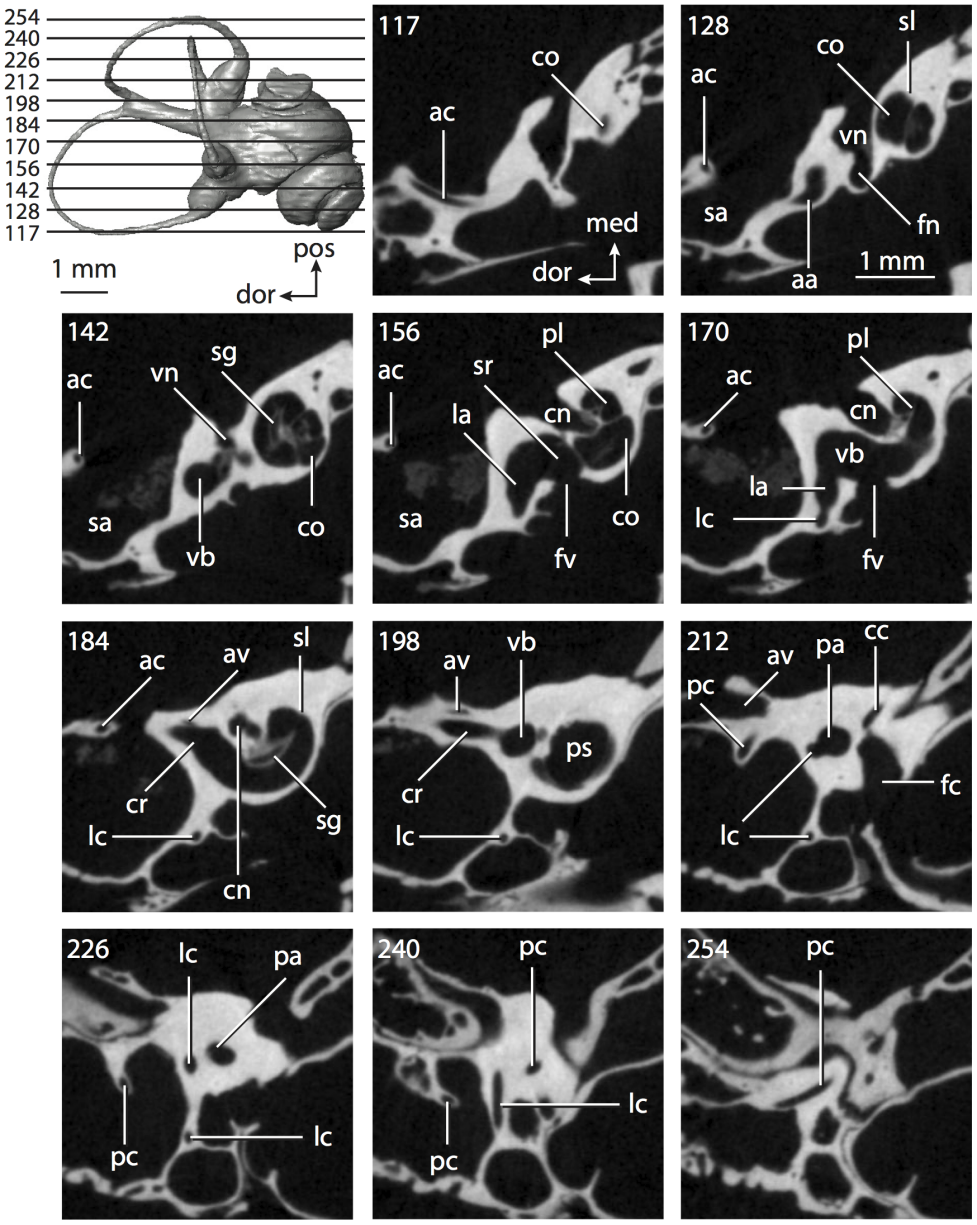
CT slices through ear region of*Sylvilagus floridanus*. Abbreviations listed at the end of the Materials and Methods section.

Two lagomorph species examined here were *Lepus californicus* (Figures 58–59) and *Sylvilagus floridanus* (Figures 60–61). The bony labyrinth of *Lepus* is the most voluminous and longest (Table 1), and the black-tailed jackrabbit (*Lepus*) is a larger species overall than the eastern cottontail (*Sylvilagus*) [89]. The volume of the cochlea of *Lepus* is over twice that measured for *Sylvilagus* (Table 2), although the relative contribution that the cochlea of each species to total labyrinthine volume is comparable between the species (54% for *Lepus*; 55% for *Sylvilagus*).

The length of the cochlear canal of *Lepus* is slightly larger than that of *Sylvilagus* (Table 2), although the cochlea of the cottontail (Figure 60D) coils to a greater degree than the jackrabbit (Figure 58). Likewise, the secondary bony lamina extends a greater relative distance along the radial wall of the cochlea in *Sylvilagus* than in *Lepus*, and the aspect ratio of the cochlea of *Sylvilagus* (Figure 60E) is greater than that calculated for *Lepus* (Figure 58E). The apical turns of the cochleae of both lagomorphs sit upon the basal whorl, as is observed in *Mus musculus* and *Cavia porcellus*, and the plane of the basal turn of the cochlea forms a similar angle with the plane of the lateral semicircular canal in both *Lepus* and *Sylvilagus* (Table 2). The scala tympani of the cochlea is expanded internal to the fenestra cochleae, which leads to a robust canaliculus cochleae in each species. The canaliculus of *Lepus* forms a straight tube that is subcircular in cross-section (cc in Figure 58), but the bony canal is flattened and curves ventrally in *Sylvilagus* (cc in Figure 60).

The fenestrae vestibuli are less elliptical in the lagomorphs than for the rodents (stapedial ratios in Table 3), but they are not as round as the fenestra of the microchiropteran bat *Nycteris grandis*. A gentle constriction of the vestibule divides the spherical and elliptical recesses in both *Lepus* and *Sylvilagus*, where the elliptical recesses of the lagomorphs are elongated with distinct excavations at the anterior and posterior ends (expressed as pedestals for the ampullae of the semicircular canals on the endocasts).

The bony channel for the vestibular aqueduct exits the inner ear cavities medial to the vestibular aperture of the common crus (av in Figure 59, slice 80 for *Lepus*; Figure 61, slice 184 for *Sylvilagus*). The channel is a delicate passage in *Sylvilagus*, and it does not end as a flattened fissure as in most other mammals, including *Lepus*. The channel is longer in *Lepus* than it is in *Sylvilagus*, both in terms of absolute length (Table 3) and length relative to the common crus (Table 2; channel terminates ventral to the apex of the common crus in *Sylvilagus*, but dorsal to the top of the crus in *Lepus*).

The lateral semicircular canal opens directly into the vestibule dorsal to the posterior ampulla in both lagomorphs (lc in Figures 59 and 61), giving the plane of the lateral canal a dorsal position in relation to the posterior semicircular canal (lc in Figures 58A and 60A). The level of the lateral semicircular canal compared to the posterior canal in the lagomorphs is similar to that observed in *Macroscelides proboscideus* (sagittal labyrinthine in Table 3; lc in Figure 12A), where the lateral canal divides the arc of the posterior canal when the labyrinth is viewed anteriorly.

The planes of the anterior and posterior semicircular canals form the greatest angle between canals in both *Lepus* and *Sylvilagus* (Table 3). The smallest angle between canals in *Lepus* was measured between the anterior and lateral semicircular canals, and the smallest angle measured within the labyrinth of *Sylvilagus* is between the posterior and lateral canals. The arc of the anterior semicircular canal not only has the greatest radius of curvature in both *Lepus* and *Sylvilagus*, but the length of the slender portion of the anterior semicircular canal in both *Lepus* and *Sylvilagus* is greater than either the lateral or posterior canal (Table 4). Likewise, the volume of the anterior semicircular canal of *Lepus* (0.32 mm^3^) is greater than either the lateral (0.25 mm^3^) or posterior canals (0.24 mm^3^), although the most voluminous canal within the labyrinth of *Sylvilagus* is the lateral semicircular canal (0.19 mm^3^ versus 0.16 mm^3^ for the anterior and 0.14 mm^3^ for the posterior canals). The cross-sectional diameter of the posterior semicircular canal of *Lepus* is greater than either the anterior or lateral semicircular canal (Table 4).

The aspect ratios of the anterior and posterior canals are greater in *Sylvilagus* than in *Lepus* (Table 5), but the ratio calculated for the arc of the lateral canal is greater in *Lepus* than *Sylvilagus*. As in the majority of mammals described so far, the ratio of the length of the slender portion of the posterior semicircular canal to its arc radius in *Sylvilagus* (5.13) is greater than that computed for the anterior (4.84) and lateral semicircular canals (4.38). However, the greatest ratio among the canals of *Lepus* was calculated for the anterior semicircular canal (4.89; 4.13 for the lateral canal; 4.80 for the posterior canal).

The posterior semicircular canal is the least planar canal in both taxa (Table 5), where the canal of *Sylvilagus* deviates from its plane to a greater degree than that of *Lepus*. The posterior canal of *Sylvilagus* deviates to a substantial degree (ratio of linear deviation over cross-sectional diameter is 3.79), as does the posterior canal of *Lepus* (ratio is 1.09). The lateral semicircular canal of *Lepus* is the most planar among all of the canals between the two species, but the linear deviation is not substantial for either species (ratio of 0.24 for *Lepus*; 0.51 for *Sylvilagus*). The anterior semicircular canal deviates from its average plane by a lesser degree in *Lepus*, and only the anterior canal of *Sylvilagus* deviates to a substantial degree (ratio is 1.28; 0.59 for *Lepus*).

There are no unambiguous synapomorphies that support monophyly of Lagomorpha within Glires or Euarchontoglires. The states reconstructed for the ancestor of Lagomorpha are the same as those for both Rodentia and Glires, as the lagomorphs retain the ancestral therian condition of the largest radius of curvature measured for the anterior semicircular canal arc, the placental condition of the direct vestibular entrance of the lateral semicircular canal, the ancestral boreoeutherian condition of the high position of the lateral semicircular canal compared to the ampullar opening of the posterior canal, and the glire condition of the high aspect ratio of the cochlea. The cochlea of the most recent common ancestor of lagomorphs coils 751° and contributes 53% to the total volume of the inner ear cavities.

#### Primates

Primates consists of two major lineages, Strepsirhini which includes the lemurs and lorises, and Haplorhini which includes monkeys and apes. The haplorhines are divided further into three groups, which are Tarsiidae (tarsiers), Platyrhini (New World monkeys), and Catarhini (Old World monkeys and apes). Monophyly of all of these clades is supported by numerous phylogenetic analyses [66], [227]–[231].

**Figure 62:**
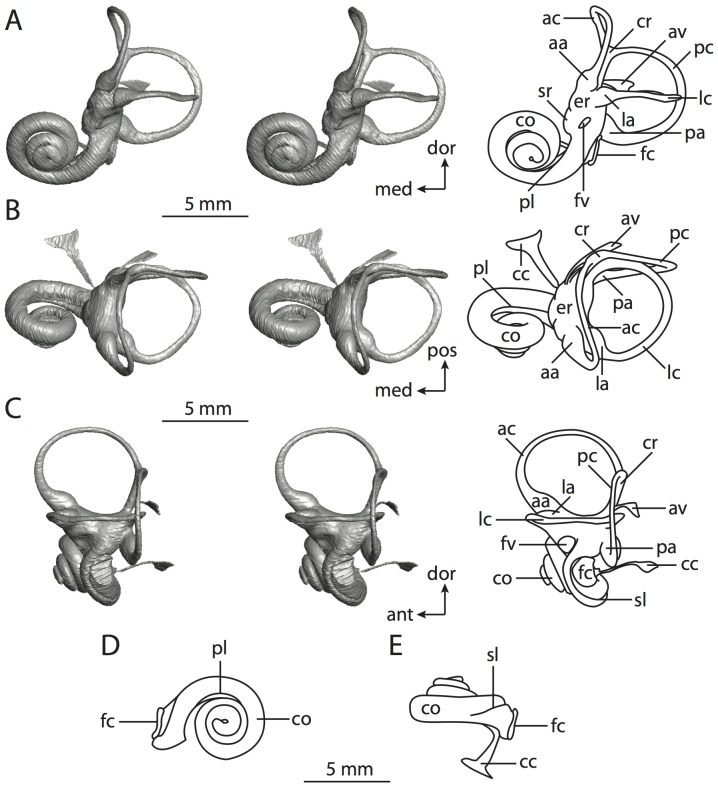
Bony labyrinth of*Macaca mulatta* (images reversed). **A**, stereopair and labeled line drawing of digital endocast in anterior view; **B**, stereopair and labeled line drawing of digital endocast in dorsal view; **C**, stereopair and labeled line drawing of digital endocast in lateral view; **D**, line drawing of cochlea viewed down axis of rotation to display degree of coiling; **E**, line drawing of cochlea in profile. Abbreviations listed at the end of the Materials and Methods section.

**Figure 63:**
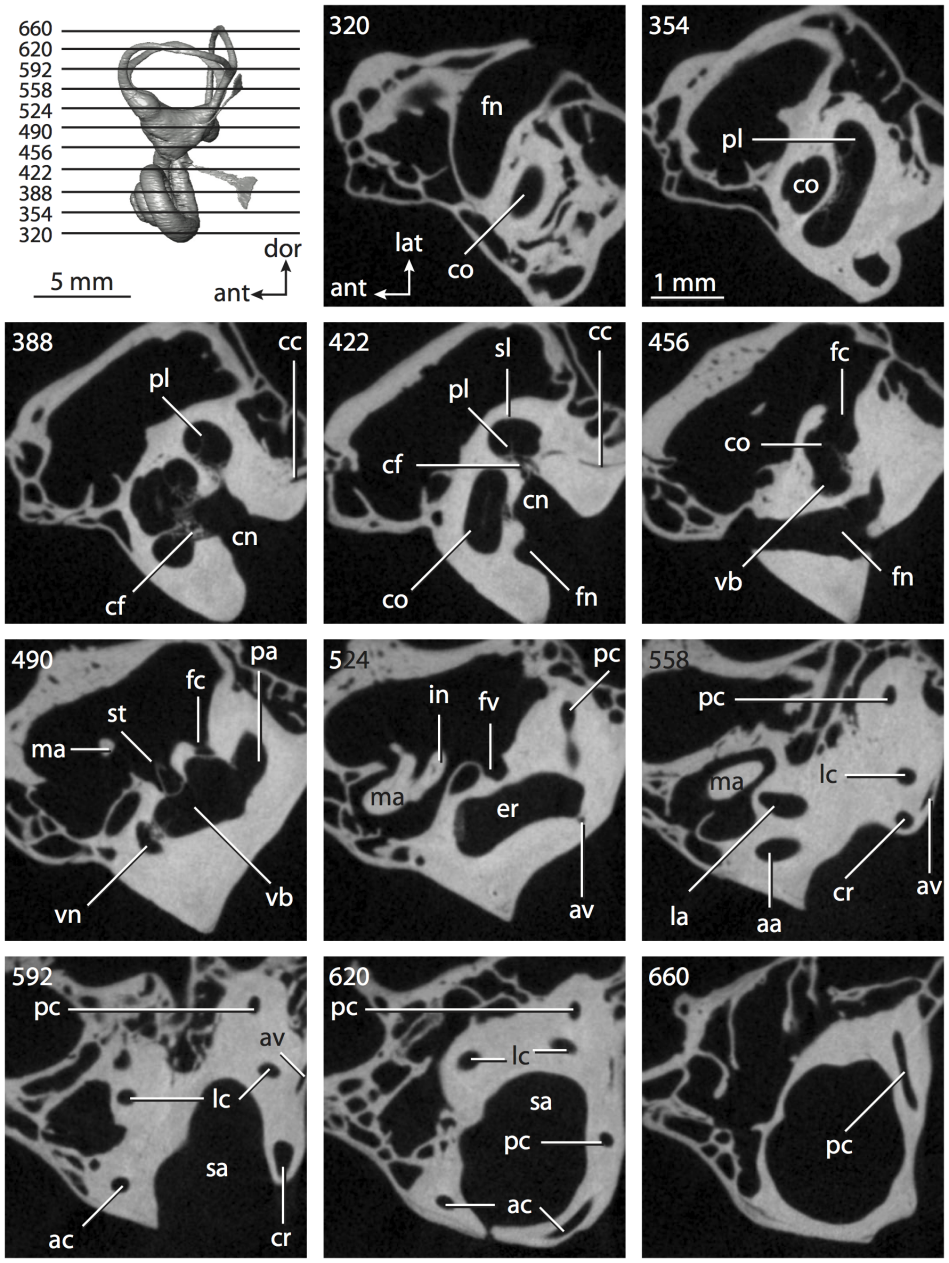
CT slices through ear region of*Macaca mulatta*. Abbreviations listed at the end of the Materials and Methods section.

**Figure 64:**
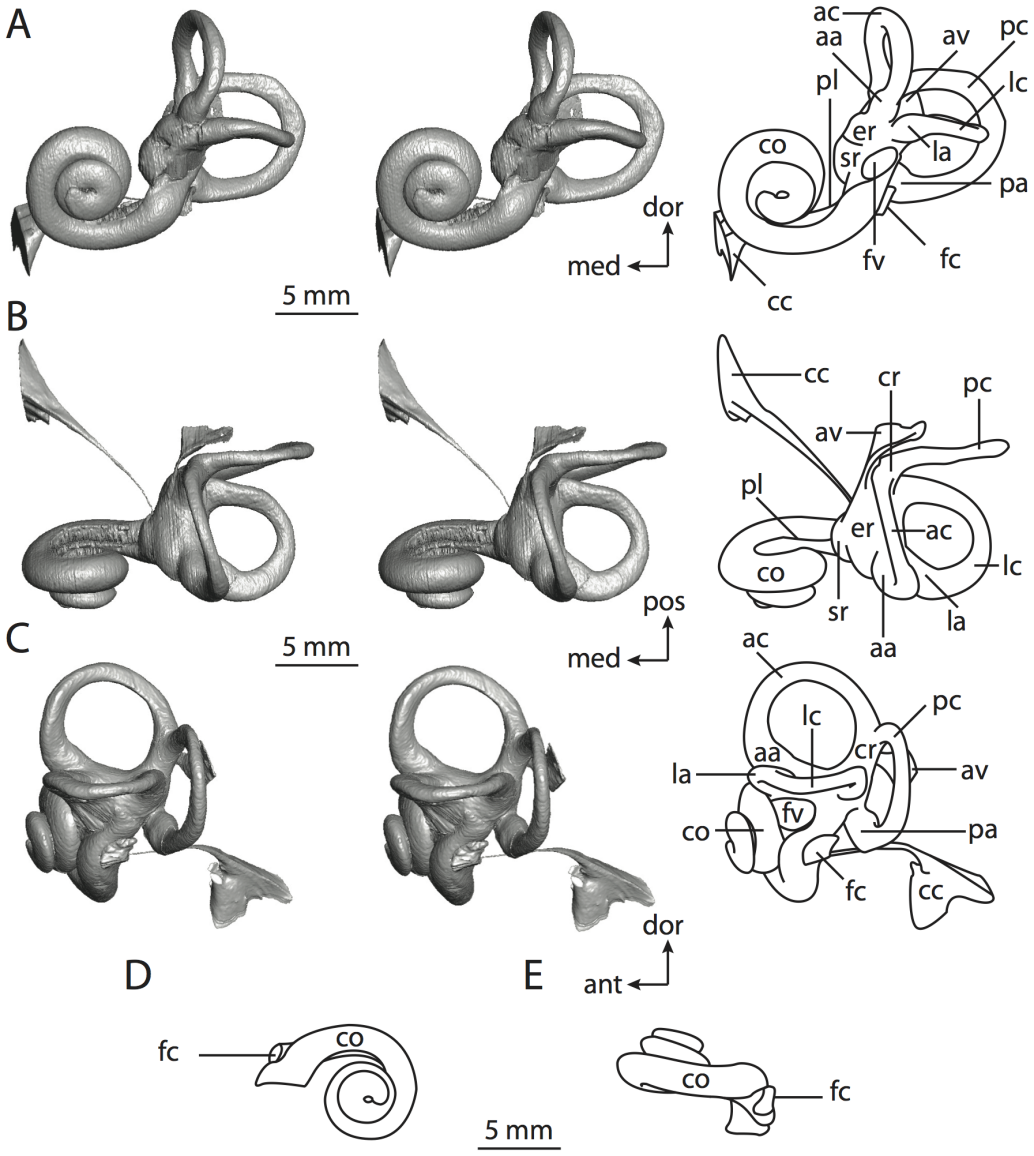
Bony labyrinth of*Homo sapiens* (images reversed). **A**, stereopair and labeled line drawing of digital endocast in anterior view; **B**, stereopair and labeled line drawing of digital endocast in dorsal view; **C**, stereopair and labeled line drawing of digital endocast in lateral view; **D**, line drawing of cochlea viewed down axis of rotation to display degree of coiling; **E**, line drawing of cochlea in profile. Abbreviations listed at the end of the Materials and Methods section.

**Figure 65:**
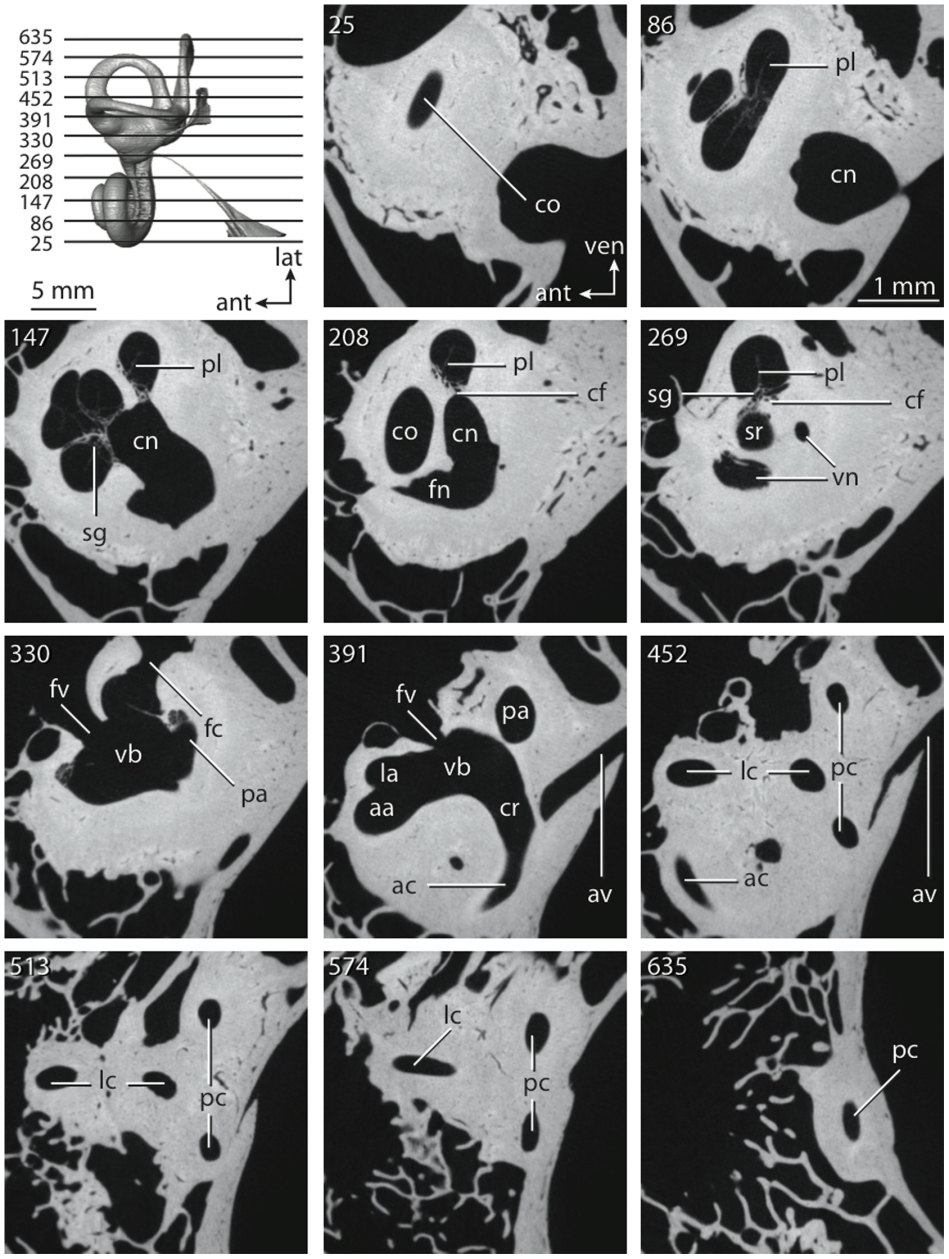
CT slices through ear region of*Homo sapiens*. Abbreviations listed at the end of the Materials and Methods section.

The two primate species examined here are the rhesus monkey, *Macaca mulatta* (Figures 62–63), and the human, *Homo sapiens* (Figures 64–65). The average body mass of adult humans (74–86 kg) [91] is significantly greater than that of rhesus monkeys (4.7 kg) [89], and this pattern is mirrored by the volume and length of the bony labyrinth (Table 1). The human cochlea is larger than that of *Macaca* in absolute volume and canal length (Table 2), but the cochlea of *Homo* contributes a lesser amount to the entire bony labyrinth than does the cochlear cavity of *Macaca* (50% and 43% respectively). Only the cochlea of the elephantimorph proboscidean contributes less (31%) to the bony labyrinth among the mammal species discussed so far.

The cochlea of *Macaca* completes a greater degree of coiling than the cochlea of *Homo* (Table 2; Figures 62D and 64D), and the secondary bony lamina persists to a greater relative distance in the rhesus monkey than the human (sl in Figure 63). The aspect ratios of the cochlea in profile for *Macaca* and *Homo* are low (Table 2). The scala tympani is expanded internal to the fenestra cochleae. The canaliculus cochleae for the cochlear aqueduct exits the inner ear from this excavation, and the canaliculus forms a long tunnel ending in a triangular cavity in *Homo*. The canaliculus forms a flattened and outwardly flaring passage in *Macaca*.

The apical turns of the cochlea are separated from the basal whorl (fitting inside of the basal whorl when the cochlea is viewed down its axis of rotation), and the apical turns sit on top of one another (Figures 62D and 64D). A difference between the angle formed by the planes of the basal turn of the cochlea and lateral semicircular canal was measured between the primate species, where the angle was much larger in *Homo* than measured in *Macaca* (Table 2). The angle between the cochlea and lateral canal of *Macaca* is similar to that observed in the elephantimorph proboscidean, *Procavia*, and *Felis*, but the angle in *Homo* is greater than that in any other mammal (Table 2).

The fenestrae vestibuli of the primates are among the most elliptical fenestrae among the mammals examined here (stapedial ratio in Table 3), similar to *Cavia porcellus*. The vestibule is constricted internal to the fenestra vestibuli, thereby defining the border between the spherical and elliptical recesses. The bony channel for the vestibular aqueduct leaves the inner ear dorsal to the medial edge of the common crus and terminates as a fissure in both species (av in Figures 63, slices 524–592 and 65, slices 391–452), and the channel is robust in *Homo.* The semicircular canals of *Homo* are relatively thick compared to the canals of *Macaca* (Figures 62 and 64), and the common crus of *Homo* is short and stout, similar to the crus in *Manis tricuspis* (cr in Figure 40).

The greatest angle between the planes of any two semicircular canals in primates was measured between the anterior and posterior semicircular canals (Table 4). The planes of the anterior and lateral semicircular canals of *Homo* form a similar angle, and the angle between the posterior and lateral semicircular canals is nearly 90° (Table 4). The posterior limb of the lateral semicircular canal opens directly into the vestibule, nearly equidistant between the vestibular apertures of the common crus and posterior ampulla, in both primate species (lc in Figures 62A and 64A). The sagittal labyrinthine indices of *Macaca* and *Homo* are greater than that calculated for any other mammal discussed here (Table 3). The closest non-primate to approach this level is *Procavia*.

The posterior semicircular canal of *Homo* is the largest in all dimensions explored in this study, including the arc radius of curvature, for which the anterior canal has the greatest value in most mammals (Table 4). In fact, the radius of the anterior semicircular canal is greater than either the lateral or posterior canal in *Macaca*. The length of the slender portions of the posterior semicircular canals of both *Homo* and *Macaca* are greater than either the anterior or lateral canals (Table 4). The posterior semicircular canal of *Homo* has a cross-sectional diameter that is over twice as large as the diameter measured in *Macaca*.

The aspect ratios of the arcs of the semicircular canals are similar between the two primate taxa (Table 5). The highest aspect ratio in each species was calculated for the posterior canal arc, in which the heights and widths of the canal arcs are nearly identical. The ratio of the length of the slender portion of the posterior semicircular canal to its arc radius is larger than the other canals in both *Macaca* (5.13; 4.74 for anterior; 4.29 for lateral) and *Homo* (4.76; 4.61 for anterior; 4.39 for lateral).

The anterior semicircular canal is the least planar canal in each primate (Table 5). The total deviation of the lateral semicircular canal in *Macaca* is less than that measured for the posterior canal, and the posterior semicircular canal is the most planar within the labyrinth of *Homo*. The deviation of the anterior canal is substantial for both primates (ratio of total linear deviation to cross-sectional diameter is 1.08 for *Homo*; 3.75 for *Macaca*), but only the posterior canal of *Macaca* deviates substantially (ratio is 1.11; 0.6 for *Homo*). The degree of deviation of the lateral semicircular canal is not substantial in either species (ratio is 0.34 for *Homo*; 0.66 for *Macaca*).

There are no unambiguous synapomorphies in the bony labyrinth to support monophyly of Primates, and the clade retains the ancestral primatomorphan morphology of the cochlear spiral in that the cochlea has a low aspect ratio in profile. The anterior semicircular canal arc has the largest radius of curvature, which is retained from the ancestor to Theria, although the greatest radius in *Homo* was measured for the posterior canal arc. The arc of the posterior semicircular canal of no other euarchontoglire is the largest in terms of radius of curvature, and the only mammals for which the posterior canal arc is the greatest are *Manis* (only member of Laurasiatheria with the posterior canal the greatest), *Dasypus* (the distribution within Xenarthra beyond this taxon is unknown), and *Orycteropus* and *Procavia* among afrotherians.

The ancestral primate retained the ancestral placental condition of the direct vestibular entrance of lateral semicircular canals in the absence of a secondary common crus, and the plane of the lateral canal was high relative to the ampullar entrance of the posterior canal, which was retained from the ancestor of Boreoeutheria, if not earlier (state is equivocal for Placentalia). The cochlea of the ancestor of Primates coiled 980°, and the cochlea contributed 48% of the total labyrinthine volume, which is the same value as that reconstructed for Paenungulata, but slightly less than that for Primatomorpha (50%).

#### Dermoptera

The colugos are gliding mammals divided into two extant species, *Cynocephalus volans* and *Galeopterus variegatus* [137], and the bony labyrinth of *Cynocephalus* is used as a representative of Dermoptera. Phylogenetic analyses based on molecular data reconstruct a close relationship between Primates and Dermoptera [66], [231]–[232], with the occasional result of Dermoptera nested within Primates [112], [233].

**Figure 66:**
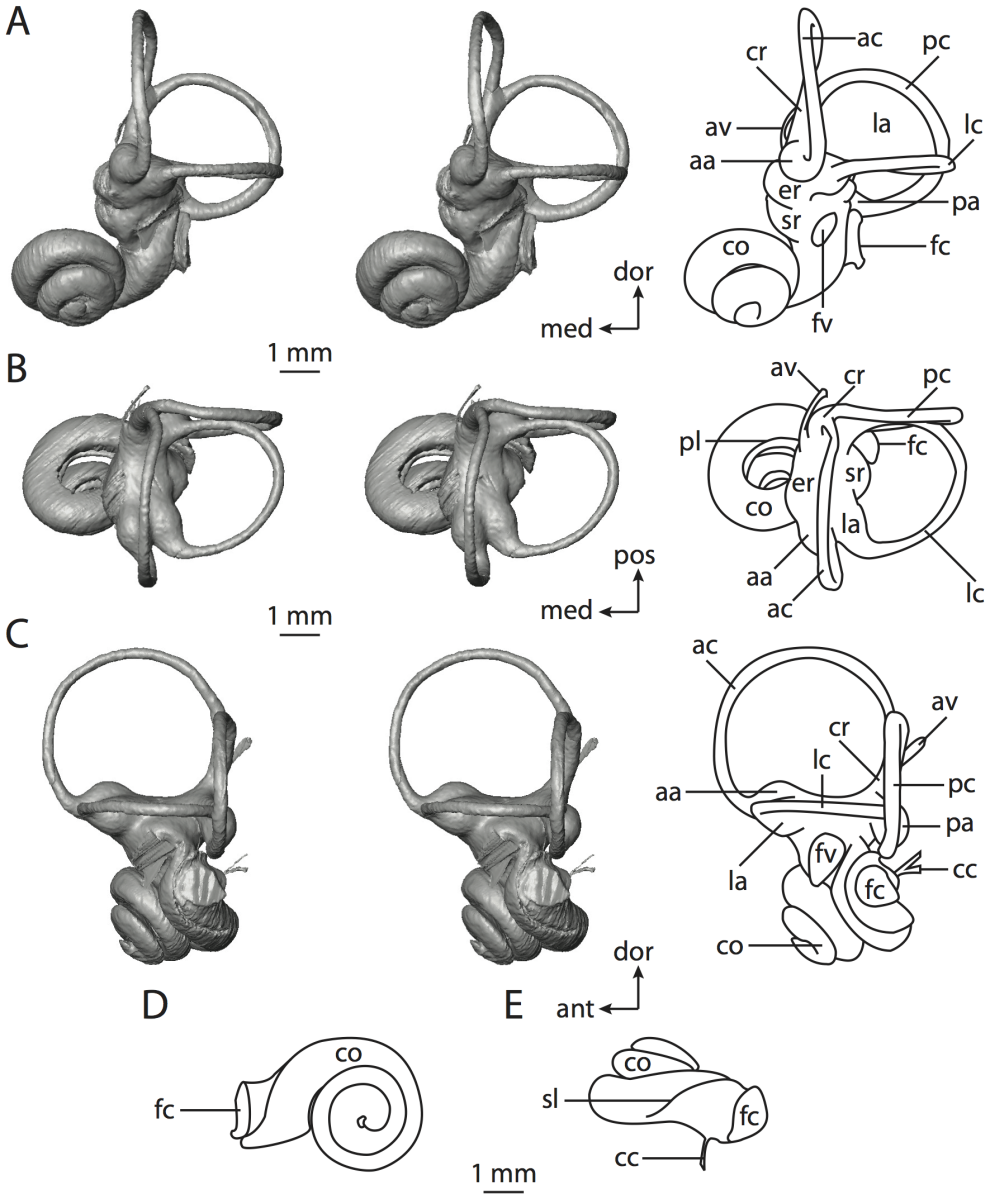
Bony labyrinth of*Cynocephalus volans*. **A**, stereopair and labeled line drawing of digital endocast in anterior view; **B**, stereopair and labeled line drawing of digital endocast in dorsal view; **C**, stereopair and labeled line drawing of digital endocast in lateral view; **D**, line drawing of cochlea viewed down axis of rotation to display degree of coiling; **E**, line drawing of cochlea in profile. Abbreviations listed at the end of the Materials and Methods section.

**Figure 67:**
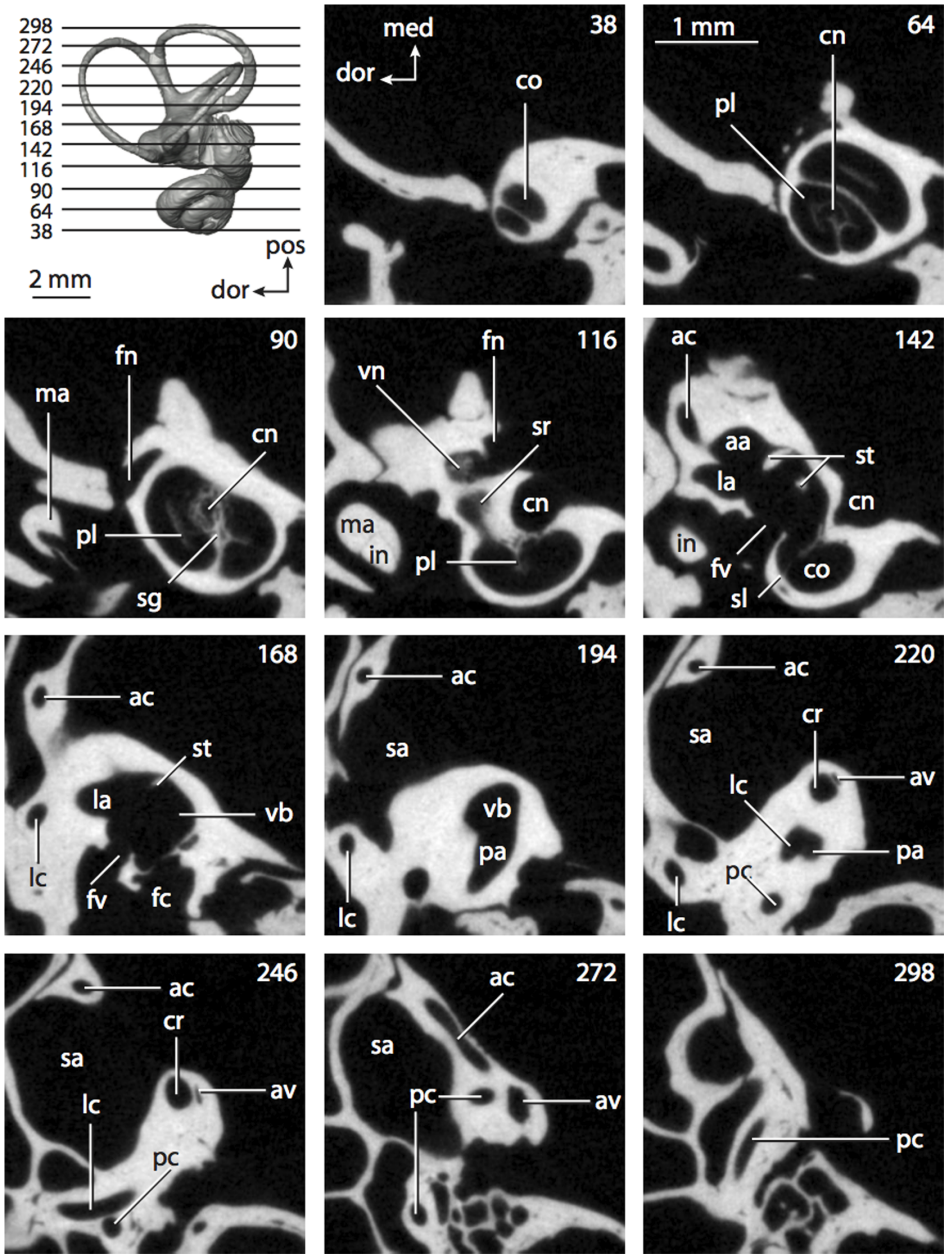
CT slices through ear region of*Cynocephalus volans*. Abbreviations listed at the end of the Materials and Methods section.

Although the average body mass of *Cynocephalus* is less than the rabbit *Sylvilagus floridanus* [89], the dimensions of the inner ear of the colugo are greater than that for the rabbit (Table 1). The cochlear canal of *Cynocephalus* contributes 48% of the total labyrinthine volume, which is similar to the contribution calculated for *Homo sapiens* (50%). The cochlear spiral completes nearly two and two thirds whorls (Table 2), and the secondary bony lamina persists for around one fifth of the basal turn of the cochlea, as is illustrated in Figures 66–67. The apical turns of the cochlea fit within the basal coils when the cochlea is viewed down its axis of rotation (Figure 66D). The bony canaliculus cochleae is developed as a delicate tube that curves along its course. A second channel, which likely carried a blood vessel in life, extends away from the bony labyrinth alongside the canaliculus cochleae (cc in Figure 66C).

The fenestra vestibuli is elliptical (identical stapedial ratio as calculated for *Tadarida brasiliensis*; Table 3), and a constriction of the vestibule internal to the fenestra vestibuli can be used to distinguish between the spherical and elliptical recesses (sr and er in Figure 66A). The ampullae are very round in *Cynocephalus*, and the posterior limb of the lateral semicircular canal opens directly into the vestibule immediately dorsal to the vestibular aperture of the posterior ampulla (lc in Figures 66B and 67, slice 220). The bony channel for the vestibular aqueduct is a straight tube that exits the inner ear cavities medial to the vestibular aperture of the common crus (Figures 66B and 67, slice 220).

The planes of the anterior and posterior semicircular canals of *Cynocephalus* form a 90° angle with each other, and the other angles between semicircular canals are not far off (Table 3; Figure 66). The anterior semicircular canal is the largest in terms of arc radius and length of the slender portion of the canal (Table 4). This pattern is observed in most of the mammals considered for this study. However, the lateral semicircular canal is the largest in terms of cross-sectional diameter. The arcs of the semicircular canals are circular, particularly the arc of the posterior canal (Table 5). The ratio of the length of the slender portion of the anterior semicircular canal to the arc radius of *Cynocephalus* (5.15) is greatest among the canals (ratio of lateral equals 4.75; ratio of posterior equals 4.94), which is different than the condition in most of the mammals examined here, where the greatest ratio is observed in the posterior semicircular canal. The anterior canal is the least planar of the three semicircular canals (Table 5). Likewise, the anterior canal deviates to a substantial degree (ratio of total linear deviation over cross-sectional canal diameter is 1.64), but the deviations of the lateral and posterior canals are not substantial (ratios are 0.24 and 0.28 respectively).

The bony labyrinth of *Cynocephalus* retains all states reconstructed in the most recent common ancestor of Primatomorpha (Primates plus Dermoptera). The aspect ratio of the cochlea is low (retained from Primatomorpha), the lateral semicircular canal is high compared to the ampullar opening of the posterior semicircular canal (retained from Boreoeutheria), the lateral canal opens into the vestibule directly in the absence of a secondary common crus (retained from Placentalia), and the greatest arc radius of curvature was measured for the anterior semicircular canal (retained from Theria). The contribution of the cochlea calculated for *Cynocephalus* (48%) is retained from the ancestor of Primatomorpha (50%), and the coiling of the cochlea (954°) is similar to that reconstructed for the ancestor of Euarchontoglires (957°).

#### Scandentia

**Figure 68:**
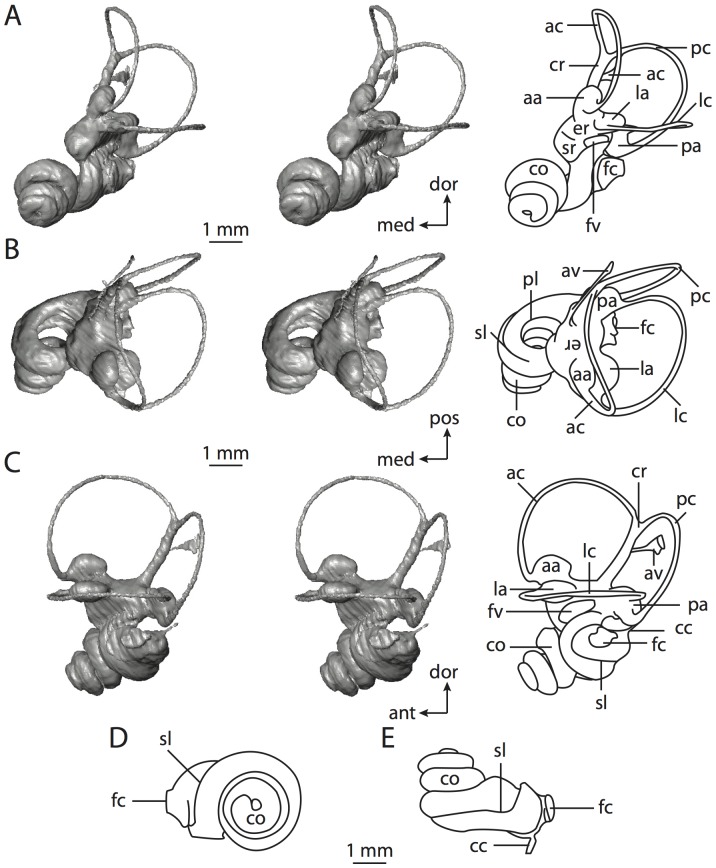
Bony labyrinth of*Tupaia glis.* **A**, stereopair and labeled line drawing of digital endocast in anterior view; **B**, stereopair and labeled line drawing of digital endocast in dorsal view; **C**, stereopair and labeled line drawing of digital endocast in lateral view; **D**, line drawing of cochlea viewed down axis of rotation to display degree of coiling; **E**, line drawing of cochlea in profile. Abbreviations listed at the end of the Materials and Methods section.

**Figure 69:**
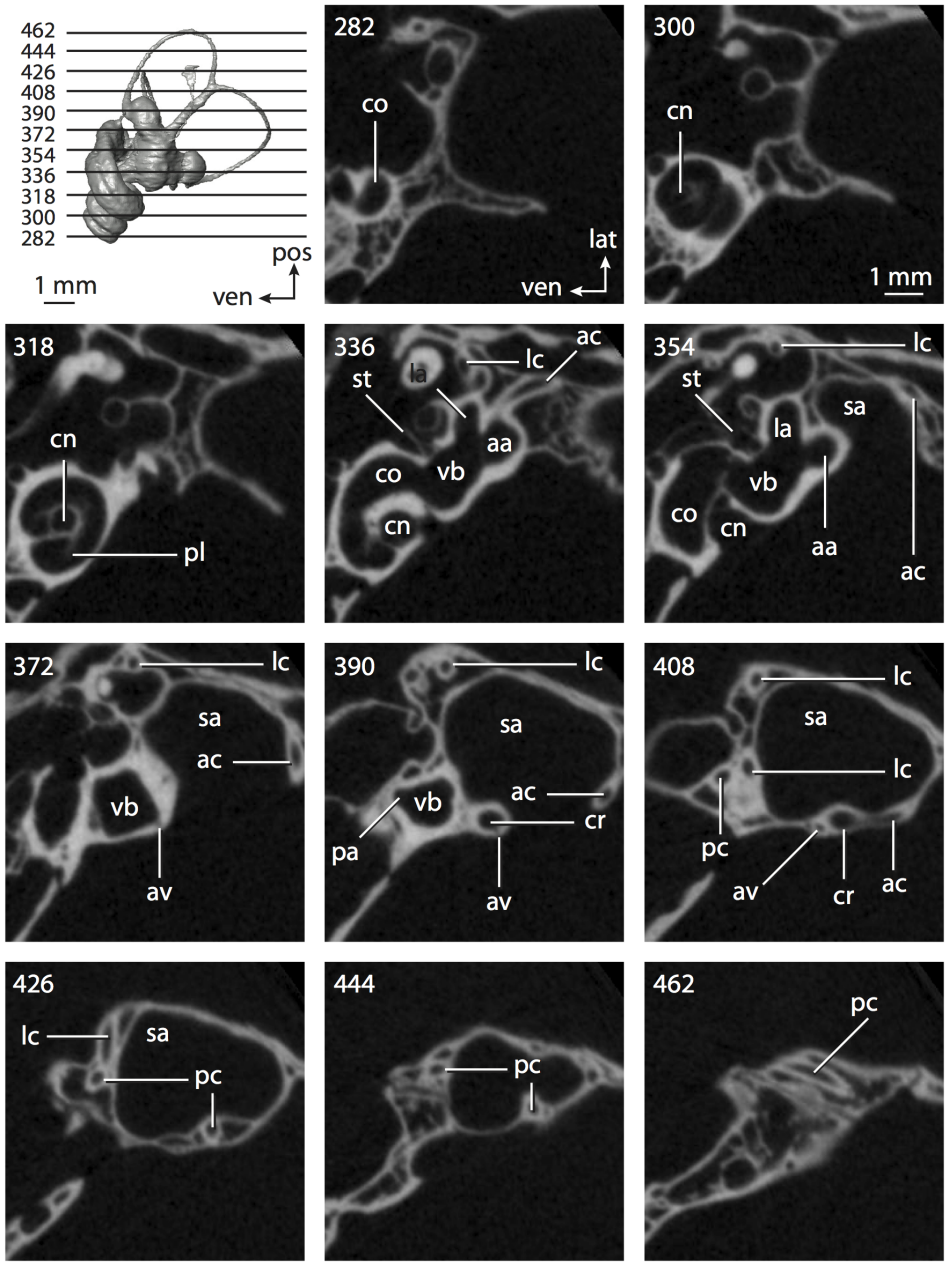
CT slices through ear region of*Tupaia glis.* Abbreviations listed at the end of the Materials and Methods section.

The final species to be considered here is the tree shrew, *Tupaia glis* (Figures 68–69). Scandentians were considered to have “insectivoran” affinities in early classifications [193], as well as close associations with Macroscelidea [99]. The results of later studies have been used to remove tree shrews from Lipotyphla and to postulate a closer relationship between Scandentia and Primates, at times with tree shrews included within Primates [100], [234]. Most mammalian systematists today agree that Scandentia is a clade exclusive of Primates [235]–[237], and the majority of anatomical and molecular evidence supports the monophyly of Euarchonta (Primates, Dermoptera, Scandentia), even if the relationships within the clade remain unresolved [66], [98], [112], [238]–[239].

The overall anterior-posterior length of the bony labyrinth of *Tupaia* is similar to that measured for the dermopteran *Cynocephalus volans* (Table 1), despite a body mass of *Cynocephalus* that is one order of magnitude larger than that of *Tupaia* [89]. The cochlea of *Tupaia* contributes 55% of the total inner ear volume and completes over three turns (Table 2; Figure 68D), and the secondary bony lamina extends beyond half of the basal coil (sl in Figure 68B and C). The aspect ratio of the cochlear spiral in profile is among the highest calculated among the mammal sample (Table 2; Figure 68E). The scala tympani of the cochlea is expanded internal to the fenestra cochleae, from which the canaliculus cochleae exits the cochlea. The canaliculus is a straight tube that extends posterodorsally from the scala tympani (cc in Figure 68C).

The shape of the fenestra vestibuli of *Tupaia* is elliptical, with a stapedial (aspect) ratio of similar to the rhesus monkey, *Macaca mulatta* (Table 3). A slight constriction of the vestibule internal to the fenestra vestibuli separates the spherical and elliptical recesses (sr and er in Figure 68A). The bony channel for the vestibular aqueduct opens immediately anterior to the medial edge of the vestibular aperture of the common crus (av in Figure 69, slices 372–408). The channel curves posteriorly and terminates in a triangular fissure. The posterior limb of the lateral semicircular canal does not open directly into the vestibule, but rather into the anterior aspect of the posterior ampulla (lc and pa in Figure 68B). A groove extends from the entry point of the lateral canal to the vestibule across the anterior wall of the posterior ampulla (the groove is expressed as a rounded ridge on the endocast). The lateral semicircular canal does not join with the posterior canal, and a secondary common crus is not formed.

The semicircular canals form delicate arcs (Figure 68), and the canals themselves are slender compared to the rest of bony labyrinth (as opposed to the thick canals observed in *Homo*; Figure 64). The plane of the posterior semicircular canal forms obtuse angles with both the anterior lateral canals (Table 3). The angle between the planes of the anterior and lateral semicircular canals is considerably more acute. The arc radius of curvature of the anterior semicircular canal of *Tupaia* is greater than either the lateral or posterior canals (Table 4). Similarly, the length of the slender portion of the anterior semicircular canal is the greatest. The lateral semicircular canal is largest in terms of cross-sectional diameter also.

The arc of the anterior semicircular canal appears more circular than the lateral and posterior canal arcs (Figure 68), although the aspect ratio of the posterior canal arc is higher than that calculated for the anterior canal (Table 5). This is a result of the method employed to measure the height and width of the posterior semicircular canal arc, which does not reflect the shape of the arc in this situation. The aspect ratio of the lateral semicircular canal more accurately represents the ellipse formed by the lateral canal arc. The ratio of the length of the slender anterior semicircular canal to the arc radius (5.35) is not the greatest, as is observed in most of the mammals examined here, but rather the smallest. The ratio for the lateral canal (5.46) is the largest, and the ratio for the posterior canal falls in between (5.40). The anterior semicircular canal is the least planar among the three canals (Table 5; ac in Figure 68A–B). Both the anterior and posterior semicircular canals deviate from their planes by a substantial amount (ratios of total linear deviation over cross-sectional diameter are 3.76 and 1.54 respectively). The lateral semicircular canal does not deviate from its plane substantially (ratio is 0.97), although nearly so.

The bony labyrinth of *Tupaia* is derived from the ancestral eutherian condition in that the plane of the lateral semicircular canal is high in relation to the ampullar entrance of the posterior canal, although this state was inherited from the most recent common ancestor of boreoeutherians. The lateral semicircular canal opens into the posterior ampulla separate from the posterior canal (a secondary common crus is not formed), a condition that is unique to *Tupaia* among euarchontoglires, but shared by *Hemicentetes*, Cetacea, *Equus*, Carnivora (except *Canis*), and the bats *Nycteris* and *Rhinolophus*. The greatest arc radius of curvature was measured for the anterior semicircular canal in *Tupaia*, which is consistent for most of the therian mammals considered here.

The high aspect ratio of the cochlea of *Tupaia* is derived from the ancestral eutherian condition, which the taxon shares with Glires within Euarchontoglires. The shape of the cochlear spiral may be a synapomorphy supporting a *Tupaia* plus Glires clade, although the ancestral state of Euarchontoglires is equivocal with respect to this character. The cochlea coils to a greater degree (1125°) than that reconstructed for the ancestor of Euarchontoglires (957°), but less than one half turn. The cochlea of Scandentia contributes 55% of the total labyrinthine volume, which is he same percentage calculated for the cochlea of Boreoeutheria.

### Dimension Comparisons

**Figure 70:**
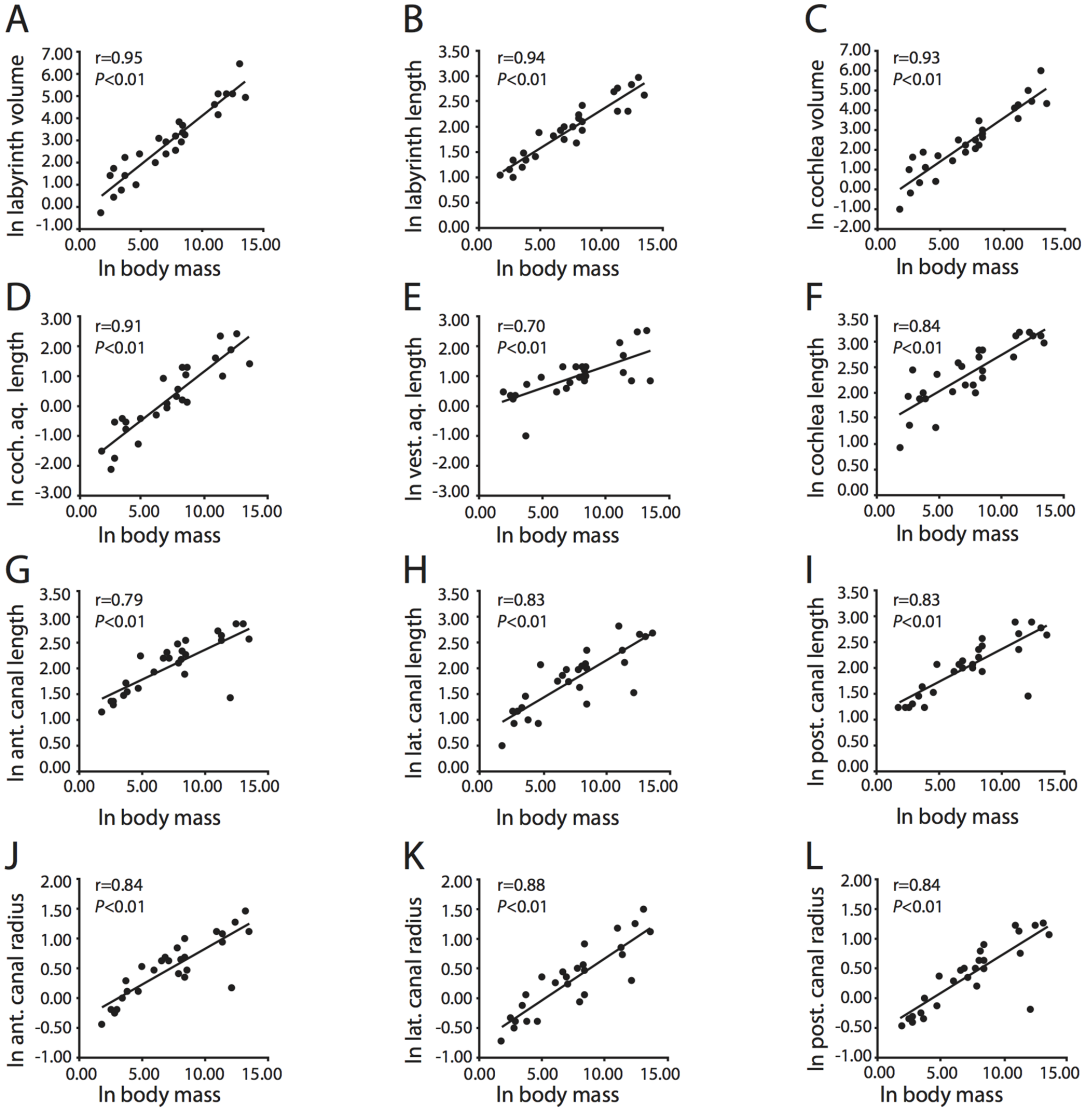
Bivariate plots of labyrinth dimensions over body mass. All data logarithmically transformed using the natural logarithm (ln). **A**, total labyrinth volume over body mass; **B**, total length of labyrinth over body mass; **C**, volume of cochlea over body mass; **D**, length of canaliculus cochleae for aqueduct of cochlea over body mass; **E**, length of bony channel for aqueduct of vestibule over body mass; **F**, length of cochlear canal over body mass; **G**, length of slender portion of anterior semicircular canal over body mass; **H**, length of slender portion of lateral semicircular canal over body mass; **I**, length of slender portion of posterior semicircular canal over body mass; **J**, arc radius of curvature of anterior semicircular canal over body mass; **K**, arc radius of curvature of lateral semicircular canal over body mass; **L**, arc radius of curvature of posterior semicircular canal over body mass. The outlier that falls well below regression line in G-L is *Tursiops truncatus*.

Large-bodied animals tend to have absolutely large bony labyrinths. For example, the inner ear cavities of large-bodied *Trichechus manatus* and *Equus caballus* are among the most voluminous, while the inner ears of *Mus musculus* and *Sorex monticolus* are the smallest. In order to test if there is a correlation between body size and inner ear dimensions, the coefficient of correlation was calculated between specific measurements and body mass (Table 6). The total size of the bony labyrinth, both in terms of the total volume of the cavities and length of the inner ear, are related strongly to body mass across the sample when the data are transformed using the natural logarithm (Figure 70). A coefficient of correlation (r; not to be confused with the radius of the slender portion of a canal of previous authors [54]) of 0.94 was calculated between labyrinth length and body mass, and a coefficient of 0.95 between total labyrinthine volume and body mass.

**Table 6:** Coefficients of correlation (*r*) and significance values (*P*) for dimensions over body mass^a^.

Measurement	r	*P*
Labyrinth		
* Volume*	*0.95*	*<0.01*
* Length*	*0.94*	*<0.01*
Cochlea		
* Volume*	*0.93*	*<0.01*
Percent of Total Volume	0.13	0.51
* Canal Length*	*0.84*	*<0.01*
* Aqueduct Length*	*0.91*	*<0.01*
Coiling	0.02	0.92
2° Lamina Extension	0.36	0.06
Angle with Lateral Canal	0.36	0.06
Aspect Ratio	0.19	0.33
Vestibule		
* Aqueduct Length*	*0.70*	*<0.01*
Stapedial Ratio	0.26	0.18
Semicircular Canal Orientation		
Anterior-Lateral	0.15	0.44
Anterior-Posterior	0.07	0.72
Lateral-Posterior	0.33	0.08
Semicircular Canal Dimensions		
* Anterior Radius*	*0.85*	*<0.01*
* Lateral Radius*	*0.88*	*<0.01*
* Posterior Radius*	*0.84*	*<0.01*
* Anterior Length*	*0.79*	*<0.01*
* Lateral Length*	*0.83*	*<0.01*
* Posterior Length*	*0.83*	*<0.01*
* Anterior Diameter*	*0.83*	*<0.01*
* Lateral Diameter*	*0.81*	*<0.01*
* Posterior Diameter*	*0.82*	*<0.01*
Anterior Linear Deviation	0.33	0.09
* Lateral Linear Deviation*	*0.64*	*<0.01*
Posterior Linear Deviation	0.35	0.07
Anterior Angular Deviation	0.17	0.39
Lateral Angular Deviation	0.19	0.33
Posterior Angular Deviation	0.08	0.69
Anterior Aspect Ratio	0.19	0.33

a Data logarithmically transformed using the natural logarithm. Ratios with *P*-Values under 0.05 (in italics) are considered significant correlations.

Because the size of the bony labyrinth is significantly correlated to the overall size of the animal, the dimensions of the bony labyrinth can be used to estimate the body mass of fossil species. The length of the bony labyrinth rather than its volume is used here to make this estimate, because it is less prone to error. Volumes of the inner ear constituents are calculated from the segmented endocast, where boundaries between the middle and inner ear cavities, or else between the cochlea and vestibule, can be ambiguous for some species. Consistent boundaries are maintained as much as possible, but the longitudinal measure of the length of the bony labyrinth is less ambiguous.

The equation for the regression line between the length of the bony labyrinth and body mass is y = 0.151x+0.8212, where “x” equals the body mass and “y” equals the length of the bony labyrinth. The accuracy of the equation can be tested by estimating the body mass was of *Canis familiaris* specimen, which was not incorporated in the correlations with body mass (see discussion in the Materials and Methods section). Using the equation above, the estimated body mass of the specimen is 4.5 kg, which is at the low range of dog body masses (3.4 to 31.3) [92]. In fact, the breed of dog used in this study is from a Chihuahua, which is among the smallest breeds of domestic dog. This equation can be used to calculate body masses for extinct taxa. For example, the estimated body masses of the oreodont *Bathygenys reevesi* and the fossil balaenopterid whale are 2.5 kg and 1608.3 kg respectively.

Additional dimensions significantly scale with body mass (see Table 6), including the length and volume of the cochlear canal (r equals 0.84 and 0.93 respectively), lengths of the bony channels for the cochlear aqueducts (canaliculus cochleae; r equals 0.91) and vestibule (r equals 0.70), lengths of the slender portions of the anterior (r equals 0.79), lateral (r equals 0.83), and posterior semicircular canals (0.83), as well as the radii of curvature of the semicircular canal arcs (r equals 0.85 for anterior; 0.88 for lateral; 0.84 for posterior). In all cases, the mammals with the largest inner ear dimensions are large-bodied animals (see Figure 70). The aspect ratios of the cochlea (r equals 0.19) and anterior semicircular canal arc (r equals 0.19) do not correlate with body mass. The aspect ratios of the lateral and posterior canal arcs are positively correlated with body mass (r equals 0.50 for lateral; 0.61 for posterior), but the correlations are not considered significant (coefficient of determination r^2^ equals 0.25 and 0.37 respectively; see the Materials and Methods section below).

The degree of coiling exhibited by the cochlea is not correlated with body mass (r equals 0.02). That is, large animals, such as *Equus caballus*, do not have a significantly more or less coiled cochlea than smaller species. Species with a large number of cochlear whorls do not have significantly more voluminous cochleae (r equals 0.25), although weak positive correlations are found between degree of coiling and both canal length (r equals 0.54) and aspect ratio of cochlear spiral (r equals 0.57), as summarized in Table 7. Cochlear volume is not correlated with the aspect ratio of the cochlear spiral (r equals 0.06), although a strong positive correlation was found between the volume and length of the cochlear canal (r equals 0.93). A long cochlear canal does not signify a high-spired cochlea (r equals 0.13).

**Table 7:** Coefficients of correlation*r* (with significance values *P* in parentheses) calculated for dimensions of the cochlea^a^.

	Degree of Coiling	Canal Volume	Canal Length	Aspect Ratio
**Degree of Coiling**	–	0.25 (0.14)	*0.54 (<0.01)*	*0.57 (<0.01)*
**Canal Volume**	0.25 (0.14)	–	*0.93 (<0.01)*	0.06 (0.72)
**Canal Length**	*0.54 (<0.01)*	*0.93 (<0.01)*	–	0.13 (0.44)
**Aspect Ratio**	*0.57 (<0.01)*	0.06 (0.72)	0.13 (0.44)	–

a Data logarithmically transformed using the natural logarithm. Values in italics indicate correlation, although only correlations with coefficients over 0.70 are considered strong.

Table 8 summarizes correlations among dimensions of the semicircular canals. In short, strong correlations were observed between the semicircular canal arc radii of curvature and length of the slender portion of the canal (r ranging from 0.94 to 0.99) for all three canals. The correlations between the aspect ratios of the anterior and later canal arcs and respective lengths or arc radii are not correlated (Table 8), although weak correlations were recovered between posterior canal aspect ratio and both arc radius and canal length (r equals 0.40 and 0.41 respectively).

**Table 8:** Coefficients of correlation*r* (with *P* in parentheses) calculated for dimensions of the semicircular canals^a^.

	Ant			Lat			Post		
	Radius	Length	Ratio	Radius	Length	Ratio	Radius	Length	Ratio
**Radius**	–	*0.98 (<0.01)*	0.30 (0.07)	–	*0.94 (<0.01)*	0.24 (0.15)	–	*0.99 (<0.01)*	*0.40 (0.01)*
**Length**	*0.98 (<0.01)*	–	0.35 (0.03)	*0.94 (<0.01)*	–	0.23 (0.12)	*0.99 (<0.01)*	–	*0.41 (0.01)*
**Ratio**	0.30 (0.07)	0.35 (0.03)	–	0.24 (0.15)	0.23 (0.12)	–	*0.40 (0.01)*	*0.41 (0.01)*	–

a Data logarithmically transformed using the natural logarithm. Values in italics indicate correlation, although only correlations with coefficients over 0.70 are considered strong.

## Discussion

### General Patterns

Observation of variation across the sample of bony labyrinths of placental mammals is a long-recognized phenomenon predating the seminal works of Gustaf Retzius in the late 19^th^ Century [240]. However, the nature of this variation has received only a superficial treatment in the scientific literature; exceptions include only a handful of studies [83], [73], [144], [241]. Variation in the degree of coiling in the cochlea in particular is related to phylogeny [37] and function [21]. The broad range of over 1,400° (nearly 4 turns) within the placental sample examined here may be a reflection of taxonomic diversity, where at least 5,421 extant mammal species are recognized [137]–[138], as well as physiological diversity, where a range of auditory sensitivities extend from infrasonic in proboscideans and cetaceans [22], [242]–[243] to ultrasonic frequencies in some chiropterans, soricid lipotyphlans, and tenrecs [244]–[246].

General patterns in the bony labyrinth anatomy include the arc radius of curvature of the anterior semicircular canal being the largest among the three canals in the majority of the mammals examined here (24 out of 32 species). This pattern has been observed in most mammal species [24], [42], [77], [86], [96], [247]–[249], and a large anterior semicircular canal arc signifies that the majority of mammals are most sensitive to rotational head movement in the pitch (anterior-posterior) direction [29]. Exceptions include *Dasypus novemcinctus*, where the posterior canal is the most sensitive, or *Eumetopias jubatus* where the lateral canal is the most sensitive. In the case of *Eumetopias*, the size of the lateral semicircular canal as the largest might be related to an aquatic lifestyle as discussed previously [58]. The same relative pattern among semicircular canal dimensions is observed in the other aquatic mammals *Trichechus manatus* and *Tursiops truncatus*.

The posterior semicircular canal is the least planar of the three canals in more bony labyrinths (15 out of 32 species) than either the anterior (9 out of 32 species) or lateral canals (8 out of 32species), and the lateral canal is the most planar for the majority of taxa (18 out of 32 species). The ratio of the total linear deviation to the cross-sectional diameter of the semicircular canal is used in the present study to describe the degree of planar deviation of a semicircular canal, where a ratio above 1 (linear deviation greater than diameter) is considered substantial. Any physiological importance of planar deviation has yet to be explored in a rigorous sense, and such substantial deviation may not have any basis in function. The ratio is used for descriptive and comparative purposes only. Although the ratio is arbitrary, evidence suggests that, even in species with broad ranges of planarities (such as in *Monodelphis domestica*) [75], there is not much intraspecific variation in whether or not the ratio is substantial (Table 9). The degree of deviation exhibited both by the anterior and posterior semicircular canals is considered substantial in half of the taxa examined here (16 out of 32), although the deviations of the two canals are not always substantial within the same labyrinth. The deviation of the lateral semicircular canal is substantial in only one quarter of the mammals.

**Table 9:** Linear deviations of the semicircular canals of*Monodelphis domestica* a.

	Specimens (TMM M)											
	7595	8261	8265	7536	8266	7539	7542	8267	7545	8268	8273	7599
Linear Deviations												
Anterior	0.00	0.08	0.05	0.11	0.11	0.00	0.08	0.07	0.10	0.11	0.11	0.07
Lateral	0.00	0.00	0.00	0.05	0.05	0.00	0.00	0.05	0.05	0.04	0.07	0.06
Posterior	0.00	0.08	0.08	0.07	0.07	0.10	0.06	0.06	0.09	0.09	0.09	0.07
Canal Diameters												
Anterior	0.20	0.21	0.29	0.19	0.19	0.21	0.23	0.17	0.17	0.17	0.23	0.18
Lateral	0.27	0.24	0.25	0.19	0.19	0.19	0.22	0.19	0.25	0.20	0.20	0.20
Posterior	0.26	0.18	0.22	0.24	0.19	0.22	0.25	0.22	0.27	0.20	0.24	0.26
Ratios of Linear Deviations over Diameters												
Anterior	0.00	0.38	0.17	0.58	0.58	0.00	0.35	0.41	0.59	0.65	0.48	0.39
Lateral	0.00	0.00	0.00	0.26	0.26	0.00	0.00	0.26	0.20	0.20	0.35	0.30
Posterior	0.00	0.44	0.36	0.29	0.37	0.45	0.24	0.27	0.33	0.45	0.38	0.27

a Scanning parameters were published previously [75]. All specimens housed at the Texas Natural Sciences Center, Austin Texas (TMM M). Linear dimensions expressed in millimeters.

### Functional Considerations

A strong relationship between the size of the bony labyrinth and body mass is to be expected. This phenomenon causes a tricky situation when morphologies within the inner ear are used to make functional interpretations. A clear positive correlation between the arc radius of curvature and sensitivity is evident [29], with absolutely larger canals being more sensitive to rotational movement than smaller canals. Additionally, the size of the canals has been related to locomotor behavior in extant and extinct mammals [10], [23], [26], [250]–[251].

The size of a semicircular canal arc appears to be correlated with agility [24]. In theory, agile mammals will have a larger semicircular canal arc radius (averaged over the three canals within a labyrinth) than slower animals of the same body size. The average radius of the anterior, lateral, and posterior semicircular canals was calculated for each taxon examined in this study, and the average was divided by body mass in order to normalize the data (Table 10). No correlation is recovered when the ratios of arc radius over body mass are plotted over agility (based on a six point scoring system developed by Spoor and colleagues [24]). All data were logarithmically transformed using the natural log.

**Table 10:** Ratios of Semicircular Canal Arc Radius over Body Mass and Canal Length^a^.

Taxon	Radius/Average Body Mass				Length/Radius		
	Ant	Lat	Post	Average	Ant	Lat	Post
Marsupialia							
* Didelphis*	0.05	0.03	0.04	0.04	5.63	5.47	6.11
Eutheria							
* Kulbeckia*	NA	NA	NA	NA	4.80	4.29	4.75
* Ukhaatherium*	NA	NA	NA	NA	4.55	4.28	4.88
* Zalambdalestes*	NA	NA	NA	NA	4.77	4.36	4.53
Zhelestid	NA	NA	NA	NA	4.96	4.40	5.15
Afrotheria							
* Chrysochloris*	2.47	1.52	1.5980	1.8613	4.30	3.89	5.07
* Hemicentetes*	1.00	0.62	0.8045	0.8068	4.52	3.59	5.41
* Macroscelides*	3.43	2.74	2.6670	2.9490	4.25	4.00	5.10
Elephantimorpha	NA	NA	NA	NA	4.93	4.70	4.41
* Orycteropus*	0.01	0.01	0.0060	0.01	4.96	5.03	.5.39
* Procavia*	0.05	0.05	0.06	0.05	5.14	4.28	4.90
* Trichechus*	0.001	0.001	0.0010	0.001	4.02	3.18	4.67
Xenarthra							
* Dasypus*	0.035	0.03	0.040	0.03	5.91	4.63	5.88
Laurasiatheria							
* Atelerix*	0.14	0.10	0.14	0.13	4.74	4.15	4.74
Balaenopteridae	NA	NA	NA	NA	4.19	4.05	4.94
* Bathygenys*	NA	NA	NA	NA	5.08	4.68	5.59
* Canis*	NA	NA	NA	NA	4.97	4.50	5.14
* Equus*	0.001	0.001	0.001	0.001	4.79	4.02	5.32
* Eumetopias*	0.0004	0.0004	0.0004	0.0004	4.33	4.72	4.92
* Felis*	0.06	0.05	0.06	0.05	4.57	4.45	4.93
* Manis*	0.03	0.02	0.04	0.03	4.52	3.49	4.23
* Nycteris*	3.31	2.97	2.70	2.99	4.48	3.91	5.51
* Pteropus*	0.36	0.30	0.31	0.32	4.37	4.56	5.20
* Rhinolophus*	4.81	4.02	4.32	4.38	4.25	4.64	5.25
* Sorex*	10.7	7.95	10.4	9.68	4.91	3.38	5.44
* Sus*	0.003	0.002	0.003	0.003	4.86	3.87	4.89
* Tadarida*	6.97	6.0395	6.0601	6.3556	4.62	4.45	4.88
* Tursiops*	0.001	0.001	0.001	0.001	3.47	3.38	5.17
Euarchontoglires							
* Cavia*	0.26	0.22	0.22	0.23	4.79	4.13	5.02
* Cynocephalus*	0.19	0.15	0.17	0.17	5.15	4.75	4.94
* Homo*	0.004	0.003	0.004	0.004	4.61	4.39	4.76
* Lepus*	0.10	0.07	0.07	0.08	4.89	4.13	4.80
* Macaca*	0.06	0.05	0.05	0.06	4.74	4.29	5.13
* Mus*	5.01	3.89	4.31	4.40	4.98	4.12	5.39
* Sylvilagus*	0.16	0.11	0.12	0.13	4.84	4.38	5.13
* Tupaia*	1.32	1.10	1.14	1.18	5.35	5.46	5.40

a Ratio of radius over body mass multiplied by 100. Sources for body mass: *Eumetopias* [90], *Homo* [91], all other taxa [89].

Although the radius of curvature does not correlate to the agility scores of previous authors [24] when the radius is divided by body mass, the ratios of aquatic taxa are nearly an order of magnitude smaller than the ratios calculated for terrestrial animals, regardless of their evolutionary relationships (see Table 10). This suggests that bony labyrinth morphology can be used to identify aquatic tendencies [23], [25], [85]. For example, the size ratio between the cochlea and vestibular apparatus of cetaceans is greater than that observed in most mammals, and this led to a hypothesis that a reduced vestibular system is an evolutionary response to the rapid body movements within an aquatic environment exhibited by extant cetaceans [23]. Because the mobility of the head and neck in cetaceans nearly is eliminated owing to fusion of cervical vertebrae in some taxa, the vestibulo-collic and vestibulo-ocular reflexes that stabilize the head and eyes during rapid rotations of the body are no longer effective [86]. Thus, larger, more sensitive semicircular canals may no longer compensate for agile movements when the head is unable to move. A reduced vestibular apparatus would reduce sensitivity of the system [29], and lessen any ill effects of an agile lifestyle with cervical fusion.

Although a fully aquatic lifestyle, increased agility, and reduced vestibular systems are observed individually within many mammal taxa, cetaceans are unique in having the full suite of these characteristics. For example, the vestibule and its associated semicircular canals contribute a significantly smaller proportion of the entire bony labyrinth in *Rhinolophus ferrumequinum*, similar to that observed in cetaceans, but *Rhinolophus* does not inhabit an aquatic environment. Likewise, sirenians are fully aquatic, but they are among the least agile mammals [24] and their cervical vertebrae are not fused [136].

The low contribution of the vestibule (or inversely, the high contribution of the cochlea) might be related to an aquatic lifestyle nonetheless. As an initial investigation of this hypothesis, the relative contributions of the cochlea and vestibule are compared between terrestrial and aquatic taxa examined in this study. Because bats are the only true volant mammals and their ears likely are specialized for aerial locomotion, Chiroptera was not incorporated into this comparison. The vestibular contribution of *Tursiops* and the balaenopterid (6% and 9% respectively) are less than that observed in terrestrial mammals (range of 28% for *Macroscelides* to 69% for the elephantimorph). The vestibular contribution of *Trichechus* (29%) is on the low end of the entire mammal range, but it is still greater than the vestibular apparati of both *Macroscelides* (28%) and *Chrysochloris* (29%), which are strictly terrestrial. Furthermore, the vestibular apparatus of *Eumetopias* contributes 47% of total labyrinthine volume, which is only slightly larger than the mean for terrestrial mammals (44%). This suggests that aquatic behavior alone cannot explain a reduced vestibular system in all aquatic taxa.

Although the ranges of vestibular contribution overlap between the terrestrial and aquatic samples, the means of each group may differ significantly. The small number of aquatic species used here limits the effectiveness of statistical analysis. The hypothesis that the mean contribution of the vestibule differs significantly between terrestrial and aquatic mammals was tested, with a two-tailed *t*-test assuming unequal variances (determined through an *F*-test). An *a priori* significance level of 0.05 was selected [88], and the null hypothesis is that the mean contributions of the vestibule are equal between the aquatic and terrestrial samples. The results of the analysis (p = 0.007, which is less than the significance level of 0.05) reject the null hypothesis and suggest that the vestibular contribution of aquatic mammals is significantly less than that of terrestrial mammals. However, a more thorough analysis incorporating larger sample sizes is needed before a formal conclusion can be made. The aquatic sample is very small, and cetaceans are overrepresented (50% of the aquatic taxa) within the sample, which potentially skews the analysis. Furthermore, a large cochlea relative to a small vestibule could be the result of an enlargement of the cochlea, and reduction of the vestibule, or a combination of both. Such a phenomenon deserves further investigation.

Nonetheless, the ratio of semicircular canal arc over body mass is significantly reduced compared to terrestrial species. Furthermore, the vestibules of the two aquatic species *Trichechus manatus* and *Eumetopias jubatus* appear as though they have been compressed (see Figures 18 and 36). The deflation may, in essence, reflect a reduction of the membranous utricle and saccule within the bony vestibule.

Further aspects of bony labyrinth morphology are thought to be related to aquatic behavior, namely dimensions of the semicircular canals and their respective arcs. The ratio between the length of the slender portion of the semicircular canal over arc radius reflects the frequencies of neural impulses transduced from mechanoreceptors within the membranous labyrinth during rotation of the canal [85]. Differences in the ratio might indicate different locomotor abilities, such as semiaquatic versus fully terrestrial. The only pattern observed in the data presented here is that the ratios of length to radius of the anterior semicircular canal of aquatic species tends to be less than that calculated for their close terrestrial relatives (Table 10). Although a pattern is observed, the sample size and taxonomic resolution of the current study is insufficient to postulate a formal connection between the ratio of the length of the slender portion of the duct to arc radius and locomotor behavior.

The aspect ratios of the arcs of the anterior and posterior semicircular canals of aquatic amniotes tend to be significantly lower than their close terrestrial relatives [25], [252]. However, an opposite situation is observed across the mammalian sample examined here, where the radii of the arcs of the two vertical canals (anterior and posterior) are greater for every case in which the canals were compared between aquatic and their closest terrestrial relatives (see Table 3). Methodological differences in the calculation of aspect ratios between the present investigation and previous studies [25], [252] might account for the discrepancy in the pattern, or else mammals may in fact exhibit the opposite pattern from other amniotes. In addition, the taxonomic resolution may be too coarse, and the pattern of low aspect ratios may be reflected in comparisons of much more closely related species.

An additional avenue that has yet to be explored sufficiently is not in the shape or size of the semicircular canals, but rather in the variation of orientations among the canals [144], [253]–[255]. Ongoing research by those and other authors seeks to relate the angles between canal planes and the vectors of canals to sensitivity and locomotor behaviors, and the result of that research will allow the reconstruction of behaviors in extinct mammals.

### Phylogenetic Considerations

There are major structural differences within the membranous labyrinth that likely contain a phylogenetic signal [30], [49], [256]. Two particular features identified in previous studies [49], pigmentation within the membranes and size of the perilymphatic space surrounding the membranous semicircular ducts (which are filled with endolymph themselves), cannot be assessed from the morphology of the bony labyrinth alone. The perilymphatic space has been considered to be an important character in regard to mammal phylogeny, and a large space likely is ancestral for mammals given the large space within the semicircular canals of birds and other reptiles [49]. Unfortunately, the caliber of the bony canal approximates the shape of the membranous duct within, but not necessarily the size [96].

Additional features that may have an importance in determining evolutionary relationships that can be observed within the bony labyrinth include the size of otoliths within the vestibular apparatus, coiling of the cochlea, and shape of the cochlear spiral. The otoliths are tiny in most mammal species, but sizeable otoliths have been observed within the labyrinths of the marsupial *Petrogale penicillata*, the cetaceans *Balaena australis* (now referred to as *Eubalaena australis*) and *Phocaena communis*, the sirenian *Dugong dugon*, and the pinniped carnivorans *Phoca vitulina*, *Halichoerus grypus*, and *Otaria pulsilla* [30]–[31], [49]. However, otoliths were not observed in the CT image data of any specimen examined in the present study, including investigated members of Cetacea, Sirenia, and Carnivora.

There are several reasons for the absence of otoliths on the CT scans. The composition and density of otoliths makes it virtually impossible that they would have been missed in CT data if present. Indeed, CT scans of many non-mammalian vertebrates reveal otoliths [257]–[259]. Alternatively, otoliths may be lacking within the bony labyrinth at the time of scanning, either because large otoliths do not occur in life in these species, or else through loss during skeletal preparation. The latter cannot be ruled out, because specimens with previously observed large otoliths [30]–[31], [49] that were used in this study are dried skulls, and it is conceivable that the otoliths fell out of the ear cavity once the membranes holding them decayed. Even so, otoliths were not observed in specimens that were preserved in alcohol, such as *Chrysochloris* and *Atelerix*, thereby preserving the membranous labyrinths with the otoliths supposedly intact.

Two cochlear shapes termed “sharp-pointed” (observed in rodents, lagomorphs, and non-pinniped carnivorans) and “flattened” (observed in pinnipeds, primates, cetartiodactyls and perissodactyls) are thought to be phylogenetically informative [49]. The distinction between the two morphotypes is not clear, although they roughly correspond to the aspect ratios of the cochlea reported in the present investigation. That is, the taxa with the “sharp-pointed” condition tend towards high aspect ratios, above 0.55, whereas the aspect ratios calculated for species with “flattened” cochleae are 0.55 or less. A couple of exceptions to the generality are *Eumetopias*, which has a cochlear aspect ratio of 0.68 similar to other carnivorans, and *Sus*, which has an aspect ratio of 0.71. However, Gray [30] described the cochlea of *Sus* as intermediate between “flattened” and “sharp-pointed”, but he described the cochleae of pinnipeds as “flattened”.

**Figure 71:**
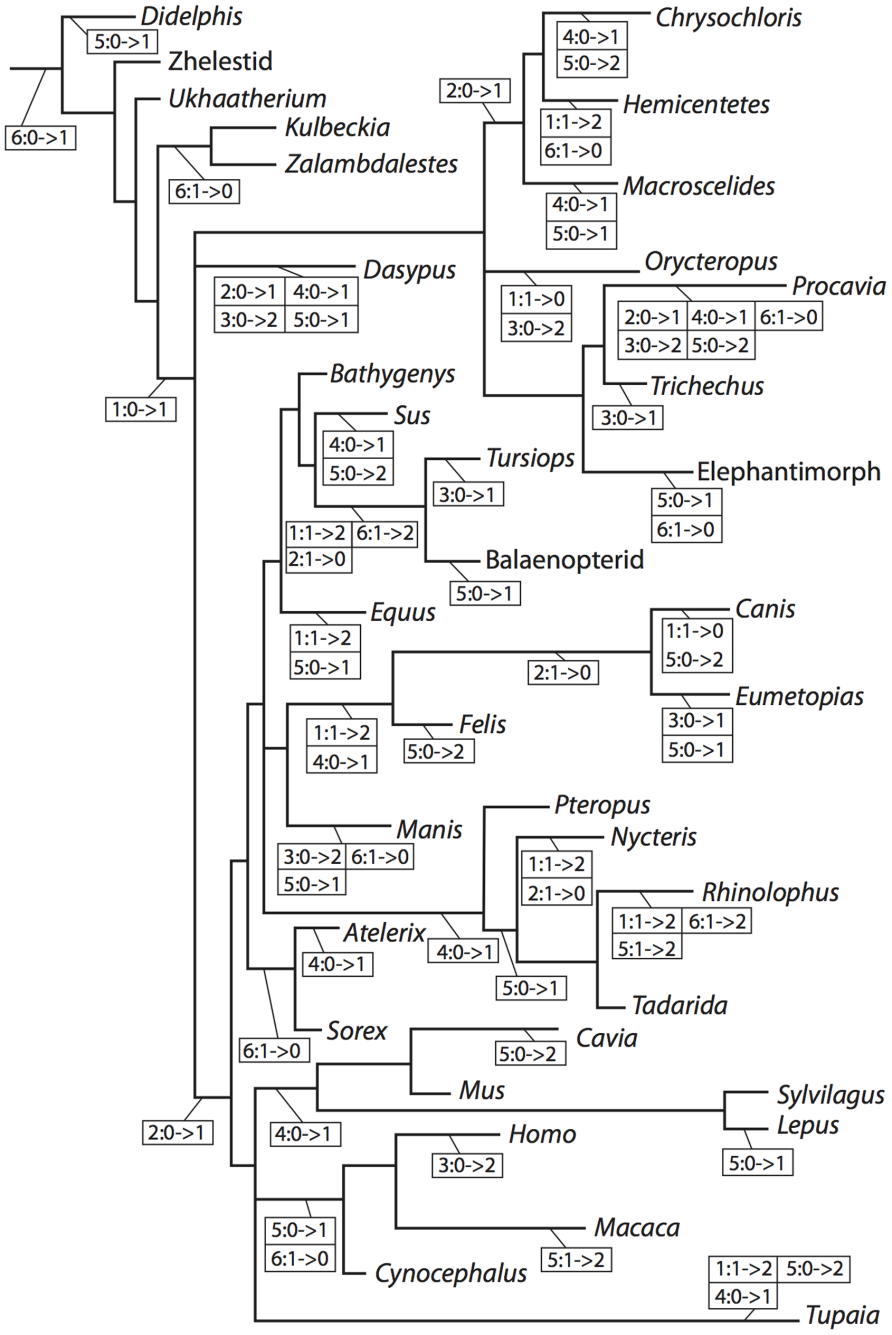
Characters of the bony labyrinth traced across placental mammal phylogeny. Characters: **1**, Entry of posterior limb of lateral semicircular canal – (0): secondary common crus, (1): posterior ampulla, (2): vestibule; **2**, Position of plane of lateral semicircular canal relative to posterior semicircular canal when labyrinth is in anterior view – (0): low (sagittal labyrinthine index = 0.0), (1): hight (sagittal labyrinthine index >0.0); **3**, Largest semicircular canal arc radius of curvature – (0): anterior canal, (1): lateral canal, (2): posterior canal; **4**, Shape of cochlear spiral – (0): low (aspect ratio ≤0.55), (1): high (aspect ratio >0.55); **5**, Number of cochlear turns – (0): 1 to 2 turns (∼360–720°), (1): 2 to 3 turns (720–1080°), (2): over 3 turns (>1080°); **6**, Percent volume of total bony labyrinth conrtributed by cochlea – (0): ≤50%, (1): 51–75%, (2): >75%.

The cochlea of *Cavia porcellus* has the highest aspect ratio (1.29), and it is the only species in this study in which the height of the cochlear spiral is greater than the width across the basal turn of the cochlea. Similar high-spired cochleae are observed within other caviomorph rodents, including *Hydrochoerus capybara* [31], *Myocastor coypu* [260], *Dolichotis patagonum* (personal observation), and *Chinchilla laniger* (personal observation). A high-spired cochlea is likely a synapomorphy for caviomorph rodents (character 4 in Figure 71), although the cochlea of the North American porcupine, *Erethizon dorsatum*, which is nested well within Caviomorpha [261]–[262], is low spired as observed in *Mus musculus* (Figure 54) and non-caviomorph rodents [30]–[31]. Absence of the high-spired cochlea might be a retention of the ancestral state in *Erethizon*, but it more likely is a reversal. A more thorough investigation of the bony labyrinths of caviomorph and closely related non-caviomorph rodents is needed to fully explore the issue.

The coiling of the cochlea is phylogenetically informative and can be used to distinguish therian and non-therian mammals [35], where all extant therian cochleae are coiled to at least 360°. One exception is *Uchkudukodon nessovi* from the Cretaceous of Uzbekistan, which has a cochlea that completes less than 360° [263]. A single turn likely is plesiomorphic for Eutheria [37], [83], and a high degree of coiling developed more than once within placental lineages (character 5 in Figure 71). However, the number of cochlear whorls was not found in the present study to be phylogenetically informative within Placentalia and could not be used to distinguish major placental clades. For example, *Mus musculus* and *Pteropus lylei* both possess a low degree of coiling (628° and 656° respectively), but other members of their clades possess high degrees of coiling (e.g., 1457° in *Cavia porcellus* and 1115° in *Rhinolophus ferrumequinum*). Phylogenetic patterns extend beyond Mammalia, and additional systematic information can be found in the inner ear labyrinths of squamate reptiles [43]–[47].

The stapedial ratio is an index commonly used in phylogenetic analyses to explore the relationships between Mesozoic therians [3]–[5], [264]. It often is assumed that, with a few exceptions [87], [97], marsupials have fenestrae vestibuli that are more circular (with a height to width ratio of the stapedial footplate below 1.8; dimensions of the fenestra vestibuli are used as a proxy in the absence of the stapes) than placentals [87]. The only marsupial considered here (*Didelphis virginiana*) does, in fact, possess a fenestra vestibuli that falls below the 1.8 cut-off (ratio of 1.6). However half of the placentals examined here (15; note that no ratio was calculated for *Bathygenys*) exhibit the ‘marsupial condition’ (below 1.8) of Segall [87]. In fact, the ratio for *Nycteris* is 1.0, which is the observed condition among monotremes [87]. This result indicates that a thorough exploration of the stapedial ratio across a broad range of marsupial and placental taxa is necessary before using the ratio in phylogenetic studies, or the character should be treated as continuous at the very least.

A further example of the phylogenetic significance of the bony labyrinth of mammals is the relative contributions of the cochlea and vestibule to the entire labyrinth (character 6 in Figure 71). The size of the vestibular apparatus certainly is correlated to function (as discussed above), but a reduced vestibule is also a synapomorphy for Cetacea, if only among cetartiodactyls. The contribution of the vestibule of *Rhinolophus* is similar to that observed in cetaceans, but the vestibular (or inversely, cochlear) contribution calculated for other bats fall within the range of other mammals. Because a large cochlea is phylogenetically informative for Cetacea, the phenomenon may also be informative within Chiroptera, upon which a denser sampling of taxa might shed light. Furthermore, a low contribution of the cochlea independently unites zalambdalestids, primatomorphs, and eulipotyphlans (character 6 in Figure 71).

Variation was observed among the largest semicircular canal arc radius of curvature. Although the anterior canal arc is the largest in most species examined here, the lateral semicircular canal arc has the largest radius in *Trichechus*, *Tursiops*, and *Eumetopias*, and the posterior canal arc is largest in *Dasypus*, *Homo*, *Manis*, and *Orycteropus*. However, the distribution of which canal is largest is scattered across the phylogeny and the anterior semicircular canal arc is reconstructed as the largest at every ancestral node excepting the most recent common ancestor of *Procavia* and *Trichechus* (character 3 in Figure 71, Table S3).

The secondary common crus between the lateral and posterior semicircular canals is an ancestral feature for Theria (character 1 in Figure 71). All Cretaceous therians possess the secondary crus [34], [83], although the structure is lost within most extant placental groups (Figure 71). In fact, the only extant mammals considered in this study having the secondary crus are the marsupial *Didelphis virginiana*, as well as the placentals *Canis familiaris* and *Orycteropus afer*. The lateral semicircular canal opens directly into the vestibule at its posterior end in the vast majority of the species examined here (20 out of 32), and absence of a secondary common crus is a synapomorphy for crown Placentalia (Figure 71).

A third state is entry of the lateral semicircular canal into the posterior ampulla rather than the vestibule, but separate from the posterior canal (a secondary common crus is not developed). Although the entry of the posterior limb of the lateral semicircular canal does not express any major pattern with the taxonomic sampling employed by the current study, potential for informativeness at lower levels is apparent. For example, the lateral canal opens into the posterior ampulla in the cetaceans, but it opens into the vestibule in terrestrial artiodactyls (*Bathygenys* and *Sus*). Even so, entry of the lateral canal into the posterior ampulla is reconstructed as a synapomorphy of Cetacea, as well as Carnivora (character 1 in Figure 71). A denser sampling at lower taxonomic levels within these groups, as well as Perissodactyla and Chiroptera, may reveal phylogenetic patterns that are lost at the coarse resolution of this study.

Possibly related to the entry of the posterior limb of the lateral semicircular canal is the position of the plane of the lateral canal with respect to the posterior semicircular canal. Ancestrally, the lateral canal took a low position but is elevated in most placental clades (character 2 in Figure 71). Within Placentalia, a low position of the lateral semicircular canal unites Cetacea and Caniformia. In both of those situations, the low position of the canal plane may be the result of the entry of the lateral canal into either the secondary common crus or posterior ampulla. However, the entry of the lateral semicircular canal into the posterior ampulla does not necessarily indicate a low position of the lateral canal (e.g., *Equus*; Figure 71).

### Conclusions

Great advancements have been made in our understanding of the anatomy, physiology, and evolution of the inner ear labyrinth over the past 50 years. The morphological descriptions of the bony labyrinth across Placentalia presented here illustrate the anatomical diversity present within and among major clades of mammals. Not surprisingly, many of the individual dimensions of the inner ear correlate with each other, as well as the overall body size of the individual animal. Certainly the morphology of the inner ear cavities is physiologically significant (e.g., size of a semicircular canal is related to its sensitivity), but potentially phylogenetic patterns are observed (e.g., high aspect ratio of the cochlea of caviomorph rodents). In order to fully understand the functional and evolutionary implications within the structure of the bony labyrinth, both physiology and phylogeny must be considered, as these two phenomena are not mutually exclusive. Future detailed studies of the inner ear among closely related species will increase our knowledge of the phylogenetic and functional implications of the inner ear, and foster the application of bony labyrinth morphology to the biological interpretation of fossil vertebrates.

## Supporting Information

Table S1 **Taxa examined and scanning parameters.** ^a^ Definitions of parameters are as follows: FR, field of reconstruction refers to the dimensions of an individual CT slice, expressed in millimeters; Pixel, interpixel spacing, or vertical and horizontal dimensions of an individual pixel, expressed in millimeters, and calculated as FR/Size; Size, number of pixels in a CT slice, either 512×512 or 1024×1024 pixels; Slices, number of CT slices through the ear collected in the coronal (original) slice plane; Space, interslice spacing, or distance between consecutive slices, expressed in millimeters. ^b^ Taxonomy and systematic arrangement follows published phylogenies [6], [66]. Institutional abbreviations: AMNH, American Museum of Natural History, New York; MSW, Mortality South West; PSS-MAE, Collections of Joint Paleontological and Stratigraphic Section of the Geological Institute, Mongolian Academy of Science, Ulaanbaatar – American Museum of Natural History, New York; SDSNH, San Diego Society of Natural History, San Diego, CA; TMM, Texas Natural Science Center, Austin, TX; URBAC, Uzbekistan/Russian/British/American/Canadian joint paleontological expedition, Kyzylkum Desert, Uzbekistan, specimens in the Institute of Zoology, Tashkent; UTO-HS, University of Texas at Austin, Department of Anthropology Teaching Collection. ^c^ This specimen was the 156^th^ Mortality South West in 2003, collected by S. Rommel at University of North Carolina Wilmington.
(PDF)

Table S2 **Additional information, imagery, and sources of data selected specimens.** Further imagery is available at http://morphobank.org/index.php/Projects/ProjectOverview/project_id/833. Institutional abrreviations listed in Table S1.
(PDF)

Table S3 **Ancestral character state reconstructions for ancestral nodes in text** **Figure 2** **.** Letters in the first column refer to node labels in text Figure 2. Ancestral states reconstructed in Mesquite [94]. Definitions of characters: LSC Entry, entry of the posterior limb of the lateral semicircular canal into the secondary common crus, posterior ampulla, or vestibule; LSC Position, position of the plane of the lateral semicircular canal relative to the inferior limb of the posterior semicircular canal when the bony labyrinth is in anterior view; Largest Canal, largest semicircular canal arc radius of curvature among the anterior, lateral, and posterior semicircular canals; Cochlea Ratio, aspect ratio of cochlear spiral binned as either low (ratio equaling 0.55 or below) or high (ratio above 0.55), numerical values reported in text; Coiling, number of turns completed by the cochlea binned as 1–2 turns (360–720°), 2–3 turns (720–1080°), or 3+ turns (over 1080°), numerical values reported in text; % Cochlea, percent volume of the cochlea to total labyrinthine volume binned as ≤50%, 51–75%, or >75%, numerical values reported in text.
(PDF)
