# Correction: Comparative Anatomy of the Bony Labyrinth (Inner Ear) of Placental Mammals

**DOI:** 10.1371/journal.pone.0137149

**Published:** 2015-08-26

**Authors:** Eric G. Ekdale

The specimen number for Eumetopias jubatus in Table S1 of the Supporting Information is incorrect. This specimen is not accessioned into a museum collection and thus does not have an associated specimen number. Specimen number TMM M-171 is associated with Equus caballus as identified correctly in the table.
