# Supplementary material for: Comparative Anatomy of the Bony Labyrinth (Inner Ear) of Placental Mammals
Source: PLoS One. 2013 Jun 21;8(6):e66624. doi: 10.1371/journal.pone.0066624 (PMC3689836; doi:10.1371/journal.pone.0066624)
Supplement: Table S1 — Taxa examined and scanning parameters. a Definitions of parameters are as follows: FR, field of reconstruction refers to the dimensions of an individual CT slice, expressed in millimeters; Pixel, interpixel spacing, or vertical and horizontal dimensions of an individual pixel, expressed in millimeters, and calculated as FR/Size; Size, number of pixels in a CT slice, either 512×512 or 1024×1024 pixels; Slices, number of CT slices through the ear collected in the coronal (original) slice plane; Space, interslice spacing, or distance between consecutive slices, expressed in millimeters. b Taxonomy and systematic arrangement follows published phylogenies [6], [66]. Institutional abbreviations: AMNH, American Museum of Natural History, New York; MSW, Mortality South West; PSS-MAE, Collections of Joint Paleontological and Stratigraphic Section of the Geological Institute, Mongolian Academy of Science, Ulaanbaatar – American Museum of Natural History, New York; SDSNH, San Diego Society of Natural History, San Diego, CA; TMM, Texas Natural Science Center, Austin, TX; URBAC, Uzbekistan/Russian/British/American/Canadian joint paleontological expedition, Kyzylkum Desert, Uzbekistan, specimens in the Institute of Zoology, Tashkent; UTO-HS, University of Texas at Austin, Department of Anthropology Teaching Collection. c This specimen was the 156th Mortality South West in 2003, collected by S. Rommel at University of North Carolina Wilmington. (PDF) [file pone.0066624.s001.pdf]

TABLE S1. Taxa examined and scanning parameters[a].

**Taxon**[b]	**Slices**	**Space**	**FR**	**Pixel**	**Size**
Marsupialia					
Didelphis virginiana (TMM M-2517)	111	0.132	61	0.0596	1024
Eutheria					
Kulbeckia kulbecke (URBAC 04-36)	387	0.016	14.9	0.0146	1024
Ukhaatherium nessovi (PSS-MAE 110)	59	0.080	15	0.0290	512
Zalambdalestes lechei (PSS-MAE 108)	66	0.113	24.5	0.0479	512
Zhelestid (URBAC 03-39)	536	0.016	14.9	0.0146	1024
Afrotheria					
Afrosoricida					
Chrysochloris sp. (AMNH 82372)	85	0.050	31	0.0303	1024
Hemicentetes semispinosum (AMNH 100837)	56	0.067	48	0.0469	1024
Macroscelidea					
Macroscelides proboscideus (AMNH 161535)	151	0.055	22.5	0.0220	1024
Tubulidentata					
Orycteropus afer (AMNH 51909)	136	0.202	95	0.0930	1024
Hyracoidea					
Procavia capensis (TMM M-4351)	180	0.799	70	0.0683	1024
Sirenia					
Trichechus manatus (MSW 03156)[c]	229	0.300	80	0.1563	512
Proboscidea					
Elephantimorpha (TMM 933-950)	275	0.134	53	0.0518	1024
Xenarthra					
Dasypus novemcinctus (TMM M-152)	494	0.291	25	0.0244	1024
Laurasiatheria					
Ceatartiodactyla					
Bathygenys reevesi (TMM 40209-198)	149	0.141	64	0.0625	1024
Sus scrofa (TMM M-2689)	601	0.033	31	0.0303	1024
Balaenopteridae (TMM 42958-35)	1131	0.072	64.8	0.0633	1024
Tursiops truncatus (SDSNH 21212)	346	0.128	40	0.0391	1024
Perissodactyla					
Equus caballus (TMM M-171)	645	0.115	54	0.0567	1024
Carnivora					
Canis familiaris (TMM M-150)	92	0.144	68	0.0664	1024
Eumetopias jubatus (TMM M-171)	645	0.115	54	0.0527	1024
Felis catus (TMM M-968)	627	0.033	31	0.0303	1024
Pholidota					
Manis tricuspis (AMNH 53896)	101	0.116	35.5	0.0347	1024
Chiroptera					
Pteropus lylei (AMNH 237593)	188	0.447	41	0.0400	1024
Nycteris grandis (AMNH 268369)	70	0.072	67	0.0654	1024
Rhinolophus ferrumequinum (AMNH 245591)	45	0.097	44	0.0430	1024
Tadarida brasiliensis (TMM M-3030)	380	0.010	9.9	0.0097	1024

TABLE S1. (Continued)

Eulipotyphla					
Atelerix albiventris (unvouchered)	68	0.082	65	0.0635	1024
Sorex monticolus (unvouchered)	130	0.265	12	0.0117	1024
Euarchontoglires					
Rodentia					
Cavia porcellus (TMM M-7283)	728	0.038	29.4	0.0287	1024
Mus musculus (TMM M-3196)	84	0.044	12.6	0.0246	512
Lagomorpha					
Lepus californicus (TMM M-7500)	114	0.144	67	0.0654	1024
Sylvilagus floridanus (TMM M-2689)	325	0.034	30	0.0293	1024
Primates					
Macaca mulatta (TMM M-5987)	1121	0.033	31	0.0303	1024
Homo sapiens (UTO-HS01)	636	0.027	24.84	0.0272	1024
Dermoptera					
Cynocephalus volans (AMNH 187859)	350	0.028	22	0.0215	1024
Scandentia					
Tupaia glis (TMM M-2256)	537	0.038	59	0.0576	1024

[a] Definitions of parameters are as follows: FR, field of reconstruction refers to the dimensions of an individual CT slice, expressed in millimeters; Pixel, interpixel spacing, or vertical and horizontal dimensions of an individual pixel, expressed in millimeters, and calculated as FR/Size; Size, number of pixels in a CT slice, either 512X512 or 1024X1024 pixels; Slices, number of CT slices through the ear collected in the coronal (original) slice plane; Space, interslice spacing, or distance between consecutive slices, expressed in millimeters.

[b] Taxonomy and systematic arrangement follows published phylogenies [6,66]. Institutional abbreviations: AMNH, American Museum of Natural History, New York; MSW, Mortality South West; PSS-MAE, Collections of Joint Paleontological and Stratigraphic Section of the Geological Institute, Mongolian Academy of Science, Ulaanbaatar – American Museum of Natural History, New York; SDSNH, San Diego Society of Natural History, San Diego, CA; TMM, Texas Natural Science Center, Austin, TX; URBAC, Uzbekistan/ Russian/ British/ American/ Canadian joint paleontological expedition, Kyzylkum Desert, Uzbekistan, specimens in the Institute of Zoology, Tashkent; UTO-HS, University of Texas at Austin, Department of Anthropology Teaching Collection

[c] This specimen was the 156th Mortality South West in 2003, collected by S. Rommel at University of North Carolina Wilmington.
