# Supplementary material for: Comparative Anatomy of the Bony Labyrinth (Inner Ear) of Placental Mammals
Source: PLoS One. 2013 Jun 21;8(6):e66624. doi: 10.1371/journal.pone.0066624 (PMC3689836; doi:10.1371/journal.pone.0066624)
Supplement: Table S2 — Additional information, imagery, and sources of data selected specimens. Further imagery is available at http://morphobank.org/index.php/Projects/ProjectOverview/project_id/833. Institutional abrreviations listed in Table S1. (PDF) [file pone.0066624.s002.pdf]

TABLE S2. Additional information, imagery, and sources of data selected specimens. Further imagery is available at http://morphobank.org/index.php/Projects/ProjectOverview/project_id/833. Institutional abrreviations listed in Table S1.

Taxon	Online and Literature Data Sources
Marsupialia	
Didelphis virginiana (TMM M-2517)	http://digimorph.org/specimens/Didelphis_virginiana/
Eutheria	
Kulbeckia kulbecke (URBAC 04-36)	[83,97]
Ukhaatherium nessovi (PSS-MAE 110)	[83]
Zalambdalestes lechei (PSS-MAE 108)	[83]
Zhelestid (URBAC 03-39)	[83,97]
Afrotheria	
Afrosoricida	
Chrysochloris sp. (AMNH 82372)	http://digimorph.org/specimens/Chrysochloris_sp/head/
Hemicentetes semispinosum (AMNH 100837)	http://digimorph.org/specimens/Hemicentetes_semispinosus/body/
Macroscelidea	
Macroscelides proboscideus (AMNH 161535)	http://digimorph.org/specimens/macroscelides_proboscideus/skull/
Tubulidentata	
Orycteropus afer (AMNH 51909)	http://digimorph.org/specimens/Orycteropus_afer/
Hyracoidea	
Procavia capensis (TMM M-4351)	http://digimorph.org/specimens/Procavia_capensis/
Sirenia	
Trichechus manatus (MSW 03156)[c]	–
Proboscidea	
Elephantimorpha (TMM 933-950)	[84]
Xenarthra	
Dasypus novemcinctus (TMM M-152)	[83]
Laurasiatheria	
Ceatartiodactyla	
Bathygenys reevesi (TMM 40209-198)	http://digimorph.org/specimens/Bathygenys_reevesi/
Sus scrofa (TMM M-2689)	–
Balaenopteridae (TMM 42958-35)	–
Tursiops truncatus (SDSNH 21212)	http://digimorph.org/specimens/Tursiops_truncatus/
Perissodactyla	

Equus caballus (TMM M-171)	–
Carnivora	
Canis familiaris (TMM M-150)	http://digimorph.org/specimens/Canis_familiaris/chihuahua/
Eumetopias jubatus (TMM M-171)	–
Felis catus (TMM M-968)	–
Pholidota	
Manis tricuspis (AMNH 53896)	http://digimorph.org/specimens/Manis_tricuspis/skull/
Chiroptera	
Pteropus lylei (AMNH 237593)	http://digimorph.org/specimens/Pteropus_lylei/
Nycteris grandis (AMNH 268369)	http://digimorph.org/specimens/Nycteris_grandis/head/
Rhinolophus ferrumequinum (AMNH 245591)	http://digimorph.org/specimens/Rhinolophus_ferrumequinum/head/
Tadarida brasiliensis (TMM M-3030)	[83]
Eulipotyphla	
Atelerix albiventris (uncatalogued)	http://digimorph.org/specimens/Hedgehog_sp/head/
Sorex monticolus (uncatalogued)	http://digimorph.org/specimens/Sorex_monticolus/head/
Euarchontoglires	
Rodentia	
Cavia porcellus (TMM M-7283)	http://digimorph.org/specimens/Cavia_porcellus/
Mus musculus (TMM M-3196)	http://digimorph.org/specimens/Mus_musculus/
Lagomorpha	
Lepus californicus (TMM M-7500)	http://digimorph.org/specimens/Lepus_californicus/
Sylvilagus floridanus (TMM M-2689)	–
Primates	
Macaca mulatta (TMM M-5987)	–
Homo sapiens (UTO-HS01)	http://digimorph.org/specimens/Homo_sapiens/
Dermoptera	
Cynocephalus volans (AMNH 187859)	–
Scandentia	
Tupaia glis (TMM M-2256)	–
