# Supplementary material for: Comparative Anatomy of the Bony Labyrinth (Inner Ear) of Placental Mammals
Source: PLoS One. 2013 Jun 21;8(6):e66624. doi: 10.1371/journal.pone.0066624 (PMC3689836; doi:10.1371/journal.pone.0066624)
Supplement: Table S3 — Ancestral character state reconstructions for ancestral nodes in text Figure 2 . Letters in the first column refer to node labels in text Figure 2. Ancestral states reconstructed in Mesquite [94]. Definitions of characters: LSC Entry, entry of the posterior limb of the lateral semicircular canal into the secondary common crus, posterior ampulla, or vestibule; LSC Position, position of the plane of the lateral semicircular canal relative to the inferior limb of the posterior semicircular canal when the bony labyrinth is in anterior view; Largest Canal, largest semicircular canal arc radius of curvature among the anterior, lateral, and posterior semicircular canals; Cochlea Ratio, aspect ratio of cochlear spiral binned as either low (ratio equaling 0.55 or below) or high (ratio above 0.55), numerical values reported in text; Coiling, number of turns completed by the cochlea binned as 1–2 turns (360–720°), 2–3 turns (720–1080°), or 3+ turns (over 1080°), numerical values reported in text; % Cochlea, percent volume of the cochlea to total labyrinthine volume binned as ≤50%, 51–75%, or >75%, numerical values reported in text. (PDF) [file pone.0066624.s003.pdf]

TABLE S3. Ancestral character state reconstructions for ancestral nodes in text Figure 2[a].

Node Names	LSC Entry	LSC Position	Largest Canal	Cochlea Ratio	Coiling	% Cochlea
A. Theria	secondary common crus	low	anterior	low	1-2 turns	51-75%
B. Eutheria	secondary common crus	low	anterior	low	1-2 turns	51-75%
C. *Ukhatherium*-Placentalia	secondary common crus	low	anterior	low	1-2 turns	51-75%
D. Zalambdalestidae+Placentalia	secondary common crus	low	anterior	low	1-2 turns	≤50%
E. Zalamdalestidae	secondary common crus	low	anterior	low	1-2 turns	≤50%
F. Placentalia	vestibule	–	anterior	–	1-2 turns	51-75%
G. Afrotheria	vestibule	–	anterior	–	1-2 turns	51-75%
H. Afrosoricida+*Macroscelides*	vestibule	high	anterior	–	1-2 turns	51-75%
I. Afrosoricida	vestibule	high	anterior	–	1-2 turns	51-75%
J. Paenungulata	vestibule	–	anterior	–	1-2 turns	–
K. *Procavia*+*Trichechus*	vestibule	–	–	–	1-2 turns	–
L. Boreoeutheria	vestibule	high	anterior	–	1-2 turns	51-75%
M. Laurasiatheria	vestibule	high	anterior	–	1-2 turns	51-75%
N. Ungulates+Ferae+Chiroptera	vestibule	high	anterior	–	–	51-75%
O. Cetartiodactyla+Perissodactyla	vestibule	high	anterior	low	–	51-75%
P. Cetartiodactyla	vestibule	high	anterior	low	–	51-75%
Q. *Sus*+Cetacea	vestibule	high	anterior	low	–	51-75%
R. Cetacea	posterior ampulla	low	anterior	low	–	>75%
S. Ferae	vestibule	high	anterior	–	–	51-75%
T. Carnivora	posterior ampulla	high	anterior	high	–	51-75%
U. Caniformia	posterior ampulla	low	anterior	high	–	51-75%
V. Chiroptera	vestibule	high	anterior	high	–	51-75%
W. Microchiroptera	–	high	anterior	high	3+ turns	51-75%
X. *Rhinolophus*+*Tadarida*	–	high	anterior	high	3+ turns	51-75%
Y. Eulipotyphla	vestibule	high	anterior	–	1-2 turns	≤50%
Z. Euarchontoglires	vestibule	high	anterior	–	1-2 turns	51-75%
a. Glires	vestibule	high	anterior	high	1-2 turns	51-75%
b. Rodentia	vestibule	high	anterior	high	1-2 turns	51-75%

c. Lagomorpha	vestibule	high	anterior	high	1-2 turns	51-75%
d. Primatomorpha	vestibule	high	anterior	low	3+ turns	≤50%
e. Primates	vestibule	high	anterior	low	3+ turns	≤50%

[a] Letters in the first column refer to node labels in text Figure 2. Ancestral states reconstructed in Mesquite [94]. Definitions of characters: LSC Entry, entry of the posterior limb of the lateral semicircular canal into the secondary common crus, posterior ampulla, or vestibule; LSC Position, position of the plane of the lateral semicircular canal relative to the inferior limb of the posterior semicircular canal when the bony labyrinth is in anterior view; Largest Canal, largest semicircular canal arc radius of curvature among the anterior, lateral, and posterior semicircular canals; Cochlea Ratio, aspect ratio of cochlear spiral binned as either low (ratio equaling 0.55 or below) or high (ratio above 0.55), numerical values reported in text; Coiling, number of turns completed by the cochlea binned as 1-2 turns (360-720°), 2-3 turns (720-1080°), or 3+ turns (over 1080°), numerical values reported in text; % Cochlea, percent volume of the cochlea to total labyrinthine volume binned as ≤50%, 51-75%, or >75%, numerical values reported in text.
